# Ruthenium-Catalyzed C–H Alkenylation of Flavones with Alkenes: Chemoselective Synthesis and Mechanistic Insights from DFT Studies

**DOI:** 10.1021/acs.joc.6c00235

**Published:** 2026-06-23

**Authors:** Nathalia S. de Oliveira, Luana G. de Souza, Maria Eduarda C. L. de Oliveira, Marina A. Alves, Asier Carral-Menoyo, Nuria Sotomayor, Alcides J. M. da Silva

**Affiliations:** † Laboratório de Catálise Orgânica, Instituto de Pesquisas de Produtos Naturais, Universidade Federal do Rio de Janeiro, Ilha do Fundão, CCS, Bloco H − Sala H1-29, Rio de Janeiro, RJ 21941-599, Brazil; ‡ Departamento de Química Analítica, Instituto de Química, Centro de Tecnologia e Ciências, Universidade do Estado do Rio de Janeiro, Rua São Francisco Xavier 524, Pav. Haroldo Lisboa da Cunha − Maracanã, Rio de Janeiro, RJ 20550-900 Brazil; § Laboratório de Metabolômica Aplicada À Medicina de Sistemas (Meta2MS), Instituto de Pesquisas de Produtos Naturais, Universidade Federal do Rio de Janeiro, Rio de Janeiro, RJ 21941-598, Brazil; ∥ Departamento de Química Orgánica, Facultad de Ciencia y Tecnología, 16402 Universidad del País Vasco, Euskal Herriko Unibertsitatea UPV/EHU Apdo. 644, Bilbao 48080, Spain

## Abstract

A strategy for the selective C–H alkenylation of flavones is described. By using the carbonyl group of the flavone core as an intrinsic directing group, a series of 5-alkenylated derivatives were synthesized via coupling with alkenes, including acrylates, styrene, methyl vinyl ketone, and phenyl vinyl sulfone, affording the desired products in good to excellent yields (up to 92%). Notably, when a carbamate substituent was present at C7, the reaction proceeded with complete chemoselectivity, exclusively yielding C-5 alkenylated products. Although carbamate groups are widely recognized as efficient directing groups in alkenylation reactions, they proved to be ineffective within the flavone scaffold. Density functional theory (DFT) calculations were performed to rationalize the observed chemoselectivity and to provide mechanistic insight into the C–H activation process. The preference for C5 alkenylation arises from the lower energy profiles of the corresponding transition states and intermediates, whereas C8 functionalization is disfavored due to a greater tendency toward protodemetalation over migratory insertion.

## Graphical abstract

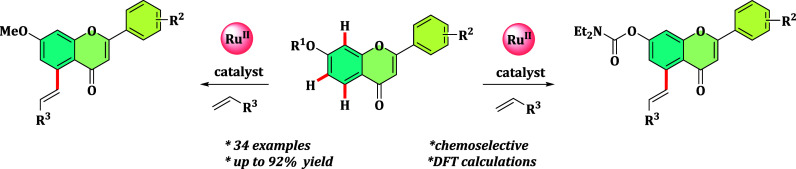

## Introduction

Flavonoids are a diverse class of polyphenolic compounds widely distributed in nature and play crucial roles in biological processes, including defense mechanisms, polymerization, growth regulation, and nitrogen fixation.^1^ They are categorized into several subgroups according to structural variations, such as anthocyanidins, aurones, flavanones, flavones, flavonols, flavanonols, chalcones, and isoflavones.^2–6^ Among these, flavones (2-phenylchromen-4-one) represent a prominent subclass that has attracted significant synthetic interest due to their broad spectrum of biological activities,^7^ including antioxidant,^8^ antiviral,^9^ anticancer,^10^ and anti-inflammatory^11^ properties. Of particular interest are flavones substituted at the C6 or C8 positions with *E*-propenoic acid moieties, several of which have recently been isolated and biologically evaluated (Figure 1). Torosaflavone E (**1**), demethyltorosaflavone (**2**), and 8-C-*E*-propenoic acid (**3**) were isolated from the ethanolic extract of *Alternanthera philoxeroides*, with compounds (**1**) and (**2**) displaying notable antidepressant activity.^12^ Likewise, luteolin 8-C-*E*-propenoic acid (**4**) was isolated from *Turnera diffusa*.^13^ Mimocaesalin D (**5**) and mimocaesalin C (**6**), obtained from *Mimosa caesalpiniifolia*, were also reported, with compound **6** exhibiting potent antifungal activity and marked selectivity against *Candida krusei* (IC_50_ = 44 nM).^14^

**Figure 1:**
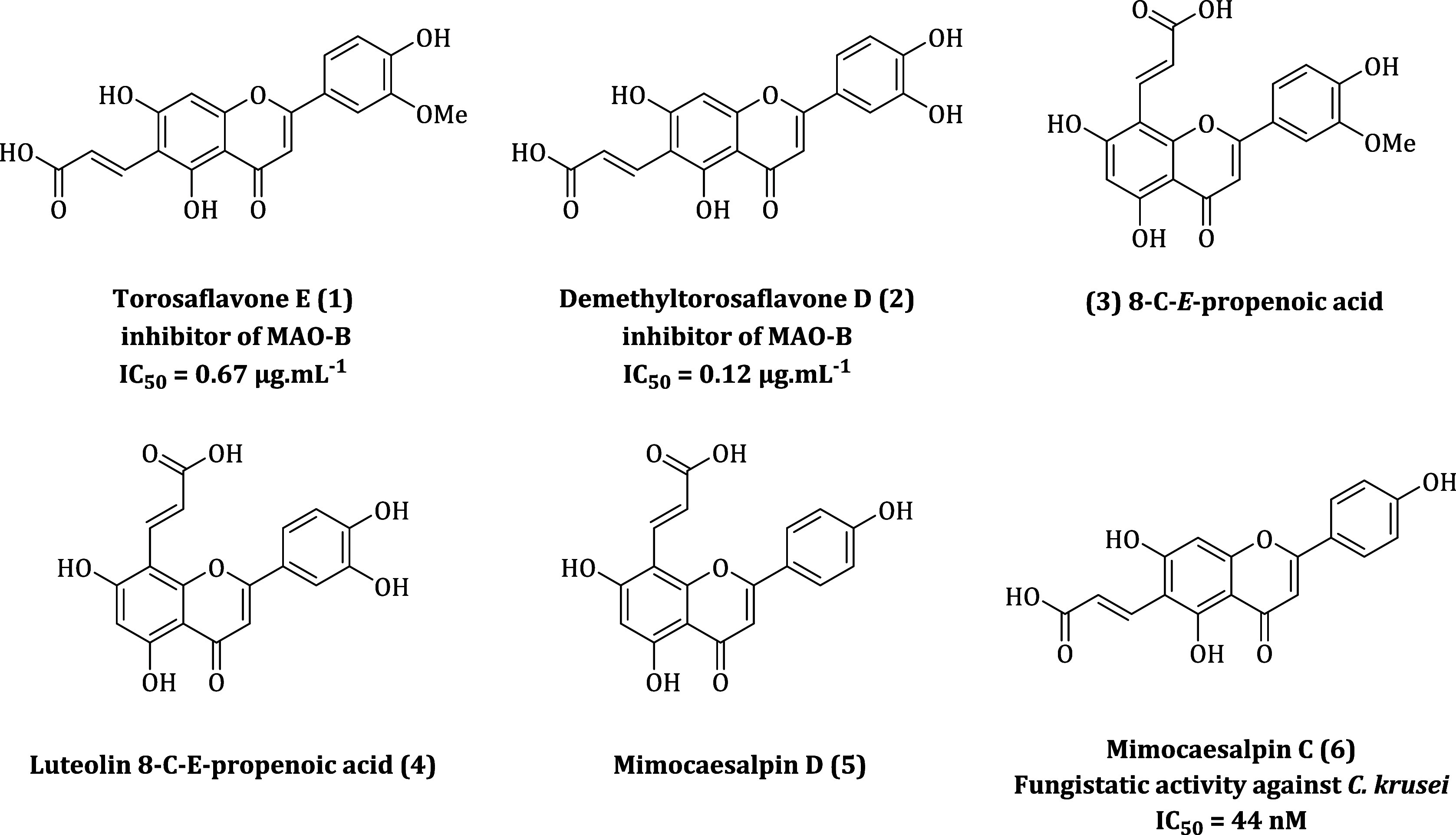
Torosoflavone (**1**), Demethyltorosaflavone (**2**), (E)-3-(5,7-dihydroxy-2-(4-hydroxy-3-methoxyphenyl)-4-oxo-4H-chromen-8-yl)­acrylic acid (**3**), Luteolin 8-C-*E*-propenoic acid (**4**), Mimocaesalpin D (**5**), and Mimocaesalpin C (**6**).

The direct functionalization of C–H bonds catalyzed by transition metals has emerged as a powerful and widely explored strategy in organic synthesis for the formation of C–C bonds.^15^ Among these transformations, alkenylation reactions of aromatic rings via C–H activation, often referred to as oxidative Heck-type reactions, have gained prominence as alternatives to the classical Heck coupling. These approaches eliminate the use of prefunctionalized aryl halides, which typically contain C–X or C–M bonds. The first example of C–C bond formation via palladium-catalyzed C–H bond cleavage was reported by Moritani and Fujiwara in the late 1960s. In this pioneering work, aryl-substituted olefins were synthesized through the reaction of olefins with benzene derivatives under oxidative conditions, starting the development of modern C–H bond functionalization strategies.^16^ This methodology provides a convenient approach for the construction and functionalization of various heterocyclic frameworks, including coumarins,^17^ quinolines,^18^ and chromanes.^19^ Beyond palladium complexes,^20^ a variety of other transition metals have been successfully employed in C–H activation reactions, including rhodium (Rh), ruthenium (Ru), iridium (Ir), and cobalt (Co).^21–24^ Notably, Murai and coworkers were pioneers in the development of olefination reactions of arenes containing directing groups, employing rhodium and ruthenium catalysts to promote site-selective C–H bond activation.^25^

The use of directing groups (DGs) has become essential to coordinate with the transition metal catalyst, guiding it toward specific C–H bonds based on favorable distance and geometric orientation, thus enabling differentiation among proximal sites.^26^ A wide range of directing groups has been reported in the literature, including aldehydes, ketones, carboxylic acids, esters, amides, pyridines, triazoles, and, notably, *O*-carbamates (−OCONR_2_). In the case of weakly coordinating directing groups, such as carbamates, the resulting metallacycles tend to be thermodynamically less stable, which often facilitates C–H functionalization due to reduced kinetic barriers. Moreover, carbamates offer additional advantages, including broad substrate compatibility, tolerance to diverse transformations, and effectiveness across multiple transition metal catalysts.^27^

The use of carbamate as directing group in alkenylation reactions was first reported independently by Feng and Gong,^28^ who demonstrated the regioselective *ortho*-olefination of carbamate-protected phenols with acrylates and styrenes via Rh­(III)-catalyzed C–H activation. Lutz Ackermann and coworkers expanded this strategy using Ru­(II) complexes to promote carbamate-directed *ortho*-olefinations.^29^ More recently, Reddy et al. described the oxidative *ortho*-alkenylation of phenyl carbamates with alkenes under catalytic conditions employing [RuCl_2_(*p*-cymene)]_2_, AgSbF_6_, and Cu­(OAc)_2_·H_2_O under ambient air (Scheme 1A).^30^

**Scheme 1:**
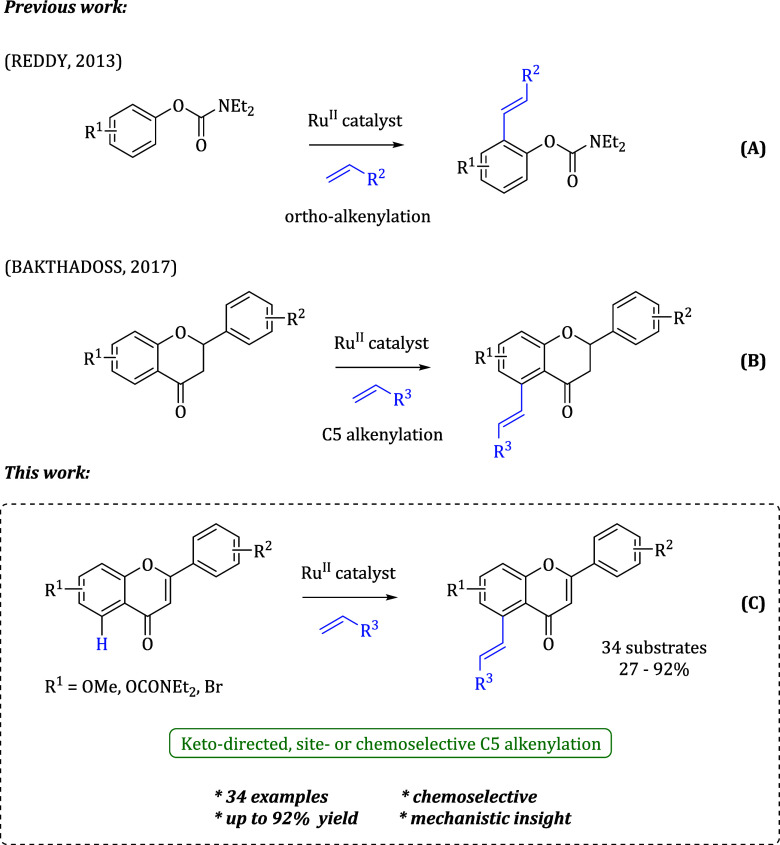
Ketone Directed Alkenylation of Flavones

Carbamate-directed site-selective C–H activation on flavone scaffolds has also been documented. Liu and coworkers described a palladium-catalyzed regioselective arylation at the C6 position of 7-*O*-carbamate flavones.^31^ In addition, C–H alkylation and alkenylation strategies have been applied to chromone derivatives.^32a,32b^ For instance, Kim’s group reported rhodium­(III)-catalyzed C–H functionalization of chromones with maleimides, affording a variety of succinimide derivatives.^32c^ Furthermore, Bakthadoss and coworkers described the synthesis of a C5-alkenylated chromane core through oxidative, keto-directed C–H activation catalyzed by ruthenium complexes using alkenes as coupling partners (Scheme 1B).^32d^ Herein, we report the coupling of 6- methoxy-, 7-methoxy-, 7-bromo-, and 7-*O*-carbamate flavones with alkenes such as acrylates, vinyl sulfones, and styrenes in the presence of [RuCl_2_(*p*-cymene)]_2_ (Scheme 1C). Moreover, density functional theory (DFT) calculations were performed to investigate the selectivity conferred by the carbamate and the ketone group and to obtain mechanistic insight into the reaction pathway.

## Results and Discussion

We initiated our studies by synthesizing C5-alkenylated flavones, inspired by the work of Bakthadoss.^32a^ The reaction of 7-methoxyflavone (**7a**) with methyl acrylate (**8a**) in the presence of [RuCl_2_(*p*-cymene)]_2_ (4 mol %), AgSbF_6_ (20 mol %), and Cu­(OAc)_2_·H_2_O (30 mol %) in 1,4-dioxane at 100 °C for 18 h afforded the desired product **9a** in 74% yield (Table 1, entry 1). Increasing the amount of Cu­(OAc)_2_·H_2_O to 2.2 equiv and conducting the reaction under a nitrogen atmosphere improved the yield to 89% (entry 2). Screening of alternative solvents, including DME, toluene, THF, and DCM, revealed no improvement over 1,4-dioxane (entries 3–6). Finally, reducing the catalyst loading of [RuCl_2_(*p*-cymene)]_2_ to 2.5 mol % resulted in a diminished yield of 66% (entry 7).

**Table 1:**
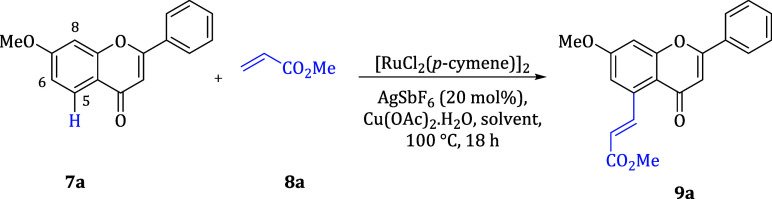
Optimization Studies for Keto-Directed C5 Alkenylation[Table-fn tbl1fn1]

Entry	Solvent	Oxidant	% Yield of 9a
1^a^	1,4-dioxane	Cu(OAc)_2_	**74**
2^b^	1,4-dioxane	Cu(OAc)_2_	**89**
3	DME	Cu(OAc)_2_	**50**
4	Toluene	Cu(OAc)_2_	**59**
5	THF	Cu(OAc)_2_	**63**
6	DCM	Cu(OAc)_2_	**69**
7^c^	1,4-dioxane	Cu(OAc)_2_	**66**

i All reactions were carried out under the following conditions: **7a** (1.0 equiv), **8a** (3.0 equiv), [RuCl_2_(*p*-cymene)]_2_ (4 mol %), additive (20 mol %), and Cu­(OAc)_2_.H_2_O in solvent at 100 °C for 18 h. [a] Cu­(OAc)_2_.H_2_O (30 mol %), under air. [b] Cu­(OAc)_2_.H_2_O (2.2 equiv), under N_2_. [c] [RuCl_2_(*p*-cymene)]_2_ (2.5 mol %).

To explore the reaction scope, [RuCl_2_(*p*-cymene)]_2_ (4 mol %), AgSbF_6_ (20 mol %), and Cu­(OAc)_2_·H_2_O (2.2 equiv) in 1,4-dioxane at 100 °C for 18 h were employed as the standard conditions (Scheme 2). The reaction of 7-methoxyflavone (**7a**) with ethyl acrylate (**8b**) and *n*-butyl acrylate (**8c**) afforded the corresponding C5-alkenylated flavones in 69% and 49% yield, respectively, whereas *t*-butyl acrylate (**8d**) failed to deliver the desired product. Other alkenes, including styrene (**8e**), methyl vinyl ketone (**8f**), and phenyl vinyl sulfone (**8g**), were also compatible. In contrast, acrylonitrile (**8h**), another electron-deficient alkene, did not undergo coupling, and no products were observed with sterically hindered or cyclic substrates such as maleic anhydride (**8i**).

**Scheme 2:**
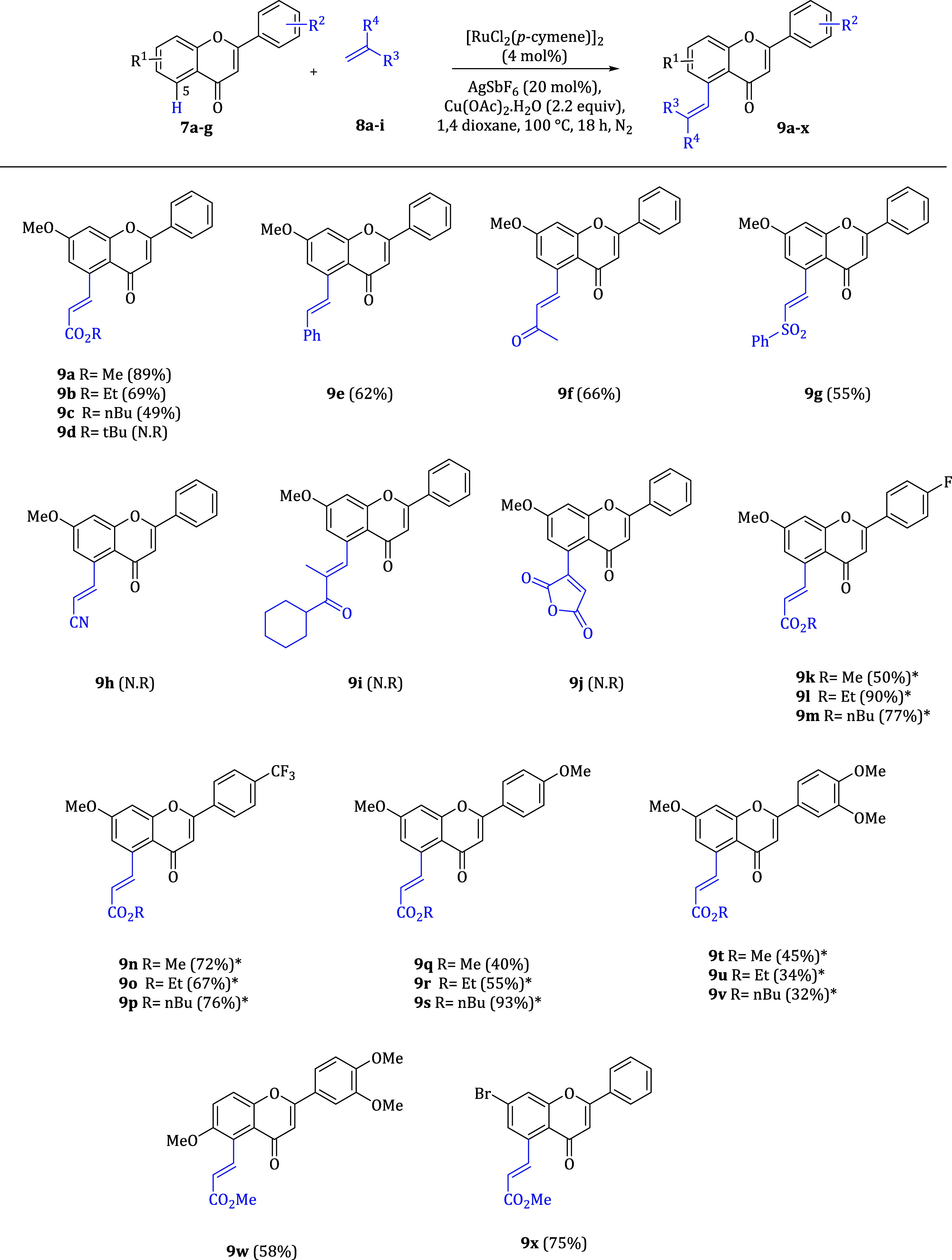
Scope of C5-Alkenylated Flavones^a^

a [*] DME solvent

We next examined the influence of substituents on the phenyl ring of the flavone in reactions with methyl, ethyl, and *n*-butyl acrylates. Flavone bearing 4-fluoro (**7b**) and 4-trifluoromethyl (**7c**) substituents was initially evaluated using dioxane as the solvent; however, this condition proved inefficient, and the starting materials were recovered. After changing the solvent to DME, flavone **7b** reacted with acrylates to afford the desired products in good to excellent yields (50–90%). Similarly, 4-trifluoromethyl-substituted flavone **7c** reacted efficiently with methyl acrylate, ethyl acrylate, and *n*-butyl acrylate, affording the corresponding products in 72% (**9n**), 67% (**9o**), and 76% (**9p**) yields, respectively. In contrast, methoxy-substituted flavone on the phenyl ring (**7d**) generally provided only moderate yields, except in the case of *n*-butyl acrylate (**9s**, 93%), which gave slightly improved efficiency. Flavone **7e,** bearing a 3,4-dimethoxy-substituted phenyl ring, afforded poor yields under the standard conditions.

We further investigated the effect of the methoxy substitution at the C6 position. Flavone **7f**, bearing a methoxy group on ring A at C6, afforded the desired product **9w** in good yield. Subsequently, we evaluated 7-bromoflavone (**7g**) as a substrate using 6.0 equivalents of methyl acrylate (**8a**), which afforded the corresponding C5-alkenylated flavone (**9x**) in a 75% yield. This halogenated substituent represents a valuable synthetic handle for future derivatization and cross-coupling transformations.

To the best of our knowledge, the alkenylation of flavones via C–H activation directed by a carbamate group has not been reported. We therefore investigated the influence of a 7-*O*-carbamate as a directing group. Initially, the coupling of 7-*O*-carbamate flavone **10a** with methyl acrylate **8a** under previously optimized Ru­(II) conditions proceeded sluggishly and required a prolonged reaction time. Inspired by Reddy et al.,^30^ DME was evaluated as the solvent, which exclusively afforded the C5-alkenylated product **11a** in 83% yield with remarkable chemoselectivity, as confirmed by GC-MS analysis.

Reaction at the C5 position was confirmed by ^1^H NMR spectroscopy, which showed a doublet with *J* = 2.3 Hz, consistent with *meta*-coupling between H6 and H8. Further confirmation was obtained using 2D NMR techniques (HMBC, HSQC, COSY). HMBC correlations of H6 (δH 7.2 ppm) with C8 (δC 112.1 ppm), as well as with the vinylic β-carbon (δC 144.2 ppm), unambiguously established the site of functionalization. These results suggest that, under the Ru­(II)-catalyzed conditions, the ketone group exhibits stronger directing ability than the carbamate.

Interestingly, these findings are in contrast with the results obtained by Reddy et al.^30^ According to their work, even in the presence of formyl or methoxycarbonyl substituents, C–H activation occurred selectively *ortho* to the carbamate group, showcasing the stronger directing effect of that moiety. Similarly, Liu et al.^31^ reported the Pd-catalyzed C–H arylation of flavones directed by a 7-*O*-carbamate, which selectively occurred at C6.

The scope of the reaction was then evaluated using 7-*O*-carbamate flavone derivatives **10a**–**e** with various acrylates **8a**–**g** under the optimized conditions (Scheme 3). Ethyl acrylate (**8b**) and *n*-butyl acrylate (**8c**) furnished the corresponding C5-alkenylated products **11b** and **11c** in 75% and 92% yield, respectively. Notably, the reaction with ethyl acrylate was more efficient when Cu­(OAc)_2_·H_2_O was used in catalytic amounts. In contrast, *tert*-butyl acrylate (**8d**) did not afford any product, likely due to steric hindrance, which may inhibit either olefin coordination or subsequent migratory insertion.

**Scheme 3:**
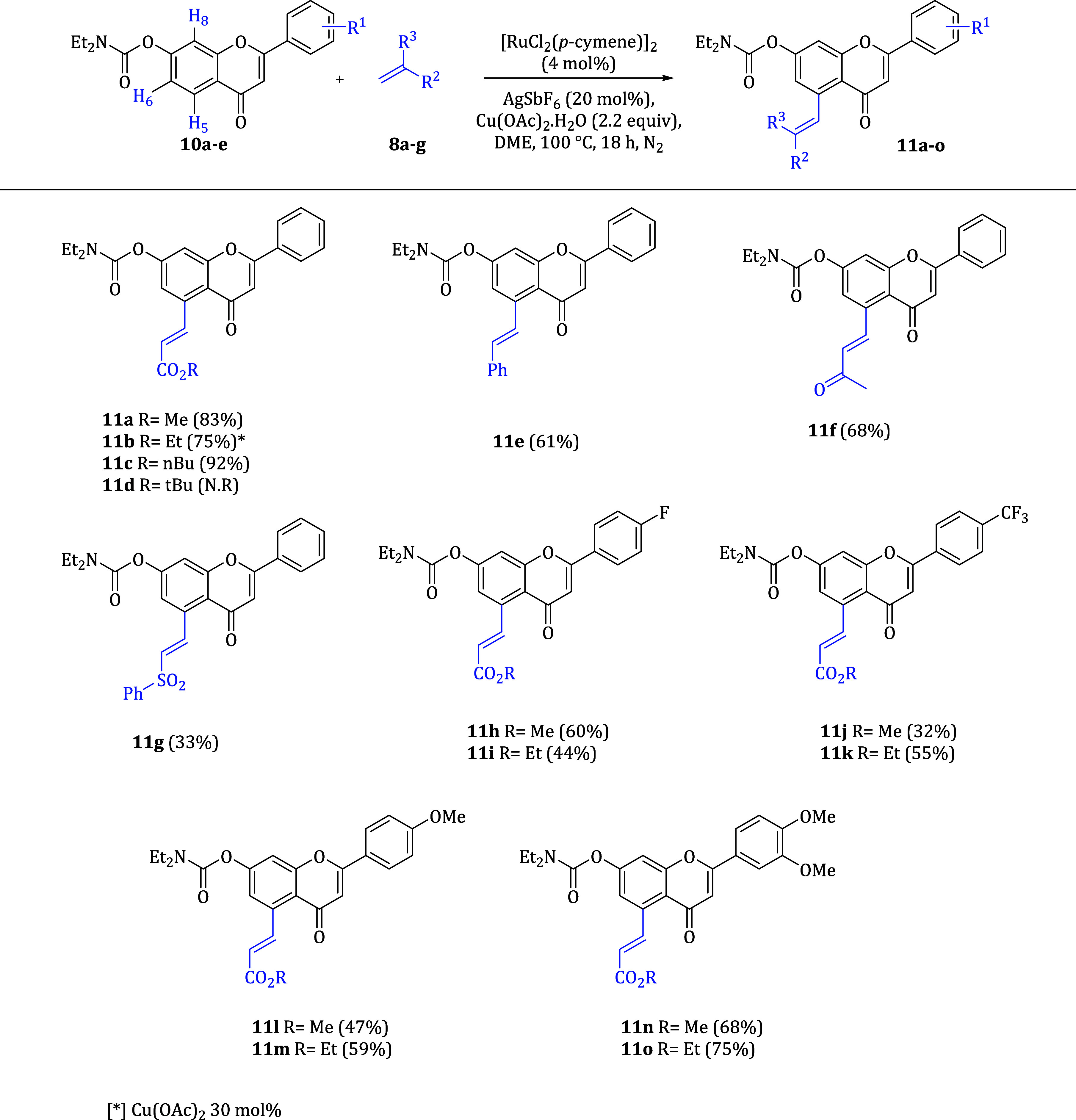
Scope of 5-Alkenylated 7-*O*-Carbamate Flavones

Other alkenes were also examined. Styrene (**8e**) and methyl vinyl ketone (**8f**) reacted efficiently, giving **11e** and **11f** in 61% and 68% yields, respectively. Phenyl vinyl sulfone (**8g**) afforded **11g** in moderate yield, consistent with previous observations for C5-alkenylation of 7-methoxyflavone, likely due to the strong electron-withdrawing nature of the sulfone group, which reduces reactivity toward coordination or migratory insertion. Acrylonitrile and maleic anhydride failed to produce alkylated products under these conditions.

The effect of substituents on the phenyl ring was also investigated using methyl and ethyl acrylates. Flavone bearing a 4-fluoro substituent (**10b**) provided moderate yields (44–60%). 4-Trifluoromethyl-substituted flavone **10c** afforded **11j** and **11k** in 32% and 55% yield, respectively, reflecting a reduced reactivity, surely due to the presence of strongly electron-withdrawing groups. In contrast, electron-donating substituents generally improved efficiency: the 4-methoxy-substituted flavone **10d** gave **11l** and **11m** in 47% and 59% yield, while the 3,4-dimethoxy substituted flavone **10e** afforded **11n** and **11o** in 68% and 75% yield. These results indicate that electron-donating groups enhance the efficiency of C5-selective alkenylation.

To assess the practicality of this strategy, a scale-up reaction was performed on a 1 mmol scale with **10a** using methyl acrylate **8a** as a coupling partner and affording flavone **11a** in 82% yield. The result was consistent with that of the small-scale reaction, confirming the robustness and scalability of the method.

With the experimental results in hand, we decided to carry out DFT studies to elucidate the mechanism operating in the reaction. All structures were optimized using density functional theory (DFT) as implemented in Gaussian 16,^33^ with B3LYP^34^ as the functional, 6–31G­(d,p) as basis set for nonmetallic atoms, and LANL2DZ^35^ as basis set for ruthenium. Final energies were obtained by performing single-point calculations on the previously optimized structures at M06 ^36^/6–311++G­(d,p) level of theory for nonmetallic atoms and the SDD basis set for ruthenium,^37^ introducing solvation factors with the IEF-PCM^38^ method, and 1,4-dioxane as the solvent. The stationary points were characterized by frequency calculations in order to verify that they had the correct number of imaginary frequencies.

First, some studies were conducted to explain the regioselectivity of the reaction. According to the experimental results, the Ru­(II)-catalyzed alkenylation described herein proceeds selectively at the C5 position, indicating a coordination of the Ru­(II) center to the ketone, surpassing coordination to the carbamate directing group. To study the mentioned selectivity, first, the most favored carbamate-directed position was checked (C8 activation vs C6 activation). The calculated results showed that C–H activation at C8 would be favored by 3.0 kcal/mol compared to the also possible C6 position (Figure 2a).

**Figure 2:**
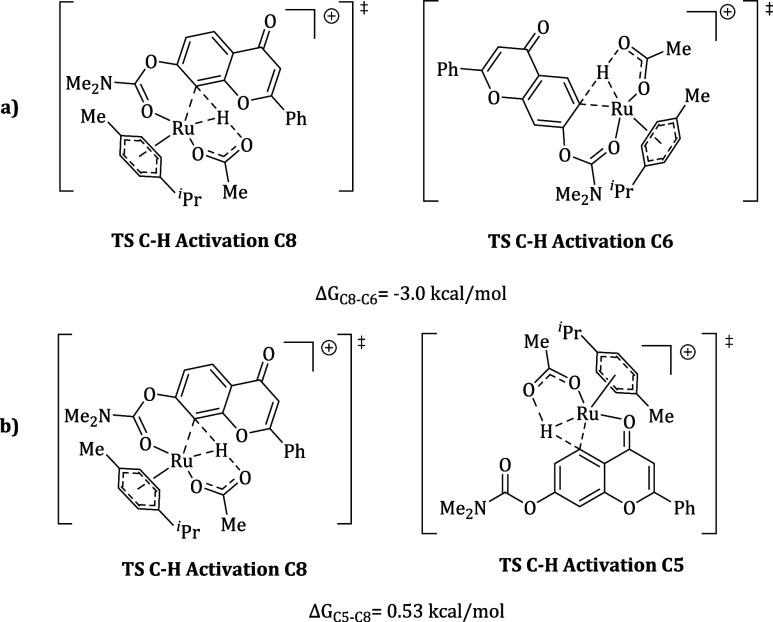
DFT studies on the most favored position for C–H activation.

Once the favored carbamate-directed position was determined, it was compared with the ketone-directed C5 position (Figure 2b). In agreement with the experimental results, C–H activation at C5 would be favored by 0.53 kcal/mol. However, this small difference would not explain complete selectivity toward alkenylation at that position, and therefore, C–H activation and subsequent migratory insertion steps were studied for both coordination sites.

First, the reaction mechanism for the C8 alkenylation was calculated (Scheme 4, Red Pathway), taking complex **A**_**Ket**_ as the starting point, where the Ru­(II) center is coordinated to the ketone. The metal would initially coordinate to the carbamate moiety to generate **A**_**Carb**_ (ΔG = 4.8 kcal/mol). Afterward, a reversible C–H activation would take place with an activation energy of 22.7 kcal/mol starting from **A**_**Ket**_. This process would render complex **B**_**Carb**_, which, after ligand exchange, would generate **C**_**Carb**_, containing the methyl acrylate coupling partner. Then, a rate-determining migratory insertion would occur (ΔG‡ = 10.9 kcal/mol) to form complex **D**_**Carb**_. This study showed that C–H activation is highly reversible, since proto-demetalation from **B**_**Carb**_ to give complex **A**_**Carb**_ has an activation energy of 10.3 kcal/mol, while 11.5 kcal/mol are required for migratory insertion to proceed.

**Scheme 4:**
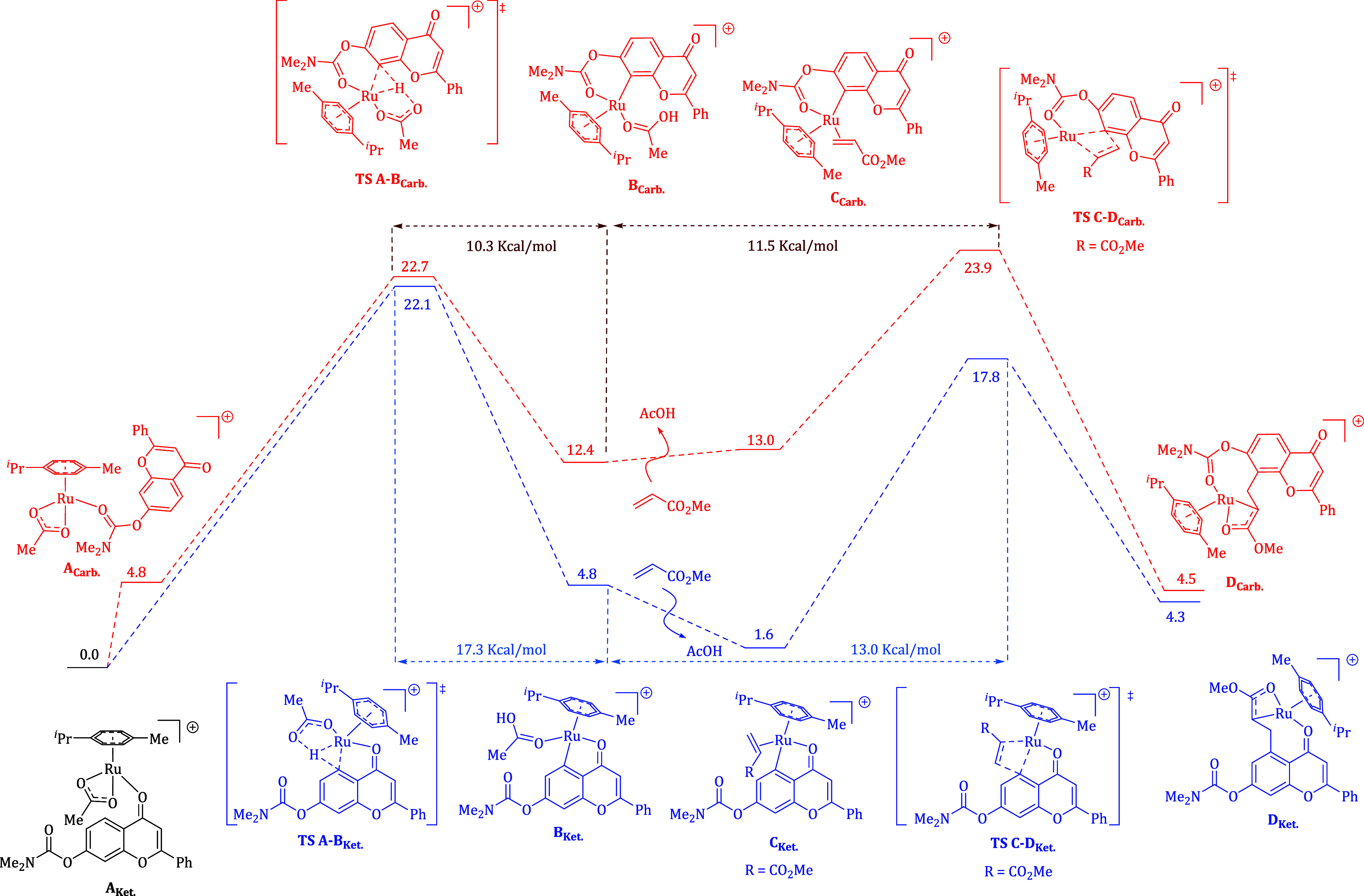
Free-Energy Profile of Ru­(II)-Catalyzed Alkenylation of Flavones at C8 vs C5 Characterized at M06/6–311++G­(d,p) Level of Theory (Energy Values Expressed in kcal/mol)

Considering this data, we then calculated the C–H activation and migratory insertion steps for the ketone-directed process (Scheme 4, Blue Pathway). According to the results, after a rate-determining and reversible C–H activation process (ΔG‡ = 22.1 kcal/mol), complex **B**_**Ket**_ would be generated, which is much more stable than its carbamate counterpart **B**_**Carb**_ (ΔG_Ket.‑Carb._ = −7.6 kcal/mol). The increased stability of this complex makes the proto-demetalation step to recover **A**_**Ket**_ less favorable (ΔG‡ = 17.3 kcal/mol). After ligand exchange to incorporate the methyl acrylate coupling partner, a reversible, non-rate-determining migratory insertion is proposed to take place with an activation energy of 16.2 kcal/mol to give complex **D**_**Ket**_.

In summary, the experimentally observed preference toward alkenylation at C5 would be explained by the fact that the proposed transition states and intermediates for the C5-alkenylation are, in all cases, more stable than the C8-alkenylation counterparts. In addition, for the C8 alkenylation, the protodemetalation step to recover the initial complex **A**_**Carb**_ has a lower activation barrier (10.3 kcal/mol) than the energy required for the transformation to proceed to the migratory insertion step (11.5 kcal/mol). This scenario is not observed for the C5 alkenylation. In this case, protodemetalation from **B**_**Ket**_ to regenerate **A**_**Ket**_ has an activation energy of 17.3 kcal/mol, while 13.0 kcal/mol is required to achieve migratory insertion.

Finally, in order to confirm the reversibility of the C–H activation step, deuterium exchange experiments were carried out (Scheme 5). The results obtained are consistent with the calculated mechanistic pathways, as the same degree of deuterium incorporation was observed for positions C5 and C8, which have very similar activation energies for the C–H activation step. Besides, deuterium incorporation was considerably lower at C6, where C–H activation is more energetically demanding.

**Scheme 5:**
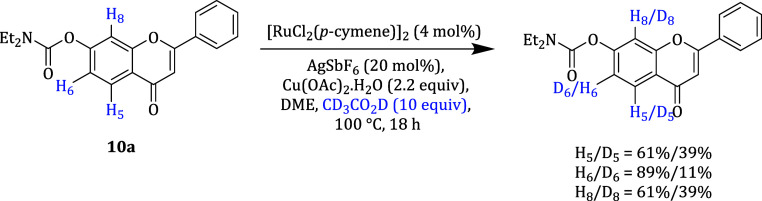
Deuterium Incorporation in Flavone **10a**

As observed in computational studies, the C8 alkenylation should not be favored, even when the reaction at C5 is not possible due to the reversible C–H activation and the rate-determining migratory insertion. To corroborate this hypothesis, different substituents were placed at C5 in order to block the position, and in all cases, no reactivity was observed at all (Scheme 6).

**Scheme 6:**
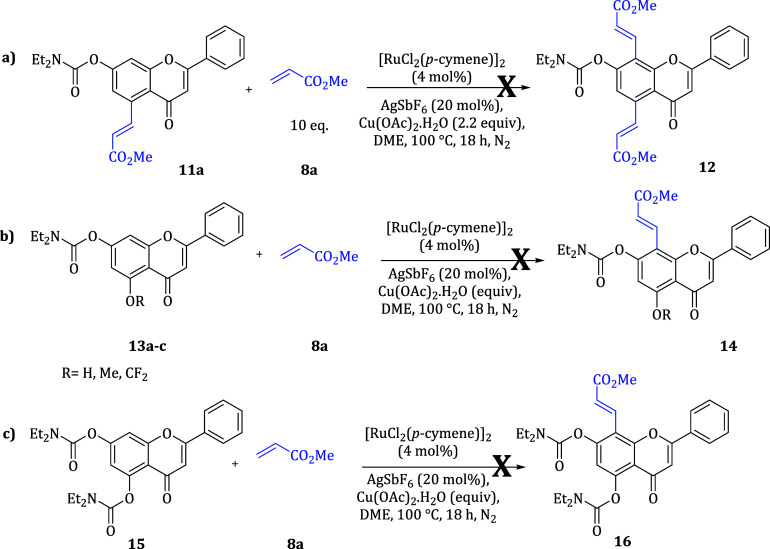
Experiments Carried Out Blocking C5 Position

First, **10a** was treated with an excess of methyl acrylate (10 equiv), and separately, exposure of monoalkenylated flavone **11a** to the same conditions was carried out. Those experiments failed to deliver any dialkenylated product, obtaining, in both cases, only the monoalkenylated flavone **11a**, indicating limited reactivity for further C–H activation (Scheme 6a), in agreement with DFT studies. We then tested chrysin derivatives **13a**–**c** (Scheme 6b) under the previously established Ru­(II)-catalyzed reaction conditions, as well as 5,7-dicarbamate flavone **15** (Scheme 6c), recovering, in all cases the corresponding starting material.

Once with all those results in hand, we proposed the following catalytic cycle for the Ru­(II)-catalyzed C5 alkenylation of flavones (Scheme 7). First of all, the reaction of the Ru­(II) precatalyst [RuCl_2_(*p*-cymene)]_2_ would generate the active catalytic species that would coordinate to a molecule of the substrate **10a** generating complex **A**_**Ket**_. This species would undergo a reversible and rate-determining C–H activation process to give **B**_**Ket**_, which, after release of AcOH and incorporation of the methyl acrylate coupling partner, is proposed to form **C**_**Ket**_. After reversible and non-rate-determining migratory insertion, enolate **D**_**Ket**_ would be formed. Subsequent β-hydride elimination would generate complex **E**_**Ket**_, which contains the experimentally observed C5-alkenylated product, releasing it after ligand exchange with acetate. Ruthenium­(II)-hydride complex **F** would undergo reductive elimination to give a Ru(0) complex **G** that regenerates the initial complex **A**_**Ket**_ after oxidation and coordination of a molecule of **10a**.

**Scheme 7:**
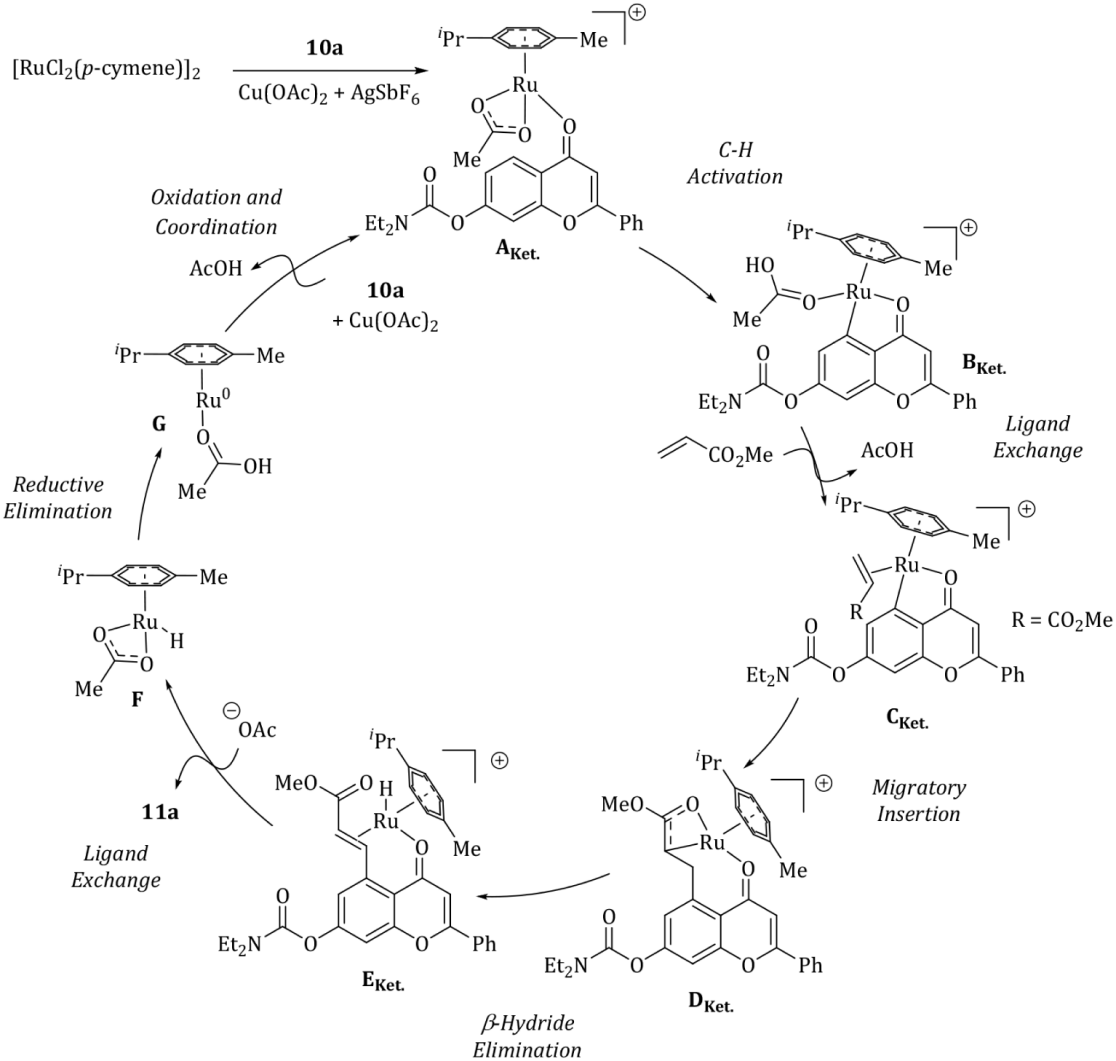
Proposed Catalytic Cycle

## Conclusion

This study demonstrates that Ru­(II)-catalyzed C–H alkenylation enables efficient and selective functionalization of the C5 position in flavonoid-derived scaffolds using a ketone directing group. The reaction tolerates a broad range of coupling partners, including acrylates, styrene, methyl vinyl ketone, and phenyl vinyl sulfones, as well as both electron-donating and electron-withdrawing substituents on the phenyl ring. The computational DFT studies support the reactivity of the system and the proposed mechanism. These results underscore the versatility of this method and its potential for the late-stage modification of bioactive flavonoids.

## Experimental Section

### General Information

All reagents and solvents were purchased from Sigma-Aldrich/Merck and TCI and were used without further purification unless otherwise noted. The progress of reactions was monitored by TLC, and flash column chromatography was carried out on silica gel 60 (SiliCycle, 230–400 mesh). Analytical thin-layer chromatography (TLC) was performed using precoated TLC sheets of silica gel 60 F254 (SiliCycle), and the spots were visualized under UV light (254 and 365 nm). Known compounds were synthesized according to previously reported procedures (see Supporting Information). The alkenes were obtained from commercial sources and used without further purification. NMR spectra were recorded on Varian NMR spectrometers (400 or 500 MHz for ^1^H; 101 or 126 MHz for ^13^C) using CDCl_3_ as the solvent and TMS as an internal standard at room temperature. Chemical shifts (δ) are given in ppm and calibrated using the signal of tetramethylsilane (TMS) as the internal reference (δH and δC = 0 ppm). Data are reported as follows: chemical shift (multiplicity, coupling constant(s), integration). Coupling constants were quoted to the nearest 0.1 Hz, and multiplicity was reported according to standard abbreviations. Structural assignments were made with additional information from gCOSY, gHSQC, and gHMBC experiments. High-resolution mass spectrometric analyses were performed on a Q Exactive hybrid quadrupole–Orbitrap mass spectrometer (Thermo Fisher Scientific, Bremen, Germany) equipped with an electrospray ionization (ESI) source operating in positive ion mode. The melting points were obtained on a Fisatom 430 and are uncorrected.

### General Procedures and Characterization Data

#### General Procedure for the Ru­(II)-Catalyzed C–H Alkenylation of Flavones

In a reaction vial, flavone (0.10 mmol, 1.0 equiv), [RuCl_2_(*p*-cymene)]_2_ (0.004 mmol, 4 mol %), Cu­(OAc)_2_·H_2_O (0.22 mmol, 2.2 equiv), and AgSbF_6_ (0.020 mmol, 20 mol %) were added. Note: AgSbF_6_ was handled inside a nitrogen-filled glovebag due to its sensitivity to moisture. The alkene coupling partner (3.0 equiv) and the solvent (1 mL of either 1,2-dimethoxyethane or 1,4-dioxane) were then added. The reaction mixture was stirred at 100 °C in an oil bath under a nitrogen atmosphere for 18 h. After cooling to room temperature, the reaction mixture was diluted with ethyl acetate and filtered through a short pad of Celite. The filtrate was washed with saturated aqueous ammonium chloride, dried over anhydrous sodium sulfate, and concentrated under reduced pressure. The crude product was purified by silica gel column chromatography using a gradient of hexanes and ethyl acetate as the eluent to afford the desired alkenylated flavone.

#### Methyl (E)-3-(7-methoxy-4-oxo-2-phenyl-4H-chromen-5-yl)­acrylate (**9a**)

Following General Procedure, the compound was prepared from 7-methoxy-2-phenyl-4H-chromen-4-one (**7a**) (25.2 mg, 0.1 mmol, 1 equiv) and methyl acrylate (**8a**) (25.8 mg, 0.3 mmol, 3.0 equiv) in 1,4-dioxane (1 mL) and it was purified using columm chromatography on silica gel (AcOEt:Hex = 2:8) as yield as yellow solid, 30 mg, 89% yield. MP: 155–157 °C. ^1^H NMR (500 MHz, CDCl_3_) δ 8.99 (d, *J* = 15.9 Hz, 1H), 7.91 – 7.87 (m, 2H), 7.54 – 7.50 (m, 3H), 7.01 (d, *J* = 2.4 Hz, 1H), 7.00 (d, *J* = 2.4 Hz, 1H), 6.71 (s, 1H), 6.24 (d, *J* = 15.9 Hz, 1H), 3.94 (s, 3H), 3.83 (s, 3H).^13^C­{^1^H} NMR (126 MHz, CDCl_3_) δ 178.9, 166.9, 162.9, 161.8, 159.0, 144.8, 138.3, 131.5, 131.3, 129.0, 126.1, 121.2, 115.6, 113.5, 108.6, 101.9, 55.9, 51.8. HRMS (ESI) *m*/*z* [M + H]^+^ calcd. for C_20_H_16_O_5_ 337.0998; found: 337.1069.

#### Ethyl (E)-3-(7-methoxy-4-oxo-2-phenyl-4H-chromen-5-yl)­acrylate (**9b**)

Following General Procedure, the compound was prepared from 7-methoxy-2-phenyl-4H-chromen-4-one (**7a**) (25.2 mg, 0.1 mmol, 1 equiv) and ethyl acrylate (**8b**) (30.0 mg, 0.3 mmol, 3.0 equiv) in 1,4-dioxane (1 mL). It was purified with columm chromatography on silica gel (AcOEt:Hex = 2:8) as a white solid, 24 mg, 69% yield. MP: 157–159 °C. ^1^H NMR (400 MHz, CDCl_3_) δ 8.98 (d, *J* = 15.9 Hz, 1H), 7.90 – 7.85 (m, 2H), 7.55 – 7.49 (m, 3H), 7.02 – 6.96 (m, 2H), 6.70 (s, 1H), 6.23 (d, *J* = 15.9 Hz, 1H), 4.29 (q, 2H), 3.93 (s, 3H), 1.36 (t, 3H). ^13^C­{^1^H} NMR (126 MHz, CDCl_3_) δ 178.9, 166.5, 162.8, 161.8, 159.0, 144.5, 138.4, 131.5, 131.3, 129.0, 126.1, 121.7, 115.6, 113.5, 108.6, 101.8, 60.6, 55.9, 14.3. HRMS (ESI) *m*/*z* [M + H]^+^ calcd. for C_21_H_18_O_5_ 351.1154, found 351.1230.

#### *N*-Butyl (E)-3-(7-methoxy-4-oxo-2-phenyl-4H-chromen-5-yl)­acrylate (**9c**)

Following General Procedure, the compound was prepared from 7-methoxy-2-phenyl-4H-chromen-4-one (**7a**) (25.2 mg, 0.1 mmol, 1 equiv) and *n*-butyl acrylate (**8c**) (38.4 mg, 0.3 mmol, 3.0 equiv) in 1,4-dioxane (1 mL), and it was purified with columm chromatography on silica gel (AcOEt:Hex = 2:8) to yield a white solid, 17 mg, 49% yield. MP: 155–157 °C. ^1^H NMR (500 MHz, CDCl_3_) δ 9.00 (d, *J* = 15.9 Hz, 1H), 7.92 – 7.88 (m, 2H), 7.54 – 7.50 (m, 3H), 7.04 – 6.99 (m, 2H), 6.72 (s, 1H), 6.24 (d, *J* = 15.9 Hz, 1H), 4.24 (t, 2H), 3.95 (s, 3H), 1.75 – 1.70 (m, 2H), 1.51 – 1.43 (m, 2H), 0.98 (d, 3H). ^13^C­{^1^H} NMR (126 MHz, CDCl_3_) δ 178.9, 166.6, 162.9, 161.8, 159.0, 144.5, 138.5, 131.5, 131.4, 129.0, 126.1, 121.7, 115.6, 113.5, 108.6, 101.9, 64.5, 55.9, 30.8, 19.2, 13.8. HRMS (ESI) *m*/*z* [M + H]^+^ calcd. for C_23_H_22_O_5_ 379.1467, found 379.1545.

#### (E)-7-methoxy-2-phenyl-5-styryl-4H-chromen-4-one (**9e**)

Following General Procedure, the compound was prepared from 7-methoxy-2-phenyl-4H-chromen-4-one (**7a**) (25.2 mg, 0.1 mmol, 1 equiv) and styrene (**8e**) (31.2 mg, 0.3 mmol, 3.0 equiv) in 1,4-dioxane (1 mL). It was purified with columm chromatography on silica gel (AcOEt:Hex = 2:8) as a yellow solid, 22 mg, 62% yield. MP: 167–169 °C. ^1^H NMR (400 MHz, CDCl_3_) δ 8.70 (d, *J* = 16.2 Hz, 1H), 7.92 – 7.87 (m, 2H), 7.64 – 7.59 (m, 2H), 7.51 (m, 3H), 7.40 – 7.34 (m, 2H), 7.30 – 7.25 (m, 1H), 7.16 (d, *J* = 2.5 Hz, 1H), 6.98 (d, *J* = 16.2 Hz, 1H), 6.90 (d, *J* = 2.5 Hz, 1H), 6.69 (s, 1H), 3.94 (s, 3H). ^13^C­{^1^H} NMR (101 MHz, CDCl_3_) δ 179.6, 162.8, 161.2, 159.3, 141.5, 137.4, 131.92, 131.5, 131.3, 129.0, 128.6, 128.3, 127.9, 127.1, 126.1, 115.2, 111.6, 108.8, 100.1, 55.8. HRMS (ESI) *m*/*z* [M + H]^+^ calcd. for C_24_H_18_O_3_ 355.1256, found 355.1333.

#### (E)-7-methoxy-5-(3-oxobut-1-en-1-yl)-2-phenyl-4H-chromen-4-one (**9f**)

Following General Procedure, the compound was prepared from 7-methoxy-2-phenyl-4H-chromen-4-one (**7a**) (25.2 mg, 0.1 mmol, 1 equiv) and methyl vinyl ketone (**8f**) (21.0 mg, 0.3 mmol, 3.0 equiv) in 1,4-dioxane (1 mL), and it was purified with columm chromatography on silica gel (AcOEt:Hex = 2:8) as brown solid, 21 mg, 66% yield. MP: 160–162 °C. ^1^H NMR (400 MHz, CDCl_3_) δ 8.99 (d, *J* = 16.4 Hz, 1H), 7.92 – 7.87 (m, 2H), 7.57 – 7.50 (m, 3H), 7.05 (d, *J* = 2.5, 1H), 7.01 (d, *J* = 2.5 Hz, 1H), 6.71 (s, 1H), 6.44 (d, *J* = 16.4 Hz, 1H), 3.94 (s, 3H), 2.49 (s, 3H). ^13^C­{^1^H} NMR (101 MHz, CDCl_3_) δ 199.7, 179.2, 162.9, 162.0, 159.1, 144.2, 138.4, 131.6, 131.2, 131.1, 129.1, 126.1, 115.4, 113.2, 108.6, 102.2, 55.9, 26.3. HRMS (ESI) *m*/*z* [M + H]^+^ calcd. for C_20_H_16_O_4_ 321.1049, found 321.1127.

#### (E)-7-methoxy-2-phenyl-5-(2-(phenylsulfonyl)­vinyl)-4H-chromen-4-one (**9g**)

Following General Procedure, the compound was prepared from 7-methoxy-2-phenyl-4H-chromen-4-one (**7a**) (25.2 mg, 0.1 mmol, 1 equiv) and phenyl vinyl sulfone (**8g**) (50.4 mg, 0.3 mmol, 3.0 equiv) in 1,4-dioxane (1 mL), and it was purified with columm chromatography on silica gel (AcOEt:Hex = 2:8) as yellow solid, 23 mg, 55% yield. MP: 164–166 °C. ^1^H NMR (400 MHz, CDCl_3_) δ 8.95 (d, *J* = 15.3 Hz, 1H), 8.12 – 8.09 (m, 2H), 7.90 – 7.87 (m, 2H), 7.67 – 7.47 (m, 6H), 7.02 (d, *J* = 2.5 Hz, 1H), 6.94 (d, *J* = 2.5 Hz, 1H), 6.72 (s, 1H), 6.66 (d, *J* = 15.3 Hz, 1H), 3.93 (s, 3H). ^13^C­{^1^H} NMR (101 MHz, CDCl_3_) δ 178.5, 162.9, 162.2, 158.9, 144.0, 140.5, 136.2, 133.3, 131.6, 131.2, 130.0, 129.3, 129.1, 128.0, 126.1, 115.7, 113.9, 108.4, 102.4, 56.0. HRMS (ESI) *m*/*z* [M + Na]^+^calcd. for C_24_H_18_O_5_S 441.0875, found 441.0776.

#### Methyl (E)-3-(2-(4-fluorophenyl)-7-methoxy-4-oxo-4H-chromen-5-yl)­acrylate (**9k**)

Following General Procedure, the compound was prepared from 2-(4-fluorophenyl)-7-methoxy-4H-chromen-4-one (**7b**) (27.0 mg, 0.1 mmol, 1 equiv) and methyl acrylate (**8a**) (25.8 mg, 0.3 mmol, 3.0 equiv) in DME (1 mL), and it was purified with columm chromatography on silica gel (AcOEt:Hex = 2:8) as white solid, 17 mg, 50% yield. MP: 195–197 °C. ^1^H NMR (400 MHz, CDCl_3_) δ 8.96 (d, *J* = 15.9 Hz, 1H), 7.93 – 7.86 (m, 2H), 7.21 (dd, *J* = 9.4, 7.8 Hz, 2H), 7.01 (d, *J* = 2.5 Hz, 1H), 6.99 (d, *J* = 2.5 Hz, 1H), 6.65 (s, 1H), 6.24 (d, *J* = 15.9 Hz, 1H), 3.95 (s, 3H), 3.83 (s, 3H). ^13^C­{^1^H} NMR (126 MHz, CDCl_3_) δ 178.7, 166.9, 165.7, 163.7, 162.9, 160.9, 159.0, 144.7, 138.4, 128.4, 128.3, 121.3, 116.4, 116.2, 115.5, 113.5, 108.4, 101.9, 55.9, 51.8. HRMS (ESI) *m*/*z* [M + H]^+^ calcd. for C_20_H_15_FO_5_ 355.0904, found 355.0980.

#### Ethyl (E)-3-(2-(4-fluorophenyl)-7-methoxy-4-oxo-4H-chromen-5-yl)­acrylate (**9l**)

Following General Procedure, the compound was prepared from 2-(4-fluorophenyl)-7-methoxy-4H-chromen-4-one (**7b**) (27.0 mg, 0.1 mmol, 1 equiv) and ethyl acrylate (**8b**) (30.0 mg, 0.3 mmol, 3.0 equiv) in DME (1 mL) and it was purified with columm chromatography on silica gel (AcOEt:Hex = 2:8) as white solid, 33 mg, 90% yield. MP: 189–191 °C. ^1^H NMR (500 MHz, CDCl_3_) δ 8.97 (d, *J* = 15.9 Hz, 1H), 7.91 – 7.87 (m, 2H), 7.23 – 7.19 (m, 2H), 7.01 (d, *J* = 2.5 Hz, 1H), 6.98 (d, *J* = 2.5 Hz, 1H), 6.65 (s, 1H), 6.24 (d, *J* = 15.9 Hz, 1H), 4.30 (q, 2H), 3.94 (s, 3H), 1.36 (t, 3H). ^13^C­{^1^H} NMR (126 MHz, CDCl_3_) δ 178.7, 166.4, 165.7, 163.7, 162.9, 160.8, 158.9, 144.4, 138.5, 128.34, 128.27, 121.8, 116.4, 116.2, 115.5, 113.5, 108.4, 101.8, 60.6, 55.9, 14.3. HRMS (ESI) *m*/*z* [M + H]^+^ calcd. for C_21_H_17_FO_5_ 369.1060, found 369.1140.

#### *N*-Butyl (E)-3-(2-(4-fluorophenyl)-7-methoxy-4-oxo-4H-chromen-5-yl)­acrylate (**9m**)

Following General Procedure, the compound was prepared from 2-(4-fluorophenyl)-7-methoxy-4H-chromen-4-one (**7b**) (27.0 mg, 0.1 mmol, 1 equiv) and *n*-butyl acrylate (**8c**) (38.4 mg, 0.3 mmol, 3.0 equiv) in DME (1 mL), and it was purified with columm chromatography on silica gel (AcOEt:Hex = 3:7) as yellow solid, 30.5 mg, 77% yield. MP: 157–159 °C. ^1^H NMR (500 MHz, CDCl_3_) δ 8.97 (d, *J* = 15.9 Hz, 1H), 7.91 – 7.85 (m, 2H), 7.23 – 7.18 (m, 2H), 7.01 (d, *J* = 2.5, 1H), 6.97 (d, *J* = 2.5 Hz, 1H), 6.65 (s, 1H), 6.24 (d, *J* = 15.9 Hz, 1H), 4.24 (t, 2H), 3.94 (s, 3H), 1.75 – 1.70 (m, 2H), 1.50 – 1.43 (m, 2H), 0.98 (t, 3H). ^13^C­{^1^H} NMR (126 MHz, CDCl_3_) δ 178.7, 166.5, 165.7, 163.7, 162.9, 160.8, 158.9, 144.4, 138.5, 128.3, 128.3, 121.8, 116.3, 116.2, 115.4, 113.5, 108.3, 101.8, 64.5, 55.9, 30.8, 19.2, 13.8. HRMS (ESI) *m*/*z* [M + H]^+^ calcd. for C_23_H_21_FO_5_ 397.1373, found 397.1449.

#### Methyl (E)-3-(7-methoxy-4-oxo-2-(4-(trifluoromethyl)­phenyl)-4H-chromen-5-yl)­acrylate (**9n**)

Following General Procedure, the compound was prepared from 7-methoxy-2-(4-(trifluoromethyl)­phenyl)-4H-chromen-4-one (**7c**) (32.0 mg, 0.1 mmol, 1 equiv) and methyl acrylate (**8a**) (25.8 mg, 0.3 mmol, 3.0 equiv) in DME (1 mL), and it was purified with columm chromatography on silica gel (AcOEt:Hex = 2:8) as white solid, 29 mg, 72% yield. MP: 189–191 °C. ^1^H NMR (500 MHz, CDCl_3_) δ 8.96 (d, *J* = 15.9 Hz, 1H), 8.02 (d, *J* = 8.2 Hz, 2H), 7.78 (d, *J* = 8.2 Hz, 2H), 7.04 – 7.01 (m, 2H), 6.76 (s, 1H), 6.25 (d, *J* = 15.9 Hz, 1H), 3.96 (s, 3H), 3.84 (s, 3H). ^13^C­{^1^H} NMR (126 MHz, CDCl_3_) δ 178.5, 166.8, 163.1, 160.0, 159.0, 144.5, 138.4, 134.7, 133.2, 132.9, 126.4, 126.0, 121.4, 115.5, 113.8, 109.8, 101.8, 56.0, 51.8. HRMS (ESI) *m*/*z* [M + H]^+^ calcd. for C_21_H_15_F_3_O_5_ 405.0872, found 405.0946.

#### Ethyl (E)-3-(7-methoxy-4-oxo-2-(4-(trifluoromethyl)­phenyl)-4H-chromen-5-yl)­acrylate (**9o**)

Following General Procedure, the compound was prepared from 7-methoxy-2-(4-(trifluoromethyl)­phenyl)-4H-chromen-4-one (**7c**) (32.0 mg, 0.1 mmol, 1 equiv) and ethyl acrylate (**8b**) (30.0 mg, 0.3 mmol, 3.0 equiv) in DME (1 mL), and it was purified with columm chromatography on silica gel (AcOEt:Hex = 3:7) as white solid, 28 mg, 67% yield. MP: 198–200 °C. ^1^H NMR (500 MHz, CDCl_3_) δ 8.96 (d, *J* = 15.9 Hz, 1H), 8.03 – 8.00 (m, 2H), 7.79 – 7.77 (m, 2H), 7.05 (d, *J* = 2.5 Hz, 1H), 7.01 (d, *J* = 2.5 Hz, 1H), 6.77 (s, 1H), 6.25 (d, *J* = 15.9 Hz, 1H), 4.30 (q, 2H), 3.96 (s, 3H), 1.37 (t, 3H). ^13^C­{^1^H} NMR (126 MHz, CDCl_3_) δ 178.6, 166.4, 163.1, 160.1, 159.0, 144.3, 138.6, 134.7, 133.0, 126.5, 126.0, 124.7, 122.0, 115.6, 113.8, 109.8, 101.8, 60.7, 56.0, 14.3. HRMS (ESI) *m*/*z* [M + H]^+^ calcd. for C_22_H_17_F_3_O_5_ 419.1028, found 419.1109.

#### *N*-Butyl (E)-3-(7-methoxy-4-oxo-2-(4-(trifluoromethyl)­phenyl)-4H-chromen-5-yl)­acrylate (**9p**)

Following General Procedure, the compound was prepared from 7-methoxy-2-(4-(trifluoromethyl)­phenyl)-4H-chromen-4-one (**7c**) (32.0 mg, 0.1 mmol, 1 equiv) and *n*-butyl acrylate (**8c**) (38.4 mg, 0.3 mmol, 3.0 equiv) in DME (1 mL), and it was purified with columm chromatography on silica gel (AcOEt:Hex = 2:8) as a brown solid, 34 mg, 76% yield. MP: 188–190 °C. ^1^H NMR (500 MHz, CDCl_3_) δ 8.96 (dd, *J* = 15.8 Hz, 1H), 8.03 – 7.99 (m, 2H), 7.79 – 7.76 (m, 2H), 7.04 (d, *J* = 2.5 Hz, 1H), 7.00 (d, *J* = 2.5 Hz, 1H), 6.76 (s, 1H), 6.25 (d, *J* = 15.8 Hz, 1H), 4.24 (t, 2H), 3.96 (s, 3H), 1.75 – 1.69 (m, 2H), 1.50 – 1.44 (m, 2H), 0.98 (t, 3H). ^13^C­{^1^H} NMR (126 MHz, CDCl_3_) δ 178.6, 166.5, 163.1, 160.0, 159.0, 144.2, 138.6, 134.8, 133.2, 132.9, 126.4, 126.00, 122.0, 115.5, 113.7, 109.8, 101.8, 64.6, 56.0, 30.8, 19.2, 13.8. HRMS (ESI) *m*/*z* [M + H]^+^ calcd. for C_24_H_21_F_3_O_5_ 447.1341, found 447.1421.

#### Methyl (E)-3-(7-methoxy-2-(4-methoxyphenyl)-4-oxo-4H-chromen-5-yl)­acrylate (**9q**)

Following General Procedure, the compound was prepared from 7-methoxy-2-(4-methoxyphenyl)-4H-chromen-4-one (**7d**) (28.2 mg, 0.1 mmol, 1 equiv) and methyl acrylate (**8a**) (25.8 mg, 0.3 mmol, 3.0 equiv) in DME (1 mL), and it was purified with columm chromatography on silica gel (AcOEt:Hex = 3:7) to yield a white solid, 11 mg, 40% yield. MP: 170–172 °C. ^1^H NMR (500 MHz, CDCl_3_) δ 9.01 (d, *J* = 16.1 Hz, 1H), 7.86 – 7.83 (m, 2H), 7.03 – 6.98 (m, 4H), 6.63 (s, 1H), 6.24 (d, *J* = 15.9 Hz, 1H), 3.94 (s, 3H), 3.89 (s, 3H), 3.83 (s, 3H). ^13^C­{^1^H} NMR (126 MHz, CDCl_3_) δ 178.9, 166.9, 162.7, 162.3, 161.9, 158.9, 145.0, 138.3, 127.8, 123.6, 121.1, 115.6, 114.4, 113.3, 107.2, 101.9, 55.9, 55.5, 51.8. HRMS (ESI) *m*/*z* [M + H]^+^ calcd for C_21_H_18_O_6_ 367.1103, found 367.1184.

#### Ethyl (E)-3-(7-methoxy-2-(4-methoxyphenyl)-4-oxo-4H-chromen-5-yl)­acrylate (**9r**)

Following General Procedure, the compound was prepared from 7-methoxy-2-(4-methoxyphenyl)-4H-chromen-4-one (**7d**) (28.2 mg, 0.1 mmol, 1 equiv) and ethyl acrylate (**8b**) (30.0 mg, 0.3 mmol, 3.0 equiv) in DME (1 mL) and it was purified with columm chromatography on silica gel (AcOEt:Hex = 2:8) as yellow solid, 21 mg, 55% yield. MP: 195–197 °C. ^1^H NMR (500 MHz, CDCl_3_) δ 9.01 (d, *J* = 15.9 Hz, 1H), 7.86 – 7.82 (m, 2H), 7.04 – 6.99 (m, 3H), 6.98 (d, *J* = 2.5 Hz, 1H), 6.63 (s, 1H), 6.23 (d, *J* = 15.9 Hz, 1H), 4.29 (q, 2H), 3.94 (s, 3H), 3.89 (s, 3H), 1.36 (t, 3H). ^13^C­{^1^H} NMR (126 MHz, CDCl_3_) δ 178.9, 166.5, 162.7, 162.3, 161.9, 158.9, 144.7, 138.4, 127.8, 123.6, 121.6, 115.6, 114.4, 113.2, 107.2, 101.9, 60.6, 55.9, 55.5, 14.3. HRMS (ESI) *m*/*z* [M + H]^+^ calcd. for C_22_H_20_O_6_ 380.1260, found 380.1339.

#### *N*-Butyl (E)-3-(7-methoxy-2-(4-methoxyphenyl)-4-oxo-4H-chromen-5-yl)­acrylate (**9s**)

Following General Procedure, the compound was prepared from 7-methoxy-2-(4-methoxyphenyl)-4H-chromen-4-one (**7d**) (28.2 mg, 0.1 mmol, 1 equiv) and *n*-butyl acrylate (**8c**) (38.4 mg, 0.3 mmol, 3.0 equiv) in DME (1 mL), and it was purified with columm chromatography on silica gel (AcOEt:Hex = 3:7) as yellow solid, 38 mg, 93% yield. MP: 139–141 °C. ^1^H NMR (500 MHz, CDCl_3_) δ 9.00 (d, *J* = 15.9, 1H), 7.83 (dd, *J* = 9.0, 2.2 Hz, 2H), 7.00 (m, 3H), 6.96 (d, J = 2.4 Hz, 1H), 6.62 (s, 1H), 6.23 (d, *J* = 15.9, 1H), 4.23 (t, 2H), 3.94 (s, 3H), 3.89 (s, 3H), 1.74 – 1.70 (m, 2H), 1.50 – 1.44 (m, 2H), 0.97 (t, 3H). ^13^C­{^1^H} NMR (126 MHz, CDCl_3_) δ 178.9, 166.6, 162.7, 162.3, 161.8, 158.9, 144.7, 138.4, 127.8, 123.6, 121.5, 115.5, 114.4, 113.2, 107.1, 101.8, 64.5, 55.9, 55.5, 30.8, 19.2, 13.8. HRMS (ESI) *m*/*z* [M + H]^+^ calcd. for C_24_H_24_O_6_ 409.1645, found 409.1649.

#### Methyl (E)-3-(2-(3,4-dimethoxyphenyl)-7-methoxy-4-oxo-4H-chromen-5-yl)­acrylate (**9t**)

Following General Procedure, the compound was prepared from 2-(3,4-dimethoxyphenyl)-7-methoxy-4H-chromen-4-one (**7e**) (31.2 mg, 0.1 mmol, 1 equiv) and methyl acrylate (**8a**) (25.8 mg, 0.3 mmol, 3.0 equiv) in DME (1 mL), and it was purified with columm chromatography on silica gel (AcOEt:Hex = 3:7) as yellow solid, 18 mg, 45% yield. MP: 190–192 °C. ^1^H NMR (500 MHz, CDCl_3_) δ 9.00 (d, *J* = 15.9 Hz, 1H), 7.52 (dd, *J* = 8.4, 2.1 Hz, 1H), 7.33 (d, *J* = 2.2 Hz, 1H), 7.00 (d, *J* = 2.2 Hz, 1H), 6.99 – 6.96 (m, 2H), 6.63 (s, 1H), 6.24 (d, *J* = 15.9 Hz, 1H), 3.99 (s, 3H), 3.97 (s, 3H), 3.95 (s, 3H), 3.84 (s, 3H). ^13^C­{^1^H} NMR (126 MHz, CDCl_3_) δ 178.8, 166.9, 162.7, 161.8, 158.9, 152.0, 149.3, 144.9, 138.2, 123.8, 121.1, 119.8, 115.5, 113.3, 111.1, 108.7, 107.5, 101.9, 56.14, 56.08, 55.9, 51.8. HRMS (ESI) *m*/*z* [M + H]^+^ calcd. for C_22_H_20_O_7_ 397.1209, found 397.1285.

#### Ethyl (E)-3-(2-(3,4-Dimethoxyphenyl)-7-Methoxy-4-Oxo-4H-Chromen-5-Yl)­acrylate (**9u**)

Following General Procedure, the compound was prepared from 2-(3,4-dimethoxyphenyl)-7-methoxy-4H-chromen-4-one (**7e**) (31.2 mg, 0.1 mmol, 1 equiv) and ethyl acrylate (**8b**) (30.0 mg, 0.3 mmol, 3.0 equiv) in DME (1 mL) and it was purified with columm chromatography on silica gel (AcOEt:Hex = 3:7) as yellow solid, 14 mg, 34% yield. MP: 195–197 °C. ^1^H NMR (500 MHz, CDCl_3_) δ 9.01 (d, *J* = 16.0 Hz, 1H), 7.53 (dd, *J* = 8.4, 2.2 Hz, 1H), 7.34 (d, *J* = 2.5 Hz, 1H), 7.02 (d, *J* = 2.5 Hz, 1H), 6.99 (d, *J* = 2.6 Hz, 1H), 6.97 (s, 1H), 6.64 (s, 1H), 6.24 (d, *J* = 16.0 Hz, 1H), 4.30 (q, 2H), 3.99 (s, 3H), 3.97 (s, 3H), 3.95 (s, 3H), 1.36 (t, 3H).). ^13^C­{^1^H} NMR (101 MHz, CDCl_3_) δ 178.9, 166.5, 162.7, 161.8, 158.9, 152.0, 149.3, 144.6, 138.4, 123.8, 121.6, 119.8, 115.6, 113.3, 111.1, 108.7, 107.5, 101.9, 60.6, 56.13, 56.08, 55.9, 14.3. HRMS (ESI) *m*/*z* [M + H]^+^ calcd. for C_23_H_22_O_7_ 411.1366, found 411.1449.

#### *N*-Butyl (E)-3-(2-(3,4-dimethoxyphenyl)-7-methoxy-4-oxo-4H-chromen-5-yl)­acrylate (**9v**)

Following General Procedure, the compound was prepared from 2-(3,4-dimethoxyphenyl)-7-methoxy-4H-chromen-4-one (**7e**) (31.2 mg, 0.1 mmol, 1 equiv) and *n*-butyl acrylate (**8c**) (38.4 mg, 0.3 mmol, 3.0 equiv) in DME (1 mL), and it was purified with columm chromatography on silica gel (AcOEt:Hex = 3:7) as yellow solid, 14 mg, 32% yield. MP: 145–147 °C. ^1^H NMR (500 MHz, CDCl_3_) δ 9.04 – 8.98 (m, 1H), 7.52 (dd, *J* = 8.5, 2.1 Hz, 1H), 7.34 (d, *J* = 2.2 Hz, 1H), 7.02 (dd, *J* = 2.5, 0.7 Hz, 1H), 6.99 – 6.97 (m, 2H), 6.64 (s, 1H), 6.25 (d, *J* = 15.9 Hz, 1H), 4.24 (t, *J* = 6.7 Hz, 2H), 3.99 (s, 3H), 3.97 (s, 3H), 3.95 (s, 3H), 1.75 – 1.69 (m, 2H), 1.50 – 1.43 (m, 2H), 0.98 (t, *J* = 7.4 Hz, 3H). ^13^C­{^1^H} NMR (101 MHz, CDCl_3_) δ 178.9, 166.6, 162.7, 161.8, 158.9, 152.0, 149.3, 144.6, 138.4, 123.8, 121.6, 119.8, 115.6, 113.3, 111.1, 108.7, 107.5, 101.9, 64.5, 56.12, 56.08, 55.9, 30.8, 19.2, 13.8. HRMS (ESI) calcd for C_25_H_26_O_7_ 439.1679, found 439.1752.

#### Methyl (E)-3-(2-(3,4-dimethoxyphenyl)-6-methoxy-4-oxo-4H-chromen-5-yl)­acrylate (**9w**)

Following General Procedure, the compound was prepared from 2-(3,4-dimethoxyphenyl)-6-methoxy-4H-chromen-4-one (**7f**) (31.2 mg, 0.1 mmol, 1 equiv) and methyl acrylate (**8a**) (25.8 mg, 0.3 mmol, 3.0 equiv) in 1,4-dioxane (1 mL), and it was purified with columm chromatography on silica gel (AcOEt:Hex = 5:5) as yellow solid, 23 mg, 58% yield. MP: 203–205 °C. ^1^H NMR (500 MHz, CDCl_3_) δ 8.83 (d, *J* = 16.3 Hz, 1H), 7.59 – 7.50 (m, 2H), 7.38 – 7.31 (m, 2H), 6.97 (d, *J* = 8.5 Hz, 1H), 6.66 (s, 1H), 6.59 (d, *J* = 16.3 Hz, 1H), 3.99 (s, 3H), 3.96 (s, 3H), 3.92 (s, 3H), 3.84 (s, 3H). ^13^C­{^1^H} NMR (126 MHz, CDCl_3_) δ 178.7, 167.0, 160.7, 154.0, 151.0, 150.6, 148.3, 138.4, 122.9, 122.8, 121.50, 121.46, 118.9, 118.6, 116.5, 110.1, 107.7, 106.2, 55.5, 55.12, 55.07, 50.7. HRMS (ESI) *m*/*z* [M + H]^+^ calcd. for C_22_H_20_O_7_ 397.1281, found 397.1285.

#### Methyl (E)-3-(7-bromo-4-oxo-2-phenyl-4H-chromen-5-yl)­acrylate (**9x**)

Following General Procedure, the compound was prepared from 7-bromo-2-phenyl-4H-chromen-4-one (**7g**) (30.1 mg, 0.1 mmol, 1 equiv) and methyl acrylate (**8a**) (51.6 mg, 0.6 mmol, 6.0 equiv) in 1,4-dioxane (1 mL), and it was purified with columm chromatography on silica gel (AcOEt:Hex = 2:8) as white solid, 29 mg, 75% yield. MP: 220–222 °C. ^1^H NMR (500 MHz, CDCl_3_) δ 8.93 (d, *J* = 15.9 Hz, 1H), 7.89 (d, *J* = 7.3 Hz, 2H), 7.79 (s, 1H), 7.62 – 7.46 (m, 4H), 6.77 (s, 1H), 6.27 (d, *J* = 15.9 Hz, 1H), 3.84 (s, 3H). ^13^C­{^1^H} NMR (126 MHz, CDCl_3_) δ 178.8, 166.5, 162.2, 157.2, 143.4, 138.4, 131.9, 130.8, 129.1, 127.8, 127.0, 126.2, 122.4, 122.2, 120.3, 108.9, 51.9. HRMS (ESI) *m*/*z* [M + H]^+^ calcd. for C_19_H_13_BrO_4_ 385.0069, found 385.0071.

#### Methyl (E)-3-(7-((diethylcarbamoyl)­oxy)-4-oxo-2-phenyl-4H-chromen-5-yl)­acrylate (**11a**)

Following General Procedure, the compound was prepared from 4-oxo-2-phenyl-4H-chromen-7-yl diethylcarbamate (**10a**) (33.7 mg, 0.1 mmol, 1 equiv) and methyl acrylate (**8a**) (25.8 mg, 0.3 mmol, 3.0 equiv) in DME (1 mL), and it was purified with columm chromatography on silica gel (AcOEt:Hex = 2:8) as white solid, 35 mg, 83% yield. MP: 153–155 °C. ^1^H NMR (500 MHz, CDCl_3_) δ 9.02 (d, *J* = 16.0, 1H), 7.91 – 7.88 (m, 2H), 7.54 – 7.49 (m, 4H), 7.24 (dd, *J* = 2.3, 0.7 Hz, 1H), 6.76 (s, 1H), 6.29 (d, *J* = 15.9 Hz, 1H), 3.83 (s, 3H), 3.47 (q, 2H), 3.44 – 3.39 (q, 2H), 1.30 (t, 3H), 1.24 (t, 3H). ^13^C­{^1^H} NMR (126 MHz, CDCl_3_) δ 179.0, 166.8, 162.3, 157.8, 154.6, 152.8, 144.2, 138.1, 131.7, 131.1, 129.1, 126.2, 121.7, 118.7, 118.6, 112.1, 108.7, 51.8, 42.6, 42.2, 14.3, 13.3. HRMS (ESI) *m*/*z* [M + H]^+^ calcd. for C_24_H_23_NO_6_ 422.1598, found 422.1599.

#### Ethyl (E)-3-(7-((diethylcarbamoyl)­oxy)-4-oxo-2-phenyl-4H-chromen-5-yl)­acrylate (**11b**)

Following General Procedure, the compound was prepared from 4-oxo-2-phenyl-4H-chromen-7-yl diethylcarbamate (**10a**) (33.7 mg, 0.1 mmol, 1 equiv) and ethyl acrylate (**8b**) (30.0 mg, 0.3 mmol, 3.0 equiv) in DME (1 mL), and it was purified with columm chromatography on silica gel (AcOEt:Hex = 2:8) as white solid, 33 mg, 75% yield. MP: 116–118 °C. ^1^H NMR (400 MHz, CDCl_3_) δ 9.01 (d, *J* = 15.9 Hz, 1H), 7.92 – 7.86 (m, 2H), 7.53 (m, 4H), 7.25 (d, *J* = 2.3 Hz, 1H), 6.77 (s, 1H), 6.28 (d, *J* = 15.9 Hz, 1H), 4.29 (q, 2H), 3.48 (q, 2H), 3.43 (q, 2H), 1.36 (t, 3H), 1.30 (t, 3H), 1.25 (t, 3H). ^13^C­{^1^H} NMR (126 MHz, CDCl_3_) δ 179.0, 166.4, 162.3, 157.9, 154.6, 152.8, 143.9, 138.2, 131.7, 131.2, 129.1, 126.2, 122.2, 118.6, 118.5, 112.0, 108.7, 60.6, 42.6, 42.2, 14.3, 13.3. HRMS (ESI) *m*/*z* [M + H]^+^ calcd. for C_25_H_25_NO_6_ 436.1682, found 436.1762.

#### *N*-Butyl (E)-3-(7-((diethylcarbamoyl)­oxy)-4-oxo-2-phenyl-4H-chromen-5-yl)­acrylate (**11c**)

Following General Procedure, the compound was prepared from 4-oxo-2-phenyl-4H-chromen-7-yl diethylcarbamate (**10a**) (33.7 mg, 0.1 mmol, 1 equiv) and *n*-butyl acrylate (**8c**) (38.4 mg, 0.3 mmol, 3.0 equiv) in DME (1 mL), it was purified with columm chromatography on silica gel (AcOEt:Hex = 2:8) as white solid, 43 mg, 92% yield. MP: 109–111 °C. ^1^H NMR (500 MHz, CDCl_3_) δ 9.02 (d, *J* = 15.9 Hz, 1H), 7.92 – 7.89 (m, 2H), 7.53 (m, 4H), 7.26 (d, *J* = 2.3 Hz, 1H), 6.78 (s, 1H), 6.29 (d, *J* = 15.9 Hz, 1H), 4.24 (t, 2H), 3.49 (q, 2H), 3.44 (q, 2H), 1.72 (m, 2H), 1.47 (m, 2H), 1.30 (t, 3H), 1.26 (t, 3H), 0.98 (t, 3H). ^13^C­{^1^H} NMR (126 MHz, CDCl_3_) δ 179.0, 166.5, 162.3, 157.9, 154.6, 152.8, 143.9, 138.3, 131.7, 131.2, 129.1, 126.2, 122.2, 118.7, 118.6, 112.0, 108.7, 64.5, 42.6, 42.2, 30.8, 19.2, 14.3, 13.8, 13.3. HRMS (ESI) *m*/*z* [M + H]^+^ calcd. for C_27_H_29_NO_6_ 464.1995, found 464.2073.

#### (E)-4-oxo-2-phenyl-5-styryl-4H-chromen-7-yl Diethylcarbamate (**11e**)

Following General Procedure, the compound was prepared from 4-oxo-2-phenyl-4H-chromen-7-yl diethylcarbamate (**10a**) (33.7 mg, 0.1 mmol, 1 equiv) and styrene (**8e**) (31.2 mg, 0.3 mmol, 3.0 equiv) in DME (1 mL) and it was purified with columm chromatography on silica gel (AcOEt:Hex = 2:8) as yellow solid, 27 mg, 61% yield. MP: 150–152 °C. ^1^H NMR (400 MHz, CDCl_3_) δ 8.73 (d, *J* = 16.2 Hz, 1H), 7.92 – 7.89 (m, 2H), 7.64 – 7.61 (m, 2H), 7.55 – 7.49 (m, 4H), 7.38 – 7.35 (m, 2H), 7.29 (m, 1H), 7.26 (s, 1H), 7.04 (d, *J* = 16.2 Hz, 1H), 6.75 (s, 1H), 3.50 (q, 2H), 3.45 (q, 2H), 1.31 (t, 3H), 1.26 (t, 3H). ^13^C­{^1^H} NMR (101 MHz, CDCl_3_) δ 179.8, 161.7, 158.2, 154.5, 153.1, 141.4, 137.3, 132.5, 131.5, 131.3, 129.0, 128.6, 128.0, 127.8, 127.2, 126.1, 118.1, 116.9, 110.0, 108.8, 42.5, 42.1, 14.3, 13.3. HRMS (ESI) *m*/*z* [M + H]^+^ calcd. for C_28_H_25_NO_4_ 440.1784, found 440.1861.

#### (E)-4-oxo-5-(3-oxobut-1-en-1-yl)-2-phenyl-4H-chromen-7-yl Diethylcarbamate (**11f**)

Following General Procedure, the compound was prepared from 4-oxo-2-phenyl-4H-chromen-7-yl diethylcarbamate (**10a**) (33.7 mg, 0.1 mmol, 1 equiv) and methyl vinyl ketone (**8f**) (21.0 mg, 0.3 mmol, 3.0 equiv) in DME (1 mL). It was purified with columm chromatography on silica gel (AcOEt:Hex = 2:8) as brown solid, 27.5 mg, 68% yield. MP: 155–157 °C. ^1^H NMR (500 MHz, CDCl_3_) δ 9.01 (d, *J* = 16.4 Hz, 1H), 7.92 – 7.90 (m, 2H), 7.54 (m, 4H), 7.29 – 7.28 (m, 1H), 6.78 (s, 1H), 6.49 (d, *J* = 16.4 Hz, 1H), 3.49 (q, 2H), 3.44 (q, 2H), 2.50 (s, 3H), 1.31 (t, 3H), 1.25 (t, 3H). ^13^C­{^1^H} NMR (126 MHz, CDCl_3_) δ 199.5, 179.3, 162.50 158.0, 154.7, 152.8, 143.5, 138.2, 131.8, 131.4, 131.0, 129.1, 126.2, 118.5, 118.4, 112.3, 108.6, 42.6, 42.2, 26.4, 14.3, 13.3. HRMS (ESI) *m*/*z* [M + H]^+^ calcd. for C_24_H_23_NO_5_ 406.1576, found 406.1653.

#### (E)-4-oxo-2-phenyl-5-(2-(phenylsulfonyl)­vinyl)-4H-chromen-7-yl Diethylcarbamate (**11g**)

Following General Procedure, the compound was prepared from 4-oxo-2-phenyl-4H-chromen-7-yl diethylcarbamate (**10a**) (33.7 mg, 0.1 mmol, 1 equiv) and phenyl vinyl sulfone (**8g**) (50.4 mg, 0.3 mmol, 3.0 equiv) in DME (1 mL), and it was purified with columm chromatography on silica gel (AcOEt:Hex = 2:8) as white solid, 17 mg, 33% yield. MP: 190–192 °C. ^1^H NMR (500 MHz, CDCl_3_) δ 8.91 (d, *J* = 15.3 Hz, 1H), 8.03 – 8.00 (m, 2H), 7.83 – 7.78 (m, 2H), 7.54 – 7.45 (m, 7H), 7.10 (d, *J* = 2.2 Hz, 1H), 6.69 (s, 1H), 6.64 (d, *J* = 15.3 Hz, 1H), 3.39 (q, 2H), 3.34 (q, 2H), 1.22 (t, 3H), 1.17 (t, 3H). ^13^C­{^1^H} NMR (126 MHz, CDCl_3_) δ 178.6, 162.6, 157.7, 154.6, 152.6, 143.2, 140.4, 135.8, 133.4, 131.8, 131.0, 130.6, 129.3, 129.1, 128.1, 126.2, 119.0, 118.8, 112.7, 108.5, 42.6, 42.2, 14.3, 13.3. HRMS (ESI) *m*/*z* [M + H]^+^ calcd. for C_28_H_25_NO_6_S 504.1403, found 504.1484.

#### Methyl (E)-3-(7-((diethylcarbamoyl)­oxy)-2-(4-fluorophenyl)-4-oxo-4H-chromen-5-yl)­acrylate (**11h**)

Following General Procedure, the compound was prepared from 2-(4-fluorophenyl)-4-oxo-4H-chromen-7-yl diethylcarbamate (**10b**) (35.5 mg, 0.1 mmol, 1 equiv) and methyl acrylate (**8a**) (25.8 mg, 0.3 mmol, 3.0 equiv) in DME (1 mL). It was purified with columm chromatography on silica gel (AcOEt:Hex = 2:8) as white solid, 26 mg, 60% yield. MP: 160–162 °C. ^1^H NMR (500 MHz, CDCl_3_) δ 9.00 (d, *J* = 15.9 Hz, 1H), 7.92 – 7.89 (m, 2H), 7.52 (d, *J* = 2.3 Hz, 1H), 7.27 – 7.23 (d, *J* = 2.3 Hz, 1H), 7.23 – 7.19 (m, 2H), 6.71 (s, 1H), 6.29 (d, *J* = 15.9 Hz, 1H), 3.84 (s, 3H), 3.49 (q, 2H), 3.43 (q, 2H), 1.30 (t, 3H), 1.26 (t, 3H). ^13^C­{^1^H} NMR (126 MHz, CDCl_3_) δ 178.8, 166.8, 161.3, 157.7, 154.6, 152.7, 144.1, 138.1, 128.5, 128.4, 121.8, 118.6, 118.5, 116.4, 116.2, 112.0, 108.4, 51.8, 42.6, 42.2, 14.3, 13.3. HRMS (ESI) *m*/*z* [M + H]^+^ calcd. For C_24_H_22_FNO_6_ 440.1431, found 440.1508.

#### Ethyl (E)-3-(7-((diethylcarbamoyl)­oxy)-2-(4-fluorophenyl)-4-oxo-4H-chromen-5-yl)­acrylate (**11i**)

Following General Procedure, the compound was prepared from 2-(4-fluorophenyl)-4-oxo-4H-chromen-7-yl diethylcarbamate (**10b**) (35.5 mg, 0.1 mmol, 1 equiv) and ethyl acrylate (**8b**) (30.0 mg, 0.3 mmol, 3.0 equiv) in DME (1 mL), and it was purified with columm chromatography on silica gel (AcOEt:Hex = 2:8) as white solid, 20 mg, 44% yield. MP: 149–151 °C. ^1^H NMR (400 MHz, CDCl_3_) δ 8.99 (d, *J* = 15.9 Hz, 1H), 7.93 – 7.87 (m, 2H), 7.51 (d, *J* = 2.2 Hz, 1H), 7.24 (d, *J* = 2.2 Hz, 1H), 7.20 (m, 2H), 6.71 (s, 1H), 6.28 (d, *J* = 15.9 Hz, 1H), 4.29 (q, 2H), 3.53 – 3.46 (q, 2H), 3.43 (q, 2H), 1.36 (t, 3H), 1.30 (t, 3H), 1.24 (t, 3H). ^13^C­{^1^H} NMR (101 MHz, CDCl_3_) δ 178.8, 166.3, 166.1, 163.5, 161.3, 157.7, 154.6, 152.7, 143.8, 138.2, 128.44, 128.35, 127.3, 122.3, 118.6, 118.5, 116.4, 116.2, 111.9, 108.4, 60.6, 42.6, 42.1, 14.3, 13.3. HRMS (ESI) *m*/*z* [M + H]^+^ calcd. for C_25_H_24_FNO_6_ 454.1588, found 454.1663.

#### Methyl (E)-3-(7-((diethylcarbamoyl)­oxy)-4-oxo-2-(4-(trifluoromethyl)­phenyl)-4H-chromen-5-yl)­acrylate (**11j**)

Following General Procedure, the compound was prepared from 4-oxo-2-(4-(trifluoromethyl)­phenyl)-4H-chromen-7-yl diethylcarbamate (**10c**) (40.5 mg, 0.1 mmol, 1 equiv) and methyl acrylate (**8a**) (25.8 mg, 0.3 mmol, 3.0 equiv) in DME (1 mL), and it was purified with columm chromatography on silica gel (AcOEt:Hex = 3:7) as white solid, 16 mg, 32% yield. MP: 197–199 °C. ^1^H NMR (500 MHz, CDCl_3_) δ 8.98 (d, *J* = 15.9 Hz, 1H), 8.02 (d, *J* = 8.2 Hz, 2H), 7.79 (d, *J* = 8.2 Hz, 2H), 7.55 (d, *J* = 2.2 Hz, 1H), 7.26 (d, *J* = 2.6 Hz, 1H), 6.81 (s, 1H), 6.28 (d, *J* = 15.9 Hz, 1H), 3.84 (s, 3H), 3.48 (q, 2H), 3.43 (q, 2H), 1.30 (t, 3H), 1.26 (t, 3H). ^13^C­{^1^H} NMR (126 MHz, CDCl_3_) δ 178.7, 166.7, 160.5, 157.8, 154.8, 152.7, 143.9, 138.3, 134.6, 126.6, 126.1, 124.7, 122.5, 122.0, 118.8, 118.6, 112.1, 109.9, 51.9, 42.6, 42.2, 14.3, 13.3. HRMS (ESI) *m*/*z* [M + H]^+^ calcd for C_25_H_22_F_3_NO_6_ 490.1399, found 490.1476.

#### Ethyl (E)-3-(7-((diethylcarbamoyl)­oxy)-4-oxo-2-(4-(trifluoromethyl)­phenyl)-4H-chromen-5-yl)­acrylate (**11k**)

Following General Procedure, the compound was prepared from 4-oxo-2-(4-(trifluoromethyl)­phenyl)-4H-chromen-7-yl diethylcarbamate (**10c**) (40.5 mg, 0.1 mmol, 1 equiv) and ethyl acrylate (**8b**) (30.0 mg, 0.3 mmol, 3.0 equiv) in DME (1 mL). It was purified with columm chromatography on silica gel (AcOEt:Hex = 2:8) as white solid, 28 mg, 55% yield. MP: 190–192 °C. ^1^H NMR (500 MHz, CDCl_3_) δ 8.98 (d, *J* = 15.9 Hz, 1H), 8.02 (d, *J* = 8.2 Hz, 2H), 7.79 (d, *J* = 8.2 Hz, 2H), 7.55 (d, *J* = 2.2 Hz, 1H), 7.27 (d, *J* = 2.2 Hz, 1H), 6.82 (s, 1H), 6.30 (d, *J* = 15.9 Hz, 1H), 4.30 (q, 2H), 3.49 (q, 2H), 3.44 (q, 2H), 1.36 (t, 3H), 1.31 (t, 3H), 1.26 (t, 3H). ^13^C­{^1^H} NMR (126 MHz, CDCl_3_) δ 178.7, 166.3, 160.5, 157.8, 154.8, 152.7, 143.7, 138.3, 134.6, 133.4, 126.5, 126.11, 126.08, 126.05, 122.5, 118.8, 118.6, 112.0, 109.8, 60.7, 42.6, 42.2, 14.3, 13.3. HRMS (ESI) *m*/*z* [M + H]^+^ calcd for C_26_H_24_F_3_NO_6_ 504.1556, found 504.1633.

#### Methyl (E)-3-(7-((diethylcarbamoyl)­oxy)-2-(4-methoxyphenyl)-4-oxo-4H-chromen-5-yl)­acrylate (**11l**)

Following General Procedure, the compound was prepared from 2-(4-methoxyphenyl)-4-oxo-4H-chromen-7-yl diethylcarbamate (**10d**) (36.7 mg, 0.1 mmol, 1 equiv) and methyl acrylate (**8a**) (25.8 mg, 0.3 mmol, 3.0 equiv) in DME (1 mL). It was purified with columm chromatography on silica gel (AcOEt:Hex = 2:8) as white solid, 21.5 mg, 47% yield. MP: 158–160 °C. ^1^H NMR (400 MHz, CDCl_3_) δ 9.03 (d, *J* = 16.2 Hz, 1H), 7.86 – 7.82 (m, 2H), 7.50 (d, *J* = 2.2 Hz, 1H), 7.22 (d, *J* = 2.3 Hz, 1H), 7.03 – 6.99 (m, 2H), 6.67 (s, 1H), 6.28 (d, *J* = 16.2 Hz, 1H), 3.89 (s, 3H), 3.83 (s, 3H), 3.49 (q, 2H), 3.42 (q, 2H), 1.30 (t, 3H), 1.25 (t, 3H). ^13^C­{^1^H} NMR (101 MHz, CDCl_3_) δ 178.9, 166.8, 162.5, 162.3, 157.7, 154.4, 152.8, 144.35, 144.33, 138.0, 129.5, 127.9, 123.3, 121.5, 118.6, 118.4, 114.5, 112.0, 107.2, 55.5, 51.8, 42.5, 42.1, 14.3, 13.3. HRMS (ESI) *m*/*z* [M + H]^+^ calcd for C_25_H_25_NO_7_ 452.1631, found 452.1712.

#### Ethyl (E)-3-(7-((diethylcarbamoyl)­oxy)-2-(4-methoxyphenyl)-4-oxo-4H-chromen-5-yl)­acrylate (**11m**)

Following General Procedure, the compound was prepared from 2-(4-methoxyphenyl)-4-oxo-4H-chromen-7-yl diethylcarbamate (**10d**) (36.7 mg, 0.1 mmol, 1 equiv) and ethyl acrylate (**8b**) (30.0 mg, 0.3 mmol, 3.0 equiv) in DME (1 mL), and it was purified with columm chromatography on silica gel (AcOEt:Hex = 2:8) as yellow solid, 27 mg, 59% yield. MP: 152–154 °C. ^1^H NMR (500 MHz, CDCl_3_) δ 9.02 (d, *J* = 15.9 Hz, 1H), 7.86 – 7.82 (m, 2H), 7.49 (d, *J* = 2.3 Hz, 1H), 7.23 (d, *J* = 2.3 Hz, 1H), 7.03 – 6.99 (m, 2H), 6.67 (s, 1H), 6.27 (d, *J* = 15.9 Hz, 1H), 4.29 (q, 2H), 3.89 (s, 3H), 3.48 (q, 2H), 3.43 (q, 2H), 1.36 (t, 3H), 1.30 (t, 3H), 1.25 (t, 3H). ^13^C­{^1^H} NMR (126 MHz, CDCl_3_) δ 178.9, 166.4, 162.5, 162.3, 157.7, 154.4, 152.8, 144.1, 138.1, 129.5, 127.9, 123.3, 122.0, 118.6, 118.4, 114.5, 112.0, 107.2, 60.6, 55.5, 42.6, 42.1, 14.3, 13.3. HRMS (ESI) *m*/*z* [M + H]^+^ calcd for C_26_H_27_NO_7_ 466.1788, found 466.1868.

#### Methyl (E)-3-(7-((diethylcarbamoyl)­oxy)-2-(3,4-dimethoxyphenyl)-4-oxo-4H-chromen-5-yl)­acrylate (**11n**)

Following General Procedure, the compound was prepared from 2-(3,4-dimethoxyphenyl)-4-oxo-4H-chromen-7-yl diethylcarbamate (**10e**) (39.7 mg, 0.1 mmol, 1 equiv) and methyl acrylate (**8a**) (25.8 mg, 0.3 mmol, 3.0 equiv) in DME (1 mL). It was purified with columm chromatography on silica gel (AcOEt:Hex = 4:6) as yellow solid, 33 mg, 68% yield. MP: 179–181 °C. ^1^H NMR (400 MHz, CDCl_3_) δ 9.03 (d, *J* = 15.9 Hz, 1H), 7.52 (dd, *J* = 8.3, 2.2 Hz, 2H), 7.35 (d, *J* = 2.2 Hz, 1H), 7.22 (d, *J* = 2.3 Hz, 1H), 6.97 (d, *J* = 8.5 Hz, 1H), 6.68 (s, 1H), 6.28 (d, *J* = 15.9 Hz, 1H), 3.98 (s, 3H), 3.96 (s, 3H), 3.83 (s, 3H), 3.51 – 3.46 (q, 2H), 3.46 – 3.41 (q, 2H), 1.31 (t, 3H), 1.26 (t, 3H). ^13^C­{^1^H} NMR (101 MHz, CDCl_3_) δ 177.8, 165.8, 161.2, 156.7, 153.4, 151.8, 151.1, 148.3, 143.3, 137.0, 122.5, 120.6, 118.9, 117.6, 117.4, 111.1, 110.1, 107.6, 106.5, 55.12, 55.07, 50.8, 41.5, 41.1, 13.23, 12.3. HRMS (ESI) *m*/*z* [M + H]^+^ calcd for C_26_H_27_NO_8_ 482.1737, found 482.1818.

#### Ethyl (E)-3-(7-(diethylcarbamoyl)­oxy)-2-(3,4-dimethoxyphenyl)-4-oxo-4H-chromen-5-yl)­acrylate (**11o**)

Following General Procedure, the compound was prepared from 2-(3,4-dimethoxyphenyl)-4-oxo-4H-chromen-7-yl diethylcarbamate (**10e**) (39.7 mg, 0.1 mmol, 1 equiv) and ethyl acrylate (**8b**) (30.0 mg, 0.3 mmol, 3.0 equiv) in DME (1 mL). It was purified with columm chromatography on silica gel (AcOEt:Hex = 4:6) as yellow solid, 37 mg, 75% yield. MP: 157–159 °C. ^1^H NMR (500 MHz, CDCl_3_) δ 9.02 (d, *J* = 15.9 Hz, 1H), 7.54 – 7.48 (m, 2H), 7.34 (d, *J* = 2.3 Hz, 1H), 7.23 (d, *J* = 2.3 Hz, 1H), 6.97 (d, *J* = 8.5 Hz, 1H), 6.68 (s, 1H), 6.28 (d, *J* = 15.9 Hz, 1H), 4.29 (q, 2H), 3.98 (s, 3H), 3.96 (s, 3H), 3.48 (q, 2H), 3.43 (q, 2H), 1.36 (t, 3H), 1.30 (t, 3H), 1.26 (t, 3H). ^13^C­{^1^H} NMR (101 MHz, CDCl_3_) δ 177.8, 165.4, 161.2, 156.7, 153.4, 151.8, 151.1, 148.3, 143.00, 142.99, 137.1, 122.5, 121.0, 118.9, 117.6, 117.4, 111.0, 110.1, 107.6, 106.4, 59.6, 55.09, 55.06, 41.5, 41.1, 13.3, 12.3. HRMS (ESI) *m*/*z* [M + H]^+^ calcd for C_27_H_29_NO_8_ 496.1893, found 496.1979.

### Procedure for the 1 Mmol Scale Synthesis of Compound 11a

In a reaction vial, flavone **10a** (337 mg, 1.0 mmol), [RuCl_2_(*p*-cymene)]_2_ (24.4 mg, 0.04 mmol), Cu­(OAc)_2_·H_2_O (439.2 mg, 2.2 mmol) were added. The AgSbF_6_ (68.7 mg, 0.20 mmol) was added inside a nitrogen-filled glovebag. The methyl acrylate **8a** (516.5 mg, 6.0 mmol) and 1,2 dimethoxyethane (3 mL) were then added, and the vial was sealed. The reaction mixture was stirred at 100 °C in an oil bath, under nitrogen atmosphere for 18 h. After cooling to room temperature, the reaction mixture was diluted with ethyl acetate and filtered through a short pad of Celite. The filtrate was washed with saturated aqueous ammonium chloride, dried over anhydrous sodium sulfate, and concentrated under reduced pressure. The crude product was purified by silica gel column chromatography using (AcOEt:Hex 3:7) to afford the desired product **11a** (347 mg, 82%).

### Computational Methods

All structures were optimized using density functional theory (DFT) as implemented in Gaussian 16,^33^ with B3LYP^34^ as functional, 6–31G­(d,p) as the basis set for nonmetallic atoms, and LANL2DZ^35^ as the basis set for ruthenium. Final energies were obtained by performing single-point calculations on the previously optimized structures at M06 ^36^/6–311++G­(d,p) level of theory for nonmetallic atoms and SDD basis set for ruthenium,^37^ introducing solvation factors with the IEF-PCM^38^ method, and 1,4-dioxane as the solvent. The stationary points were characterized by frequency calculations in order to verify that they had the correct number of imaginary frequencies.

## Supplementary Material



## Data Availability

The data underlying this study are available in the published article and its Supporting Information.

## Notes

^1^H NMR and ^13^C NMR spectra data of all compounds, computacional details (PDF)
