# Supplementary material for: Ruthenium-Catalyzed C–H Alkenylation of Flavones with Alkenes: Chemoselective Synthesis and Mechanistic Insights from DFT Studies
Source: J Org Chem. 2026 Jun 23;91(26):8769–83. doi: 10.1021/acs.joc.6c00235 (PMC13339625; doi:10.1021/acs.joc.6c00235)

Supporting Information

Ruthenium-Catalyzed C–H Alkenylation of Flavones with Alkenes: Chemoselective Synthesis and Mechanistic Insights from DFT Studies

*Nathalia S. de Oliveira [a], Luana G. de Souza [a,b], Maria Eduarda C. L. de Oliveira [a], Marina A. Alves [c], Asier Carral-Menoyo [d], Nuria Sotomayor [d], Alcides J. M. da Silva [a]**

[a] Laboratório de Catálise Orgânica, Instituto de Pesquisas de Produtos Naturais, Universidade Federal do Rio de Janeiro, Ilha do Fundão, CCS, Bloco H – Sala H1-29, Rio de Janeiro, RJ 21941-599, Brazil

[b] Departamento de Química Analítica, Instituto de Química, Centro de Tecnologia e Ciências, Universidade do Estado do Rio de Janeiro, Rua São Francisco Xavier 524, Pav. Haroldo Lisboa da Cunha – Maracanã, Rio de Janeiro, RJ 20550-900, Brazil

[c] Laboratório de Metabolômica Aplicada à Medicina de Sistemas (Meta2MS), Instituto de Pesquisa de Produtos Naturais, Universidade Federal do Rio de Janeiro, Rio de Janeiro, RJ 21941-598, Brazil

[d] Departamento de Química Orgánica, Facultad de Ciencia y Tecnología, Universidad del País Vasco, Euskal Herriko Unibertsitatea UPV/EHU Apdo. 644, 48080 Bilbao, Spain

* Corresponding author, **E-mail:** alcides@ippn.ufrj.br

Table of Contents

1. GENERAL INFORMATION S3
2. GENERAL PROCEDURES AND CHARACTERIZATION DATA S3
   2.1 Synthesis of 7-methoxy-2-phenyl-4*H*-chromen-4-one derivatives (7a-f) S4
   2.2 Synthesis of 7-bromo-2-phenyl-4H-chromen-4-one (7g) S7
   2.3 Synthesis of 7-hydroxy-2-phenyl-4*H*-chromen-4-one derivatives S7
   2.4 Synthesis of 4-oxo-2-phenyl-4H-chromen-7-yl diethylcarbamate derivatives (10a-c) S9
   2.5 Synthesis of 4-acetyl-3-hydroxyphenyl diethylcarbamate S11
   2.6 Synthesis of 4-oxo-2-phenyl-4H-chromen-7-yl diethylcarbamate derivatives (10d-e) S11
   2.7 General Procedure for the Ru(II)-Catalyzed C-H Alkenylation of Flavones S13
3. COMPUTACIONAL METHODS S32
4. DEUTERIUM LABELLING STUDIES S54
5. REFERENCES S55
6. COPIES OF NMR SPECTRA S57

1. General Information

All reagents and solvents were purchased from Sigma-Aldrich/Merck and TCI, and were used without further purification unless otherwise noted. The progress of reactions was monitored by TLC and flash column chromatography was carried on silica gel 60 (SiliCycle®, 230 - 400 mesh) was employed. Analytical thin layer chromatography (TLC) was performed using precoated TLC sheets of silica gel 60 F254 (SiliCycle®), and the spots were determined under UV light (254 nm and 365 nm). Known compounds were synthesized according to previously reported procedures. The alkenes were obtained from commercial sources and used without further purification. NMR spectra were recorded on Varian NMR spectrometers (400 or 500 MHz for ^{1}H; 101 or 126 MHz for ^{13}C) using $CDCl_3$ or CD_3OD as solvent and TMS as an internal standard at room temperature. Chemical shifts (δ) are given in ppm and calibrated using the signal of tetramethylsilane (TMS) as internal reference (δH and δC = 0 ppm). Data are reported as follows: Chemical shift (multiplicity, coupling constant(s), integration). Coupling constants were quoted to the nearest 0.1 Hz and multiplicity reported according to standard abbreviations. Structural assignments were made with additional information from gCOSY, gHSQC, and gHMBC experiments. High-resolution mass spectrometric analyses were performed on a Q Exactive™ hybrid quadrupole–Orbitrap mass spectrometer (Thermo Fisher Scientific, Bremen, Germany) equipped with an electrospray ionization (ESI) source operating in positive ion mode. Samples were prepared by adding 1 mL of acetonitrile to the microtube. The samples were analyzed by direct infusion at a flow rate of 10 µL/min. Spectra were acquired over the *m/z* range 100–600 at a resolving power of 70,000 (FWHM at *m/z* = 200). The high-resolution data enabled accurate mass measurement and molecular formula confirmation for the synthesized prototypes. The melting points were obtained on a Fisatom 430 and are uncorrected.

2. General procedures and characterization data

2.1 **Synthesis of 7-methoxy-2-phenyl-4*H*-chromen-4-one derivatives (7a-f)**

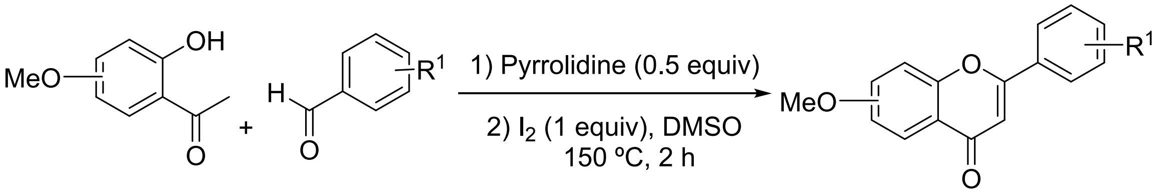

2′-Hydroxy-4′-methoxyacetophenone (0.166 g, 1.0 mmol) and benzaldehyde (0.117 g, 1.1 mmol) were mixed with pyrrolidine (0.036 g, 0.5 mmol) in DMSO (5 mL). The reaction mixture was heated in an oil bath at 150 °C for 1 h, according reported by da Silva et al.[1] After this period, iodine (0.254 g, 1.0 mmol) was added, and the mixture was maintained at the same temperature for an additional hour. Reaction progress was monitored by TLC. Upon completion, the mixture was cooled to room temperature and quenched with saturated sodium thiosulfate solution (20 mL). The resulting suspension was extracted with ethyl acetate (3 x 50 mL). The combined organic layers were washed with brine, dried over anhydrous sodium sulfate, and concentrated under reduced pressure. The crude product was purified by flash column chromatography on silica gel using a gradient of hexanes and ethyl acetate as the eluent. The structural of the isolated product was confirmed by comparison with literature data.

7-methoxy-2-phenyl-4H-chromen-4-one (7a) [1a]

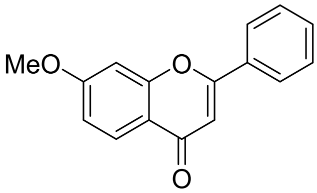

Following General Procedure 2.1, the compound was prepared from 2′-Hydroxy-4′-methoxyacetophenone (0.166 g, 1.0 mmol) and benzaldehyde (0.116 g, 1.1 mmol, 1.1 equiv) in DMSO (5 mL) and it was purified with columm chromatography on silica gel (AcOEt:Hex = 2:8) as white solid, 225 mg, 89% yield. MP: 81-83 ºC. ^{1}H NMR (400 MHz, $CDCl_3$) δ 8.14 (d, J = 8.6 Hz, 1H), 7.93 – 7.90 (m, 2H), 7.52 (dd, J = 5.2, 2.0 Hz, 3H), 7.01 – 6.96 (m, 2H), 6.77 (s, 1H), 3.94 (s, 3H). ^{13}C{^{1}H} NMR (101 MHz, $CDCl_3$) δ 177.9, 164.2, 163.0, 158.0, 131.9, 131.4, 129.0, 127.1, 126.2, 117.9, 114.4, 107.6, 100.4, 55.9.

2-(4-fluorophenyl)-7-methoxy-4H-chromen-4-one (7b) [1b]

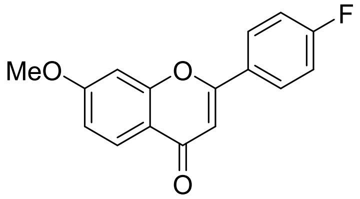

Following General Procedure 2.1, the compound was prepared from 2′-Hydroxy-4′-methoxyacetophenone (0.166 g, 1.0 mmol) and 4-fluorobenzaldehyde (0.136 g, 1.1 mmol, 1.1 equiv) in DMSO (5 mL) and it was purified with columm chromatography on silica gel (AcOEt:Hex = 2:8) as white solid, 214 mg, 79% yield. ^{1}H NMR (500 MHz, $CDCl_3$) δ 8.10 (d, J = 8.8 Hz, 1H), 7.90 – 7.85 (m, 2H), 7.21 – 7.16 (m, 2H), 6.96 (dd, J = 8.9, 2.3 Hz, 1H), 6.92 (d, J = 2.3 Hz, 1H), 6.67 (s, 1H), 3.91 (s, 3H). ^{13}C{^{1}H} NMR (126 MHz, $CDCl_3$) δ 177.6, 165.6, 164.2, 163.6, 162.0, 157.9, 128.3, 128.2, 127.0, 117.6, 116.3, 116.1, 114.4, 107.2, 100.3, 55.8.

7-methoxy-2-(4-(trifluoromethyl)phenyl)-4H-chromen-4-one (7c) [1c]

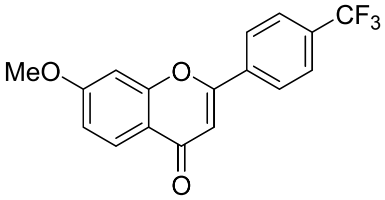

Following General Procedure 2.1, the compound was prepared from 2′-Hydroxy-4′-methoxyacetophenone (0.160 g, 1.0 mmol) and 4-trifluoromethylbenzaldehyde (0.191 g, 1.1 mmol, 1.1 equiv) in DMSO (5 mL) and it was purified with columm chromatography on silica gel (AcOEt:Hex = 2:8) as white solid, 260 mg, 81% yield. ^{1}H NMR (500 MHz, $CDCl_3$) δ 8.05 (dd, J = 8.8, 3.0 Hz, 1H), 7.95 – 7.92 (m, 2H), 7.69 (dd, J = 8.6, 1.9 Hz, 2H), 6.94 – 6.89 (m, 2H), 6.71 (s, 1H), 3.86 (s, 3H). ^{13}C{^{1}H} NMR (101 MHz, $CDCl_3$) δ 177.5, 164.4, 161.2, 157.9, 135.2, 133.1, 127.14, 126.5, 126.0, 122.2, 117.7, 114.7, 108.7, 100.4, 55.9.

7-methoxy-2-(4-methoxyphenyl)-4H-chromen-4-one (7d) [1d]

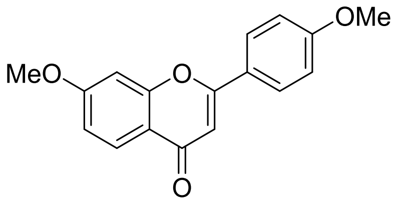

Following General Procedure 2.1, the compound was prepared from 2′-Hydroxy-4′-methoxyacetophenone (0.166 g, 1.0 mmol) and 4-methoxybenzaldehyde (0.149 g, 1.1 mmol, 1.1 equiv) in DMSO (5 mL) and it was purified with columm chromatography on silica gel (AcOEt:Hex = 2:8) as brown solid, 198 mg, 70% yield. ^{1}H NMR (500 MHz, $CDCl_3$) δ 8.02 (d, *J* = 8.8 Hz, 1H), 7.78 – 7.72 (m, 2H), 6.94 – 6.89 (m, 2H), 6.87 (dd, *J* = 8.8, 2.3 Hz, 1H), 6.85 (d, *J* = 2.3 Hz, 1H), 6.58 (s, 1H), 3.83 (s, 3H), 3.79 (s, 3H). ^{13}C{^{1}H}NMR (126 MHz, $CDCl_3$) δ 177.9, 164.0, 163.1, 162.3, 157.9, 127.8, 127.0, 124.0, 117.7, 114.4, 114.2, 106.0, 100.4, 55.8, 55.5.

2-(3,4-dimethoxyphenyl)-7-methoxy-4H-chromen-4-one (7e) [1e]

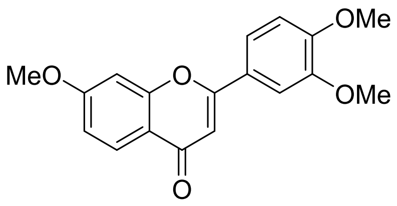

Following General Procedure 2.1, the compound was prepared from 2′-Hydroxy-4′-methoxyacetophenone (0.166 g, 1.0 mmol) and 3,4-dimethoxybenzaldehyde (0.182 g, 1.1 mmol, 1.1 equiv) in DMSO (5 mL) and it was purified with columm chromatography on silica gel (AcOEt:Hex = 2:8) as brown solid, 157 mg, 50% yield. ^{1}H NMR (500 MHz, $CDCl_3$) δ 8.03 (d, *J* = 8,5 Hz, 1H), 7.45 (dd, *J* = 8,5, 2,1 Hz, 1H), 7,27 (d, *J* = 2,1 Hz, 1H), 6,89 (dd, *J* = 8,6, 2,0 Hz, 2H), 6,87 (d, *J* = 2,0 Hz, 1H), 6,61 (s, 1H), 3,90 (s, 3H), 3,88 (s, 3H), 3,85 (s, 3H). ^{13}C{^{1}H} NMR (126 MHz, $CDCl_3$) δ 177.8, 164.1, 163.0, 157.9, 151.9, 149.2, 127.0, 124.3, 119.8, 117.7, 114.2, 111.1, 108.8, 106.3, 100.4, 56.08, 56.06, 55.8.

2-(3,4-dimethoxyphenyl)-6-methoxy-4H-chromen-4-one (7f) [1f]

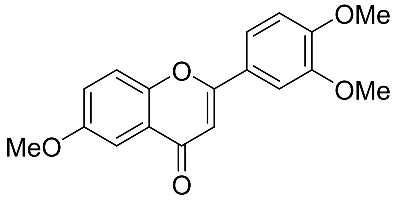

Following General Procedure 2.1, the compound was prepared from 2′-Hydroxy-5′-methoxyacetophenone (0.166 g, 1.0 mmol) and 3,4-dimethoxybenzaldehyde (0.182 g, 1.1 mmol, 1.1 equiv) in DMSO (5 mL) and it was purified with columm chromatography on silica gel (AcOEt:Hex = 5:5) as yellow solid, 247 mg, 79% yield. ^{1}H NMR (500 MHz,

$CDCl_3$) δ 7.60 (d, *J* = 2.2 Hz, 1H), 7.56 (dd, *J* = 8.5, 1.8 Hz, 1H), 7.51 (d, *J* = 9.2, 1.5 Hz, 1H), 7.39 (d, *J* = 1.8 Hz, 1H), 7.31 – 7.27 (dd, *J* = 8.8, 2.8 Hz 1H), 6.99 (d, *J* = 8.5 Hz, 1H), 6.77 (s, 1H), 3.99 (s, 3H), 3.97 (s, 3H), 3.92 (s, 3H). $^{13}C\{^1H\}$ NMR (126 MHz, $CDCl_3$) δ 178.3, 163.3, 157.0, 152.0, 151.0, 149.3, 124.4, 124.3, 123.7, 120.0, 119.4, 111.2, 108.8, 105.7, 104.9, 56.1, 56.1, 55.9.

2.2 Synthesis of 7-bromo-2-phenyl-4H-chromen-4-one (7g)

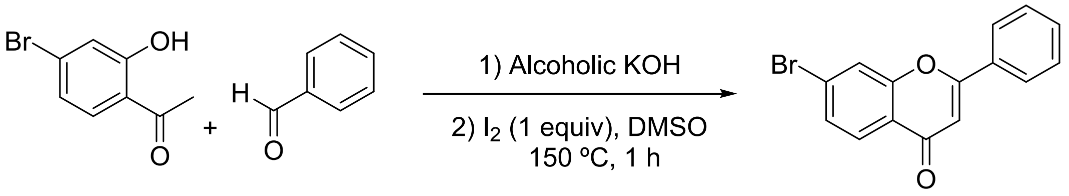

The 7-bromo-2-phenyl-4H-chromen-4-one (**7g**) was prepared according to a standard procedure previously reported by Young-SooKeum et al.[1g] 4'-Bromo-2'-hydroxyacetophenone (0.215 g, 1.0 mmol, 1 equiv) and benzaldehyde (0.106 g, 1.0 mmol, 1.0 equiv) were dissolved in 30% KOH in ethanol (5 mL). The reaction mixture was stirred at 60 ºC for 12 h. After cooling to room temperature, the mixture was neutralized with 1 N HCl and distilled water (10 mL) was added. The resulting chalcone was dissolved in DMSO (5 mL), followed by the addition of I_2 (0.254 g, 1.0 mmol, 1 equiv). The mixture was stirred at 150 ºC for 1 h. After cooling to room temperature, the reaction was quenched with saturated sodium thiosulfate solution (5 mL). The crude product was collected by vacuum filtration and purified by flash column chromatography on silica gel using (AcOEt:Hex = 2:8) as yellow solid, 247 mg, 82% yield.

^1H NMR (500 MHz, $CDCl_3$) δ 8.03 (d, *J* = 8.5 Hz, 1H), 7.84 (dd, *J* = 8.0, 1.7 Hz, 2H), 7.72 (d, *J* = 1.8 Hz, 1H), 7.51 – 7.44 (m, 4H), 6.76 (s, 1H).

2.3 Synthesis of 7-hydroxy-2-phenyl-4*H*-chromen-4-one derivatives

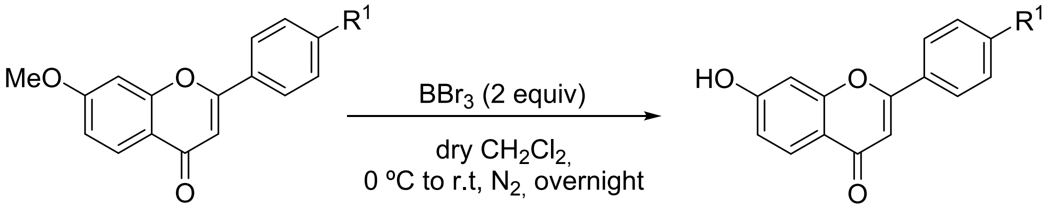

To a solution of 7-methoxy-2-phenyl-4H-chromen-4-one derivatives (**7a-e**) (0.5 mmol) in dry dichloromethane (2 mL) were added boron tribromide (200 µL, 2.5 equiv) dropwise at 0 ºC under a nitrogen atmosphere, according reported by Park et al.[2a] The

reaction mixture was then stirred at room temperature for overnight. Upon completion, the reaction was quenched by the careful dropwise addition of saturated aqueous ammonium chloride solution (5 mL) at 0 ºC. The mixture was extracted with dichloromethane (3 x 20 mL), and the combined organic layers were died over anhydrous sodium sulfate, filtered, and concentrated under reduced pressure. The resulting crude product was used in the subsequent step without further purification.

7-hydroxy-2-phenyl-4H-chromen-4-one [2a]

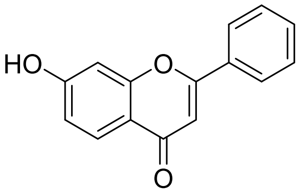

Following General Procedure 2.3, the compound was prepared from 7-methoxy-2-phenyl-4H-chromen-4-one (**7a**) (0.126 g, 0.5 mmol). The crude product was obtained as brown solid 67 mg, 56% yield. ^{1}H NMR (500 MHz, Methanol-d_4) δ 7.92 – 7.89 (m, 3H), 7.48 (m, 3H), 6.91 (d, J = 2.2 Hz, 1H), 6.86 (dd, J = 8.8, 2.3 Hz, 1H), 6.71 (s, 1H).

2-(4-fluorophenyl)-7-hydroxy-4H-chromen-4-one [2b]

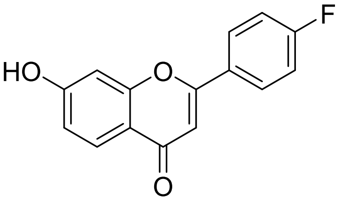

Following General Procedure 2.3, the compound was prepared from 2-(4-fluorophenyl)-7-methoxy-4H-chromen-4-one (**7b**) (0.135 g, 0.5 mmol). The crude product was obtained as brown solid 119 mg, 93% yield. ^{1}H NMR (400 MHz, Methanol-d_4) δ 8.00 – 7.93 (m, 2H), 7.89 (d, J = 8.7 Hz, 1H), 7.20 (t, J = 8.7 Hz, 2H), 6.90 (d, J = 2.3 Hz, 1H), 6.85 (dd, J = 8.7, 2.3 Hz, 1H), 6.68 (s, 1H).

7-hydroxy-2-(4-(trifluoromethyl)phenyl)-4H-chromen-4-one [2b]

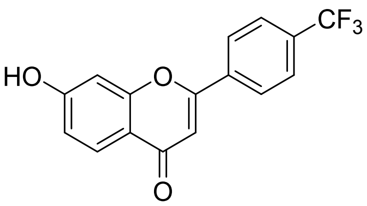

Following General Procedure 2.3, the compound was prepared from 7-methoxy-2-(4-(trifluoromethyl)phenyl)-4H-chromen-4-one (**7c**) (0.160 g, 0.5 mmol). The crude product was obtained as brown solid 121 mg, 79% yield. ^{1}H NMR (500 MHz, Methanol-d_4) δ 8.12 (m, 2H), 7.92 (d, J = 8.8 Hz, 1H), 7.79 (m, 2H), 7.72 – 7.69 (m, 1H), 6.95 (d, J = 2.3 Hz, 1H), 6.89 (dd, J = 8.8, 2.3 Hz, 1H), 6.84 (s, 1H).

2.4 **Synthesis of 4-oxo-2-phenyl-4H-chromen-7-yl diethylcarbamate derivatives (10a-c)**

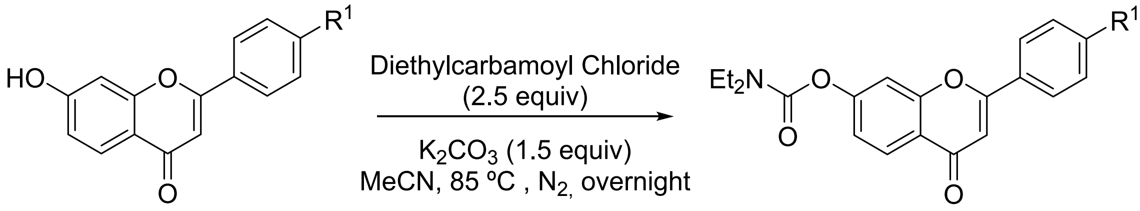

To a solution of 7-hydroxy-2-phenyl-4H-chromen-4-one derivatives (1 mmol) in HPLC-grade acetonitrile (10 mL) was added potassium carbonate (0.207 g, 1.5 equiv), followed by dropwise addition of diethylcarbamoyl chloride (0.339 g, 2.5 mmol, 2.5 equiv), according reported by Liu et al.[3] The reaction mixture was stirred in an oil bath at 85 ºC overnight under a nitrogen atmosphere. After cooling to room temperature, the reaction mixture was extracted with ethyl acetate (3 x 20 mL), and the combined organic layers were washed with brine, dried over anhydrous sodium sulfate, filtered, and concentrated under reduced pressure. The crude residue was purified by on silica gel column chromatography using a gradiente of hexanes and ethyl acetate to afford the desired product.

4-oxo-2-phenyl-4H-chromen-7-yl diethylcarbamate (10a)[3]

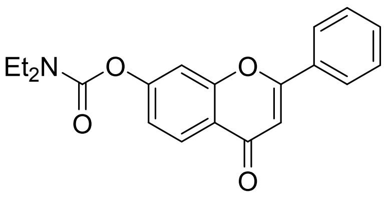

Following General Procedure 2.4, the compound was prepared from 7-hydroxy-2-phenyl-4*H*-chromen-4-one (0.238 g, 1 mmol) in HPLC-grade acetonitrile (10 mL) and it was purified with columm chromatography on silica gel (AcOEt:Hex = 3:7) as yellow solid, 280 mg, 83% yield. MP: 81-83 ºC. ^{1}H NMR (500 MHz, $CDCl_3$) δ 8.22 (d, J = 8.7 Hz, 1H), 7.93 – 7.89 (m, 2H), 7.55 – 7.50 (m, 3H), 7.48 (d, J = 2.2 Hz, 1H), 7.20 (dd, J = 8.7, 2.2 Hz, 1H), 6.81 (s, 1H), 3.48 (q, 2H), 3.43 (q, 2H), 1.29 (t, 3H), 1.24 (t, 3H). ^{13}C{^{1}H}

NMR (126 MHz, $CDCl_3$) δ 177.9, 163.6, 156.8, 155.7, 153.0, 131.7, 131.6, 129.1, 126.8, 126.3, 121.1, 119.5, 110.8, 107.6, 42.5, 42.1, 14.3, 13.3.

2-(4-fluorophenyl)-4-oxo-4H-chromen-7-yl dimethylcarbamate (10b)

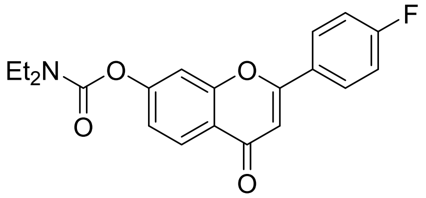

Following General Procedure 2.4, the compound was prepared from 2-(4-fluorophenyl)-7-hydroxy-4H-chromen-4-one (**7b**) (0.256 g, 1 mmol) in HPLC-grade acetonitrile (10 mL) and it was purified with columm chromatography on silica gel (AcOEt:Hex = 3:7) as white solid, 231 mg, 65% yield. MP: 97-99 ºC. ^{1}H NMR (500 MHz, $CDCl_3$) δ 8.22 (d, *J* = 8.7 Hz, 1H), 7.93 – 7.89 (m, 2H), 7.47 (d, *J* = 2.1 Hz, 1H), 7.24 – 7.19 (m, 3H), 6.74 (s, 1H), 3.48 (q, 2H), 3.43 (q, 2H), 1.29 (t, 3H), 1.24 (t, 3H). $^{13}C\{^1H\}$ NMR (101 MHz, $CDCl_3$) δ 177.8, 166.0, 163.5, 162.6, 156.7, 155.8, 153.0, 128.5, 128.4, 126.8, 121.0, 119.6, 116.4, 116.2, 110.8, 107.4, 42.5, 42.1, 14.3, 13.3. HRMS (ESI) m/z $[M+H]^+$ calcd. for $C_{20}H_{18}FNO_4$ 356.1292, found 356.1295.

4-oxo-2-(4-(trifluoromethyl)phenyl)-4H-chromen-7-yl dimethylcarbamate (10c)

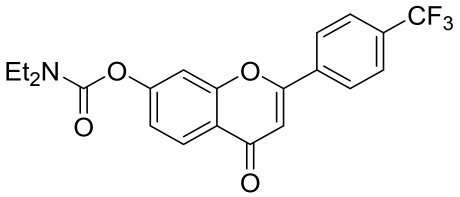

Following General Procedure 2.4, the compound was prepared from 7-hydroxy-2-(4-(trifluoromethyl)phenyl)-4H-chromen-4-one (0.306 g, 1 mmol, 1 equiv) in HPLC-grade acetonitrile (10 mL) and it was purified with columm chromatography on silica gel (AcOEt:Hex = 2:8) as white solid, 142 mg, 35% yield. MP: 65-67 ºC. ^{1}H NMR (500 MHz, $CDCl_3$) δ 8.22 (d, *J* = 8.7 Hz, 1H), 8.02 (d, *J* = 8.2 Hz, 2H), 7.79 (d, *J* = 8.2 Hz, 2H), 7.51 (d, *J* = 2.2 Hz, 1H), 7.22 (dd, *J* = 8.7, 2.2 Hz, 1H), 6.84 (s, 1H), 3.49 (q, 2H), 3.44 (q, 2H), 1.30 (t, 3H), 1.25 (t, 3H). $^{13}C\{^1H\}$ NMR (126 MHz, $CDCl_3$) δ 177.6, 161.7, 156.7, 156.0, 153.0, 135.1, 133.0, 126.9, 126.6, 126.1, 126.0, 121.0, 119.8, 110.8, 108.7, 42.5, 42.1, 14.3, 13.3. HRMS (ESI) m/z $[M+H]^+$ calcd. for $C_{21}H_{18}F_3NO_4$ 406.1188, found 406.1265.

2.5 Synthesis of 4-acetyl-3-hydroxyphenyl diethylcarbamate

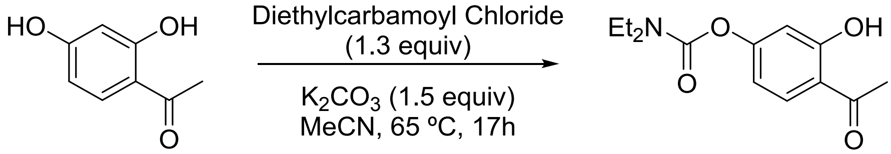

The 4-acetyl-3-hydroxyphenyl diethylcarbamate was prepared through a standard procedure according reported by Zhou et al.[4] To a solution of the starting material, 2’,4’-dihydroxyacetophenone (0.304 g, 2.0 mmol, 1 equiv), anhydrous K_2CO_3 (0.414 g, 3.0 mmol, 1.5 equiv) and HPLC-grade acetonitrile (10 mL). Diethylcarbamoyl chloride (0.352 g, 2.6 mmol, 1.3 equiv) was added dropwise in ice-bath. The mixture was heated in an oil bath at 65 ºC for 17 hours. After the reaction was completed (monitored by TLC), the mixture was extracted with ethyl acetate (3 x 50 mL) and washed with brine, dried over anhydrous sodium sulfate, and concentrated under reduced pressure. The 4-acetyl-3-hydroxyphenyl diethylcarbamate was purified on a silica gel chromatography using (AcOEt:Hex = 2:8) as light yellow oil, 426 mg, 84% yield.

^{1}H NMR (500 MHz, $CDCl_3$) δ 12.35 (s, 1H), 7.64 (d, *J* = 8.6 Hz, 1H), 6.69 – 6.63 (m, 2H), 3.34 (d, *J* = 7.6 Hz, 2H), 3.31 (d, *J* = 7.6 Hz, 2H), 2.52 (s, 3H), 1.17 (d, *J* = 7.1 Hz, 3H), 1.15 – 1.12 (m, 3H). $^{13}C\{^1H\}$ NMR (126 MHz, $CDCl_3$) δ 202.5, 162.9, 156.8, 151.9, 130.8, 116.0, 111.9, 109.7, 41.4, 41.1, 25.6, 13.2, 12.3.

2.6 Synthesis of 4-oxo-2-phenyl-4H-chromen-7-yl diethylcarbamate derivatives (10d-e)

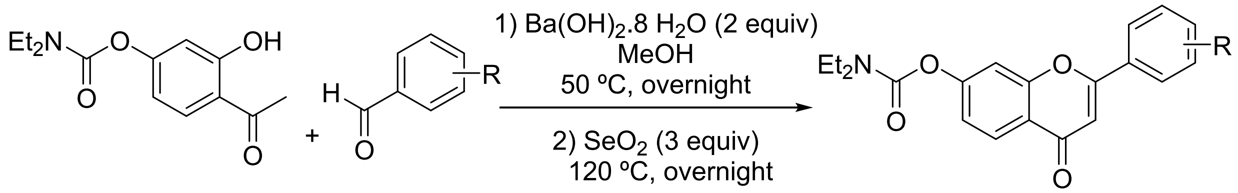

To a solution of benzaldeyde derivatives (0.6 mmol, 1.2 equiv), $Ba(OH)_2.8\ H_2O$ (0.315 g, 1 mmol, 2.0 equiv) in MeOH (3 mL), was added 4-acetyl-3-hydroxyphenyl diethylcarbamate (0.125 g, 0.5 mmol). The reaction mixture was stirred in an oil bath at 50 ºC overnight. After this interval, approximately 50 drops of a 10% HCl solution were added, resulting in the formation of a yellow precipitate, which was collected by vacuum filtration. The residue obtained in this step was dissolved in DMSO (3 mL), and SeO_2 (0.166 g, 1.5 mmol, 3.0 equiv) was added. The reaction mixture was stirred at 120 ºC overnight. After completion, the reaction mixture was extracted with ethyl acetate (4 x 50

mL). The combined organic layers were washed with distilled water (7 x 50 mL), dried over anhydrous sodium sulfate, and concentrated under reduced pressure. The crude product was purified on a silica gel chromatography using a gradient of hexanes and ethyl acetate to afford the desired product.

2-(4-methoxyphenyl)-4-oxo-4H-chromen-7-yl diethylcarbamate (10d)

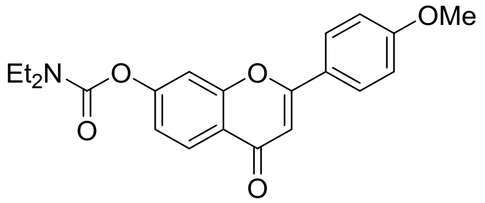

Following General Procedure 2.6, the compound was prepared from 4-acetyl-3-hydroxyphenyl diethylcarbamate (0.125 g, 0.5 mmol, 1 equiv) and 4-methoxybenzaldehyde (0.081 g, 0.6 mmol, 1.2 equiv) and it was purified with columm chromatography on silica gel (AcOEt:Hex = 3:7) as yellow solid, 65 mg, 35% yield. MP: 83-85 ºC. ^{1}H NMR (500 MHz, $CDCl_3$) δ 8.21 (d, J = 8.7 Hz, 1H), 7.87 – 7.84 (m, 2H), 7.45 (d, J = 2.1 Hz, 1H), 7.18 (dd, J = 8.7, 2.2 Hz, 1H), 7.03 – 6.99 (m, 2H), 6.73 (s, 1H), 3.88 (s, 3H), 3.50 – 3.45 (m, 2H), 3.43 (q, J = 7.0 Hz, 2H), 1.29 (t, J = 7.1 Hz, 3H), 1.24 (t, J = 7.2 Hz, 3H). $^{13}C\{^1H\}$ NMR (126 MHz, $CDCl_3$) δ 177.9, 163.6, 162.4, 156.7, 155.6, 153.1, 128.0, 126.7, 123.8, 121.0, 119.3, 114.5, 110.7, 106.1, 55.5, 42.5, 42.1, 14.3, 13.3. HRMS (ESI) m/z $[M+H]^+$ calcd. for $C_{21}H_{21}NO_5$ 368.1420, found 368.1499.

2-(3,4-dimethoxyphenyl)-4-oxo-4H-chromen-7-yl diethylcarbamate (10e)

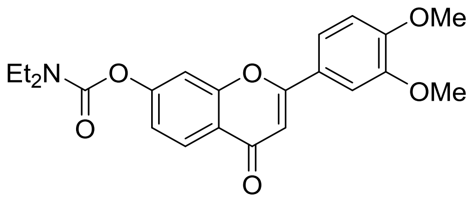

Following General Procedure 2.6, the compound was prepared from 4-acetyl-3-hydroxyphenyl diethylcarbamate (0.125 g, 0.5 mmol, 1 equiv) and 3,4-dimethoxybenzaldehyde (0.099 g, 0.6 mmol, 1.2 equiv) and it was purified with columm chromatography on silica gel (AcOEt:Hex = 3:7) as yellow solid, 135 mg, 68% yield. MP: 139-141 ºC. ^{1}H NMR (400 MHz, $CDCl_3$) δ 8.21 (d, J = 8.7 Hz, 1H), 7.53 (dd, J = 8.5, 2.1 Hz, 1H), 7.49 (d, J = 2.2 Hz, 1H), 7.38 (d, J = 2.1 Hz, 1H), 7.18 (dd, J = 8.7, 2.2 Hz, 1H), 6.98 (d, J = 8.5 Hz, 1H), 6.74 (s, 1H), 3.98 (s, 3H), 3.97 (s, 3H), 3.48 (q, 2H), 3.43 (q, 2H), 1.29 (t, 3H), 1.24 (t, 3H). $^{13}C\{^1H\}$ NMR (126 MHz, $CDCl_3$) δ 177.8, 163.5,

156.6, 155.6, 153.1, 152.1, 149.3, 126.7, 124.1, 121.0, 120.0, 119.3, 111.2, 110.7, 108.7, 106.3, 56.1, 42.5, 42.1, 14.3, 13.3. HRMS (ESI) m/z $[M+H]^+$ calcd. for $C_{22}H_{23}NO_6$ 398.1598, found 398.1606.

2.7 General Procedure for the Ru(II)-Catalyzed C-H Alkenylation of Flavones

In a sealed reaction vial, flavone (0.10 mmol, 1.0 equiv), $[RuCl_2(p\text{-cymene})]_2$ (0.004 mmol, 4 mol%), and $Cu(OAc)_2 \cdot H_2O$ (0.22 mmol, 2.2 equiv), and $AgSbF_6$ (0.020 mmol, 20 mol%) were added.[5] Note: $AgSbF_6$ was handled inside a nitrogen-filled glove bag due to its sensitivity to moisture. The alkene coupling partner (3.0 equiv) and the solvent (1 mL of either 1,2 dimethoxyethane or 1,4 dioxane) were then added. The reaction mixture was stirred in an oil bath at 100 ºC under nitrogen atmosphere for 18 hours. After cooling to the room temperature, the reaction mixture was diluted with ethyl acetate and filtered through a short pad of Celite. The filtrate was washed with satured aqueous ammonium chloride, dried over anhydrous sodium sulfate, and concentrated under reduced pressure. The crude product was purified by flash column chromatography on silica gel using a gradiente of hexanes and ethyl acetate as the eluent to afford the desired alkenylated flavone.

methyl (E)-3-(7-methoxy-4-oxo-2-phenyl-4H-chromen-5-yl)acrylate (9a)

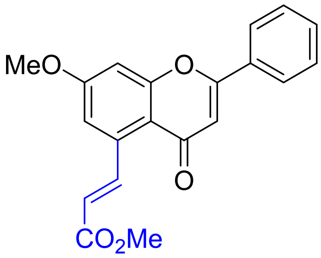

Following General Procedure 2.7, the compound was prepared from 7-methoxy-2-phenyl-4H-chromen-4-one (**7a**) (25.2 mg, 0.1 mmol, 1 equiv) and methyl acrylate (**8a**) (25.8 mg, 0.3 mmol, 3.0 equiv) in 1,4-dioxane (1 mL) and it was purified with columm chromatography on silica gel (AcOEt:Hex = 2:8) as yellow solid, 30 mg, 89% yield. MP: 155-157 ºC. ^1H NMR (500 MHz, $CDCl_3$) δ 8.99 (d, J = 15.9 Hz, 1H), 7.91 – 7.87 (m, 2H), 7.54 – 7.50 (m, 3H), 7.01 (d, J = 2.4 Hz, 1H), 7.00 (d, J = 2.4 Hz, 1H), 6.71 (s, 1H), 6.24 (d, J = 15.9 Hz, 1H), 3.94 (s, 3H), 3.83 (s, 3H). $^{13}C\{^1H\}$ NMR (126 MHz, $CDCl_3$) δ 178.9, 166.9, 162.9, 161.8, 159.0, 144.8, 138.3, 131.5, 131.3, 129.0, 126.1, 121.2, 115.6,

113.5, 108.6, 101.9, 55.9, 51.8. HRMS (ESI) m/z $[M+H]^+$ calcd. for $C_{20}H_{16}O_5$ 337.0998; found 337.1069.

ethyl (E)-3-(7-methoxy-4-oxo-2-phenyl-4H-chromen-5-yl)acrylate (9b)

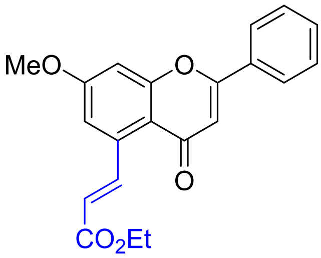

Following General Procedure 2.7, the compound was prepared from 7-methoxy-2-phenyl-4H-chromen-4-one (**7a**) (25.2 mg, 0.1 mmol, 1 equiv) and ethyl acrylate (**8b**) (30.0 mg, 0.3 mmol, 3.0 equiv) in 1,4-dioxane (1 mL) and it was purified with columm chromatography on silica gel (AcOEt:Hex = 2:8) as white solid, 24 mg, 69% yield. MP: 157-159 ºC. ^{1}H NMR (400 MHz, $CDCl_3$) δ 8.98 (d, J = 15.9 Hz, 1H), 7.90 – 7.85 (m, 2H), 7.55 – 7.49 (m, 3H), 7.02 – 6.96 (m, 2H), 6.70 (s, 1H), 6.23 (d, J = 15.9 Hz, 1H), 4.29 (q, J = 7.2 Hz, 2H), 3.93 (d, J = 2.4 Hz, 3H), 1.36 (t, J = 7.1 Hz, 3H). $^{13}C\{^1H\}$ NMR (126 MHz, $CDCl_3$) δ 178.9, 166.5, 162.8, 161.8, 159.0, 144.5, 138.4, 131.5, 131.3, 129.0, 126.1, 121.7, 115.6, 113.5, 108.6, 101.8, 60.6, 55.9, 14.3. HRMS (ESI) m/z $[M+H]^+$ calcd. for $C_{21}H_{18}O_5$ 351.1154, found 351.1230.

n-butyl (E)-3-(7-methoxy-4-oxo-2-phenyl-4H-chromen-5-yl)acrylate (9c)

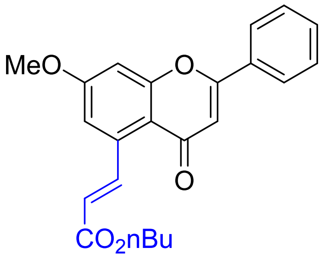

Following General Procedure 2.7, the compound was prepared from 7-methoxy-2-phenyl-4H-chromen-4-one (**7a**) (25.2 mg, 0.1 mmol, 1 equiv) and n-butyl acrylate (**8c**) (38.4 mg, 0.3 mmol, 3.0 equiv) in 1,4-dioxane (1 mL) and it was purified with columm chromatography on silica gel (AcOEt:Hex = 2:8) as white solid, 17 mg, 49% yield. MP: 155-157 ºC. ^{1}H NMR (500 MHz, $CDCl_3$) δ 9.00 (d, J = 15.9 Hz, 1H), 7.92 – 7.88 (m, 2H), 7.54 – 7.50 (m, 3H), 7.04 – 6.99 (m, 2H), 6.72 (s, 1H), 6.24 (d, J = 15.9 Hz, 1H), 4.24 (t, 2H), 3.95 (s, 3H), 1.75 – 1.70 (m, 2H), 1.51 – 1.43 (m, 2H), 0.98 (d, 3H). $^{13}C\{^1H\}$ NMR (126 MHz, $CDCl_3$) δ 178.9, 166.6, 162.9, 161.8, 159.0, 144.5, 138.5, 131.5, 131.4,

129.0, 126.1, 121.7, 115.6, 113.5, 108.6, 101.9, 64.5, 55.9, 30.8, 19.2, 13.8. HRMS (ESI) m/z $[M+H]^+$ calcd. for $C_{23}H_{22}O_5$ 379.1467, found 379.1545.

(E)-7-methoxy-2-phenyl-5-styryl-4H-chromen-4-one (9e)

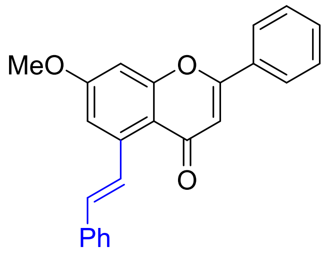

Following General Procedure 2.7, the compound was prepared from 7-methoxy-2-phenyl-4H-chromen-4-one (**7a**) (25.2 mg, 0.1 mmol, 1 equiv) and styrene (**8d**) (31.2 mg, 0.3 mmol, 3.0 equiv) in 1,4-dioxane (1 mL) and it was purified with columm chromatography on silica gel (AcOEt:Hex = 2:8) as yellow solid, 22 mg, 62% yield. MP: 167-169 ºC. ^1H NMR (400 MHz, $CDCl_3$) δ 8.70 (d, *J* = 16.2 Hz, 1H), 7.92 – 7.87 (m, 2H), 7.64 – 7.59 (m, 2H), 7.51 (qd, *J* = 4.3, 1.6 Hz, 3H), 7.40 – 7.34 (m, 2H), 7.30 – 7.25 (m, 1H), 7.16 (d, *J* = 2.5 Hz, 1H), 6.98 (d, *J* = 16.2 Hz, 1H), 6.90 (d, *J* = 2.5 Hz, 1H), 6.69 (s, 1H), 3.94 (s, 3H). $^{13}C\{^1H\}$ NMR (101 MHz, $CDCl_3$) δ 179.6, 162.8, 161.2, 159.3, 141.5, 137.4, 131.92, 131.5, 131.3, 129.0, 128.6, 128.3, 127.9, 127.1, 126.1, 115.2, 111.6, 108.8, 100.1, 55.8. HRMS (ESI) m/z $[M+H]^+$ calcd. for $C_{24}H_{18}O_3$ 355.1256, found 355.1333.

(E)-7-methoxy-5-(3-oxobut-1-en-1-yl)-2-phenyl-4H-chromen-4-one (9f)

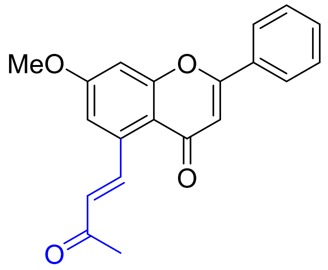

Following General Procedure 2.7, the compound was prepared from 7-methoxy-2-phenyl-4H-chromen-4-one (**7a**) (25.2 mg, 0.1 mmol, 1 equiv) and methyl vinyl ketone (**8f**) (21.0 mg, 0.3 mmol, 3.0 equiv) in 1,4-dioxane (1 mL) and it was purified with column chromatography on silica gel (AcOEt:Hex = 2:8) as brown solid, 21 mg, 66% yield. MP: 160-162 ºC. ^1H NMR (400 MHz, $CDCl_3$) δ 8.99 (d, *J* = 16.4, 1H), 7.92 – 7.87 (m, 2H), 7.57 – 7.50 (m, 3H), 7.05 (d, *J* = 2.5, 1H), 7.01 (d, *J* = 2.5 Hz, 1H), 6.71 (s, 1H),

6.44 (d, J = 16.4 Hz, 1H), 3.94 (s, 3H), 2.49 (s, 3H). $^{13}C\{^{1}H\}$ NMR (101 MHz, $CDCl_3$) δ 199.7, 179.2, 162.9, 162.0, 159.1, 144.2, 138.4, 131.6, 131.2, 131.1, 129.1, 126.1, 115.4, 113.2, 108.6, 102.2, 55.9, 26.3. HRMS (ESI) m/z $[M+H]^+$ calcd. for $C_{20}H_{16}O_4$ 321.1049, found 321.1127.

(E)-7-methoxy-2-phenyl-5-(2-(phenylsulfonyl)vinyl)-4H-chromen-4-one (9g)

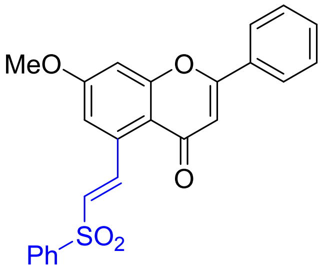

Following General Procedure 2.7, the compound was prepared from 7-methoxy-2-phenyl-4H-chromen-4-one (**7a**) (25.2 mg, 0.1 mmol, 1 equiv) and phenyl vinyl sulfone (**8g**) (50.4 mg, 0.3 mmol, 3.0 equiv) in 1,4-dioxane (1 mL) and it was purified with column chromatography on silica gel (AcOEt:Hex = 2:8) as yellow solid, 23 mg, 55% yield. MP: 164-166 ºC. ^{1}H NMR (400 MHz, $CDCl_3$) δ 8.95 (d, J = 15.3 Hz, 1H), 8.12 – 8.09 (m, 2H), 7.90 – 7.87 (m, 2H), 7.67 – 7.47 (m, 6H), 7.02 (d, J = 2.5 Hz, 1H), 6.94 (d, J = 2.5 Hz, 1H), 6.72 (s, 1H), 6.66 (d, J = 15.3 Hz, 1H), 3.93 (s, 3H). $^{13}C\{^{1}H\}$ NMR (101 MHz, $CDCl_3$) δ 178.5, 162.9, 162.2, 158.9, 144.0, 140.5, 136.2, 133.3, 131.6, 131.2, 130.0, 129.3, 129.1, 128.0, 126.1, 115.7, 113.9, 108.4, 102.4, 56.0. HRMS (ESI) m/z $[M+Na]^+$ calcd. for $C_{24}H_{18}O_5S$ 441.0875, found 441.0776.

methyl (E)-3-(2-(4-fluorophenyl)-7-methoxy-4-oxo-4H-chromen-5-yl)acrylate (9k)

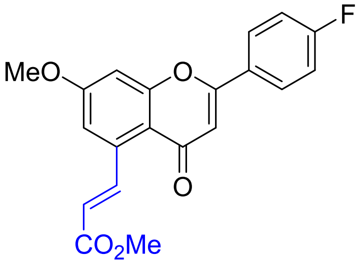

Following General Procedure 2.7, the compound was prepared from 2-(4-fluorophenyl)-7-methoxy-4H-chromen-4-one (**7b**) (27.0 mg, 0.1 mmol, 1 equiv) and methyl acrylate (**8a**) (25.8 mg, 0.3 mmol, 3.0 equiv) in DME (1 mL) and it was purified with columm chromatography on silica gel (AcOEt:Hex = 2:8) as white solid, 17 mg, 50% yield. MP: 195-197 ºC. ^{1}H NMR (400 MHz, $CDCl_3$) δ 8.96 (d, J = 15.9 Hz, 1H), 7.93 – 7.86 (m,

2H), 7.21 (dd, *J* = 9.4, 7.8 Hz, 2H), 7.01 (d, *J* = 2.5 Hz, 1H), 6.99 (d, *J* = 2.5 Hz, 1H), 6.65 (s, 1H), 6.24 (d, *J* = 15.9 Hz, 1H), 3.95 (s, 3H), 3.83 (s, 3H). $^{13}C\{^{1}H\}$ NMR (126 MHz, $CDCl_3$) δ 178.7, 166.9, 165.7, 163.7, 162.9, 160.9, 159.0, 144.7, 138.4, 128.4, 128.3, 121.3, 116.4, 116.2, 115.5, 113.5, 108.4, 101.9, 55.9, 51.8. HRMS (ESI) m/z $[M+H]^+$ calcd. for $C_{20}H_{15}FO_5$ 355.0904, found 355.0980.

ethyl (E)-3-(2-(4-fluorophenyl)-7-methoxy-4-oxo-4H-chromen-5-yl)acrylate (9l)

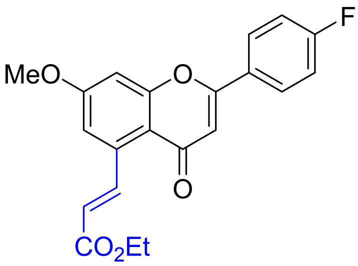

Following General Procedure 2.7, the compound was prepared from 2-(4-fluorophenyl)-7-methoxy-4H-chromen-4-one (**7b**) (27.0 mg, 0.1 mmol, 1 equiv) and ethyl acrylate (**8b**) (30.0 mg, 0.3 mmol, 3.0 equiv) in DME (1 mL) and it was purified with columm chromatography on silica gel (AcOEt:Hex = 2:8) as white solid, 33 mg, 90% yield. MP: 189-191 ºC. ^{1}H NMR (500 MHz, $CDCl_3$) δ 8.97 (d, *J* = 15.9 Hz, 1H), 7.91 – 7.87 (m, 2H), 7.23 – 7.19 (m, 2H), 7.01 (d, *J* = 2.5 Hz, 1H), 6.98 (d, *J* = 2.5 Hz, 1H), 6.65 (s, 1H), 6.24 (d, *J* = 15.9 Hz, 1H), 4.30 (q, 2H), 3.94 (s, 3H), 1.36 (t, 3H). $^{13}C\{^{1}H\}$ NMR (126 MHz, $CDCl_3$) δ 178.7, 166.4, 165.7, 163.7, 162.9, 160.8, 158.9, 144.4, 138.5, 128.34, 128.27, 121.8, 116.4, 116.2, 115.5, 113.5, 108.4, 101.8, 60.6, 55.9, 14.3. HRMS (ESI) m/z $[M+H]^+$ calcd. for $C_{21}H_{17}FO_5$ 369.1060, found 369.1140.

n-butyl (E)-3-(2-(4-fluorophenyl)-7-methoxy-4-oxo-4H-chromen-5-yl)acrylate (9m)

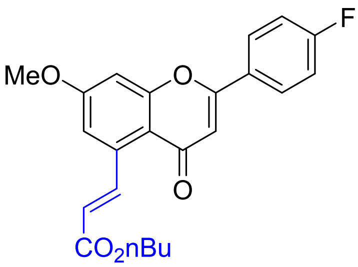

Following General Procedure 2.7, the compound was prepared from 2-(4-fluorophenyl)-7-methoxy-4H-chromen-4-one (**7b**) (27.0 mg, 0.1 mmol, 1 equiv) and n-butyl acrylate (**8c**) (38.4 mg, 0.3 mmol, 3.0 equiv) in DME (1 mL) and it was purified with columm chromatography on silica gel (AcOEt:Hex = 3:7) as yellow solid, 30.5 mg, 77% yield.

The compound was synthesized using general procedure 2.6. Yield: 77%; yellow solid. MP: 157-159 ºC. ^{1}H NMR (500 MHz, $CDCl_3$) δ 8.97 (d, *J* = 15.9 Hz, 1H), 7.91 – 7.85 (m, 2H), 7.23 – 7.18 (m, 2H), 7.01 (d, *J* = 2.6 Hz, 1H), 6.97 (d, *J* = 2.6 Hz, 1H), 6.65 (s, 1H), 6.24 (d, *J* = 15.9 Hz, 1H), 4.24 (t, 2H), 3.94 (s, 3H), 1.75 – 1.70 (m, 2H), 1.50 – 1.43 (m, 2H), 0.98 (t, 3H). ^{13}C{^{1}H} NMR (126 MHz, $CDCl_3$) δ 178.7, 166.5, 165.7, 163.7, 162.9, 160.8, 158.9, 144.4, 138.5, 128.3, 128.3, 121.8, 116.3, 116.2, 115.4, 113.5, 108.3, 101.8, 64.5, 55.9, 30.8, 19.2, 13.8. HRMS (ESI) m/z $[M+H]^+$ calcd. for $C_{23}H_{21}FO_5$ 397.1373, found 397.1449.

methyl (E)-3-(7-methoxy-4-oxo-2-(4-(trifluoromethyl)phenyl)-4H-chromen-5-yl)acrylate (9n)

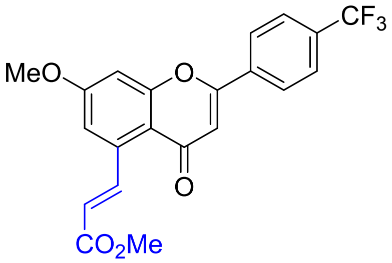

Following General Procedure 2.7, the compound was prepared from 7-methoxy-2-(4-(trifluoromethyl)phenyl)-4H-chromen-4-one (**7c**) (32.0 mg, 0.1 mmol, 1 equiv) and methyl acrylate (**8a**) (25.8 mg, 0.3 mmol, 3.0 equiv) in DME (1 mL) and it was purified with columm chromatography on silica gel (AcOEt:Hex = 2:8) as white solid, 29 mg, 72% yield. MP: 189-191 ºC. ^{1}H NMR (500 MHz, $CDCl_3$) δ 8.96 (d, *J* = 15.9 Hz, 1H), 8.02 (d, *J* = 8.2 Hz, 2H), 7.78 (d, *J* = 8.2 Hz, 2H), 7.04 – 7.01 (m, 2H), 6.76 (s, 1H), 6.25 (d, *J* = 15.9 Hz, 1H), 3.96 (s, 3H), 3.84 (s, 3H). ^{13}C{^{1}H} NMR (126 MHz, $CDCl_3$) δ 178.5, 166.8, 163.1, 160.0, 159.0, 144.5, 138.4, 134.7, 133.2, 132.9, 126.4, 126.0, 121.4, 115.5, 113.8, 109.8, 101.8, 56.0, 51.8. HRMS (ESI) m/z $[M+H]^+$ calcd. for $C_{21}H_{15}F_3O_5$ 405.0872, found 405.0946.

ethyl (E)-3-(7-methoxy-4-oxo-2-(4-(trifluoromethyl)phenyl)-4H-chromen-5-yl)acrylate (9o)

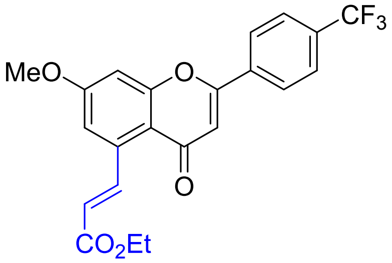

Following General Procedure 2.7, the compound was prepared from 7-methoxy-2-(4-(trifluoromethyl)phenyl)-4H-chromen-4-one (**7c**) (32.0 mg, 0.1 mmol, 1 equiv) and ethyl acrylate (**8b**) (30.0 mg, 0.3 mmol, 3.0 equiv) in DME (1 mL) and it was purified with column chromatography on silica gel (AcOEt:Hex = 3:7) as white solid, 28 mg, 67% yield. MP: 198-200 ºC. ^{1}H NMR (500 MHz, $CDCl_3$) δ 8.96 (d, J = 15.9 Hz, 1H), 8.03 – 8.00 (m, 2H), 7.79 – 7.77 (m, 2H), 7.05 (d, J = 2.5, 1H), 7.01 (d, J = 2.5 Hz, 1H), 6.77 (s, 1H), 6.25 (d, J = 15.9 Hz, 1H), 4.30 (q, 2H), 3.96 (s, 3H), 1.37 (t, 3H). $^{13}C\{^{1}H\}$ NMR (126 MHz, $CDCl_3$) δ 178.6, 166.4, 163.1, 160.1, 159.0, 144.3, 138.6, 134.7, 133.0, 126.5, 126.0, 124.7, 122.0, 115.6, 113.8, 109.8, 101.8, 60.7, 56.0, 14.3. HRMS (ESI) m/z $[M+H]^+$ calcd. for $C_{22}H_{17}F_3O_5$ 419.1028, found 419.1109.

n-butyl (E)-3-(7-methoxy-4-oxo-2-(4-(trifluoromethyl)phenyl)-4H-chromen-5-yl)acrylate (9p)

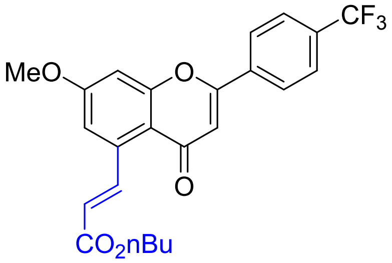

Following General Procedure 2.7, the compound was prepared from 7-methoxy-2-(4-(trifluoromethyl)phenyl)-4H-chromen-4-one (**7c**) (32.0 mg, 0.1 mmol, 1 equiv) and n-butyl acrylate (**8c**) (38.4 mg, 0.3 mmol, 3.0 equiv) in DME (1 mL) and it was purified with column chromatography on silica gel (AcOEt:Hex = 2:8) as brown solid, 34 mg, 76% yield. MP: 188-190 ºC. ^{1}H NMR (500 MHz, $CDCl_3$) δ 8.96 (dd, J = 15.8 Hz, 1H), 8.03 – 7.99 (m, 2H), 7.79 – 7.76 (m, 2H), 7.04 (d, J = 2.5 Hz, 1H), 7.00 (d, J = 2.5 Hz, 1H), 6.76 (s, 1H), 6.25 (d, J = 15.8 Hz, 1H), 4.24 (t, 2H), 3.96 (s, 3H), 1.75 – 1.69 (m, 2H), 1.50 – 1.44 (m, 2H), 0.98 (t, 3H). $^{13}C\{^{1}H\}$ NMR (126 MHz, $CDCl_3$) δ 178.6, 166.5, 163.1, 160.0, 159.0, 144.2, 138.6, 134.8, 133.2, 132.9, 126.4, 126.00, 122.0, 115.5, 113.7,

109.8, 101.8, 64.6, 56.0, 30.8, 19.2, 13.8. HRMS (ESI) m/z $[M+H]^+$ calcd. for $C_{24}H_{21}F_3O_5$ 447.1341, found 447.1421.

methyl (E)-3-(7-methoxy-2-(4-methoxyphenyl)-4-oxo-4H-chromen-5-yl)acrylate (9q)

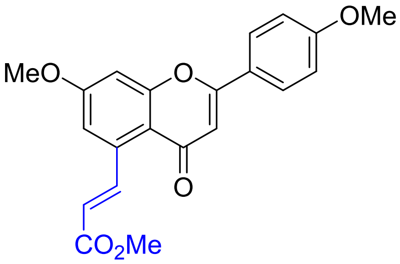

Following General Procedure 2.7, the compound was prepared from 7-methoxy-2-(4-methoxyphenyl)-4H-chromen-4-one (**7d**) (28.2 mg, 0.1 mmol, 1 equiv) and methyl acrylate (**8a**) (25.8 mg, 0.3 mmol, 3.0 equiv) in DME (1 mL) and it was purified with column chromatography on silica gel (AcOEt:Hex = 3:7) as white solid, 11 mg, 40% yield. MP: 170-172 ºC. ^1H NMR (500 MHz, $CDCl_3$) δ 9.01 (d, J = 16.1 Hz, 1H), 7.86 – 7.83 (m, 2H), 7.03 – 6.98 (m, 4H), 6.63 (s, 1H), 6.24 (d, J = 15.9 Hz, 1H), 3.94 (s, 3H), 3.89 (s, 3H), 3.83 (s, 3H). $^{13}C\{^1H\}$ NMR (126 MHz, $CDCl_3$) δ 178.9, 166.9, 162.7, 162.3, 161.9, 158.9, 145.0, 138.3, 127.8, 123.6, 121.1, 115.6, 114.4, 113.3, 107.2, 101.9, 55.9, 55.5, 51.8. HRMS (ESI) m/z $[M+H]^+$ calcd for $C_{21}H_{18}O_6$ 367.1103, found 367.1184.

ethyl (E)-3-(7-methoxy-2-(4-methoxyphenyl)-4-oxo-4H-chromen-5-yl)acrylate (9r)

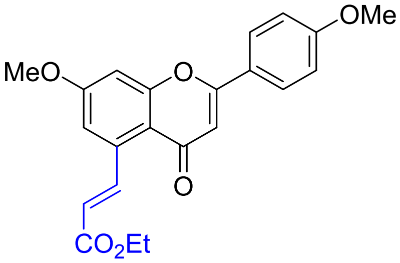

Following General Procedure 2.7, the compound was prepared from 7-methoxy-2-(4-methoxyphenyl)-4H-chromen-4-one (**7d**) (28.2 mg, 0.1 mmol, 1 equiv) and ethyl acrylate (**8b**) (30.0 mg, 0.3 mmol, 3.0 equiv) in DME (1 mL) and it was purified with columm chromatography on silica gel (AcOEt:Hex = 2:8) as yellow solid, 21 mg, 55% yield. MP: 195-197 ºC. ^1H NMR (500 MHz, $CDCl_3$) δ 9.01 (d, J = 15.9 Hz, 1H), 7.86 – 7.82 (m, 2H), 7.04 – 6.99 (m, 3H), 6.98 (d, J = 2.5 Hz, 1H), 6.63 (s, 1H), 6.23 (d, J = 15.9 Hz, 1H), 4.29 (q, 2H), 3.94 (s, 3H), 3.89 (s, 3H), 1.36 (t, 3H). $^{13}C\{^1H\}$ NMR (126 MHz, $CDCl_3$) δ

178.9, 166.5, 162.7, 162.3, 161.9, 158.9, 144.7, 138.4, 127.8, 123.6, 121.6, 115.6, 114.4, 113.2, 107.2, 101.9, 60.6, 55.9, 55.5, 14.3. HRMS (ESI) m/z $[M+H]^+$ calcd. for $C_{22}H_{20}O_6$ 381.1260, found 381.1339.

n-butyl (E)-3-(7-methoxy-2-(4-methoxyphenyl)-4-oxo-4H-chromen-5-yl)acrylate (9s)

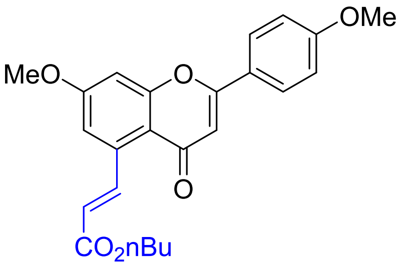

Following General Procedure 2.7, the compound was prepared from 7-methoxy-2-(4-methoxyphenyl)-4H-chromen-4-one (**7d**) (28.2 mg, 0.1 mmol, 1 equiv) and n-butyl acrylate (**8c**) (38.4 mg, 0.3 mmol, 3.0 equiv) in DME (1 mL) and it was purified with column chromatography on silica gel (AcOEt:Hex = 3:7) as yellow solid, 38 mg, 93% yield. MP: 139-141 ºC. ^{1}H NMR (500 MHz, $CDCl_3$) δ 9.00 (d, *J* = 15.9, 1H), 7.83 (dd, *J* = 9.0, 2.2 Hz, 2H), 7.00 (m, 3H), 6.96 (d, J = 2.4 Hz, 1H), 6.62 (s, 1H), 6.23 (d, *J* = 15.9, 1H), 4.23 (t, 2H), 3.94 (s, 3H), 3.89 (s, 3H), 1.74 – 1.70 (m, 2H), 1.50 – 1.44 (m, 2H), 0.97 (t, 3H). ^{13}C{^{1}H} NMR (126 MHz, $CDCl_3$) δ 178.9, 166.6, 162.7, 162.3, 161.8, 158.9, 144.7, 138.4, 127.8, 123.6, 121.5, 115.5, 114.4, 113.2, 107.1, 101.8, 64.5, 55.9, 55.5, 30.8, 19.2, 13.8. HRMS (ESI) m/z $[M+H]^+$ calcd. for $C_{24}H_{24}O_6$ 409.1645, found 409.1649.

methyl (E)-3-(2-(3,4-dimethoxyphenyl)-7-methoxy-4-oxo-4H-chromen-5-yl)acrylate (9t)

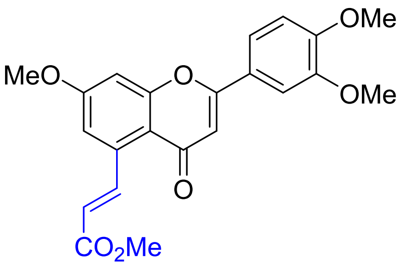

Following General Procedure 2.7, the compound was prepared from 2-(3,4-dimethoxyphenyl)-7-methoxy-4H-chromen-4-one (**7e**) (31.2 mg, 0.1 mmol, 1 equiv) and methyl acrylate (**8a**) (25.8 mg, 0.3 mmol, 3.0 equiv) in DME (1 mL) and it was purified

with columm chromatography on silica gel (AcOEt:Hex = 3:7) as yellow solid, 18 mg, 45% yield. MP: 190-192 ºC. ^{1}H NMR (500 MHz, $CDCl_3$) δ 9.00 (d, *J* = 15.9 Hz, 1H), 7.52 (dd, *J* = 8.4, 2.1 Hz, 1H), 7.33 (d, *J* = 2.2 Hz, 1H), 7.00 (d, *J* = 2.2 Hz, 1H), 6.99 – 6.96 (m, 2H), 6.63 (s, 1H), 6.24 (d, *J* = 15.9 Hz, 1H), 3.99 (s, 3H), 3.97 (s, 3H), 3.95 (s, 3H), 3.84 (s, 3H). $^{13}C\{^{1}H\}$ NMR (126 MHz, $CDCl_3$) δ 178.8, 166.9, 162.7, 161.8, 158.9, 152.0, 149.3, 144.9, 138.2, 123.8, 121.1, 119.8, 115.5, 113.3, 111.1, 108.7, 107.5, 101.9, 56.14, 56.08, 55.9, 51.8. HRMS (ESI) m/z $[M+H]^+$ calcd. for $C_{22}H_{20}O_7$ 397.1209, found 397.1285.

ethyl (E)-3-(2-(3,4-dimethoxyphenyl)-7-methoxy-4-oxo-4H-chromen-5-yl)acrylate (9u)

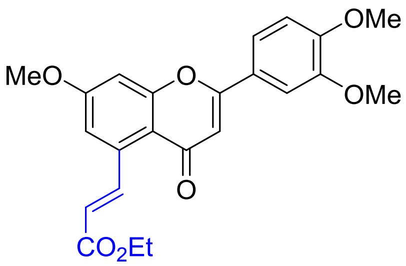

Following General Procedure 2.7, the compound was prepared from 2-(3,4-dimethoxyphenyl)-7-methoxy-4H-chromen-4-one (**7e**) (31.2 mg, 0.1 mmol, 1 equiv) and ethyl acrylate (**8b**) (30.0 mg, 0.3 mmol, 3.0 equiv) in DME (1 mL) and it was purified with columm chromatography on silica gel (AcOEt:Hex = 3:7) as yellow solid, 14 mg, 34% yield. MP: 195-197 ºC. ^{1}H NMR (500 MHz, $CDCl_3$) δ 9.01 (d, *J* = 16.0 Hz, 1H), 7.53 (dd, *J* = 8.4, 2.2 Hz, 1H), 7.34 (d, *J* = 2.5 Hz, 1H), 7.02 (d, *J* = 2.5 Hz, 1H), 6.99 (d, *J* = 2.6 Hz, 1H), 6.97 (s, 1H), 6.64 (s, 1H), 6.24 (d, *J* = 16.0 Hz, 1H), 4.30 (q, 2H), 3.99 (s, 3H), 3.97 (s, 3H), 3.95 (s, 3H), 1.36 (t, 3H). $^{13}C\{^{1}H\}$ NMR (101 MHz, $CDCl_3$) δ 178.9, 166.5, 162.7, 161.8, 158.9, 152.0, 149.3, 144.6, 138.4, 123.8, 121.6, 119.8, 115.6, 113.3, 111.1, 108.7, 107.5, 101.9, 60.6, 56.13, 56.08, 55.9, 14.3. HRMS (ESI) m/z $[M+H]^+$ calcd. for $C_{23}H_{22}O_7$ 411.1366, found 411.1449.

n-butyl (E)-3-(2-(3,4-dimethoxyphenyl)-7-methoxy-4-oxo-4H-chromen-5-yl)acrylate (9v)

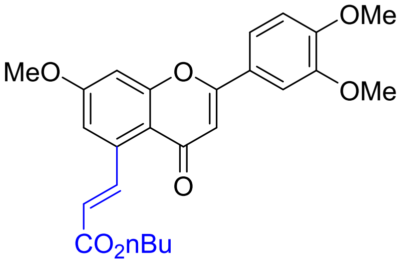

Following General Procedure 2.7, the compound was prepared from 2-(3,4-dimethoxyphenyl)-7-methoxy-4H-chromen-4-one (**7e**) (31.2 mg, 0.1 mmol, 1 equiv) and n-butyl acrylate (**8c**) (38.4 mg, 0.3 mmol, 3.0 equiv) in DME (1 mL) and it was purified with columm chromatography on silica gel (AcOEt:Hex = 3:7) as yellow solid, 14 mg, 32% yield. MP: 145-147 ºC. ^{1}H NMR (500 MHz, $CDCl_3$) δ 9.04 – 8.98 (m, 1H), 7.52 (dd, *J* = 8.5, 2.1 Hz, 1H), 7.34 (d, *J* = 2.2 Hz, 1H), 7.02 (dd, *J* = 2.5, 0.7 Hz, 1H), 6.99 – 6.97 (m, 2H), 6.64 (s, 1H), 6.25 (d, *J* = 15.9 Hz, 1H), 4.24 (t, *J* = 6.7 Hz, 2H), 3.99 (s, 3H), 3.97 (s, 3H), 3.95 (s, 3H), 1.75 – 1.69 (m, 2H), 1.50 – 1.43 (m, 2H), 0.98 (t, *J* = 7.4 Hz, 3H). $^{13}C\{^1H\}$ NMR (101 MHz, $CDCl_3$) δ 178.9, 166.6, 162.7, 161.8, 158.9, 152.0, 149.3, 144.6, 138.4, 123.8, 121.6, 119.8, 115.6, 113.3, 111.1, 108.7, 107.5, 101.9, 64.5, 56.12, 56.08, 55.9, 30.8, 19.2, 13.8. HRMS (ESI) calcd for $C_{25}H_{26}O_7$ 439.1679, found 439.1752.

methyl (E)-3-(2-(3,4-dimethoxyphenyl)-6-methoxy-4-oxo-4H-chromen-5-yl)acrylate (9w)

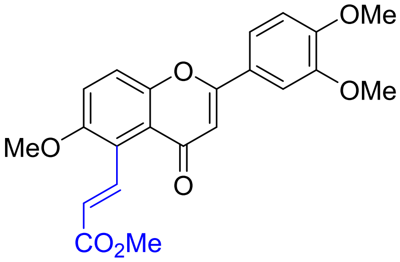

Following General Procedure 2.7, the compound was prepared from 2-(3,4-dimethoxyphenyl)-6-methoxy-4H-chromen-4-one (**7f**) (31.2 mg, 0.1 mmol, 1 equiv) and methyl acrylate (**8a**) (25.8 mg, 0.3 mmol, 3.0 equiv) in 1,4-dioxane (1 mL) and it was purified with columm chromatography on silica gel (AcOEt:Hex = 5:5) as yellow solid, 23 mg, 58% yield. MP: 203-205 ºC. ^{1}H NMR (500 MHz, $CDCl_3$) δ 8.83 (d, *J* = 16.3 Hz, 1H), 7.59 – 7.50 (m, 2H), 7.38 – 7.31 (m, 2H), 6.97 (d, *J* = 8.5 Hz, 1H), 6.66 (s, 1H), 6.59 (d, *J* = 16.3 Hz, 1H), 3.99 (s, 3H), 3.96 (s, 3H), 3.92 (s, 3H), 3.84 (s, 3H). $^{13}C\{^1H\}$ NMR (126 MHz, $cdcl_3$) δ 178.7, 167.0, 160.7, 154.0, 151.0, 150.6, 148.3, 138.4, 122.9, 122.8,

121.50, 121.46, 118.9, 118.6, 116.5, 110.1, 107.7, 106.2, 55.5, 55.12, 55.07, 50.7. HRMS (ESI) m/z $[M+H]^+$ calcd. for $C_{22}H_{20}O_7$ 397.1281, found 397.1285.

methyl (E)-3-(7-bromo-4-oxo-2-phenyl-4H-chromen-5-yl)acrylate (9x)

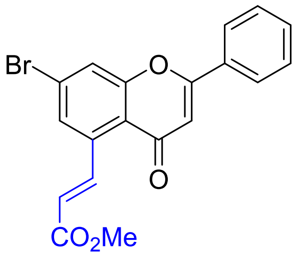

Following General Procedure 2.7, the compound was prepared from 7-bromo-2-phenyl-4H-chromen-4-one (**7g**) (30.1 mg, 0.1 mmol, 1 equiv) and methyl acrylate (**8a**) (51.6 mg, 0.6 mmol, 6.0 equiv) in 1,4-dioxane (1 mL) and it was purified with columm chromatography on silica gel (AcOEt:Hex = 2:8) as white solid, 29 mg, 75% yield. MP: 220-222 ºC. ^{1}H NMR (500 MHz, $CDCl_3$) δ 8.93 (d, J = 15.9 Hz, 1H), 7.89 (d, J = 7.3 Hz, 2H), 7.79 (s, 1H), 7.62 – 7.46 (m, 4H), 6.77 (s, 1H), 6.27 (d, J = 15.9 Hz, 1H), 3.84 (s, 3H). ^{13}C{^{1}H} NMR (126 MHz, $CDCl_3$) δ 178.8, 166.5, 162.2, 157.2, 143.4, 138.4, 131.9, 130.8, 129.1, 127.8, 127.0, 126.2, 122.4, 122.2, 120.3, 108.9, 51.9. HRMS (ESI) m/z $[M+H]^+$ calcd. for $C_{19}H_{13}BrO_7$ 385.0069, found 385.0071.

methyl (E)-3-(7-((diethylcarbamoyl)oxy)-4-oxo-2-phenyl-4H-chromen-5-yl)acrylate (11a)

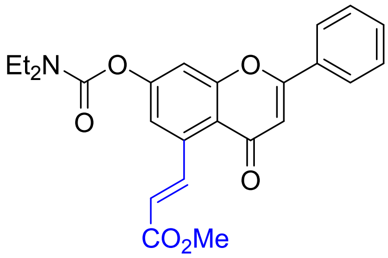

Following General Procedure 2.7, the compound was prepared from 4-oxo-2-phenyl-4H-chromen-7-yl diethylcarbamate (**10a**) (33.7 mg, 0.1 mmol, 1 equiv) and methyl acrylate (**8a**) (25.8 mg, 0.3 mmol, 3.0 equiv) in DME (1 mL) and it was purified with columm chromatography on silica gel (AcOEt:Hex = 2:8) as white solid, 35 mg, 83% yield. MP: 153-155 ºC. ^{1}H NMR (500 MHz, $CDCl_3$) δ 9.02 (d, J = 16.0, 1H), 7.91 – 7.88 (m, 2H), 7.54 – 7.49 (m, 4H), 7.24 (dd, J = 2.3, 0.7 Hz, 1H), 6.76 (s, 1H), 6.29 (d, J = 15.9 Hz, 1H), 3.83 (s, 3H), 3.47 (q, 2H), 3.44 – 3.39 (q, 2H), 1.30 (t, 3H), 1.24 (t, 3H). ^{13}C{^{1}H} NMR (126 MHz, $CDCl_3$) δ 179.0, 166.8, 162.3, 157.8, 154.6, 152.8, 144.2, 138.1, 131.7,

131.1, 129.1, 126.2, 121.7, 118.7, 118.6, 112.1, 108.7, 51.8, 42.6, 42.2, 14.3, 13.3. HRMS (ESI) m/z $[M+H]^+$ calcd. for $C_{24}H_{23}NO_6$ 422.1598, found 422.1604.

ethyl (E)-3-(7-((diethylcarbamoyl)oxy)-4-oxo-2-phenyl-4H-chromen-5-yl)acrylate (11b)

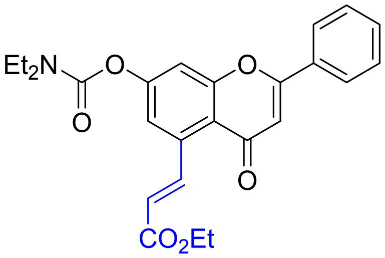

Following General Procedure 2.7, the compound was prepared from 4-oxo-2-phenyl-4H-chromen-7-yl diethylcarbamate (**10a**) (33.7 mg, 0.1 mmol, 1 equiv) and ethyl acrylate (**8b**) (30.0 mg, 0.3 mmol, 3.0 equiv) in DME (1 mL) and it was purified with columm chromatography on silica gel (AcOEt:Hex = 2:8) as white solid, 33 mg, 75% yield. MP: 116-118 ºC. ^{1}H NMR (400 MHz, $CDCl_3$) δ 9.01 (d, *J* = 15.9 Hz, 1H), 7.92 – 7.86 (m, 2H), 7.53 (m, 4H), 7.25 (d, *J* = 2.3 Hz, 1H), 6.77 (s, 1H), 6.28 (d, *J* = 15.9 Hz, 1H), 4.29 (q, 2H), 3.48 (q, 2H), 3.43 (q, 2H), 1.36 (t, 3H), 1.30 (t, 3H), 1.25 (t, 3H). ^{13}C{^{1}H} NMR (126 MHz, $CDCl_3$) δ 179.0, 166.4, 162.3, 157.9, 154.6, 152.8, 143.9, 138.2, 131.7, 131.2, 129.1, 126.2, 122.2, 118.6, 118.5, 112.0, 108.7, 60.6, 42.6, 42.2, 14.3, 13.3. HRMS (ESI) m/z $[M+H]^+$ calcd. for $C_{25}H_{25}NO_6$ 436.1682, found 436.1762.

n-butyl (E)-3-(7-((diethylcarbamoyl)oxy)-4-oxo-2-phenyl-4H-chromen-5-yl)acrylate (11c)

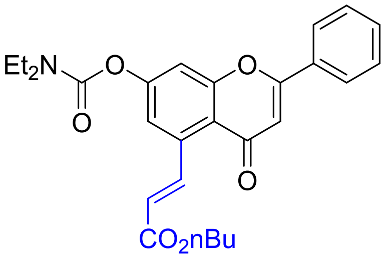

Following General Procedure 2.7, the compound was prepared from 4-oxo-2-phenyl-4H-chromen-7-yl diethylcarbamate (**10a**) (33.7 mg, 0.1 mmol, 1 equiv) and n-butyl acrylate (**8c**) (38.4 mg, 0.3 mmol, 3.0 equiv) in DME (1 mL) and it was purified with columm chromatography on silica gel (AcOEt:Hex = 2:8) as white solid, 43 mg, 92% yield. MP: 109-111 ºC. ^{1}H NMR (500 MHz, $CDCl_3$) δ 9.02 (d, *J* = 15.9 Hz, 1H), 7.92 – 7.89 (m, 2H), 7.53 (m, 4H), 7.26 (d, *J* = 2.3 Hz, 1H), 6.78 (s, 1H), 6.29 (d, *J* = 15.9 Hz, 1H), 4.24

(t, 2H), 3.49 (q, 2H), 3.44 (q, 2H), 1.72 (m, 2H), 1.47 (m, 2H), 1.30 (t, 3H), 1.26 (t, 3H), 0.98 (t, 3H). $^{13}C\{^1H\}$ NMR (126 MHz, $CDCl_3$) δ 179.0, 166.5, 162.3, 157.9, 154.6, 152.8, 143.9, 138.3, 131.7, 131.2, 129.1, 126.2, 122.2, 118.7, 118.6, 112.0, 108.7, 64.5, 42.6, 42.2, 30.8, 19.2, 14.3, 13.8, 13.3. HRMS (ESI) m/z $[M+H]^+$ calcd. for $C_{27}H_{29}NO_6$ 464.1995, found 464.2073.

(E)-4-oxo-2-phenyl-5-styryl-4H-chromen-7-yl diethylcarbamate (11e)

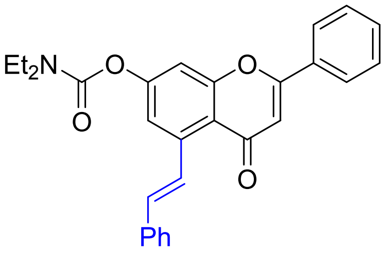

Following General Procedure 2.7, the compound was prepared from 4-oxo-2-phenyl-4H-chromen-7-yl diethylcarbamate (**10a**) (33.7 mg, 0.1 mmol, 1 equiv) and styrene (**8e**) (31.2 mg, 0.3 mmol, 3.0 equiv) in DME (1 mL) and it was purified with columm chromatography on silica gel (AcOEt:Hex = 2:8) as yellow solid, 27 mg, 61% yield. MP: 150-152 ºC. ^1H NMR (400 MHz, $CDCl_3$) δ 8.73 (d, J = 16.2 Hz, 1H), 7.92 – 7.89 (m, 2H), 7.64 – 7.61 (m, 2H), 7.55 – 7.49 (m, 4H), 7.38 – 7.35 (m, 2H), 7.29 (m, 1H), 7.26 (s, 1H), 7.04 (d, J = 16.2 Hz, 1H), 6.75 (s, 1H), 3.50 (q, 2H), 3.45 (q, 2H), 1.31 (t, 3H), 1.26 (t, 3H). $^{13}C\{^1H\}$ NMR (101 MHz, $CDCl_3$) δ 179.8, 161.7, 158.2, 154.5, 153.1, 141.4, 137.3, 132.5, 131.5, 131.3, 129.0, 128.6, 128.0, 127.8, 127.2, 126.1, 118.1, 116.9, 110.0, 108.8, 42.5, 42.1, 14.3, 13.3. HRMS (ESI) m/z $[M+H]^+$ calcd. for $C_{28}H_{25}NO_4$ 440.1784, found 440.1861.

***(*E)-4-oxo-5-(3-oxobut-1-en-1-yl)-2-phenyl-4H-chromen-7-yl diethylcarbamate (11f)**

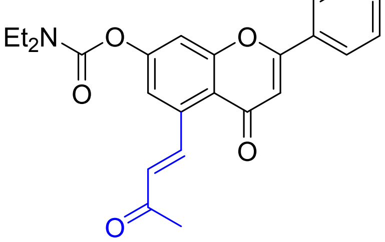

Following General Procedure 2.7, the compound was prepared from 4-oxo-2-phenyl-4H-chromen-7-yl diethylcarbamate (**10a**) (33.7 mg, 0.1 mmol, 1 equiv) and methyl vinyl ketone (**8f**) (21.0 mg, 0.3 mmol, 3.0 equiv) in DME (1 mL) and it was purified with

columm chromatography on silica gel (AcOEt:Hex = 2:8) as brown solid, 27.5 mg, 68% yield. MP: 155-157 ºC. ^{1}H NMR (500 MHz, $CDCl_3$) δ 9.01 (d, *J* = 16.4 Hz, 1H), 7.92 – 7.90 (m, 2H), 7.54 (m, 4H), 7.29 – 7.28 (m, 1H), 6.78 (s, 1H), 6.49 (d, *J* = 16.4 Hz, 1H), 3.49 (q, 2H), 3.44 (q, 2H), 2.50 (s, 3H), 1.31 (t, 3H), 1.25 (t, 3H). ^{13}C{^{1}H} NMR (126 MHz, $CDCl_3$) δ 199.5, 179.3, 162.50 158.0, 154.7, 152.8, 143.5, 138.2, 131.8, 131.4, 131.0, 129.1, 126.2, 118.5, 118.4, 112.3, 108.6, 42.6, 42.2, 26.4, 14.3, 13.3. HRMS (ESI) m/z $[M+H]^+$ calcd. for $C_{24}H_{23}NO_5$ 406.1576, found 406.1653.

(E)-4-oxo-2-phenyl-5-(2-(phenylsulfonyl)vinyl)-4H-chromen-7-yl diethylcarbamate (11g)

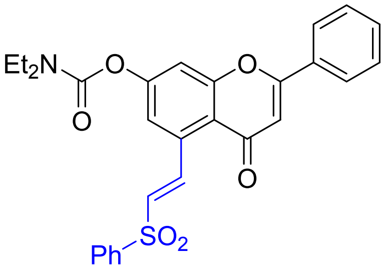

Following General Procedure 2.7, the compound was prepared from 4-oxo-2-phenyl-4H-chromen-7-yl diethylcarbamate (**10a**) (33.7 mg, 0.1 mmol, 1 equiv) and phenyl vinyl sulfone (**8g**) (50.4 mg, 0.3 mmol, 3.0 equiv) in DME (1 mL) and it was purified with columm chromatography on silica gel (AcOEt:Hex = 2:8) as white solid, 17 mg, 33% yield. MP: 190-192 ºC. ^{1}H NMR (500 MHz, $CDCl_3$) δ 8.91 (d, *J* = 15.3 Hz, 1H), 8.03 – 8.00 (m, 2H), 7.83 – 7.78 (m, 2H), 7.54 – 7.45 (m, 7H), 7.10 (d, *J* = 2.2 Hz, 1H), 6.69 (s, 1H), 6.64 (d, *J* = 15.3 Hz, 1H), 3.39 (q, 2H), 3.34 (q, 2H), 1.22 (t, 3H), 1.17 (t, 3H). ^{13}C{^{1}H} NMR (126 MHz, $CDCl_3$) δ 178.6, 162.6, 157.7, 154.6, 152.6, 143.2, 140.4, 135.8, 133.4, 131.8, 131.0, 130.6, 129.3, 129.1, 128.1, 126.2, 119.0, 118.8, 112.7, 108.5, 42.6, 42.2, 14.3, 13.3. HRMS (ESI) m/z $[M+H]^+$ calcd. for $C_{28}H_{25}NO_6S$ 504.1403, found 504.1484.

methyl (E)-3-(7-((diethylcarbamoyl)oxy)-2-(4-fluorophenyl)-4-oxo-4H-chromen-5-yl)acrylate (11h)

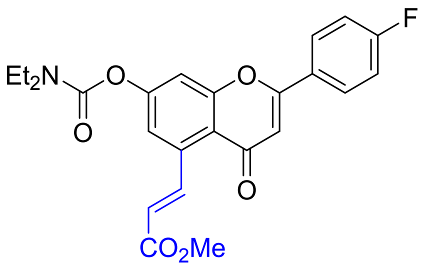

Following General Procedure 2.7, the compound was prepared from 2-(4-fluorophenyl)-4-oxo-4H-chromen-7-yl diethylcarbamate (**10b**) (35.5 mg, 0.1 mmol, 1 equiv) and methyl acrylate (**8a**) (25.8 mg, 0.3 mmol, 3.0 equiv) in DME (1 mL) and it was purified with column chromatography on silica gel (AcOEt:Hex = 2:8) as white solid, 26 mg, 60% yield. MP: 160-162 ºC. ^{1}H NMR (500 MHz, $CDCl_3$) δ 9.00 (d, *J* = 15.9 Hz, 1H), 7.92 – 7.89 (m, 2H), 7.52 (d, *J* = 2.3 Hz, 1H), 7.27 – 7.23 (d, *J* = 2.3 Hz, 1H), 7.23 – 7.19 (m, 2H), 6.71 (s, 1H), 6.29 (d, *J* = 15.9 Hz, 1H), 3.84 (s, 3H), 3.49 (q, 2H), 3.43 (q, 2H), 1.30 (t, 3H), 1.26 (t, 3H). ^{13}C{^{1}H} NMR (126 MHz, $CDCl_3$) δ 178.8, 166.8, 161.3, 157.7, 154.6, 152.7, 144.1, 138.1, 128.5, 128.4, 121.8, 118.6, 118.5, 116.4, 116.2, 112.0, 108.4, 51.8, 42.6, 42.2, 14.3, 13.3. HRMS (ESI) m/z $[M+H]^+$ calcd. for $C_{24}H_{22}FNO_6$ 440.1431, found 440.1508.

ethyl (E)-3-(7-((diethylcarbamoyl)oxy)-2-(4-fluorophenyl)-4-oxo-4H-chromen-5-yl)acrylate (11i)

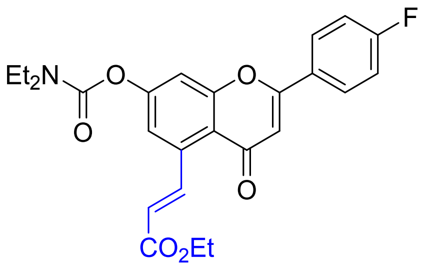

Following General Procedure 2.7, the compound was prepared from from 2-(4-fluorophenyl)-4-oxo-4H-chromen-7-yl diethylcarbamate (**10b**) (35.5 mg, 0.1 mmol, 1 equiv) and ethyl acrylate (**8b**) (30.0 mg, 0.3 mmol, 3.0 equiv) in DME (1 mL) and it was purified with column chromatography on silica gel (AcOEt:Hex = 2:8) as white solid, 20 mg, 44% yield. MP: 149-151 ºC. ^{1}H NMR (400 MHz, $CDCl_3$) δ 8.99 (d, *J* = 15.9 Hz, 1H), 7.93 – 7.87 (m, 2H), 7.51 (d, *J* = 2.2 Hz, 1H), 7.24 (d, *J* = 2.2 Hz, 1H), 7.20 (m, 2H), 6.71 (s, 1H), 6.28 (d, *J* = 15.9 Hz, 1H), 4.29 (q, 2H), 3.53 – 3.46 (q, 2H), 3.43 (q, 2H), 1.36 (t, 3H), 1.30 (t, 3H), 1.24 (t, 3H). ^{13}C{^{1}H} NMR (101 MHz, $CDCl_3$) δ 178.8, 166.3, 166.1, 163.5, 161.3, 157.7, 154.6, 152.7, 143.8, 138.2, 128.44, 128.35, 127.3, 122.3,

118.6, 118.5, 116.4, 116.2, 111.9, 108.4, 60.6, 42.6, 42.1, 14.3, 13.3. HRMS (ESI) m/z $[M+H]^+$ calcd. for $C_{25}H_{24}FNO_6$ 454.1588, found 454.1663.

methyl (E)-3-(7-((diethylcarbamoyl)oxy)-4-oxo-2-(4-(trifluoromethyl)phenyl)-4H-chromen-5-yl)acrylate (11j)

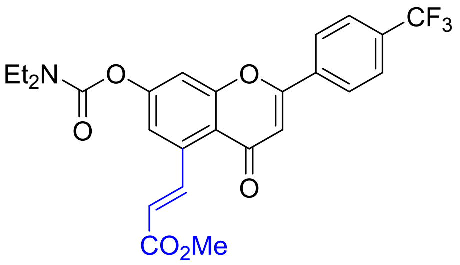

Following General Procedure 2.7, the compound was prepared from 4-oxo-2-(4-(trifluoromethyl)phenyl)-4H-chromen-7-yl diethylcarbamate (**10c**) (40.5 mg, 0.1 mmol, 1 equiv) and methyl acrylate (**8a**) (25.8 mg, 0.3 mmol, 3.0 equiv) in DME (1 mL) and it was purified with columm chromatography on silica gel (AcOEt:Hex = 3:7) as white solid, 16 mg, 32% yield. MP: 197-199 ºC. ^{1}H NMR (500 MHz, $CDCl_3$) δ 8.98 (d, J = 15.9 Hz, 1H), 8.02 (d, J = 8.2 Hz, 2H), 7.79 (d, J = 8.2 Hz, 2H), 7.55 (d, J = 2.2 Hz, 1H), 7.26 (d, J = 2.6 Hz, 1H), 6.81 (s, 1H), 6.28 (d, J = 15.9 Hz, 1H), 3.84 (s, 3H), 3.48 (q, 2H), 3.43 (q, 2H), 1.30 (t, 3H), 1.26 (t, 3H). $^{13}C\{^1H\}$ NMR (126 MHz, $CDCl_3$) δ 178.7, 166.7, 160.5, 157.8, 154.8, 152.7, 143.9, 138.3, 134.6, 126.6, 126.1, 124.7, 122.5, 122.0, 118.8, 118.6, 112.1, 109.9, 51.9, 42.6, 42.2, 14.3, 13.3. HRMS (ESI) m/z $[M + H]^+$ calcd for $C_{25}H_{22}F_3NO_6$ 490.1399, found 490.1476.

ethyl (E)-3-(7-((diethylcarbamoyl)oxy)-4-oxo-2-(4-(trifluoromethyl)phenyl)-4H-chromen-5-yl)acrylate (11k)

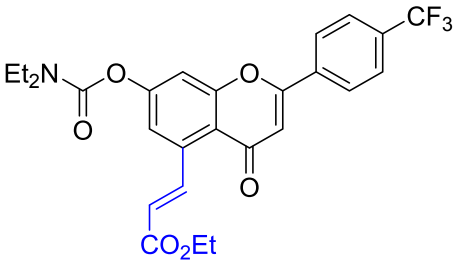

Following General Procedure 2.7, the compound was prepared from 4-oxo-2-(4-(trifluoromethyl)phenyl)-4H-chromen-7-yl diethylcarbamate (**10c**) (40.5 mg, 0.1 mmol, 1 equiv) and ethyl acrylate (**8b**) (30.0 mg, 0.3 mmol, 3.0 equiv) in DME (1 mL) and it was purified with columm chromatography on silica gel (AcOEt:Hex = 2:8) as white

solid, 28 mg, 55% yield. MP: 190-192 ºC. ^{1}H NMR (500 MHz, $CDCl_3$) δ 8.98 (d, *J* = 15.9 Hz, 1H), 8.02 (d, *J* = 8.2 Hz, 2H), 7.79 (d, *J* = 8.2 Hz, 2H), 7.55 (d, *J* = 2.2 Hz, 1H), 7.27 (d, *J* = 2.2 Hz, 1H), 6.82 (s, 1H), 6.30 (d, *J* = 15.9 Hz, 1H), 4.30 (q, 2H), 3.49 (q, 2H), 3.44 (q, 2H), 1.36 (t, 3H), 1.31 (t, 3H), 1.26 (t, 3H). $^{13}C\{^1H\}$ NMR (126 MHz, $CDCl_3$) δ 178.7, 166.3, 160.5, 157.8, 154.8, 152.7, 143.7, 138.3, 134.6, 133.4, 126.5, 126.11, 126.08, 126.05, 122.5, 118.8, 118.6, 112.0, 109.8, 60.7, 42.6, 42.2, 14.3, 13.3. HRMS (ESI) m/z $[M + H]^+$ calcd for $C_{26}H_{24}F_3NO_6$ 504.1556, found 504.1633.

methyl (E)-3-(7-((diethylcarbamoyl)oxy)-2-(4-methoxyphenyl)-4-oxo-4H-chromen-5-yl)acrylate (11l)

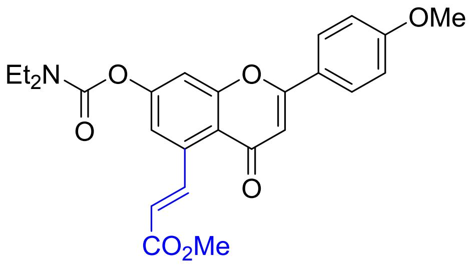

Following General Procedure 2.7, the compound was prepared from 2-(4-methoxyphenyl)-4-oxo-4H-chromen-7-yl diethylcarbamate (**10d**) (36.7 mg, 0.1 mmol, 1 equiv) and methyl acrylate (**8a**) (25.8 mg, 0.3 mmol, 3.0 equiv) in DME (1 mL) and it was purified with columm chromatography on silica gel (AcOEt:Hex = 2:8) as white solid, 21.5 mg, 47% yield. MP: 158-160 ºC. ^{1}H NMR (400 MHz, $CDCl_3$) δ 9.03 (d, *J* = 16.2 Hz, 1H), 7.86 – 7.82 (m, 2H), 7.50 (d, *J* = 2.2 Hz, 1H), 7.22 (d, *J* = 2.3 Hz, 1H), 7.03 – 6.99 (m, 2H), 6.67 (s, 1H), 6.28 (d, *J* = 16.2 Hz, 1H), 3.89 (s, 3H), 3.83 (s, 3H), 3.49 (q, 2H), 3.42 (q, 2H), 1.30 (t, 3H), 1.25 (t, 3H). $^{13}C\{^1H\}$ NMR (101 MHz, $CDCl_3$) δ 178.9, 166.8, 162.5, 162.3, 157.7, 154.4, 152.8, 144.35, 144.33, 138.0, 129.5, 127.9, 123.3, 121.5, 118.6, 118.4, 114.5, 112.0, 107.2, 55.5, 51.8, 42.5, 42.1, 14.3, 13.3. HRMS (ESI) m/z $[M + H]^+$ calcd for $C_{25}H_{25}NO_7$ 452.1631, found 452.1712.

ethyl (E)-3-(7-((diethylcarbamoyl)oxy)-2-(4-methoxyphenyl)-4-oxo-4H-chromen-5-yl)acrylate (11m)

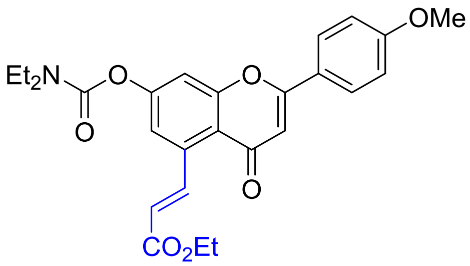

Following General Procedure 2.7, the compound was prepared from 2-(4-methoxyphenyl)-4-oxo-4H-chromen-7-yl diethylcarbamate (**10d**) (36.7 mg, 0.1 mmol, 1 equiv) and ethyl acrylate (**8b**) (30.0 mg, 0.3 mmol, 3.0 equiv) in DME (1 mL) and it was purified with columm chromatography on silica gel (AcOEt:Hex = 2:8) as yellow solid, 27 mg, 59% yield. MP: 152-154 ºC. ^{1}H NMR (500 MHz, $CDCl_3$) δ 9.02 (d, J = 15.9 Hz, 1H), 7.86 – 7.82 (m, 2H), 7.49 (d, J = 2.3 Hz, 1H), 7.23 (d, J = 2.3 Hz, 1H), 7.03 – 6.99 (m, 2H), 6.67 (s, 1H), 6.27 (d, J = 15.9 Hz, 1H), 4.29 (q, 2H), 3.89 (s, 3H), 3.48 (q, 2H), 3.43 (q, 2H), 1.36 (t, 3H), 1.30 (t, 3H), 1.25 (t, 3H). $^{13}C\{^1H\}$ NMR (126 MHz, $CDCl_3$) δ 178.9, 166.4, 162.5, 162.3, 157.7, 154.4, 152.8, 144.1, 138.1, 129.5, 127.9, 123.3, 122.0, 118.6, 118.4, 114.5, 112.0, 107.2, 60.6, 55.5, 42.6, 42.1, 14.3, 13.3. HRMS (ESI) m/z [M + H]$^+$ calcd for $C_{26}H_{27}NO_7$ 466.1788, found 466.1868.

methyl (E)-3-(7-((diethylcarbamoyl)oxy)-2-(3,4-dimethoxyphenyl)-4-oxo-4H-chromen-5-yl)acrylate (11n)

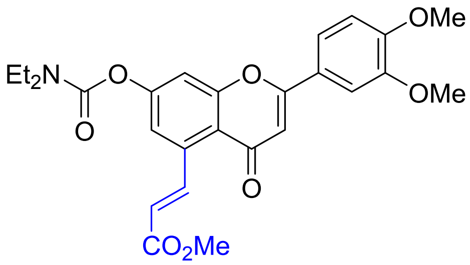

Following General Procedure 2.7, the compound was prepared from 2-(3,4-dimethoxyphenyl)-4-oxo-4H-chromen-7-yl diethylcarbamate (**10e**) (39.7 mg, 0.1 mmol, 1 equiv) and methyl acrylate (**8a**) (25.8 mg, 0.3 mmol, 3.0 equiv) in DME (1 mL) and it was purified with columm chromatography on silica gel (AcOEt:Hex = 4:6) as yellow solid, 33 mg, 68% yield. MP: 179-181 ºC. ^{1}H NMR (400 MHz, $CDCl_3$) δ 9.03 (d, J = 15.9 Hz, 1H), 7.52 (dd, J = 8.3, 2.2 Hz, 2H), 7.35 (d, J = 2.2 Hz, 1H), 7.22 (d, J = 2.3 Hz, 1H), 6.97 (d, J = 8.5 Hz, 1H), 6.68 (s, 1H), 6.28 (d, J = 15.9 Hz, 1H), 3.98 (s, 3H), 3.96 (s, 3H), 3.83 (s, 3H), 3.51 – 3.46 (q, 2H), 3.46 – 3.41 (q, 2H), 1.31 (t, 3H), 1.26 (t, 3H). $^{13}C\{^1H\}$ NMR (101 MHz, $CDCl_3$) δ 177.8, 165.8, 161.2, 156.7, 153.4, 151.8, 151.1,

148.3, 143.3, 137.0, 122.5, 120.6, 118.9, 117.6, 117.4, 111.1, 110.1, 107.6, 106.5, 55.12, 55.07, 50.8, 41.5, 41.1, 13.23, 12.3. HRMS (ESI) m/z $[M + H]^+$ calcd for $C_{26}H_{27}NO_8$ 482.1737, found 482.1818.

ethyl (E)-3-(7-((diethylcarbamoyl)oxy)-2-(3,4-dimethoxyphenyl)-4-oxo-4H-chromen-5-yl)acrylate (11o)

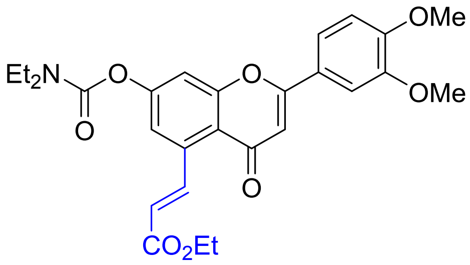

Following General Procedure 2.7, the compound was prepared from 2-(3,4-dimethoxyphenyl)-4-oxo-4H-chromen-7-yl diethylcarbamate (**10e**) (39.7 mg, 0.1 mmol, 1 equiv) and ethyl acrylate (**8b**) (30.0 mg, 0.3 mmol, 3.0 equiv) in DME (1 mL) and it was purified with columm chromatography on silica gel (AcOEt:Hex = 4:6) as yellow solid, 37 mg, 75% yield. MP: 157-159 ºC. ^1H NMR (500 MHz, $CDCl_3$) δ 9.02 (d, J = 15.9 Hz, 1H), 7.54 – 7.48 (m, 2H), 7.34 (d, J = 2.3 Hz, 1H), 7.23 (d, J = 2.3 Hz, 1H), 6.97 (d, J = 8.5 Hz, 1H), 6.68 (s, 1H), 6.28 (d, J = 15.9 Hz, 1H), 4.29 (q, 2H), 3.98 (s, 3H), 3.96 (s, 3H), 3.48 (q, 2H), 3.43 (q, 2H), 1.36 (t, 3H), 1.30 (t, 3H), 1.26 (t, 3H). $^{13}C\{^1H\}$ NMR (101 MHz, $CDCl_3$) δ 177.8, 165.4, 161.2, 156.7, 153.4, 151.8, 151.1, 148.3, 143.00, 142.99, 137.1, 122.5, 121.0, 118.9, 117.6, 117.4, 111.0, 110.1, 107.6, 106.4, 59.6, 55.09, 55.06, 41.5, 41.1, 13.3, 12.3. HRMS (ESI) m/z $[M + H]^+$ calcd for $C_{27}H_{29}NO_8$ 496.1893, found 496.1979.

3. Computacional Methods

All structures were optimized using density functional theory (DFT) as implemented in Gaussian 16,[6] with B3LYP[7] as functional, 6-31G(d,p) as basis set for non-metallic atoms, and LANL2DZ[8] as basis set for ruthenium. Final energies were obtained performing single-point calculations on the previously optimized structures at M06[9]/6-311++G(d,p) level of theory for non-metallic atoms and SDD basis set for ruthenium,[10] introducing solvation factors with the IEF-PCM[11] method, and 1,4-dioxane as solvent. The stationary

points were characterized by frequency calculations in order to verify that they have the right number of imaginary frequencies.

Cartesian coordinates of the optimized structures are shown below, as well as their single point energy and correction to Gibbs free energy (in kcal/mol).

Table S1. *Single point energy and correction to Gibbs free energy (in kcal/mol) for calculated complexes*

Complex	G_{Corr} (kcal/mol)	E_{SP} (kcal/mol)	Frecuency (cm^{-1})
A_{Ket}	304.977	-1106083.813	
A_{Carb}	306.513	-1106080.505	
TS $A\text{-}B_{Carb}$	304.927	-1106061.111	-752.54
B_{Carb}	308.856	-1106075.264	
C_{Carb}	330.385	-1154604.159	
TS $C\text{-}D_{Carb}$	327.571	-1154590.459	-291.72
D_{Carb}	329.500	-1154611.825	
TS $A\text{-}B_{Ket}$	305.106	-1106061.819	-574.40
B_{Ket}	307.456	-1106081.523	
C_{Ket}	328.826	-1154613.992	
TS $C\text{-}D_{Ket}$	329.144	-1154598.072	-283.42
D_{Ket}	329.0855	-1154611.549	
TS C-H Activation C6	305.165	-1106058.363	-890.91
Methyl Acrylate	40.813	-192244.762	
AcOH	22.504	-143718.513	

Cartesian Coordinates for Calculates Species

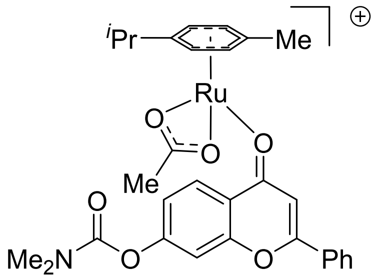

$A_{Ket.}$

C	4.10539	-1.20761	0.13446
C	3.08740	-2.14786	0.41464
C	3.79288	0.11994	-0.14801
C	2.44688	0.48534	-0.12903
C	1.76454	-1.76062	0.41598
C	1.41010	-0.42276	0.14246

O	2.18074	1.79997	-0.40117
C	0.91669	2.27478	-0.38264
C	0.03882	0.05626	0.13701
C	-0.14724	1.44724	-0.11902
H	0.97540	-2.47006	0.63579
H	4.55721	0.85508	-0.34754
H	-1.14418	1.85590	-0.04394
O	-0.90336	-0.77197	0.36053
C	0.84415	3.71596	-0.65378
C	2.01131	4.50206	-0.62056
C	1.94302	5.87111	-0.86251
C	0.71617	6.47519	-1.14501
C	-0.38664	4.33376	-0.94509
C	-0.44734	5.70182	-1.18730
H	2.84918	6.46744	-0.82830
H	0.66593	7.54293	-1.33455
H	-1.29571	3.74483	-1.00131
H	-1.40157	6.16604	-1.41541
H	2.96469	4.03880	-0.39660
H	-4.05566	1.14032	-1.84713
H	-6.83455	1.13481	-0.58046
H	-5.46039	1.97844	0.15175
C	-4.13931	0.14881	-1.41490
C	-5.99251	1.02453	0.11269
H	-2.51106	-0.64293	-2.58492
C	-5.08873	-0.08053	-0.35710
C	-3.26363	-0.86926	-1.83697
H	-6.39643	0.81324	1.10492
H	-2.99276	-3.08890	-3.79771
H	-2.50007	0.39198	4.76275
C	-5.09708	-1.36003	0.24346
C	-3.27911	-2.17744	-1.23585
O	-2.75826	-0.79625	2.48647
Ru	-3.01272	-0.57521	0.37729
C	-2.80700	-3.84790	-3.03134
C	-2.59908	0.45909	2.63028
H	-2.73544	2.03086	4.06928
H	-1.35938	-2.68932	-1.93561
C	-2.23533	1.06762	3.94848
C	-4.20052	-2.39341	-0.18898
O	-2.74090	1.17538	1.57582
C	-2.29115	-3.22594	-1.71503
H	-5.74203	-1.54453	1.09556
H	-3.74023	-4.39621	-2.86521
H	-2.06858	-4.55116	-3.42712
H	-4.18849	-3.32926	0.35609
H	-1.15437	1.24321	3.97544
C	-1.97226	-4.30743	-0.67473
H	-1.63505	-3.87154	0.27046
H	-1.17664	-4.95526	-1.05301
H	-2.83662	-4.94965	-0.47382
H	3.37554	-3.17054	0.63050
H	8.26108	-3.36628	-1.02283
H	6.63015	-3.52576	-0.33975
C	7.63536	-3.16254	-0.14688
H	8.05530	-3.70543	0.70852
O	5.36405	-1.74151	0.11641
N	7.62392	-1.72786	0.11962

C	6.51656	-0.96426	0.27428	O	6.48769	0.22540	0.52323
H	9.45305	-1.55080	1.15091	H	9.53860	-1.23490	-0.59795
C	8.92867	-1.09764	0.30168	H	8.78942	-0.03501	0.48856

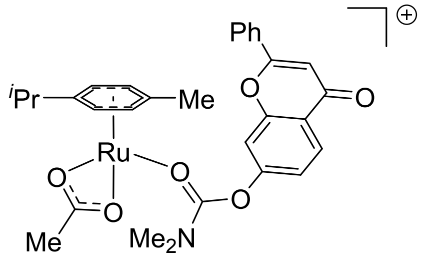

A$_{Carb.}$

C	0.28844	1.84644	2.39821	H	2.48257	-0.91840	4.57372
C	0.24716	0.45149	2.27445	H	4.22509	-0.57930	4.70445
C	-0.82343	2.57840	2.00747	H	3.66545	-2.21905	4.30487
C	-1.96703	1.93665	1.50541	H	1.16795	2.32968	2.80992
C	-0.86199	-0.22493	1.79654	H	-0.85272	3.66035	2.08801
C	-1.97300	0.53889	1.41647	H	-0.86486	-1.30423	1.70746
C	-3.16411	2.70994	1.08383	H	-8.14064	-0.41467	-1.68877
C	-4.27499	1.87748	0.64475	C	-7.32965	-0.78915	-1.07205
O	-3.04913	-0.14922	0.93271	C	-6.31434	0.07446	-0.67201
C	-4.19269	0.52298	0.57109	H	-6.33617	1.10971	-0.99572
O	-3.18999	3.93792	1.11443	C	-7.30164	-2.13280	-0.68948
O	1.32536	-0.31352	2.74170	H	-8.09563	-2.80409	-1.00192
C	2.48911	-0.30900	2.05647	C	-5.25392	-0.39399	0.12451
O	2.59921	0.14257	0.89353	C	-6.24819	-2.60927	0.09316
N	3.51950	-0.81705	2.74119	C	-5.22866	-1.74996	0.49628
C	4.82773	-0.94315	2.09055	H	-6.22317	-3.65152	0.39616
H	4.69912	-1.02123	1.01304	H	-4.41853	-2.12160	1.11299
H	5.31442	-1.84711	2.46437	H	-5.19849	2.37839	0.38472
H	5.46077	-0.08090	2.32692	H	0.56650	-2.70558	-3.71438
C	3.46266	-1.15246	4.16755	H	-0.79074	-1.69399	-4.22916

C	-0.15009	-1.95284	-3.37812	H	3.55542	-4.35284	0.53695
H	2.45366	-1.13180	-3.81400	H	2.12589	-4.92217	-0.34164
H	-0.78220	-2.39341	-2.60316	C	1.89119	1.64709	-1.86000
C	1.89011	-0.42129	-3.21836	C	0.54076	1.33325	-1.47913
C	0.54599	-0.71953	-2.87475	H	4.56049	2.76722	-2.15245
H	1.93720	-4.35370	1.32116	H	2.49042	4.04817	-3.13226
C	2.55163	0.75332	-2.73159	H	4.38828	1.90764	-0.60372
H	3.59626	0.90235	-2.97430	C	4.07891	2.79137	-1.16900
Ru	1.77449	-0.48828	-1.02189	C	2.55145	2.88284	-1.27786
C	2.48627	-4.19209	0.39147	H	0.04216	1.94846	-0.73866
C	-0.11220	0.18610	-1.97420	C	2.11632	4.11400	-2.10525
C	2.22976	-2.81237	-0.13290	H	1.02766	4.21628	-2.14515
O	1.09311	-2.24141	0.01477	H	4.46034	3.67540	-0.65071
O	3.12023	-2.15540	-0.76751	H	2.14658	2.99734	-0.26484
H	-1.09983	-0.06466	-1.60155	H	2.52310	5.02424	-1.65566

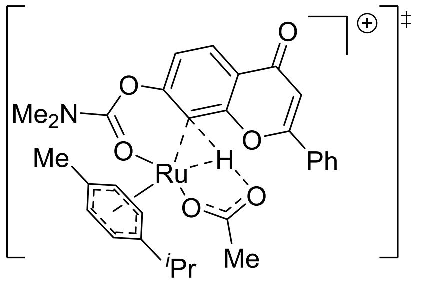

TS A-B$_{Carb.}$

C	-1.73864	-3.45239	-0.39615	O	1.65419	-1.20665	0.40813
C	-1.81890	-2.13696	0.07329	C	2.92204	-1.72983	0.27558
C	-0.48492	-4.00446	-0.60665	O	2.16935	-5.01999	-0.95992
C	0.68344	-3.27108	-0.33909	O	-3.12012	-1.74872	0.36962
C	-0.70071	-1.31576	0.31586	C	-3.52838	-0.48415	0.61149
C	0.55397	-1.95925	0.11992	O	-2.91826	0.54074	0.23683
C	2.02516	-3.87894	-0.53113	N	-4.70616	-0.42525	1.24485
C	3.12535	-2.99805	-0.16113	C	-5.34536	0.87042	1.48317

H	-5.54133	0.98718	2.55319
H	-6.29743	0.91897	0.94432
H	-4.69314	1.67106	1.14373
C	-5.46000	-1.60268	1.68495
H	-0.36414	-5.02294	-0.96250
H	-0.79547	-0.67926	1.42152
H	7.27335	-0.10862	0.18277
C	6.26223	0.01016	0.55918
C	5.27662	-0.89693	0.18085
H	5.52644	-1.70359	-0.50025
C	5.95129	1.07260	1.41185
H	6.72269	1.77721	1.70685
C	3.96013	-0.75730	0.65597
C	4.64538	1.22472	1.88204
C	3.65368	0.32032	1.50663
H	4.40054	2.04408	2.55113
H	2.64418	0.43093	1.88561
H	4.12938	-3.39697	-0.22719
H	-0.86069	-1.92341	-3.05593
H	-0.98726	-0.96362	-4.53498
C	-1.29744	-1.01855	-3.48498
H	1.12151	-0.55225	-2.23302
H	-2.38574	-1.11525	-3.46520
C	0.46842	0.31209	-2.19246
C	-0.84259	0.21454	-2.75352
H	0.32358	1.66678	4.25647
C	0.93261	1.48818	-1.54999

H	1.91877	1.48785	-1.10470
Ru	-0.93176	0.77303	-0.51932
C	-0.58951	1.95738	3.72763
C	-1.69120	1.33186	-2.55492
C	-0.73104	1.10707	2.48685
O	-0.61648	1.68813	1.35610
O	-0.93774	-0.13326	2.62640
H	-2.73188	1.26352	-2.85644
H	-1.42741	1.76001	4.40095
H	-0.54741	3.01673	3.47611
C	0.09307	2.64172	-1.44433
C	-1.23339	2.53226	-1.93203
H	2.64838	3.49220	-0.34481
H	1.72680	4.60144	-2.53227
H	1.50523	3.05885	0.94942
C	1.72772	3.78930	0.16874
C	0.55109	3.93908	-0.80393
H	-1.92661	3.35329	-1.78591
C	0.88307	4.94963	-1.92722
H	0.03205	5.11633	-2.59429
H	1.92490	4.75263	0.64745
H	-0.30710	4.32744	-0.24182
H	1.16015	5.91152	-1.48649
H	-2.64721	-4.01997	-0.56372
H	-4.85130	-2.49884	1.60865
H	-6.36147	-1.72187	1.07443
H	-5.75855	-1.46002	2.72724

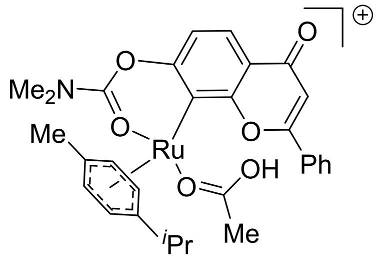

B$_{Carb.}$

C	-1.72919	-3.53478	-0.38209	C	6.27231	0.01516	-0.27460
C	-1.79941	-2.13778	-0.27403	C	5.27119	-0.94051	-0.43273
C	-0.48580	-4.13935	-0.40653	H	5.48434	-1.85777	-0.97177
C	0.67741	-3.36205	-0.30773	C	6.00289	1.21352	0.39023
C	-0.68759	-1.29412	-0.19858	H	6.78547	1.95578	0.51341
C	0.55600	-1.97078	-0.19715	C	3.98189	-0.71126	0.07643
C	2.01762	-4.00671	-0.31274	C	4.72284	1.45251	0.89568
C	3.12467	-3.07080	-0.16852	C	3.71612	0.50118	0.73829
O	1.67420	-1.17721	-0.08536	H	4.51235	2.37753	1.42478
C	2.92869	-1.73266	-0.06976	H	2.72709	0.68244	1.14511
O	2.15612	-5.22182	-0.41901	H	4.12598	-3.47920	-0.11676
O	-3.12816	-1.65469	-0.30817	H	-0.64327	-1.23909	-3.26939
C	-3.51065	-0.58250	0.40249	H	-1.11946	-0.03934	-4.47956
O	-2.71431	0.30079	0.80652	C	-1.27402	-0.36487	-3.44424
N	-4.82818	-0.51835	0.63493	H	1.18442	0.25363	-2.29104
C	-5.39705	0.64802	1.31296	H	-2.31932	-0.66569	-3.34037
H	-5.73689	0.37158	2.31661	C	0.40835	0.96580	-2.03884
H	-6.25505	1.01408	0.74129	C	-0.93103	0.75921	-2.50676
H	-4.64555	1.43002	1.39215	H	-0.50219	0.58925	4.86854
C	-5.76734	-1.59700	0.31560	C	0.70864	2.03752	-1.17055
H	-0.37006	-5.21461	-0.48930	H	1.71360	2.10642	-0.77192
H	-0.82528	-1.21657	1.85503	Ru	-0.89981	0.76572	-0.24901
H	7.26227	-0.17342	-0.67791	C	0.29099	0.75524	4.13450

C	-1.91424	1.65737	-2.01926	C	1.16539	4.12966	0.99022
C	-0.16011	0.27531	2.78822	C	0.11750	4.33434	-0.11488
O	-0.09414	0.98788	1.77494	H	-2.35606	3.53313	-0.99324
O	-0.61279	-0.96103	2.78165	C	0.59036	5.39260	-1.13739
H	-2.95455	1.48410	-2.27700	H	-0.17403	5.59528	-1.89334
H	0.55431	1.81072	4.09291	H	1.28791	5.05962	1.55272
H	1.15709	0.16753	4.45512	H	-0.80061	4.70942	0.35440
C	-0.25903	3.06571	-0.85952	H	0.81765	6.33143	-0.62368
C	-1.57065	2.83988	-1.27657	H	-2.64513	-4.11214	-0.44581
H	2.14668	3.87428	0.57554	H	-5.24157	-2.44479	-0.11363
H	1.49928	5.06340	-1.65250	H	-6.51388	-1.23612	-0.39896
H	0.86711	3.33989	1.68373	H	-6.28126	-1.91242	1.22911

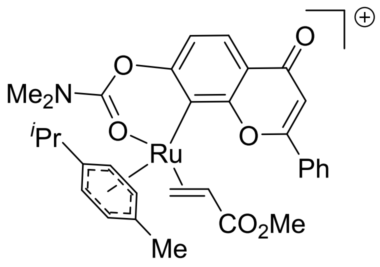

$C_{Carb.}$

C	0.00511	4.22648	-0.19238	O	-2.00412	3.06805	0.07963
C	-0.60101	2.96494	-0.08997	C	-2.85411	2.19247	-0.46053
C	1.37851	4.30115	-0.30722	O	-2.54305	1.00641	-0.74111
C	2.14189	3.12609	-0.31214	N	-4.09217	2.66317	-0.65685
C	0.09093	1.75659	-0.08724	C	-5.15065	1.77388	-1.13893
C	1.49551	1.88722	-0.20387	H	-5.38490	1.99485	-2.18569
C	3.62223	3.20263	-0.43128	H	-6.05057	1.93080	-0.53746
C	4.28874	1.91010	-0.39829	H	-4.82954	0.73823	-1.05555
O	2.23127	0.72261	-0.17718	C	-4.45902	4.07362	-0.50031
C	3.60256	0.74909	-0.25584	H	7.12307	-2.11914	0.70463
O	4.21086	4.27466	-0.54259	C	6.10074	-2.01713	0.35415

C	5.52143	-0.75331	0.28407	C	-3.78420	-3.21543	0.62011
H	6.09266	0.11407	0.59749	H	-3.83413	-2.57487	-0.26482
C	5.36892	-3.14896	-0.01451	H	-2.71893	-5.26144	2.14542
H	5.82435	-4.13307	0.03930	H	-1.76151	-3.87101	0.33418
C	4.19643	-0.59812	-0.16301	H	-4.19080	-4.19349	0.34878
C	4.05048	-3.00740	-0.45021	H	-0.61628	5.11535	-0.17781
C	3.46412	-1.74445	-0.52164	H	-3.59027	4.66350	-0.22317
H	3.47393	-3.87786	-0.74819	H	-5.22417	4.17148	0.27619
H	2.44413	-1.66003	-0.87627	H	-4.86707	4.44865	-1.44412
H	5.36602	1.89783	-0.50164	H	-0.69868	-4.78204	-3.30181
H	1.57966	1.47524	2.65175	O	0.31330	-3.04336	-1.55871
H	0.76497	1.04612	4.16051	C	-0.77051	-2.56975	-1.85166
C	0.60240	1.31856	3.11038	H	-0.34348	0.78843	-2.37127
H	0.04813	2.25909	3.09033	C	-1.52141	-4.68898	-2.59019
C	0.51664	-0.86217	1.78294	C	-0.12469	-0.16761	-1.91391
C	-0.15668	0.20966	2.44084	C	-1.13617	-1.12271	-1.80519
C	-0.21746	-1.94540	1.25374	O	-1.79762	-3.29546	-2.32833
Ru	-0.93974	-0.04284	0.17926	H	0.91153	-0.47917	-1.91324
C	-1.56950	0.19515	2.41626	H	-2.44149	-5.09317	-3.00896
C	-1.63791	-2.07153	1.43846	H	-2.14195	-0.88814	-2.13477
C	-2.29558	-0.94685	1.95409	H	-1.25555	-5.20986	-1.66744
H	-2.84916	-3.84601	3.20116	H	1.90217	5.24667	-0.39479
H	-4.44183	-2.80233	1.39246	H	-3.37774	-0.93028	2.01554
H	-1.25464	-4.44400	2.71037	H	-2.11623	1.03826	2.82535
C	-2.28302	-4.28423	2.37248	H	0.31994	-2.73001	0.73319
C	-2.34254	-3.37763	1.12026	H	1.59372	-0.84913	1.68049

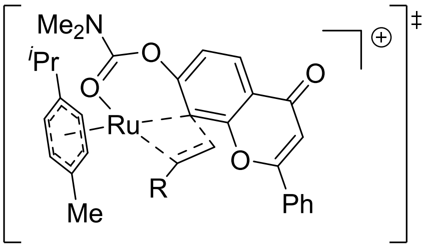

TS C-D$_{Carb.}$

C	-0.32887	4.09846	-0.22095	C	5.58171	-2.82302	-0.60150
C	-0.83328	2.80805	-0.40849	H	6.10706	-3.76777	-0.70297
C	1.02957	4.27312	-0.02964	C	4.22938	-0.37486	-0.34657
C	1.88519	3.16495	-0.05913	C	4.28154	-2.68573	-1.08966
C	-0.05012	1.64103	-0.35910	C	3.60396	-1.47346	-0.96417
C	1.34672	1.88505	-0.23371	H	3.78900	-3.51972	-1.58018
C	3.35438	3.35579	0.07568	H	2.59861	-1.38721	-1.35870
C	4.12545	2.12903	-0.04405	H	5.20391	2.21056	-0.00698
O	2.16892	0.79195	-0.31797	H	1.95276	1.27224	2.44999
C	3.53739	0.92037	-0.22879	H	1.61483	0.56289	4.02722
O	3.84905	4.46407	0.26078	C	1.14780	0.97855	3.12601
O	-2.17994	2.82198	-0.80011	H	0.59074	1.87078	3.42008
C	-3.11037	1.91412	-0.46486	C	0.76485	-1.04554	1.60658
O	-2.85695	0.81159	0.06681	C	0.25126	-0.05899	2.51368
N	-4.36025	2.29208	-0.77080	C	-0.04518	-2.10082	1.12985
C	-5.48059	1.37992	-0.53543	Ru	-0.98882	-0.17677	0.41361
H	-6.08022	1.30251	-1.44727	C	-1.12530	-0.09176	2.77252
H	-6.11558	1.76318	0.27042	C	-1.42586	-2.21508	1.49740
H	-5.10535	0.39678	-0.26220	C	-1.95057	-1.13673	2.23927
C	-4.71037	3.60826	-1.31170	H	-2.35763	-4.28367	3.10750
H	7.21252	-1.84078	0.41488	H	-4.22040	-2.91154	1.88235
C	6.20559	-1.73882	0.02244	H	-0.92512	-4.80186	2.20332
C	5.53695	-0.52567	0.15273	C	-1.98932	-4.56255	2.11451
H	6.02482	0.29910	0.66126	C	-2.24045	-3.43047	1.09278

C	-3.74089	-3.15551	0.92812	C	-1.11566	-4.28703	-2.79278
H	-3.92416	-2.33705	0.22528	C	-0.41779	0.36957	-1.85478
H	-2.51401	-5.47071	1.80352	C	-1.23815	-0.79593	-1.65147
H	-1.85610	-3.76138	0.12198	O	-1.58986	-3.00541	-2.32692
H	-4.23770	-4.04927	0.54106	H	0.61852	0.18222	-2.11335
H	-1.01314	4.93913	-0.25350	H	-2.00963	-4.84633	-3.06451
H	-3.82974	4.23936	-1.38503	H	-2.29366	-0.71975	-1.89486
H	-5.44427	4.08552	-0.65473	H	-0.56289	-4.80417	-2.00486
H	-5.15382	3.49224	-2.30583	H	1.47278	5.25247	0.11368
H	-0.46340	-4.15726	-3.65861	H	-3.01654	-1.08894	2.42794
O	0.55596	-2.37223	-2.00766	H	-1.58508	0.68965	3.36803
C	-0.63582	-2.11188	-1.99405	H	0.39764	-2.82923	0.45971
H	-0.88332	1.18841	-2.39261	H	1.80193	-1.00729	1.30104

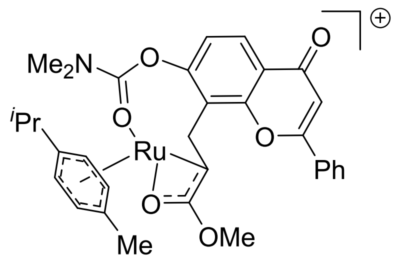

$\mathbf{D}_{\mathbf{Carb.}}$

C	0.53366	-2.35436	-2.49055	O	-3.10629	-4.24198	-1.37648
C	0.59118	-0.96595	-2.31613	O	1.75224	-0.33375	-2.78486
C	-0.62978	-3.02626	-2.15275	C	2.67218	0.20515	-1.95084
C	-1.73225	-2.32008	-1.64923	O	2.52837	0.33009	-0.72300
C	-0.47485	-0.20987	-1.83432	N	3.78076	0.60974	-2.59644
C	-1.63742	-0.93197	-1.50194	C	4.86206	1.25408	-1.85212
C	-2.98656	-3.02416	-1.27538	H	5.16415	2.16767	-2.37339
C	-4.03571	-2.14015	-0.78407	H	5.73000	0.58838	-1.78778
O	-2.67428	-0.19279	-0.99399	H	4.52227	1.50091	-0.84884
C	-3.85792	-0.80101	-0.64970	C	4.02795	0.38702	-4.02293

H	-7.64259	0.29601	1.80860	H	3.39397	-3.48651	3.67759
C	-6.84308	0.64058	1.16025	H	4.90397	-0.57554	1.73291
C	-5.89304	-0.26586	0.69822	C	4.82210	-1.55218	2.22006
H	-5.95151	-1.30507	1.00457	C	3.49981	-2.24866	1.87589
C	-6.76424	1.98797	0.79891	H	1.04975	-2.48240	0.69308
H	-7.50749	2.69244	1.15933	C	3.38657	-3.61455	2.59017
C	-4.84854	0.16287	-0.14051	H	2.46775	-4.14290	2.31826
C	-5.72620	2.42508	-0.02630	H	5.66181	-2.16835	1.88686
C	-4.77234	1.52252	-0.49173	H	3.48859	-2.43754	0.79390
H	-5.66413	3.46966	-0.31584	H	4.23382	-4.25109	2.31882
H	-3.97973	1.86310	-1.14827	H	1.39229	-2.87316	-2.90288
H	-4.99055	-2.58733	-0.53916	H	3.19619	-0.14353	-4.47688
H	-1.32252	1.77034	3.29160	H	4.94357	-0.20051	-4.14781
H	-1.96009	0.24121	3.90435	H	4.16017	1.34890	-4.52894
C	-1.49890	0.72253	3.03405	H	1.66911	5.31063	0.51530
H	1.06702	1.30496	3.86570	O	1.72598	2.61977	0.92078
H	-2.22004	0.67911	2.21371	C	0.84954	2.78877	0.04265
C	1.03236	0.41550	3.24431	H	0.38444	1.68544	-2.28580
C	-0.21587	0.01866	2.68602	C	1.78018	4.90091	-0.48994
C	2.23700	-0.34987	3.06861	C	-0.38361	1.28062	-1.62279
H	3.14181	-0.02095	3.56438	C	-0.06721	1.64199	-0.15541
Ru	1.24240	0.46221	1.06167	O	0.78654	3.86431	-0.71280
C	-0.18465	-1.07949	1.76831	H	-1.32630	1.74765	-1.92460
H	-1.08827	-1.37773	1.25021	H	1.57420	5.66043	-1.24136
C	2.26253	-1.42243	2.17592	H	-0.98160	1.78788	0.42474
C	1.03941	-1.71615	1.46086	H	2.78384	4.49022	-0.61174
H	4.93824	-1.41058	3.29994	H	-0.72976	-4.09995	-2.27183

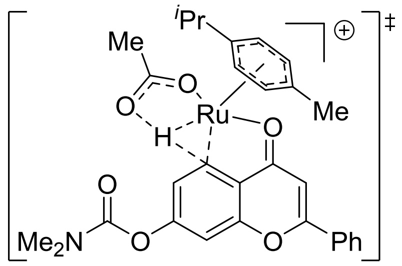

TS A-B$_{Ket.}$

C	-0.04612	2.62850	0.40496	H	8.09391	-1.79531	-0.80462
C	-0.82699	1.48860	0.68435	H	5.40293	2.17058	0.48338
C	1.33181	2.54542	0.22417	H	-1.36879	-4.29032	-1.70304
C	1.91890	1.27653	0.25804	H	-0.05032	-3.10911	-4.11013
C	-0.25165	0.22212	0.66621	H	0.87354	-3.33893	-2.61810
C	1.14316	0.12836	0.41333	C	-1.70433	-3.25969	-1.67752
O	3.27083	1.18165	0.08169	C	0.25509	-2.62889	-3.17326
C	3.88854	-0.02989	0.06530	H	-3.33808	-3.68337	-0.33593
C	1.74777	-1.16380	0.22608	C	-0.96390	-2.25157	-2.38133
C	3.16952	-1.19982	0.14289	C	-2.84264	-2.91016	-0.91444
H	-0.73803	-0.48769	1.57828	H	0.85994	-1.75434	-3.42118
H	1.92611	3.43452	0.05084	H	-5.85282	-2.02993	-1.53946
H	3.67439	-2.15555	0.10923	H	-1.87717	-4.01607	3.74798
O	1.00117	-2.19079	0.10753	C	-1.38528	-0.90748	-2.25705
C	5.34622	0.07909	-0.05672	C	-3.28008	-1.55795	-0.76921
C	5.98937	1.30496	0.19955	O	-1.30181	-2.79101	1.64310
C	7.37425	1.40429	0.10517	Ru	-1.08510	-1.73764	-0.14312
C	8.13648	0.28970	-0.25140	C	-5.73358	-1.12440	-0.93652
C	6.12333	-1.03691	-0.42189	C	-1.26496	-2.18773	2.77651
C	7.50650	-0.92937	-0.51681	H	-2.10781	-2.52181	4.71680
H	7.85970	2.35244	0.31312	H	-4.71032	-2.08958	0.69019
H	9.21648	0.37025	-0.32568	C	-1.46167	-3.04250	4.00702
H	5.65023	-1.98472	-0.65454	C	-2.49835	-0.56755	-1.42974

O	-1.06065	-0.95405	2.90331	H	-1.23765	7.19383	-0.09320
C	-4.53268	-1.23321	0.02783	H	-0.66761	5.99732	1.09301
H	-0.81484	-0.11631	-2.72968	C	-1.54546	6.39696	0.59364
H	-5.61299	-0.27585	-1.61871	H	-2.21642	6.82906	1.34392
H	-6.65713	-0.97223	-0.37036	O	-0.59161	3.88784	0.37348
H	-2.72262	0.48489	-1.28817	N	-2.24595	5.34515	-0.13950
H	-0.48932	-3.18620	4.48934	C	-1.81576	4.07316	-0.27693
C	-4.40799	0.01870	0.90971	H	-4.20456	6.11000	-0.06048
H	-3.57979	-0.07458	1.61890	C	-3.48760	5.73853	-0.80076
H	-5.32839	0.15310	1.48533	O	-2.37701	3.17726	-0.88742
H	-4.26416	0.92861	0.31674	H	-3.29128	6.53578	-1.52630
H	-1.87173	1.61347	0.93075	H	-3.90829	4.87695	-1.31465

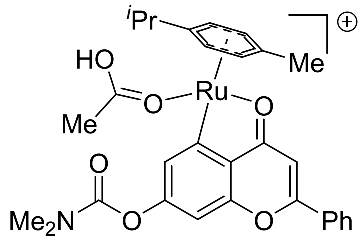

$B_{Ket.}$

C	0.68217	2.56133	0.02698	H	2.46674	-3.21673	-0.07300
C	-0.48640	1.76400	-0.05436	O	-0.05533	-2.29910	-0.19114
C	1.96796	2.01114	0.03782	C	4.83414	-1.72971	0.00169
C	2.04670	0.62627	-0.03993	C	5.83990	-0.81652	0.37209
C	-0.40462	0.38184	-0.14057	C	7.16952	-1.22274	0.42894
C	0.90119	-0.17667	-0.12937	C	7.51724	-2.53804	0.11273
O	3.28549	0.03898	-0.01877	C	5.19609	-3.05141	-0.32230
C	3.43141	-1.30509	-0.04358	C	6.52731	-3.44933	-0.26560
C	1.03416	-1.58928	-0.14964	H	7.93565	-0.51185	0.72139
C	2.33550	-2.14355	-0.09281	H	8.55566	-2.85156	0.15595
H	-0.47202	-2.93289	1.28191	H	4.44476	-3.76484	-0.64314
H	2.85449	2.62507	0.07902	H	6.79485	-4.46880	-0.52420

H	5.57368	0.20381	0.62066	C	-4.98329	-0.05796	0.68279
H	-3.06689	-3.68104	-1.61717	H	-1.92123	0.28852	-2.94099
H	-1.95853	-2.77063	-4.24087	H	-6.57745	0.62492	-0.65245
H	-0.85829	-3.22281	-2.92949	H	-7.11643	0.25127	0.99245
C	-3.16766	-2.60278	-1.54884	H	-3.43023	1.29377	-1.22746
C	-1.39965	-2.38660	-3.37948	H	-2.17093	-2.98146	4.41130
H	-4.55218	-2.68339	0.08931	C	-4.57642	1.32833	1.19891
C	-2.34614	-1.76434	-2.38952	H	-3.53514	1.34039	1.53324
C	-4.00367	-2.03971	-0.59099	H	-5.20840	1.60299	2.04818
H	-0.67207	-1.65896	-3.74548	H	-4.71247	2.10466	0.43787
H	-6.76228	-1.06022	-0.14069	H	-1.43741	2.28587	-0.03914
H	-2.45002	-1.21118	4.34743	H	-0.02246	7.00214	1.75186
C	-2.50835	-0.35938	-2.29996	H	-0.75766	5.48222	1.20244
C	-4.08018	-0.60744	-0.40948	C	-0.28008	6.40057	0.87298
O	-1.89414	-1.14849	1.87925	H	-0.98706	6.97423	0.26117
Ru	-1.84415	-1.07206	-0.31017	O	0.42999	3.90139	0.16280
C	-6.44630	-0.06517	0.18778	N	0.92861	6.10749	0.11098
C	-1.45968	-2.03082	2.63898	C	1.33695	4.87410	-0.27298
H	-0.82278	-1.87749	4.66420	H	1.11841	7.91959	-0.94746
H	-4.90863	-0.75935	1.52377	C	1.71958	7.26018	-0.31079
C	-1.75609	-2.01566	4.10948	O	2.33269	4.61006	-0.91743
C	-3.37371	0.21626	-1.31517	H	2.05013	7.82893	0.56541
O	-0.71674	-3.04044	2.24491	H	2.58743	6.91293	-0.86745

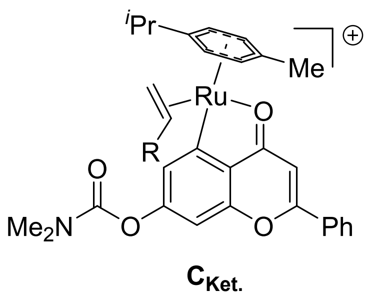

$C_{Ket.}$

R = CO_2Me

C	-0.06780	2.47092	0.22339	H	3.51130	-0.37297	0.85933
C	0.76528	1.33098	0.12483	C	2.61784	-3.65900	0.27191
C	-1.46431	2.38877	0.19739	C	2.96692	-1.30320	0.97961
C	-2.02806	1.12687	0.06235	H	2.22396	-5.55269	-0.69188
C	0.19047	0.07159	-0.00230	H	3.99856	-0.39821	3.59697
C	-1.22023	-0.01081	-0.03284	C	1.56093	-3.72624	1.19241
O	-3.39127	0.99859	0.02201	C	2.01846	-1.44233	2.04540
C	-3.97499	-0.21720	-0.14526	Ru	1.03982	-1.81321	-0.13462
C	-1.80907	-1.29594	-0.21241	C	3.02081	-0.43606	4.08657
C	-3.22896	-1.36654	-0.27599	H	1.97206	0.61271	2.52790
H	-2.07303	3.28095	0.27322	C	1.26128	-2.62956	2.06393
H	-3.71511	-2.31653	-0.45067	C	1.86004	-0.33431	3.06904
O	-1.02416	-2.30309	-0.32049	H	0.96636	-4.63075	1.26800
C	-5.44004	-0.14082	-0.18325	H	2.96244	-1.36870	4.65730
C	-6.08119	1.08644	-0.43560	H	2.96613	0.39615	4.79423
C	-7.47026	1.15393	-0.49009	H	0.44979	-2.74207	2.77249
C	-8.23863	0.00539	-0.28735	C	0.50204	-0.31527	3.78160
C	-6.22339	-1.29121	0.02750	H	-0.32803	-0.26855	3.07069
C	-7.61119	-1.21565	-0.02554	H	0.44072	0.56436	4.42807
H	-7.95399	2.10399	-0.69358	H	0.36422	-1.19295	4.42224
H	-9.32199	0.06132	-0.32859	H	1.83474	1.47758	0.10508
H	-5.75235	-2.24053	0.25900	H	1.45340	6.72741	1.96710
H	-8.20476	-2.10799	0.14552	H	0.09318	5.70352	1.46272
H	-5.48798	1.97830	-0.59907	C	0.93165	6.29483	1.10652
H	3.95777	-2.23385	-0.69659	H	0.55396	7.11651	0.48602
H	3.85023	-5.37489	-0.02093	O	0.40255	3.74722	0.42915
H	3.43774	-4.54757	-1.51958	N	1.85965	5.46649	0.34223
C	3.24987	-2.38302	0.11081	C	1.63196	4.18972	-0.04938
C	3.04451	-4.84647	-0.54483	H	2.84127	6.96232	-0.77397

C	3.08227	6.12449	-0.10928	O	3.54241	-1.03199	-2.59359
O	2.37812	3.50204	-0.72415	C	1.15108	-1.10027	-2.27428
H	3.63609	6.51293	0.75227	O	2.29141	0.83785	-2.86516
H	3.69906	5.40579	-0.64433	H	1.90573	-3.09999	-2.42832
H	3.71305	1.47322	-4.24515	H	3.21196	2.63942	-2.96768
C	3.47085	1.60623	-3.18770	H	0.06119	-2.95939	-2.38476
C	2.46548	-0.45932	-2.58627	H	0.28424	-0.48309	-2.47641
C	1.02824	-2.49266	-2.23461	H	4.31479	1.27930	-2.57900

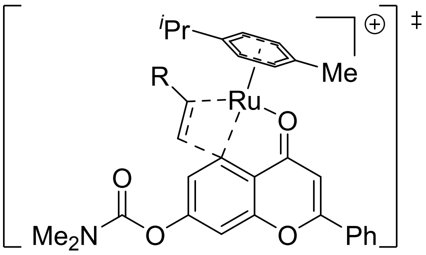

TS C-D$_{Ket.}$

R = CO_2Me

C	-1.31185	-1.22609	-1.14740	C	-4.96393	-4.98946	-0.79684
C	-0.83594	-2.54384	-1.00496	H	-5.03397	-5.91829	-0.21975
C	-0.47430	-0.14052	-0.89180	H	-5.62079	-5.07165	-1.66969
C	0.86591	-0.42164	-0.48796	H	-5.28383	-4.15367	-0.17837
C	0.49443	-2.81970	-0.69951	C	-2.99890	-5.81198	-2.06588
C	1.33845	-1.73390	-0.45229	H	8.06281	-0.28330	0.59252
C	1.69283	0.63322	0.03114	C	7.23734	-0.98644	0.63728
C	3.05753	0.31740	0.29275	C	5.94175	-0.54393	0.39178
O	2.64154	-1.99064	-0.13304	H	5.77347	0.49706	0.13711
C	3.49574	-0.97923	0.18652	C	7.47649	-2.33250	0.92738
O	1.15918	1.75603	0.30499	H	8.48881	-2.67514	1.11796
O	-1.62875	-3.63616	-1.26475	C	4.86295	-1.44700	0.43537
C	-2.94479	-3.65936	-0.80195	C	6.41307	-3.23740	0.96466
O	-3.41150	-2.77705	-0.09914	C	5.11432	-2.80258	0.71744
N	-3.58578	-4.76919	-1.22786	H	6.59590	-4.28366	1.18814

H	4.29094	-3.50631	0.75111	C	-3.62248	-0.65845	3.42024
H	3.72403	1.09328	0.64336	H	-4.52264	-0.35878	2.87498
H	-2.67676	5.04080	-0.57161	H	-1.25880	-2.11787	3.58618
H	-1.28101	5.41704	0.45065	H	-2.52744	-1.26119	1.67725
C	-2.27538	4.96592	0.44155	H	-3.77210	-1.68297	3.77312
H	-0.56731	3.81208	2.27119	H	-2.32843	-1.07296	-1.48418
H	-2.92492	5.55295	1.10160	H	-1.99520	-5.53580	-2.37584
C	-1.30753	3.11236	1.89834	H	-3.61949	-5.95893	-2.95654
C	-2.23078	3.53994	0.91570	H	-2.95756	-6.75884	-1.51515
C	-1.34011	1.78144	2.44274	H	2.60059	4.96223	-1.37581
H	-0.61789	1.52457	3.20849	O	1.67510	2.67031	-2.43573
Ru	-0.97590	1.77692	0.12584	C	0.66902	3.23756	-2.05312
C	-3.07982	2.53250	0.35491	H	0.13641	0.82315	-2.73468
H	-3.74328	2.78523	-0.46570	C	1.81298	5.30028	-2.05338
C	-2.23218	0.81308	1.96342	C	-0.72706	1.15710	-2.16136
C	-3.03305	1.19826	0.83069	C	-0.64448	2.57431	-1.80786
H	-0.95544	-0.50072	4.21032	O	0.59709	4.56382	-1.81700
H	-3.52264	-0.00805	4.29598	H	-1.67298	0.84467	-2.59326
H	-0.21769	-1.01328	2.67863	H	1.56584	6.34416	-1.86371
C	-1.12279	-1.07363	3.29148	H	-1.49936	3.20020	-2.05164
C	-2.36184	-0.58886	2.52788	H	2.14718	5.16550	-3.08437
H	-3.65707	0.44578	0.35808	H	0.85549	-3.83903	-0.64255

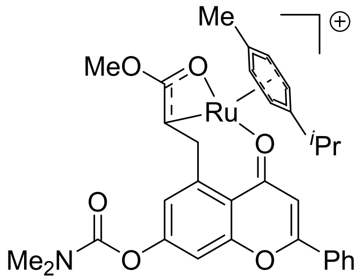

$D_{Ket.}$

C	-2.36912	-1.51066	0.99496	C	-3.44460	-0.85619	0.36559

C	-1.19226	-0.83565	1.27840	H	0.88061	3.35150	1.05848
C	-1.08125	0.53756	0.91243	H	-0.14536	-3.33932	-1.54041
C	-3.40313	0.50551	0.10859	H	-0.28844	-2.66447	-3.16395
C	-2.22713	1.18949	0.41704	C	-0.16260	-2.40477	-2.10589
C	0.14242	1.32308	1.01218	H	2.38377	-3.36003	-1.54200
C	-0.00183	2.74303	0.91312	H	-1.04267	-1.84044	-1.79398
O	-2.24910	2.54055	0.20479	C	2.35817	-2.28459	-1.68194
C	-1.17933	3.31452	0.51021	C	1.11191	-1.62633	-1.93400
O	1.29825	0.81180	1.14071	C	3.54661	-1.52598	-1.53094
O	-4.62053	-1.50621	0.08371	H	4.45484	-2.04511	-1.24427
C	-4.54983	-2.71405	-0.61916	Ru	2.05746	-0.80885	-0.06361
O	-3.50294	-3.14075	-1.07204	C	1.14559	-0.20757	-2.00433
N	-5.77054	-3.28594	-0.72083	H	0.21181	0.33282	-2.11725
C	-5.89616	-4.52979	-1.47485	C	3.60744	-0.11024	-1.81672
H	-6.57400	-4.38967	-2.32456	C	2.38573	0.52373	-2.03411
H	-6.30224	-5.31921	-0.83261	H	6.12932	-0.63888	-0.52569
H	-4.91491	-4.82687	-1.83904	H	5.98188	-0.74559	-3.15792
C	-7.00422	-2.74097	-0.16214	H	5.23487	0.62766	0.33176
H	-0.14225	7.79392	1.13104	C	5.78529	0.39922	-0.58529
C	-0.80232	7.06240	0.67599	C	4.92381	0.64597	-1.83601
C	-0.57097	5.70590	0.87854	H	2.35395	1.60295	-2.14700
H	0.25807	5.39564	1.50573	C	5.70266	0.31340	-3.12609
C	-1.88615	7.48184	-0.10047	H	5.11321	0.53454	-4.02100
H	-2.06446	8.54124	-0.25661	H	6.67675	1.03255	-0.61920
C	-1.42483	4.74606	0.30351	H	4.67177	1.71359	-1.86201
C	-2.74289	6.53676	-0.66927	H	6.62412	0.90157	-3.17236
C	-2.51929	5.17767	-0.46840	H	-2.47539	-2.55283	1.26975
H	-3.58670	6.85902	-1.27109	H	-6.80007	-1.84181	0.41230
H	-3.18468	4.44705	-0.91305	H	-7.47214	-3.48411	0.49320

H	-7.70903	-2.50045	-0.96676	C	1.09484	-2.07343	1.31557
H	4.45573	-3.02223	3.49262	O	2.38992	-2.74955	3.28373
O	3.26072	-1.36680	1.72815	H	-0.58500	-2.33494	2.65803
C	2.32857	-2.07848	2.15531	H	3.43726	-3.23964	4.95411
H	0.24371	-0.80022	2.84115	H	0.87893	-3.06513	0.90818
C	3.61089	-2.63634	4.06547	H	3.78772	-1.59234	4.32909
C	-0.11621	-1.51743	2.09764	H	-4.25488	1.02565	-0.31203

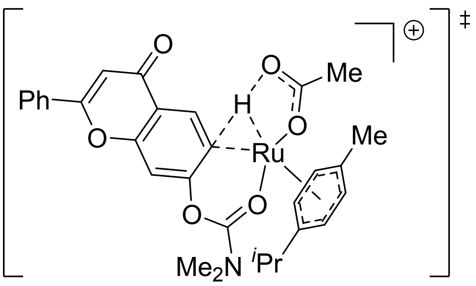

TS C-H Activation C6

C	0.39395	0.12825	-0.96043	H	4.39067	2.96118	0.21645
C	-0.15881	1.29699	-0.37618	H	4.05518	4.53662	0.98133
C	-0.53808	-0.87207	-1.30059	H	4.29332	4.43535	-0.77861
C	-1.90000	-0.77314	-1.02361	C	1.64573	4.90136	-0.32248
C	-1.50828	1.45905	-0.10425	H	0.62726	4.63579	-0.58994
C	-2.37381	0.40641	-0.42758	H	2.09208	5.47632	-1.13879
C	-2.84065	-1.87857	-1.33356	H	1.63499	5.52306	0.57932
C	-4.22977	-1.56438	-1.03682	H	1.25802	0.41333	-1.88720
O	-3.68743	0.58472	-0.14566	H	-0.21082	-1.77483	-1.80740
C	-4.60822	-0.39289	-0.46034	H	-1.89238	2.37498	0.32937
O	-2.45143	-2.95236	-1.79042	H	-9.06054	-1.33424	0.45612
O	0.59744	2.43573	-0.11142	C	-8.29008	-0.57740	0.34788
C	1.94392	2.46218	-0.00642	C	-6.98984	-0.95975	0.03379
O	2.66149	1.46176	0.21062	H	-6.75783	-2.01310	-0.08179
N	2.45127	3.69896	-0.09503	C	-8.60092	0.77229	0.53376
C	3.88654	3.91647	0.09451	H	-9.61630	1.06775	0.77933

C	-5.97715	0.00720	-0.10429	C	3.12122	0.15973	-2.73454
C	-7.60094	1.73840	0.40824	O	3.42197	-0.51626	-1.69589
C	-6.29718	1.36219	0.09504	O	2.03225	0.79266	-2.87159
H	-7.83704	2.78830	0.55127	H	4.76949	-2.20471	0.54780
H	-5.52636	2.11684	-0.00807	H	4.15956	1.17112	-4.31186
H	-4.97559	-2.30320	-1.29952	H	3.75991	-0.51218	-4.65150
H	2.17746	-4.04793	-1.51452	C	2.05693	-0.99380	2.32802
H	3.50986	-4.69059	-0.54132	C	3.41869	-1.20366	1.89825
C	3.06204	-3.77171	-0.93674	H	-0.35466	-0.31197	3.80001
H	0.57100	-3.13049	0.07174	H	1.58066	-1.56206	5.01380
H	3.78630	-3.30224	-1.60747	H	0.02357	0.93894	2.59916
C	1.37428	-2.62664	0.59778	C	0.34788	0.48762	3.54147
C	2.71215	-2.84615	0.19418	C	1.79096	-0.02591	3.46799
H	5.09823	-0.14775	-3.53593	H	4.20683	-0.60170	2.33891
C	1.05133	-1.72080	1.66217	C	2.20768	-0.69023	4.80003
H	0.00941	-1.55522	1.90558	H	3.25111	-1.01930	4.78589
Ru	2.24288	-0.63413	0.04821	H	0.26844	1.24748	4.32370
C	4.11240	0.17255	-3.87283	H	2.45202	0.83534	3.30286
C	3.74224	-2.11895	0.88531	H	2.09118	0.01999	5.62364

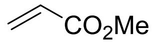

C	-8.01759	2.38168	0.00839	O	-5.67813	0.70262	0.09376
C	-6.76753	2.84533	-0.03529	O	-4.44501	2.59722	-0.04364
H	-8.87452	3.04619	-0.02614	C	-3.26070	1.78656	-0.00302
H	-8.20156	1.31404	0.08040	H	-3.22051	1.20964	0.92434
C	-5.61277	1.91297	0.01366	H	-2.42389	2.48259	-0.05759
H	-6.54091	3.90443	-0.10719	H	-3.23934	1.09334	-0.84767

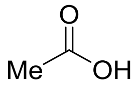

C	-5.97425	-0.43966	0.00822
C	-4.77793	0.47856	-0.06026
H	-6.49984	-0.40426	-0.94976
H	-6.66219	-0.06447	0.77058
H	-5.69055	-1.46648	0.23781
O	-4.81986	1.66488	-0.29840
O	-3.61493	-0.17625	0.17899
H	-2.91331	0.49443	0.11282

4. DEUTERIUM LABELLING STUDIES

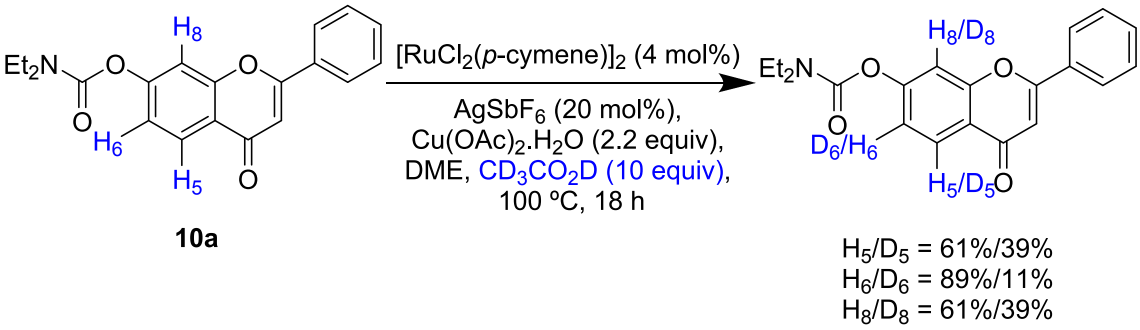

Figure 1: ^{1}H NMR (400 MHz, $CDCl_3$) Spectra of Compound D-10a.

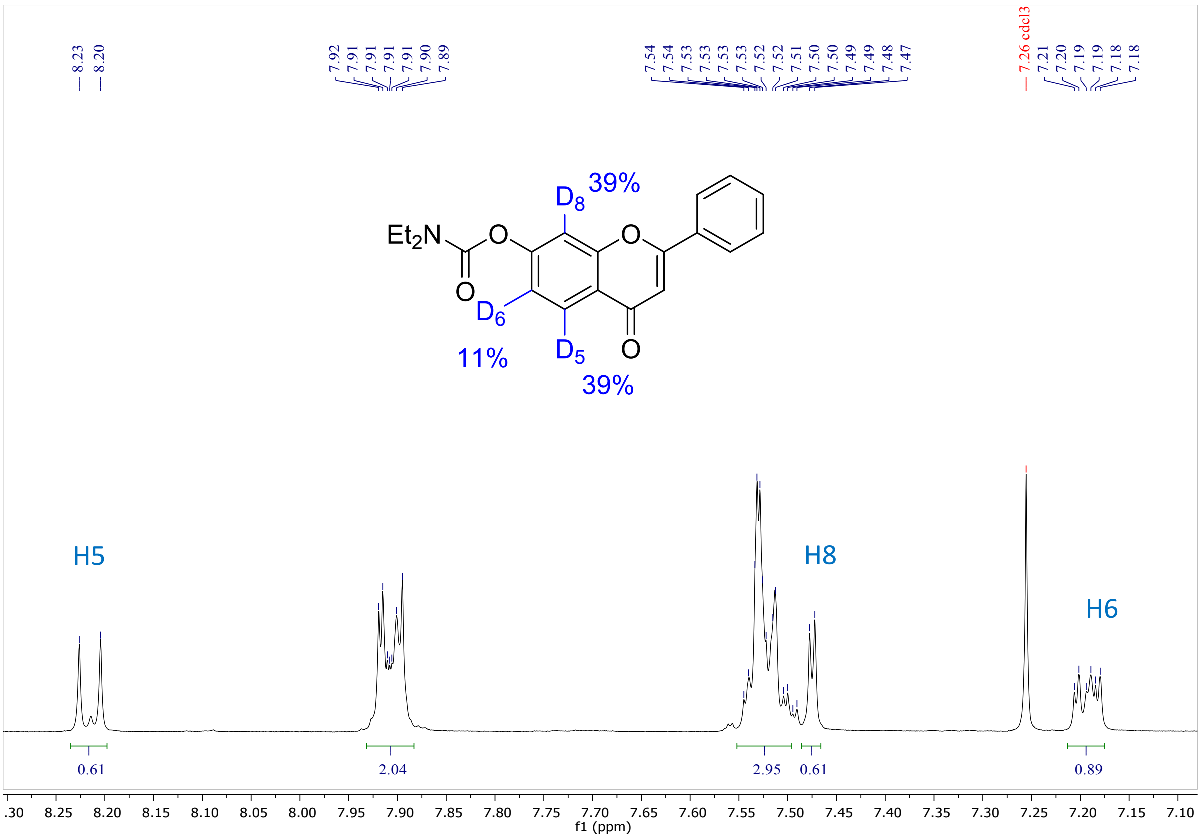

5. References

[1] (a) Schaeffer, E.; de Oliveira, N. C.; Pestana, Y.; Alves, M. A.; da Silva, A. J. M. Regio- and Chemoselective Synthesis of Polyaryl Flavones by Combination of C–O/C–H Activation and Suzuki–Miyaura Cross-Coupling Reactions. *J. Mol. Struct.* **2024**, *1299*, 137067. (b) Shah, U.; Patel, S.; Patel, M.; Jain, N.; Pandey, N.; Chauhan, A.; Ashish. In Vitro Cytotoxicity and Aromatase Inhibitory Activity of Flavonoids: Synthesis, Molecular Docking, and *In Silico* ADME Prediction. *Curr. Comput.-Aided Drug Des.* **2022**, *22*, 1370–1385. (c) Shoaib, M.; Shah, S. W. A.; Ghias, M.; Ali, N.; Umar, N.; Shah, I.; Shafiullah; Nisar, M.; Jan, T.; Tahir, M. N. Synthesis, Crystal Studies, and Biological Evaluation of Flavone Derivatives. *Pak. J. Pharm. Sci.* **2020**, *33*, 11–20. (d) Naik, M. M.; Tilve, S. G.; Kamat, V. P**.** Pyrrolidine- and Iodine-Catalyzed Domino Aldol–Michael–Dehydrogenative Synthesis of Flavones. *Tetrahedron Lett.* **2014**, *55*, 3340–3343. (e) Kawaii, S.; Ikuina, T.; Hikima, T.; Tokiwano, T.; Yoshizawa, Y. Relationship between Structure and Antiproliferative Activity of Polymethoxyflavones toward HL60 Cells. *Anticancer Res.* **2012**, *32*, 5239–5244. (f) Kampschulte, N.; Berking, T.; Çelik, I. E.; Kirsch, S. F.; Schebb, N. H. Inhibition of Cytochrome P450 Monooxygenase-Catalyzed Oxylipin Formation by Flavonoids: Evaluation of Structure–Activity Relationship toward CYP4F2-Selective Inhibitors. *Eur. J. Med. Chem.* **2022**, *238*, 114332. (g) Lim, D.-S.; Lim, D.-H.; Lee, J.-H.; Oh, E.-T.; Keum, Y.-S. Structure–Oxidative Metabolism Relationships of Substituted Flavones by Aspergillus niger. *J. Agric. Food Chem.* **2017**, *65* (14), 3056–3064.

[2] (a) Dao, T. T.; Chi, Y. S.; Kim, J.; Kim, H. P.; Kim, S.; Park, H. Synthesis and Inhibitory Activity against COX-2-Catalyzed Prostaglandin Production of Chrysin Derivatives. *Bioorg. Med. Chem. Lett.* **2004**, *14*, 1165–1167. (b) Zhang, Z.; Lai, H.; Tian, S.; Song, Y.; Na, C.; Shi, X.; He, C.; Shi, L.; Wang, S.; Ali, F.; Shu, F.; Zhang, B. Design, Synthesis, and Biological Evaluation of Chromone Derivatives as STAT1 Inhibitors for Treatment of Nonalcoholic Steatohepatitis. *J. Med. Chem.* **2026**, *69* (3), 2509–2538.

[3] Zhao, Y.; Liao, J.; Liu, C. Palladium-Catalyzed Regioselective Arylation of 7-Hydroxyflavone with Diaryliodonium Salts. *Tetrahedron Lett.* **2023**, *123*, 154573.

[4] Deng, C.; Mi, J.; Zhou, Y.; et al. Design, Synthesis, and Biological Evaluation of Novel 2-Acetylphenol-Rivastigmine Hybrids as Potential Multifunctional Agents for the Treatment of Alzheimer's Disease. *Med. Chem. Res.* **2022**, *31*, 1035–1048.

[5] Reddy, M. C.; Jeganmohan, M. Ruthenium-Catalyzed Selective Aerobic Oxidative *ortho*-Alkenylation of Substituted Phenols with Alkenes through C–H Bond Activation. *Eur. J. Org. Chem.* **2013**, 1150-1157.

[6] Gaussian 16, Revision C.01, Frisch, M. J.; Trucks, G. W.; Schlegel, H. B.; Scuseria, G. E.; Robb, M. A.; Cheeseman, J. R.; Scalmani, G.; Barone, V.; Petersson, G. A.; Nakatsuji, H.; Li, X.; Caricato, M.; Marenich, A. V.; Bloino, J.; Janesko, B. G.; Gomperts, R.; Mennucci, B.; Hratchian, H. P.; Ortiz, J. V.; Izmaylov, A. F.; Sonnenberg, J. L.; Williams-Young, D.; Ding, F.; Lipparini, F.; Egidi, F.; Goings, J.; Peng, B.; Petrone, A.; Henderson, T.; Ranasinghe, D.; Zakrzewski, V. G.; Gao, J.; Rega, N.; Zheng, G.; Liang, W.; Hada, M.; Ehara, M.; Toyota, K.; Fukuda, R.; Hasegawa, J.; Ishida, M.; Nakajima, T.; Honda, Y.; Kitao, O.; Nakai, H.; Vreven, T.; Throssell, K.; Jr. Montgomery, J. A.; Peralta, J. E.; Ogliaro, F.; Bearpark, M. J.; Heyd, J. J.; Brothers, E. N.; Kudin, K. N.; Staroverov, V. N.; Keith, T. A.; Kobayashi, R.; Normand, J.; Raghavachari, K.; Rendell, A. P.; Burant, J. C.; Iyengar, S. S.; Tomasi, J.; Cossi, M.; Millam, J. M.; Klene, M.; Adamo, C.; Cammi, R.; Ochterski, J. W.; Martin, R. L.; Morokuma, K.; Farkas, O.; Foresman, J. B.; Fox, D. J. *Gaussian,* Inc., Wallingford CT, 2016

[7] (a) Lee, C.; Yang, W.; Parr, R. G. Development of the Colle-Salvetti correlation-energy formula into a functional of the electron density. *Phys. Rev. B* **1988**, *37*, 785-789. (b) Becke, A. D. Density-functional thermochemistry. III. The role of exact exchange. *J. Chem. Phys.* **1993**, *98*, 5648-5652. (c) Kohn, W.; Becke, A. D.; Parr, R. G. Density Functional Theory of Electronic Structure. *J. Phys. Chem.* **1996**, *100*, 12974-12980.

[8] Dunning Jr., T. H.; Hay, P. J. in *Modern Theoretical Chemistry* Vol. 3, (Ed. H. F. Schaefer III), Plenum, New York, 1977, pp. 1-28.

[9] Zhao, Y.; Truhlar, D. G. The M06 suite of density functionals for main group thermochemistry, thermochemical kinetics, noncovalent interactions, excited states, and transition elements: two new functionals and systematic testing of four M06-class functionals and 12 other functionals. *Theor. Chem. Acc.* **2008**, *120*, 215-241.

[10] (a) Dolg, M.; Wedig, U.; Stoll, H.; Preuss, H. Energy-adjusted *ab initio* pseudopotentials for the first-row transition elements. *J. Chem. Phys.* **1987**, *86*, 866-872. (b) Andrae, D.; Häußermann, U.; Dolg, M.; Stoll, H.; Preuß, H. Energy-adjusted *ab initio* pseudopotentials for the second and third row transition elements. *Theor. Chim. Acta* **1990**, *77*, 123-141.

[11] (a) Cancés, E.; Mennucci, B.; Tomasi, J. A new integral equation formalism for the polarizable continuum model: Theoretical background and applications to isotropic and anisotropic dielectrics. *J. Chem. Phys.* **1997**, *107*, 3032-3041. (b) Cossi, M.; Barone, V.; Mennucci, B.; Tomasi, J. Ab initio study of ionic solutions by a polarizable continuum dielectric model. *Chem. Phys. Lett.* **1998**, *286*, 253-260. (c) Tomasi, J.; Mennucci, B.; Cancès, E. The IEF version of the PCM solvation method: an overview of a new method addressed to study molecular solutes at the QM ab initio level. *Mol. Struc.: THEO CHEM* **1999**, *464*, 211-226.

6. COPIES OF NMR SPECTRA

^{1}H NMR (400 MHz, $CDCl_3$) Spectra of 7-methoxy-2-phenyl-4H-chromen-4-one (**7a**)

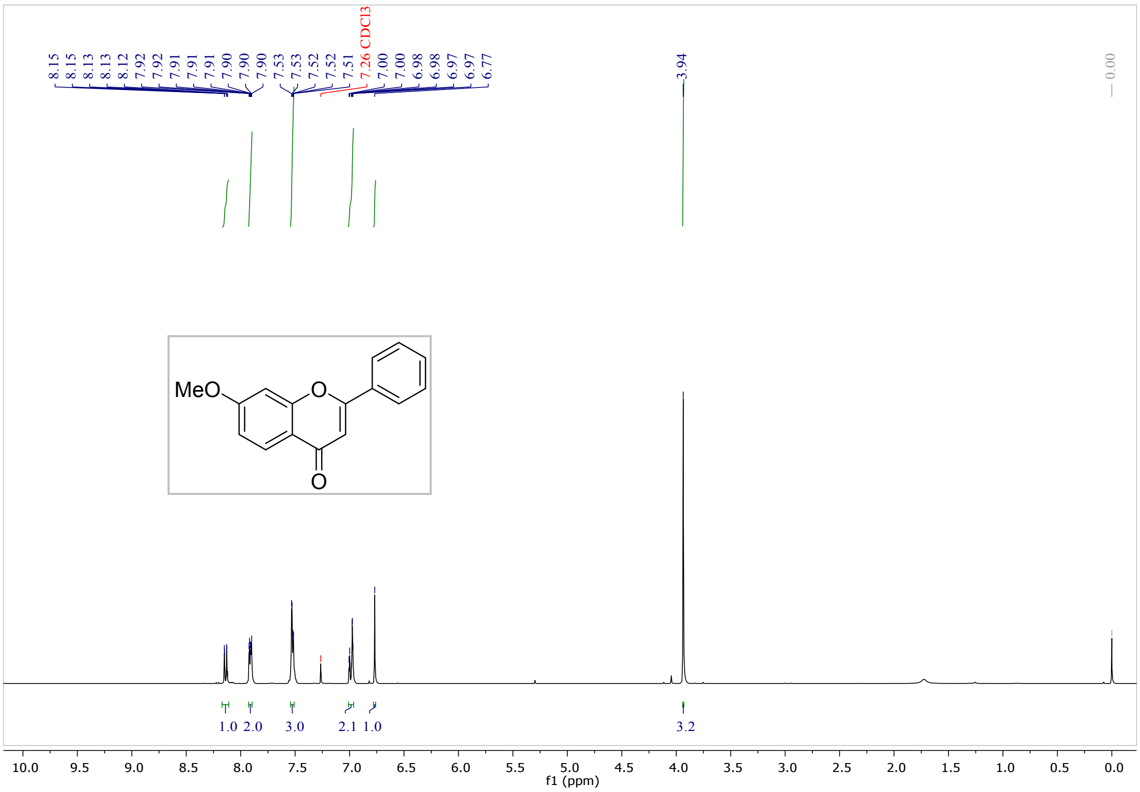

$^{13}C\{^{1}H\}$NMR (126 MHz, $CDCl_3$) Spectra of 7-methoxy-2-phenyl-4H-chromen-4-one (**7a**)

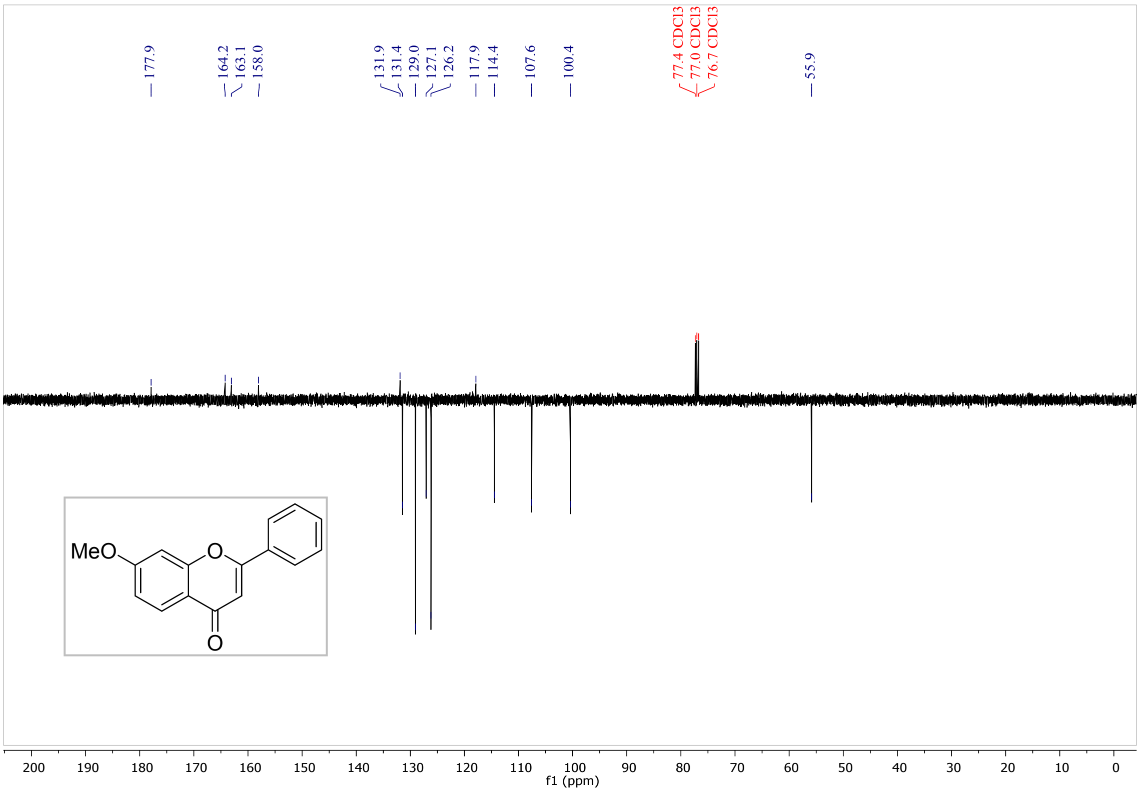

^{1}H NMR (400 MHz, $CDCl_3$) Spectra of 2-(4-fluorophenyl)-7-methoxy-4H-chromen-4-one (**7b**)

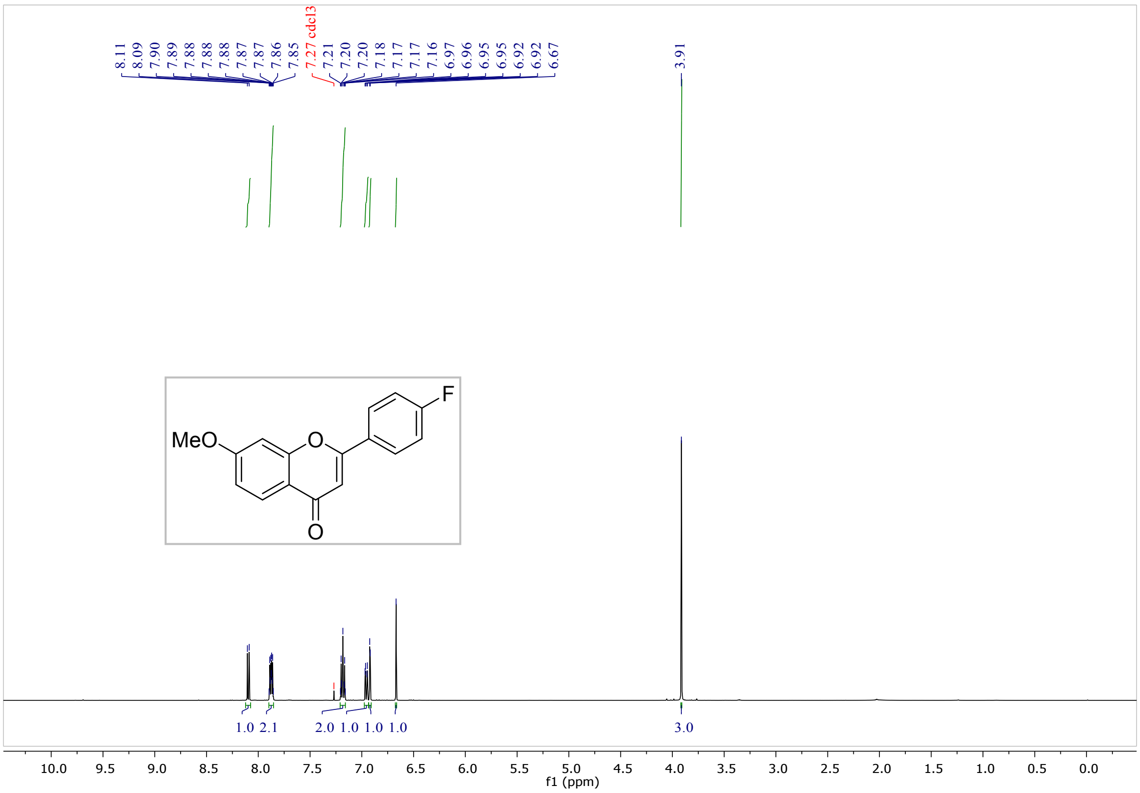

$^{13}C\{^1H\}$NMR (126 MHz, $CDCl_3$) Spectra of 2-(4-fluorophenyl)-7-methoxy-4H-chromen-4-one (**7b**)

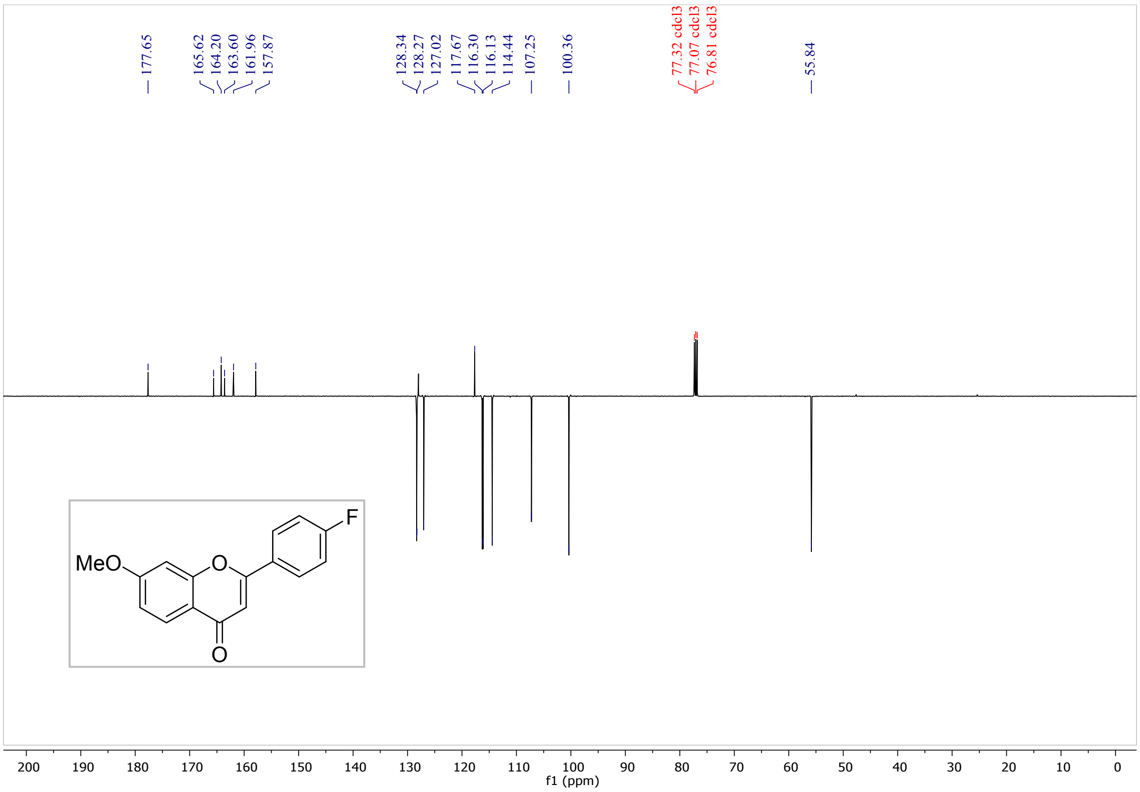

^1H NMR (500 MHz, $CDCl_3$) Spectra of 7-methoxy-2-(4-(trifluoromethyl)phenyl)-4H-chromen-4-one (**7c**)

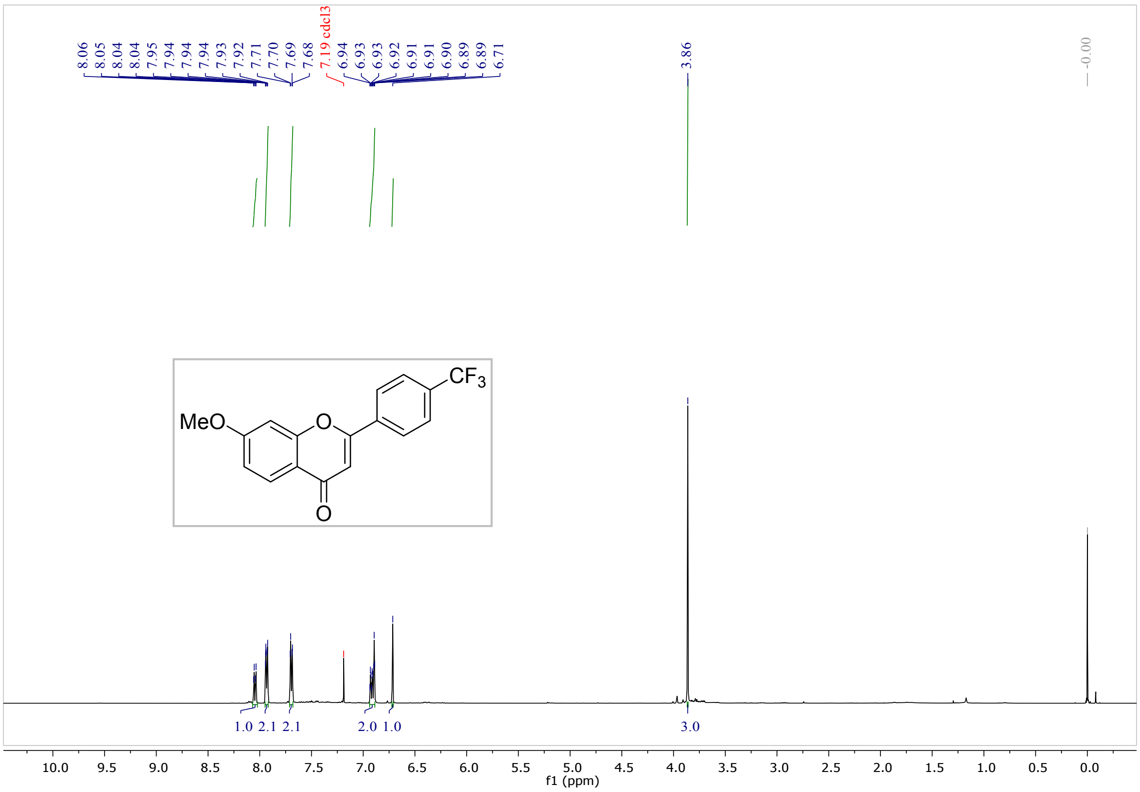

$^{13}C\{^{1}H\}$NMR (126 MHz, $CDCl_3$) Spectra of 7-methoxy-2-(4-(trifluoromethyl)phenyl)-4H-chromen-4-one (**7c**)

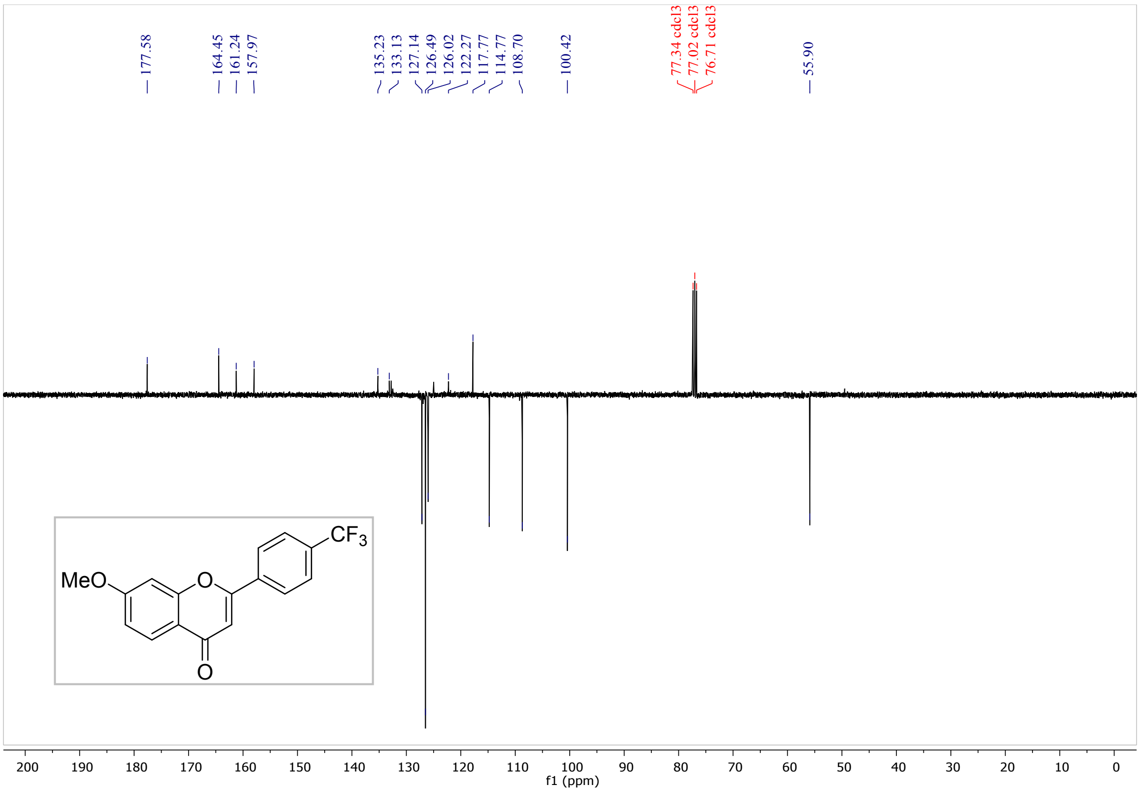

^{1}H NMR (500 MHz, $CDCl_3$) Spectra of 7-methoxy-2-(4-methoxyphenyl)-4H-chromen-4-one (**7d**)

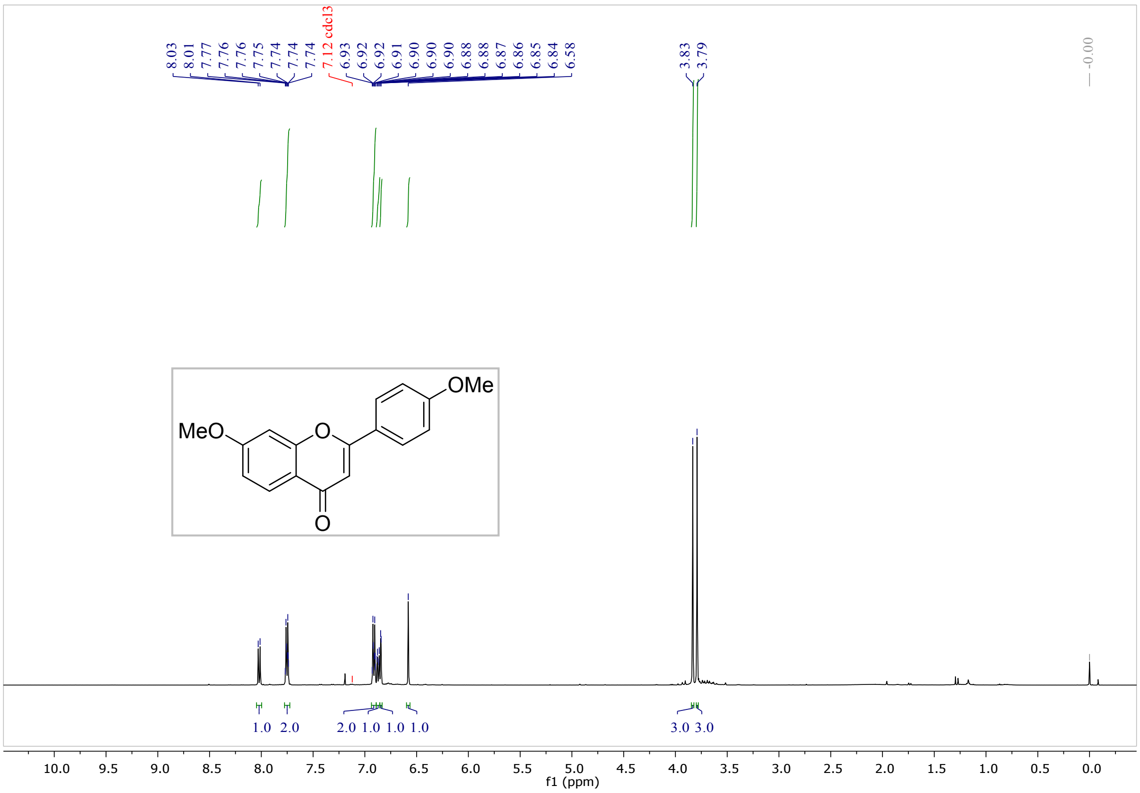

$^{13}C\{^{1}H\}$NMR (126 MHz, $CDCl_3$) Spectra of 7-methoxy-2-(4-methoxyphenyl)-4H-chromen-4-one (**7d**)

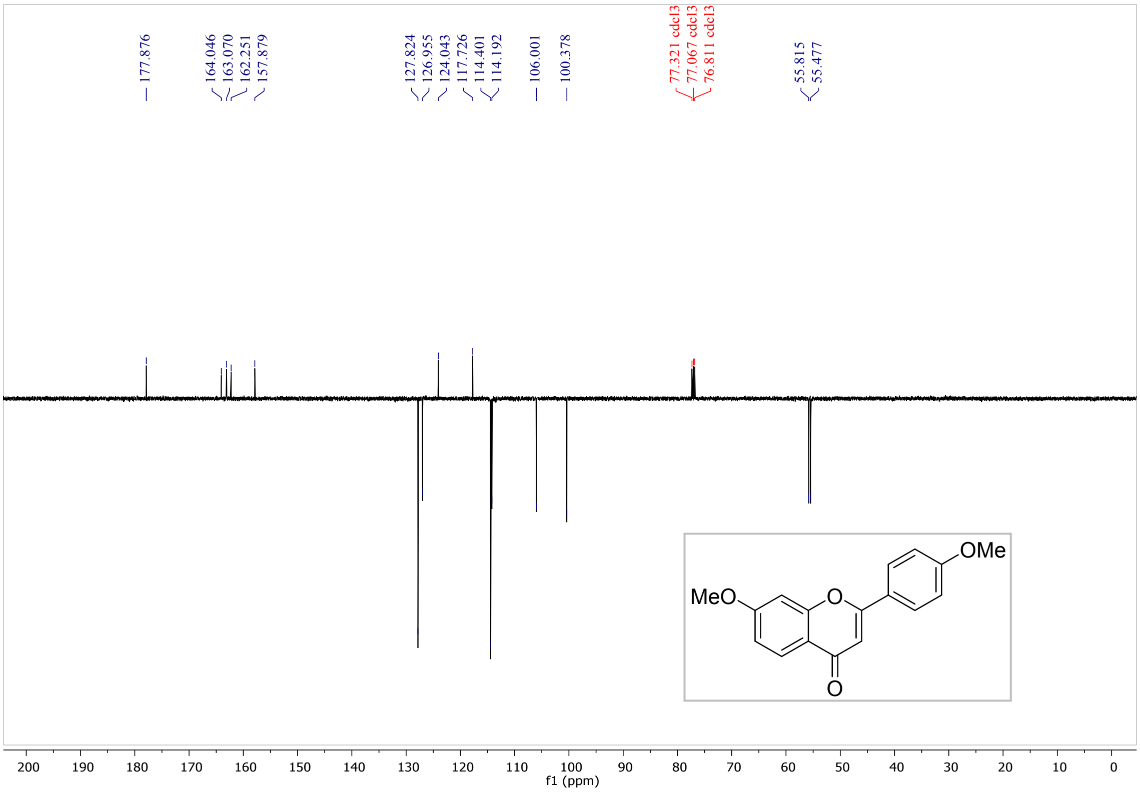

^{1}H NMR (500 MHz, $CDCl_3$) Spectra of 2-(3,4-dimethoxyphenyl)-7-methoxy-4H-chromen-4-one (**7e**)

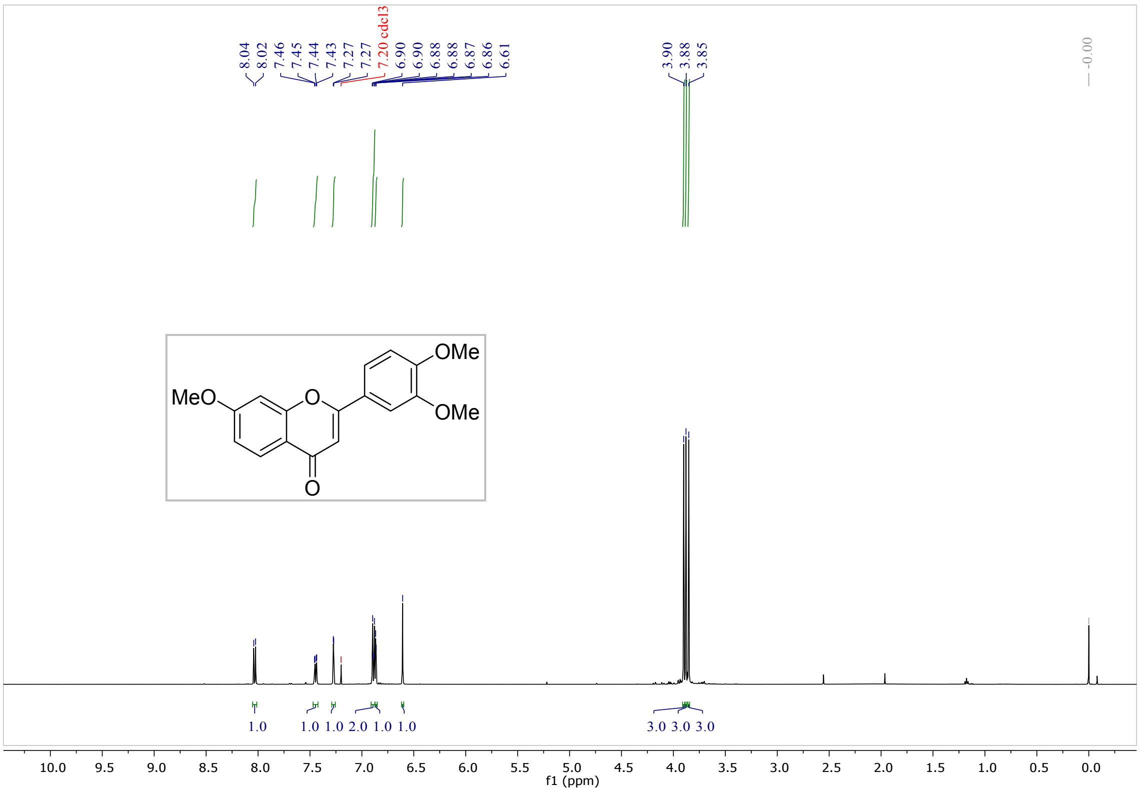

$^{13}C\{^{1}H\}$NMR (126 MHz, $CDCl_3$) Spectra of 2-(3,4-dimethoxyphenyl)-7-methoxy-4H-chromen-4-one (**7e**)

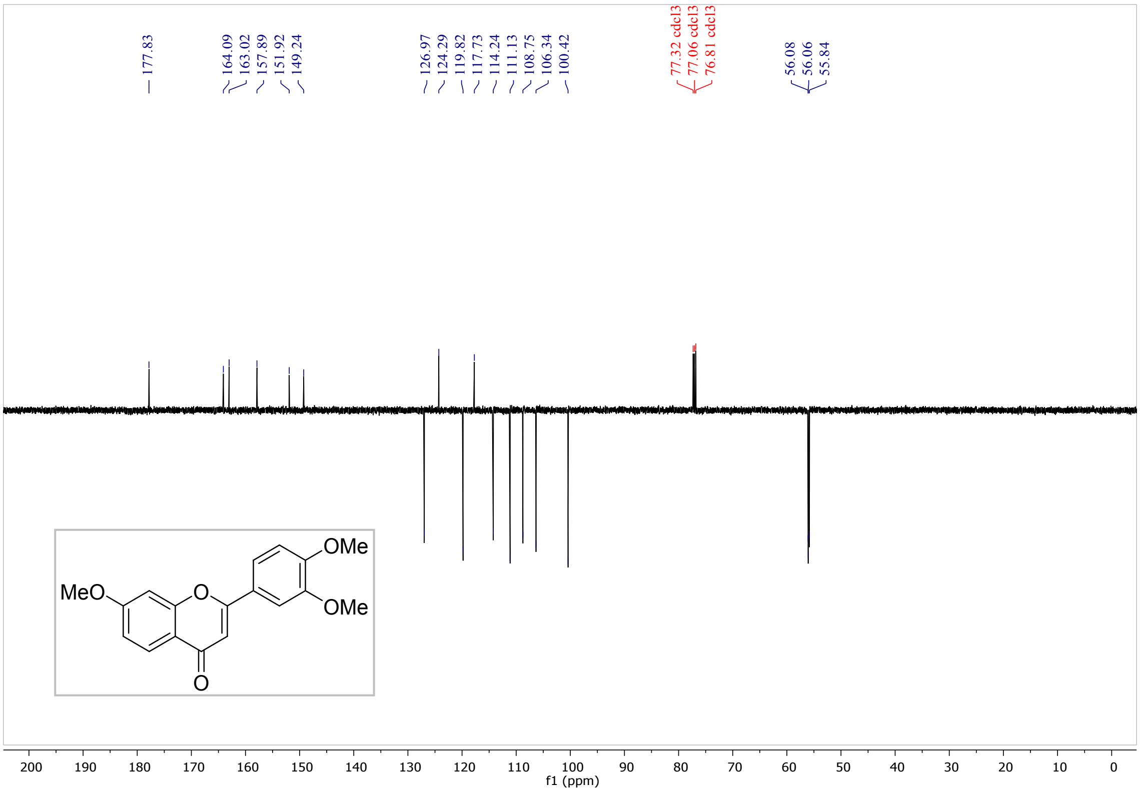

^{1}H NMR (500 MHz, $CDCl_3$) Spectra of 2-(3,4-dimethoxyphenyl)-6-methoxy-4H-chromen-4-one (**7f**)

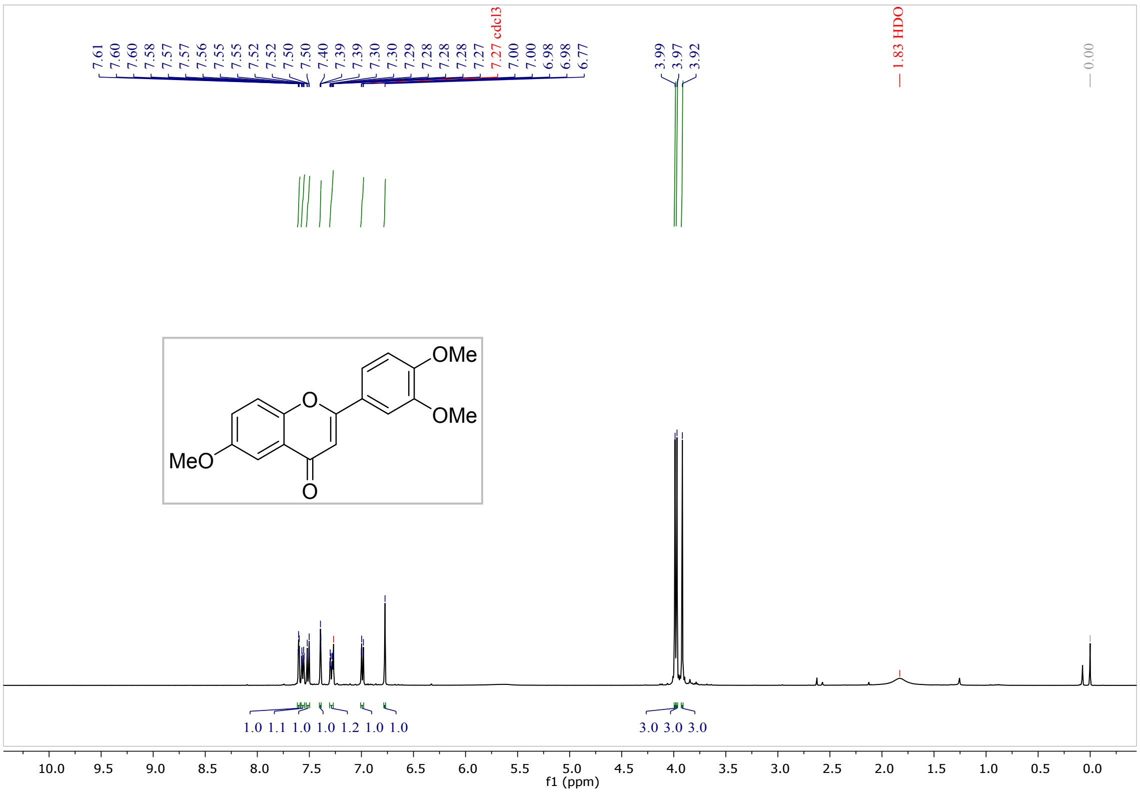

$^{13}C\{^{1}H\}$NMR (126 MHz, $CDCl_3$) Spectra of 2-(3,4-dimethoxyphenyl)-6-methoxy-4H-chromen-4-one (**7f**)

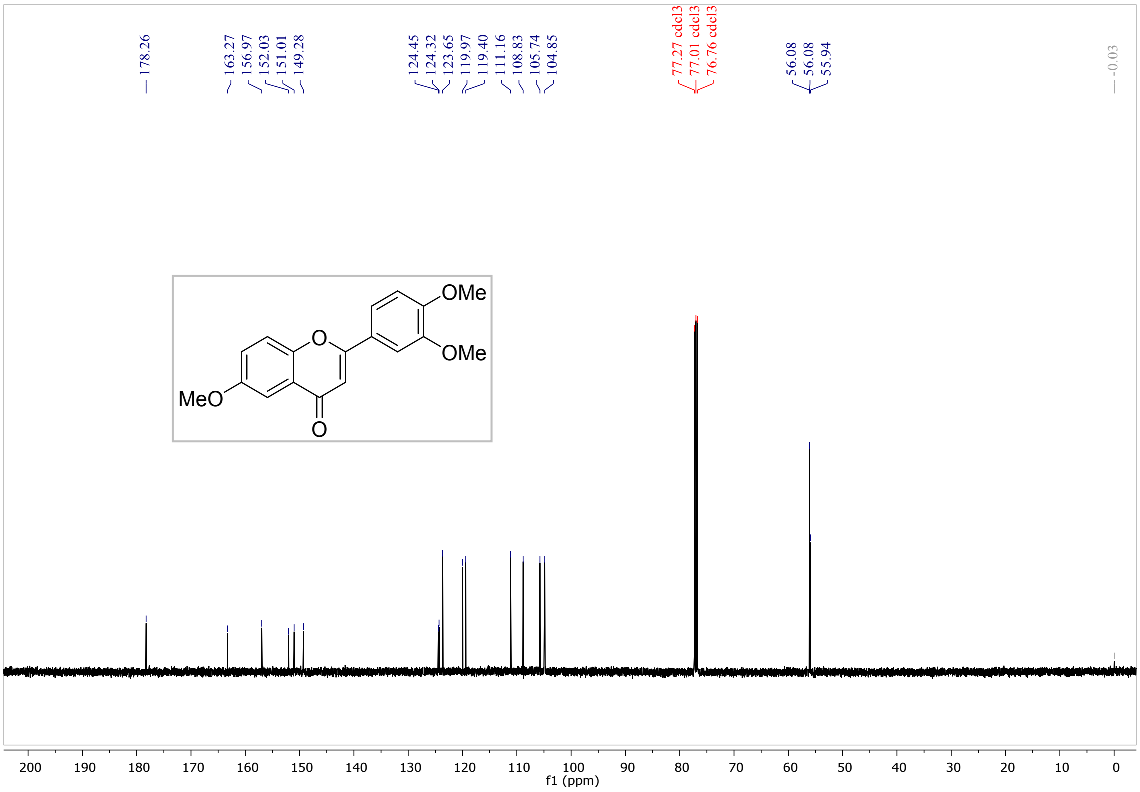

^{1}H NMR (500 MHz, $CDCl_3$) Spectra of 7-bromo-2-phenyl-4H-chromen-4-one (**7g**)

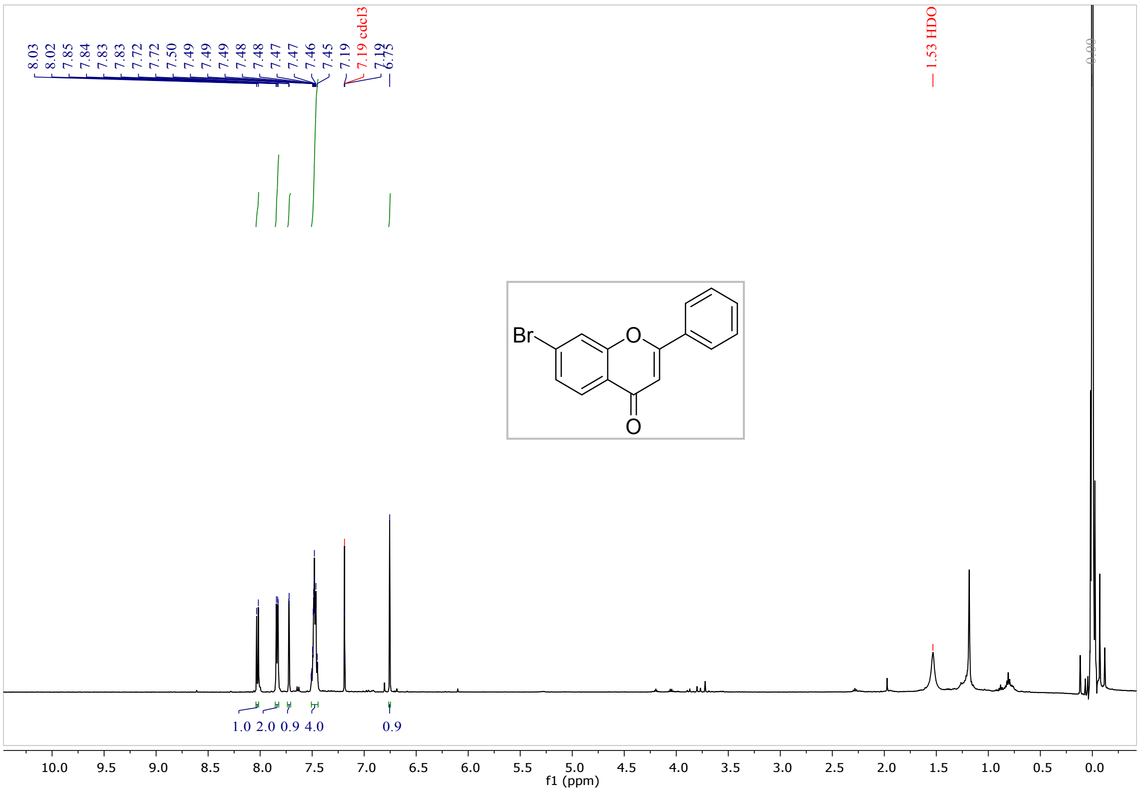

^{1}H NMR (500 MHz, Methanol-d_4) Spectra of 7-hydroxy-2-phenyl-4H-chromen-4-one

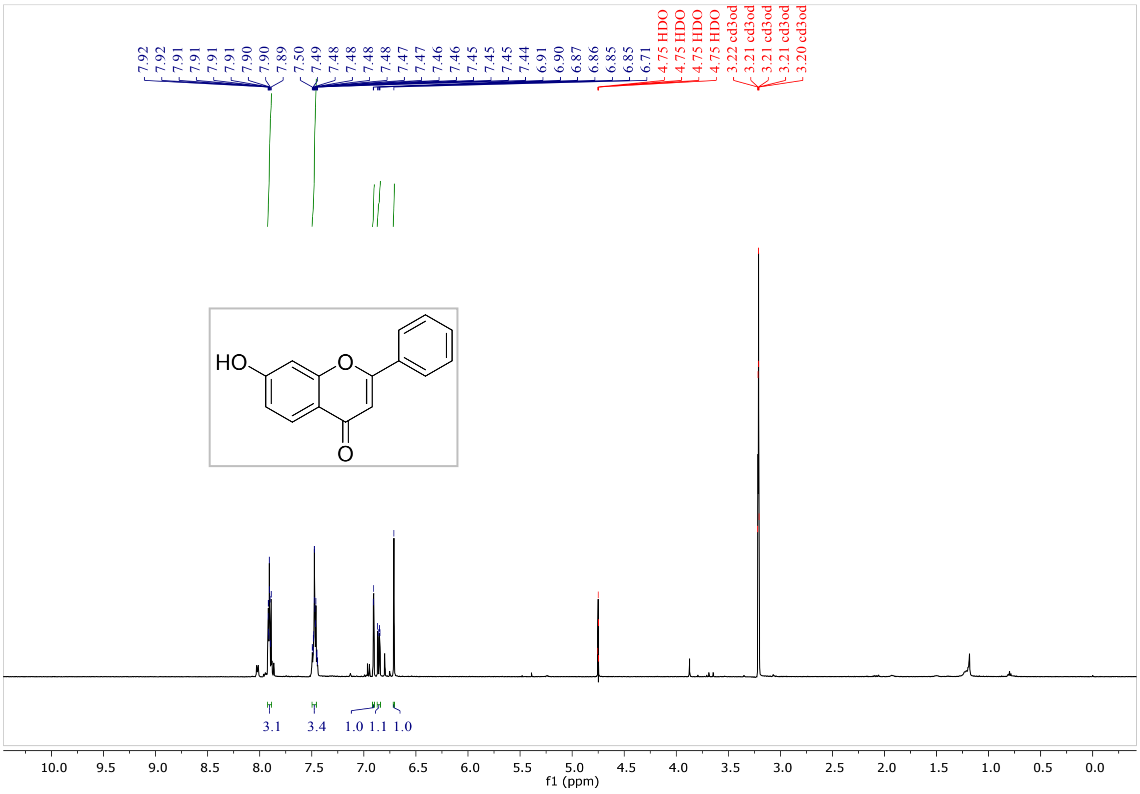

^{1}H NMR (500 MHz, Methanol-d_4) Spectra of 7-hydroxy-2-(4-(trifluoromethyl)phenyl)-4H-chromen-4-one

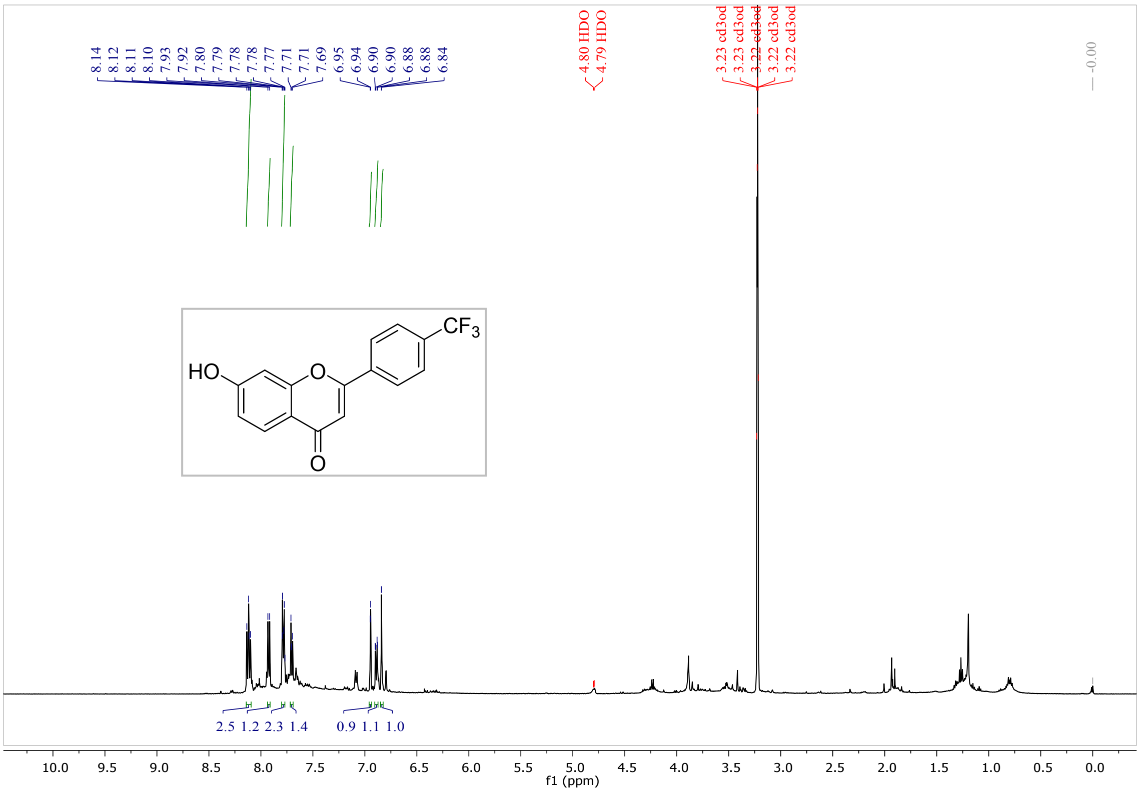

^{1}H NMR (500 MHz, Methanol-d_4) Spectra of 2-(4-fluorophenyl)-7-hydroxy-4H-chromen-4-one

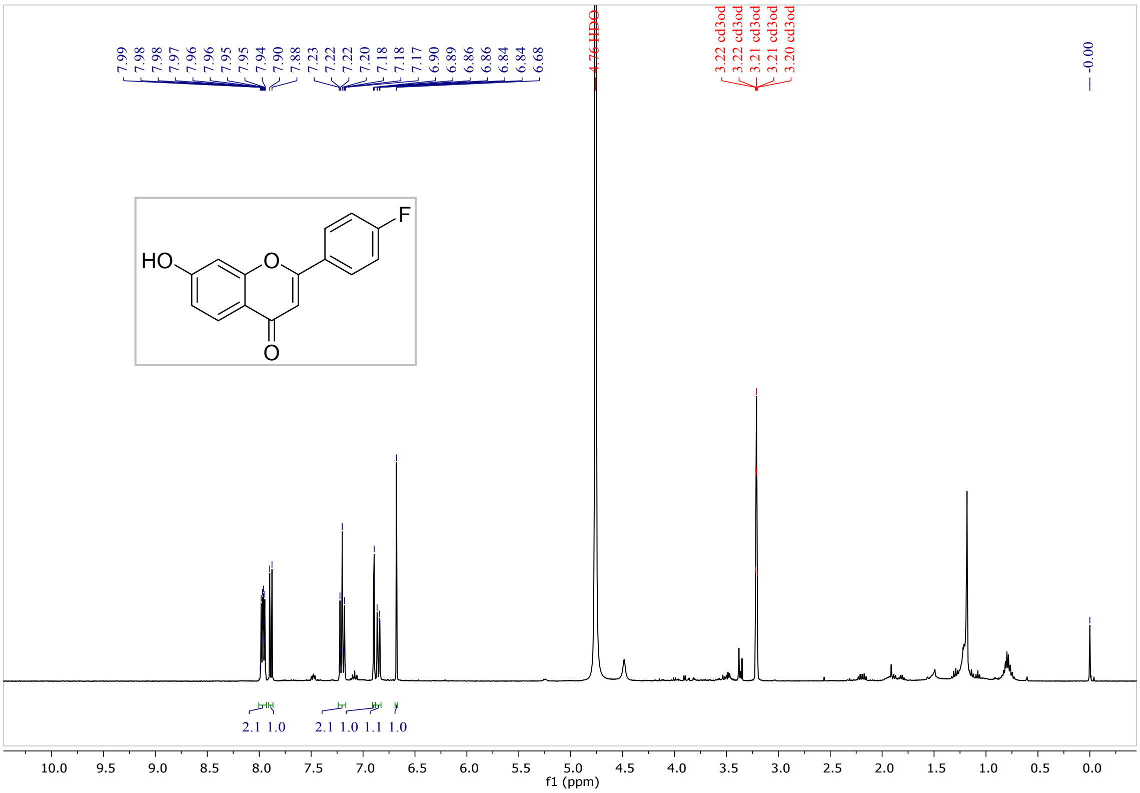

^{1}H NMR (500 MHz, $CDCl_3$) Spectra of 4-oxo-2-phenyl-4H-chromen-7-yl diethylcarbamate (**10a**)

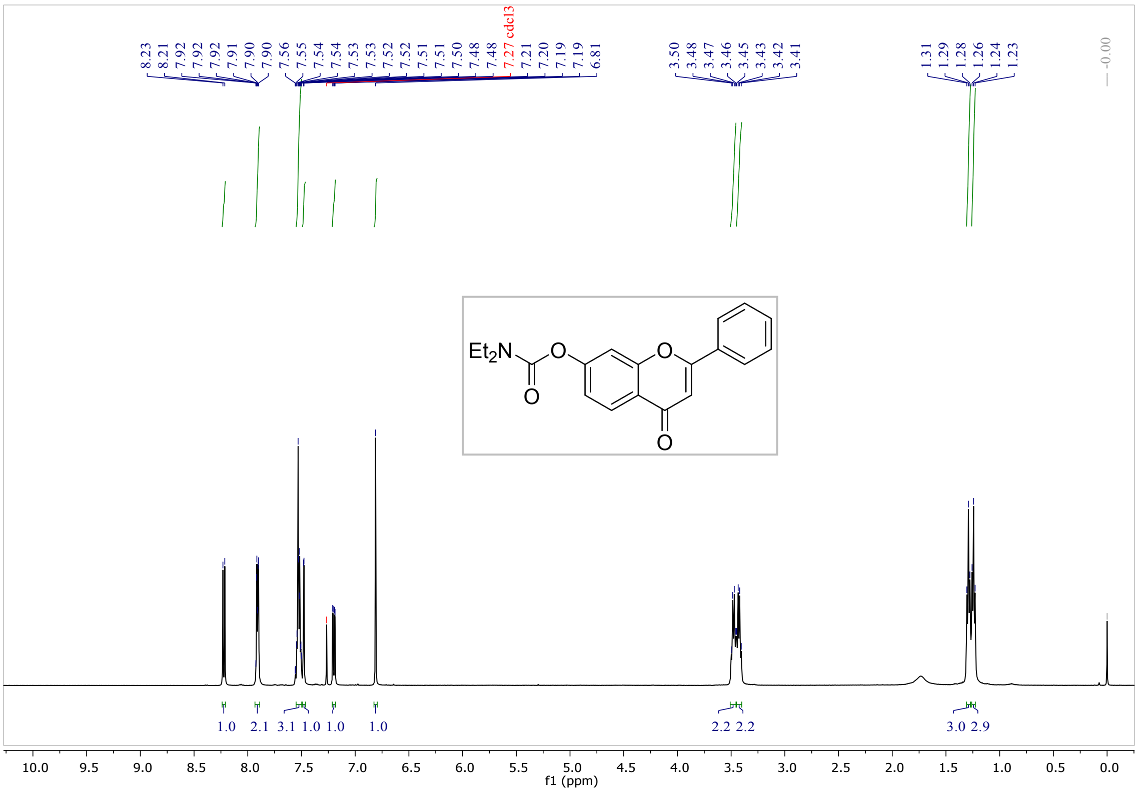

$^{13}C\{^1H\}$ NMR (126 MHz, $CDCl_3$) Spectra of 4-oxo-2-phenyl-4H-chromen-7-yl diethylcarbamate (**10a**)

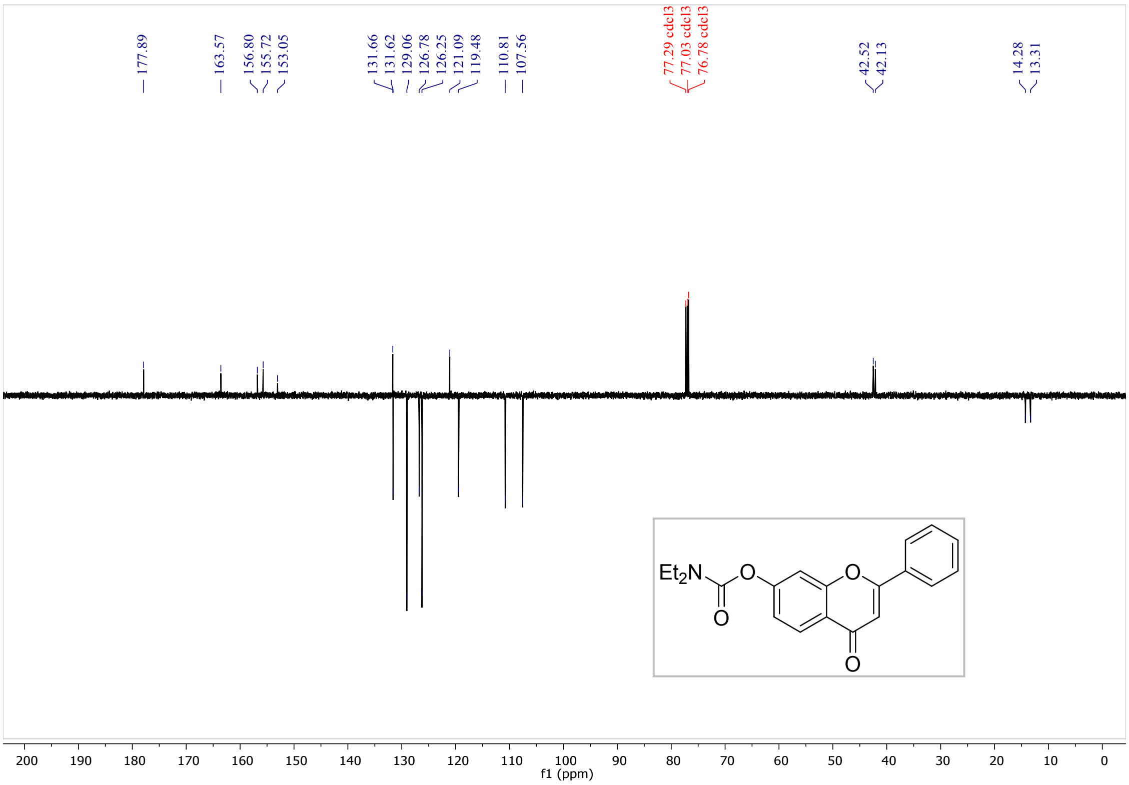

^1H NMR (500 MHz, $CDCl_3$) Spectra of 2-(4-fluorophenyl)-4-oxo-4H-chromen-7-yl diethylcarbamate (**10b**)

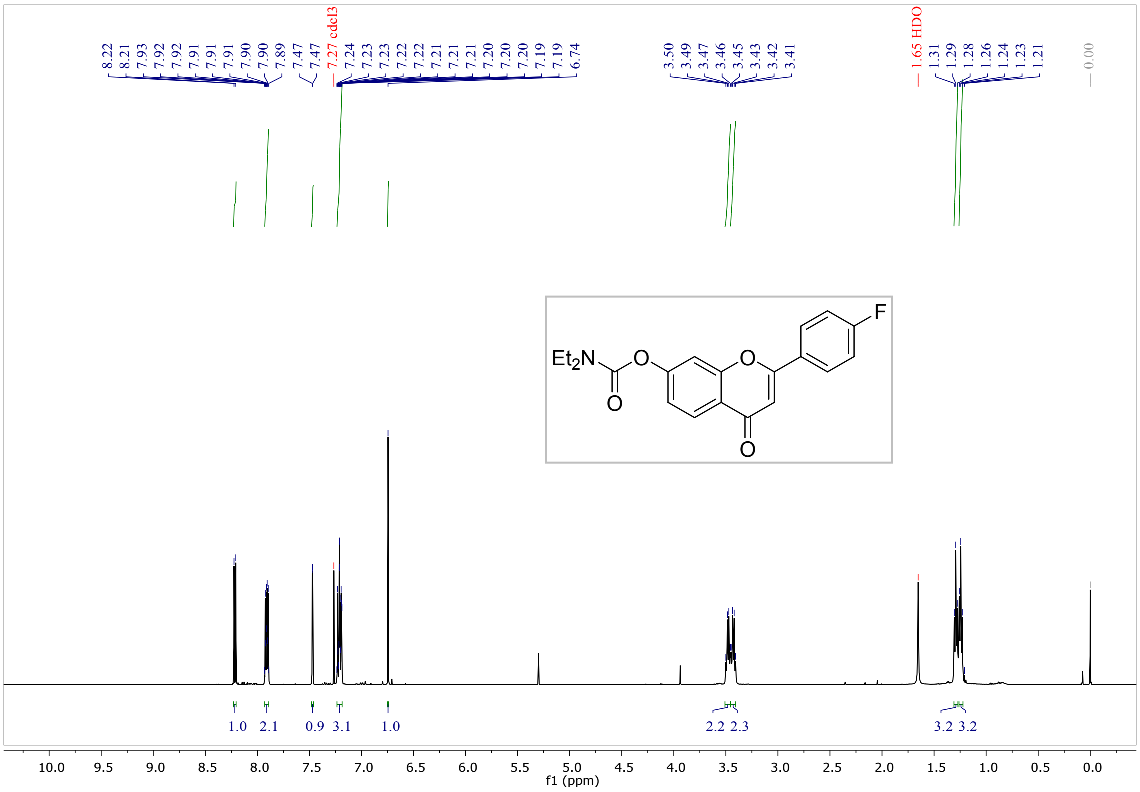

$^{13}C\{^1H\}$ NMR (101 MHz, $CDCl_3$) Spectra of 4-oxo-2-phenyl-4H-chromen-7-yl diethylcarbamate (**10b**)

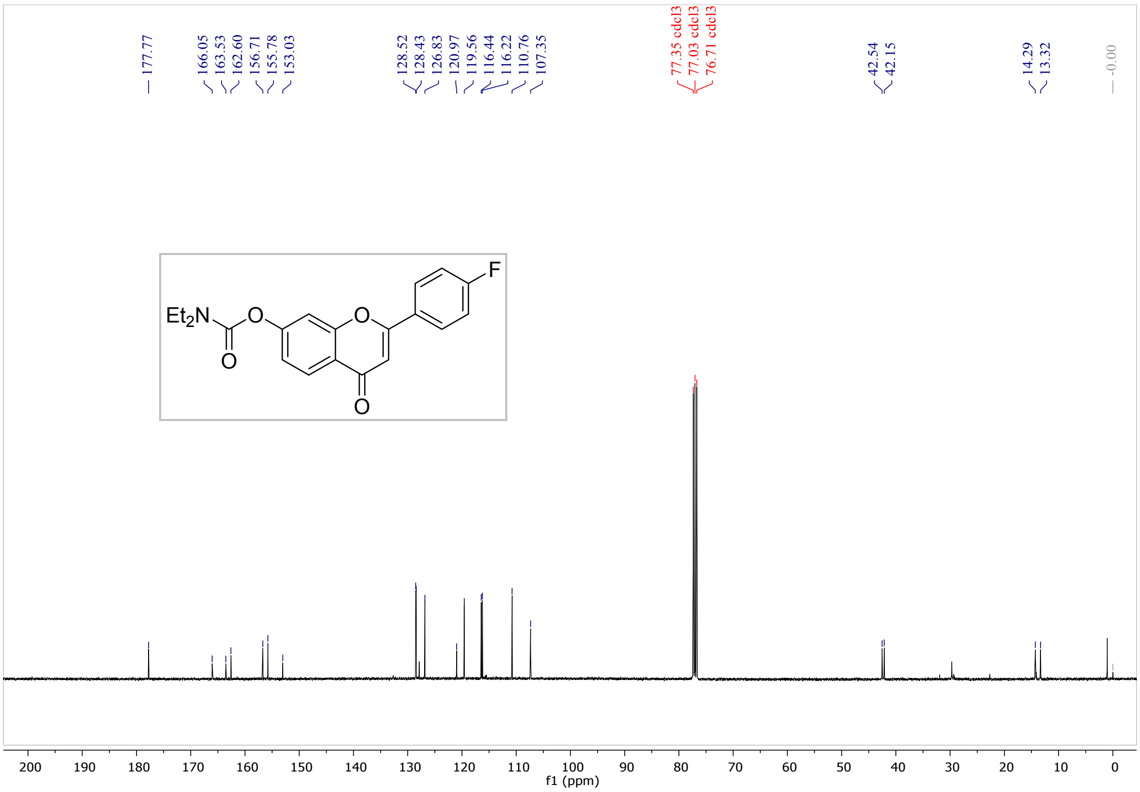

^1H NMR (500 MHz, $CDCl_3$) Spectra of 4-oxo-2-(4-(trifluoromethyl)phenyl)-4H-chromen-7-yl diethylcarbamate (**10c**)

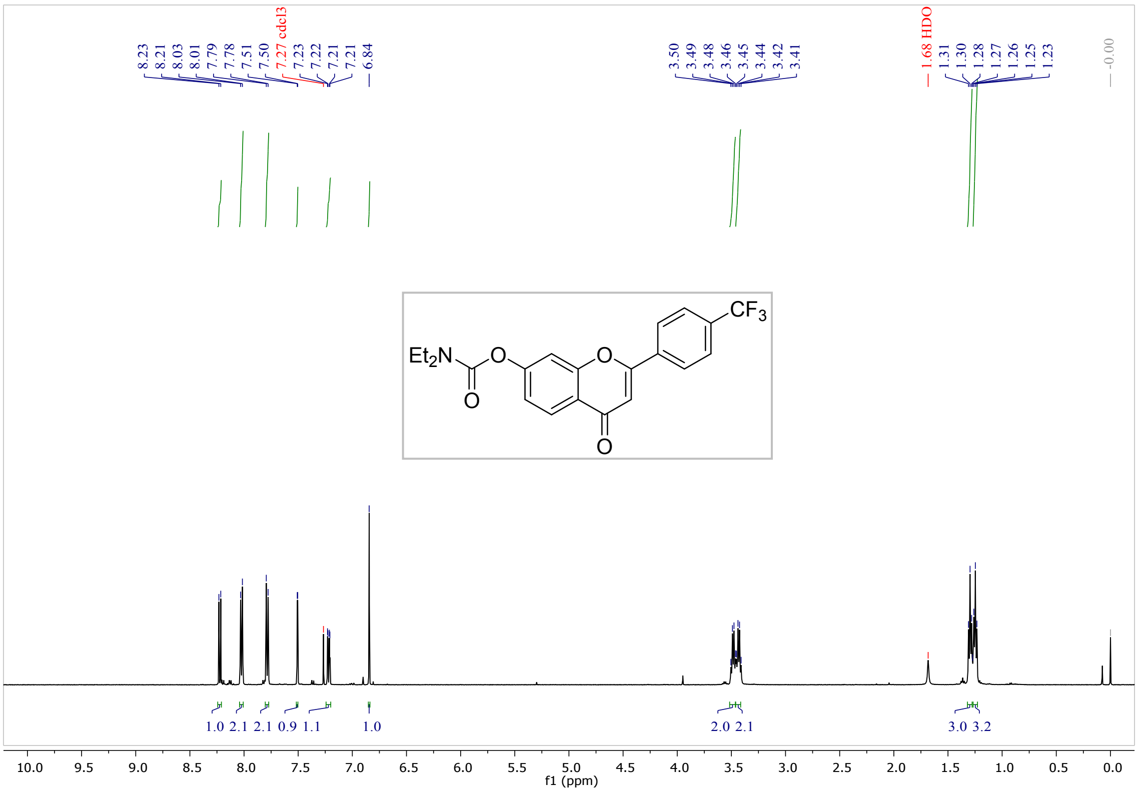

$^{13}C\{^1H\}$ NMR (126 MHz, $CDCl_3$) Spectra of 4-oxo-2-(4-(trifluoromethyl)phenyl)-4H-chromen-7-yl diethylcarbamate (**10c**)

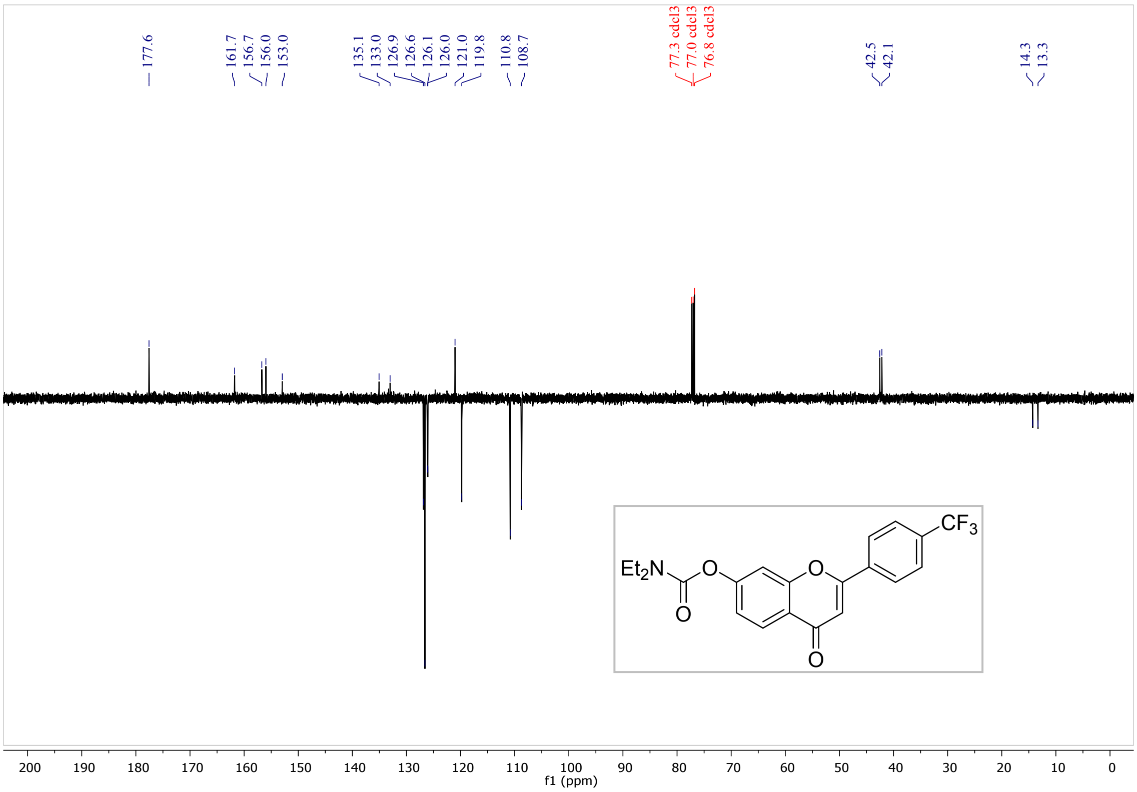

^1H NMR (500 MHz, $CDCl_3$) Spectra of 4-acetyl-3-hydroxyphenyl diethylcarbamate

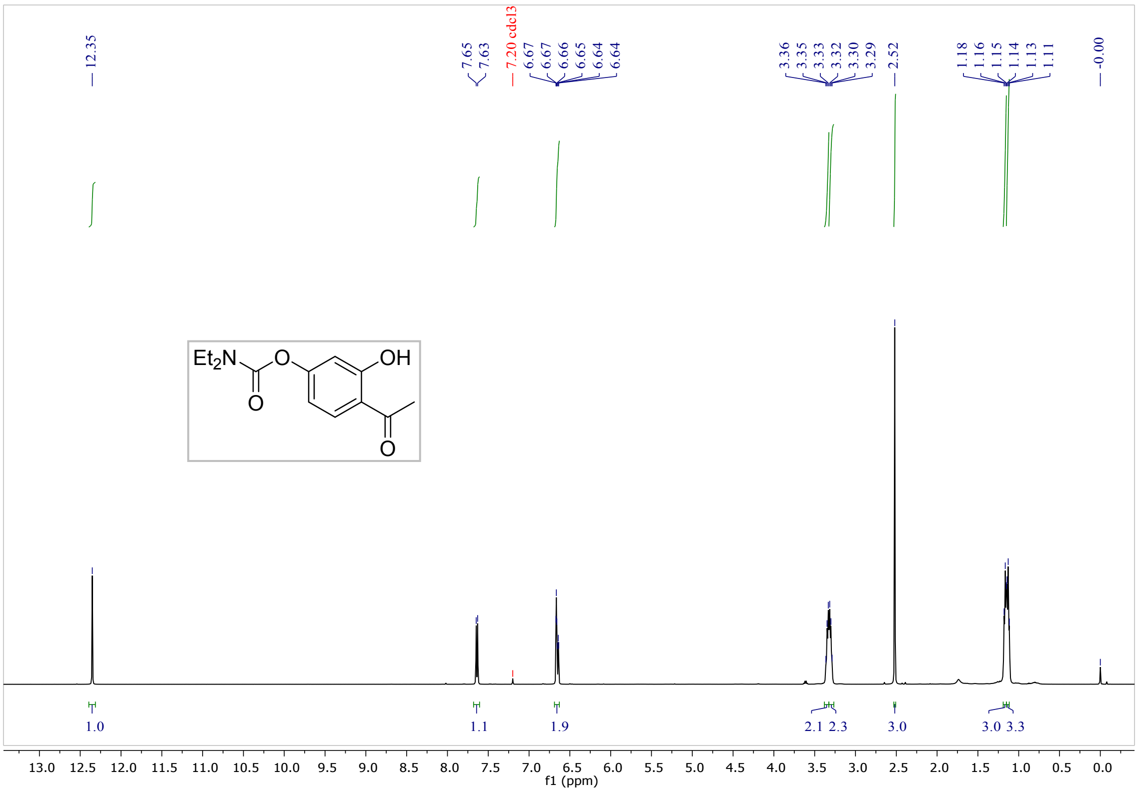

$^{13}C\{^1H\}$NMR(126 MHz, $CDCl_3$) Spectra of 4-acetyl-3-hydroxyphenyl diethylcarbamate

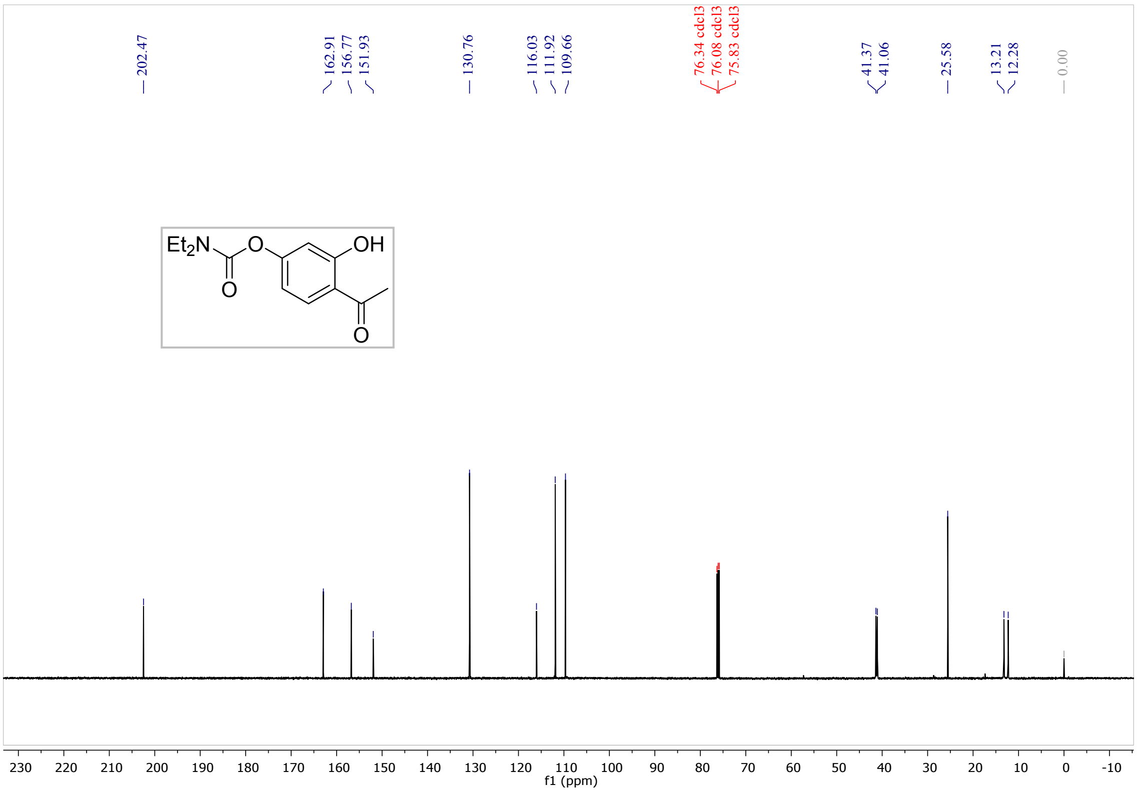

^{1}H NMR (500 MHz, $CDCl_3$) Spectra of 2-(4-methoxyphenyl)-4-oxo-4H-chromen-7-yl diethylcarbamate (**10d**)

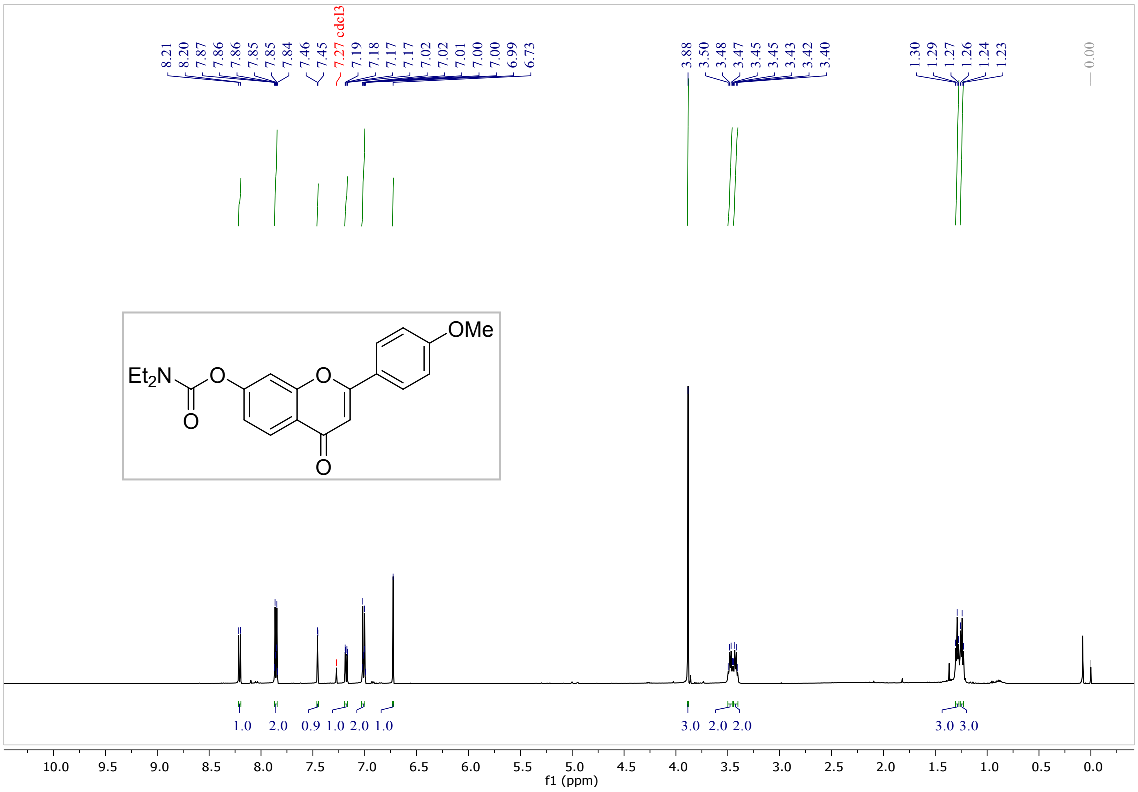

^{13}C{^{1}H} NMR (126 MHz, $CDCl_3$) Spectra of 2-(4-methoxyphenyl)-4-oxo-4H-chromen-7-yl diethylcarbamate (**10d**)

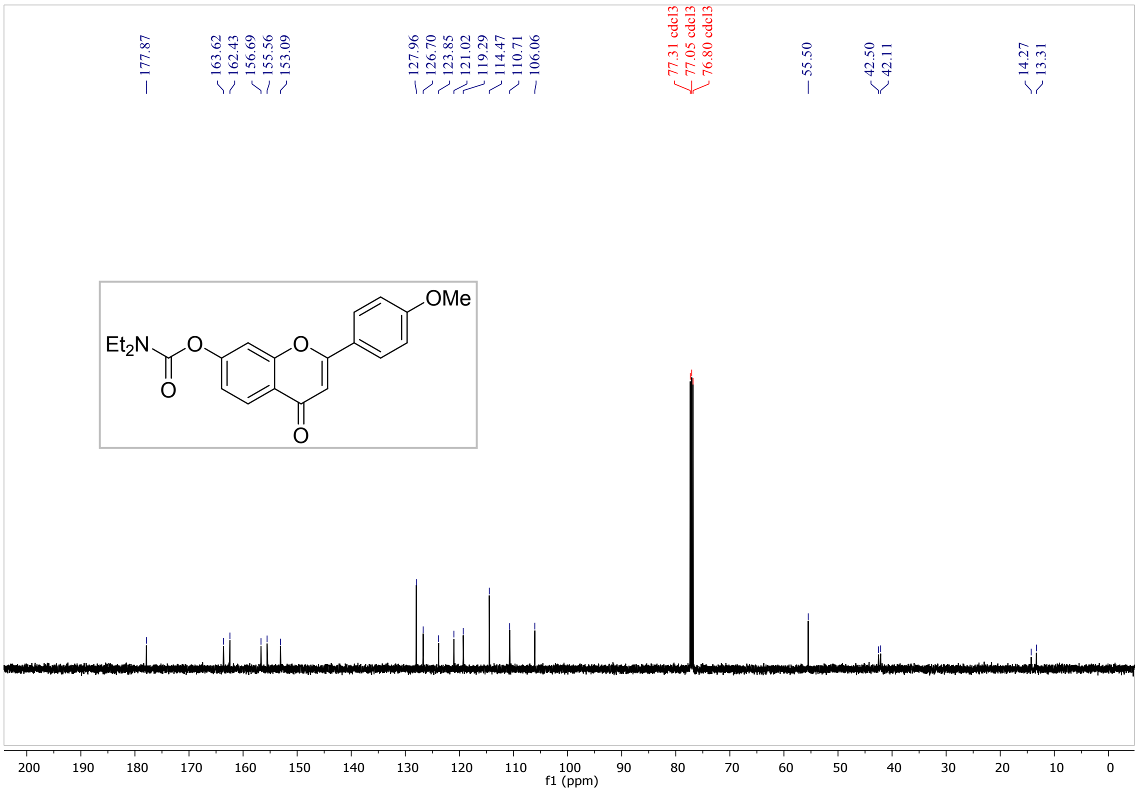

^{1}H NMR (400 MHz, $CDCl_3$) Spectra of 2-(3,4-dimethoxyphenyl)-4-oxo-4H-chromen-7-yl diethylcarbamate (**10e**)

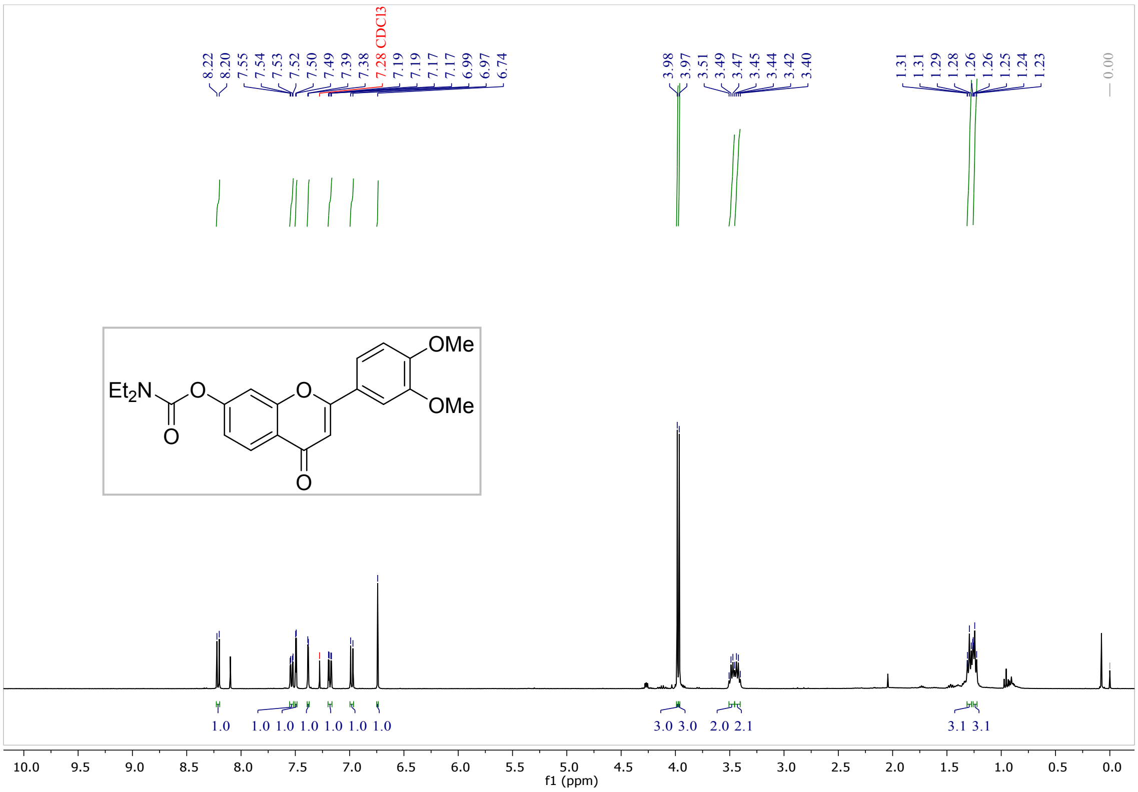

$^{13}C\{^1H\}$ NMR (126 MHz, $CDCl_3$) Spectra of 2-(3,4-dimethoxyphenyl)-4-oxo-4H-chromen-7-yl diethylcarbamate (**10e**)

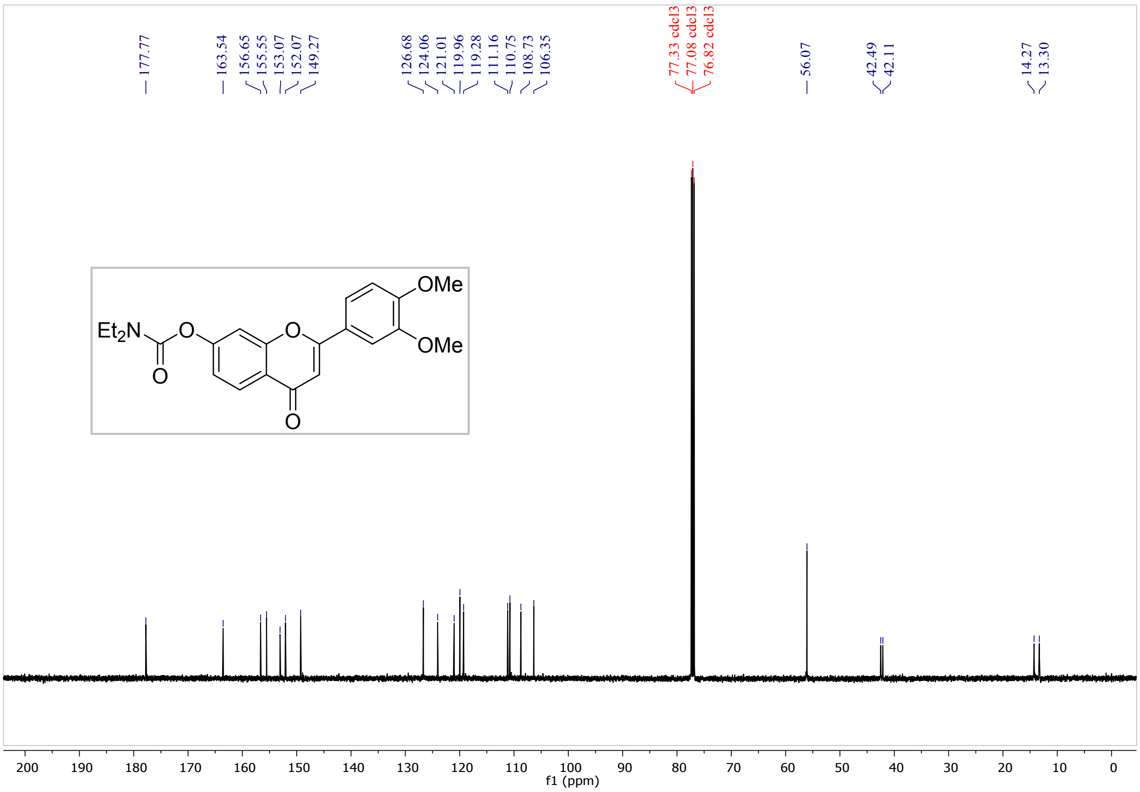

^1H NMR (500 MHz, $CDCl_3$) Spectra of Compound **9a**

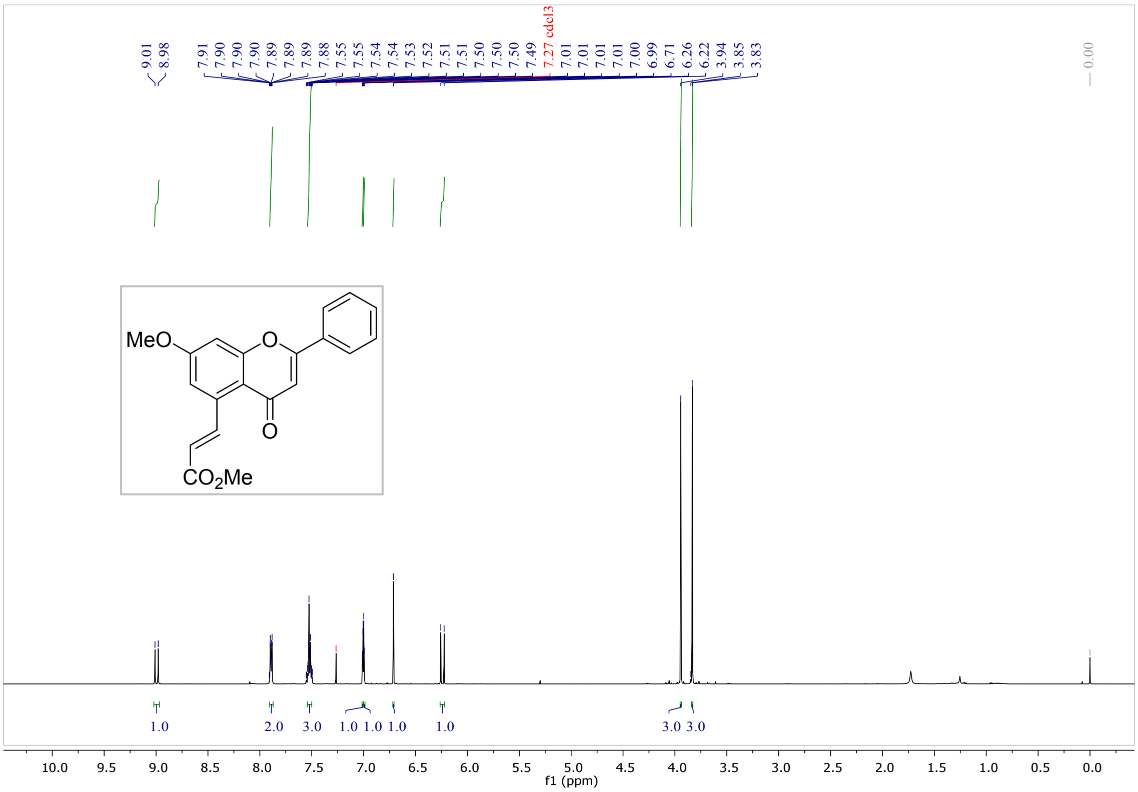

$^{13}C\{^1H\}$ NMR (126 MHz, $CDCl_3$) Spectra of Compound **9a**

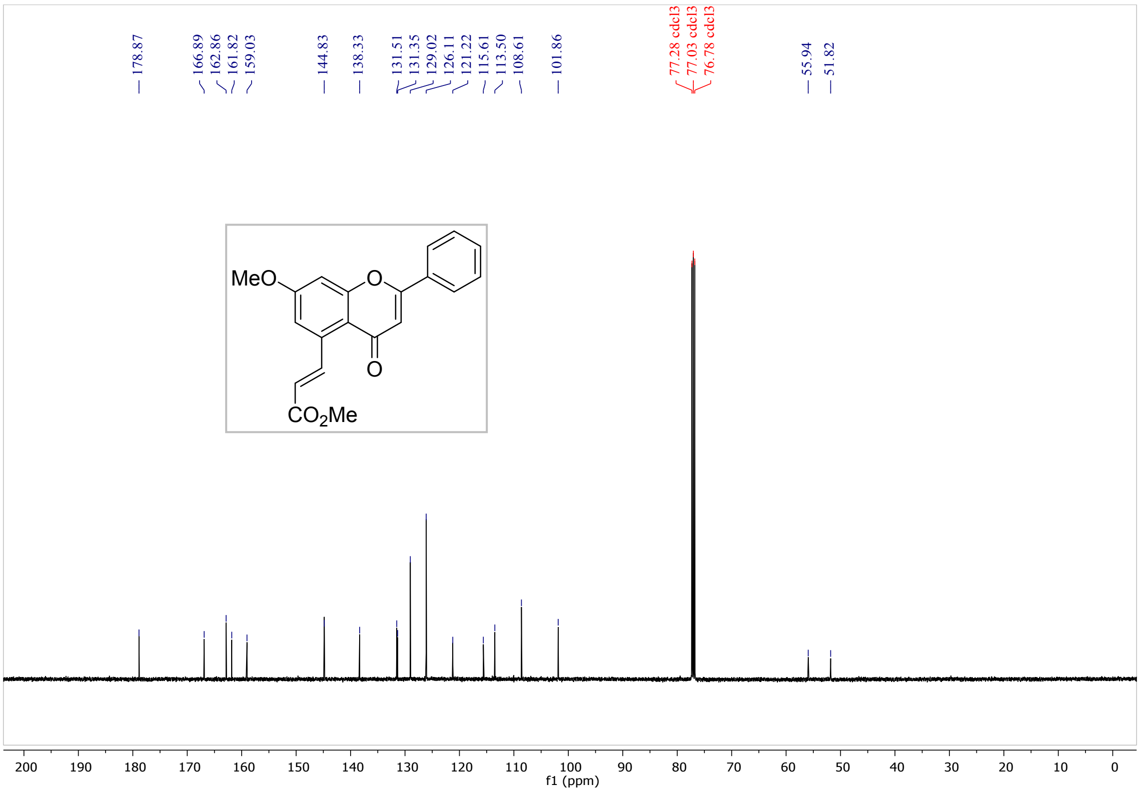

^{1}H NMR (400 MHz, $CDCl_3$) Spectra of Compound **9b**

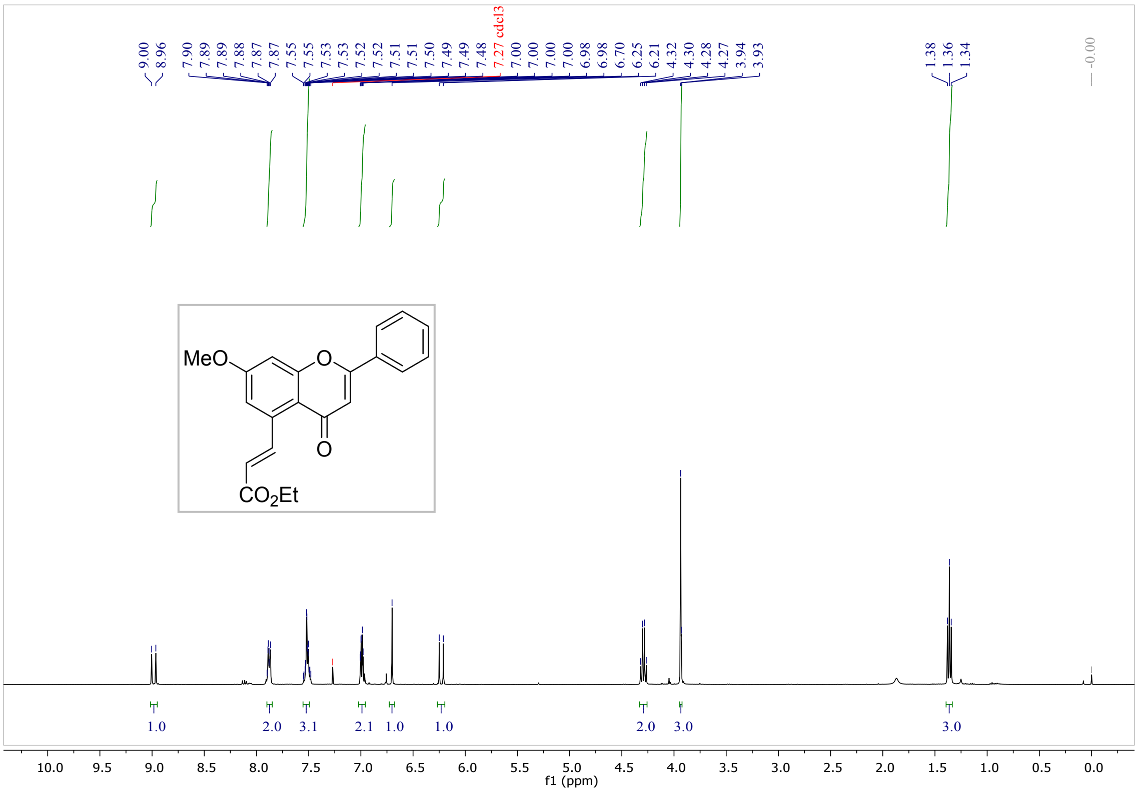

^{13}C{^{1}H} NMR (126 MHz, $CDCl_3$) Spectra of Compound **9b**

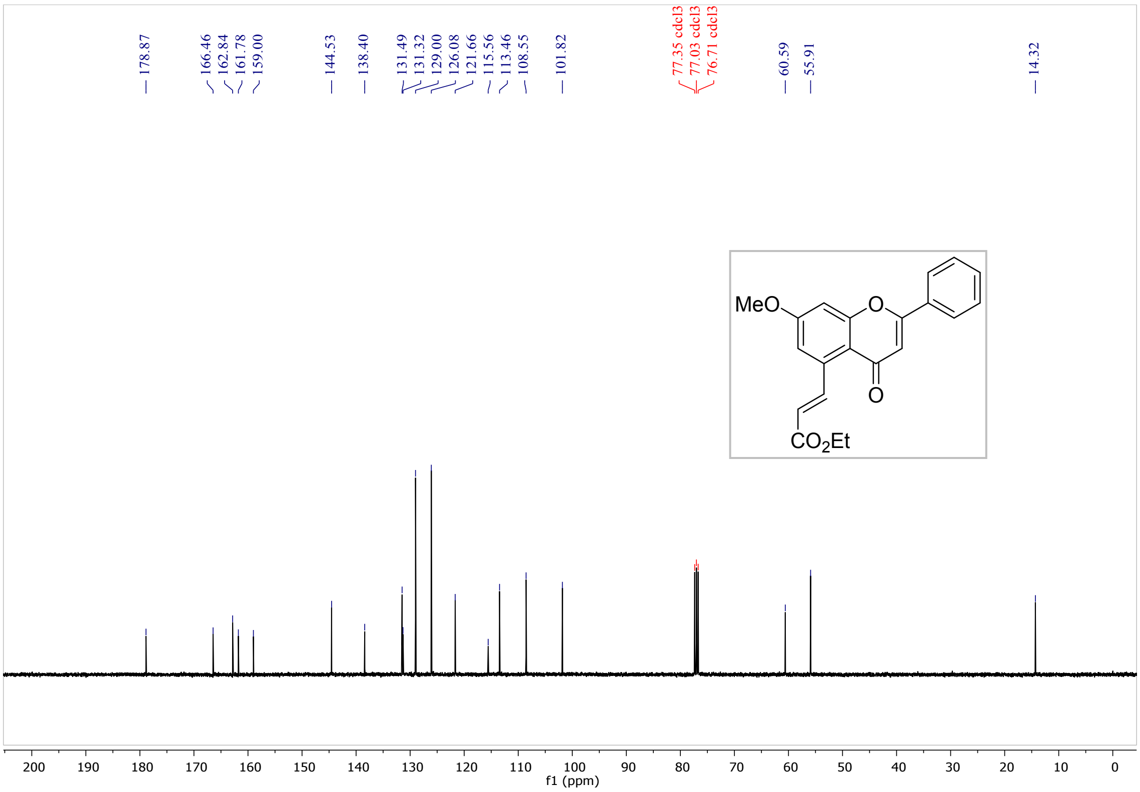

^{1}H NMR (500 MHz, $CDCl_3$) Spectra of Compound **9c**

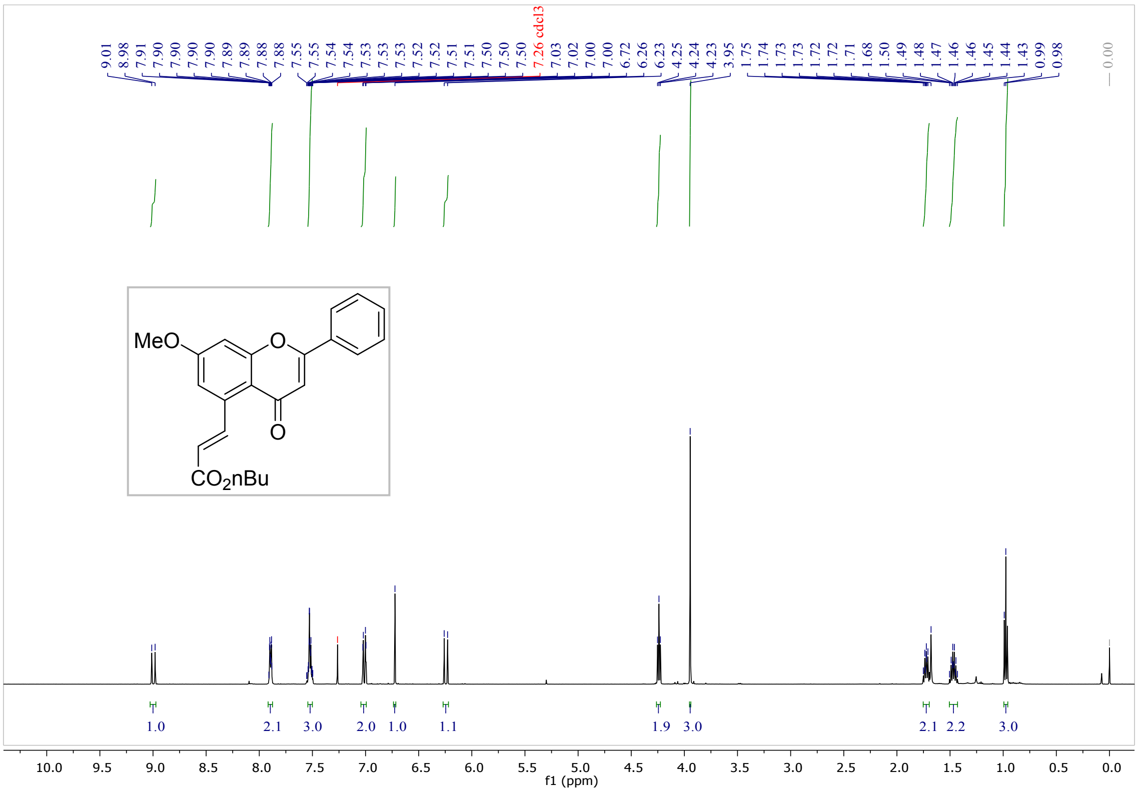

$^{13}C\{^{1}H\}$ NMR (126 MHz, $CDCl_3$) Spectra of Compound **9c**

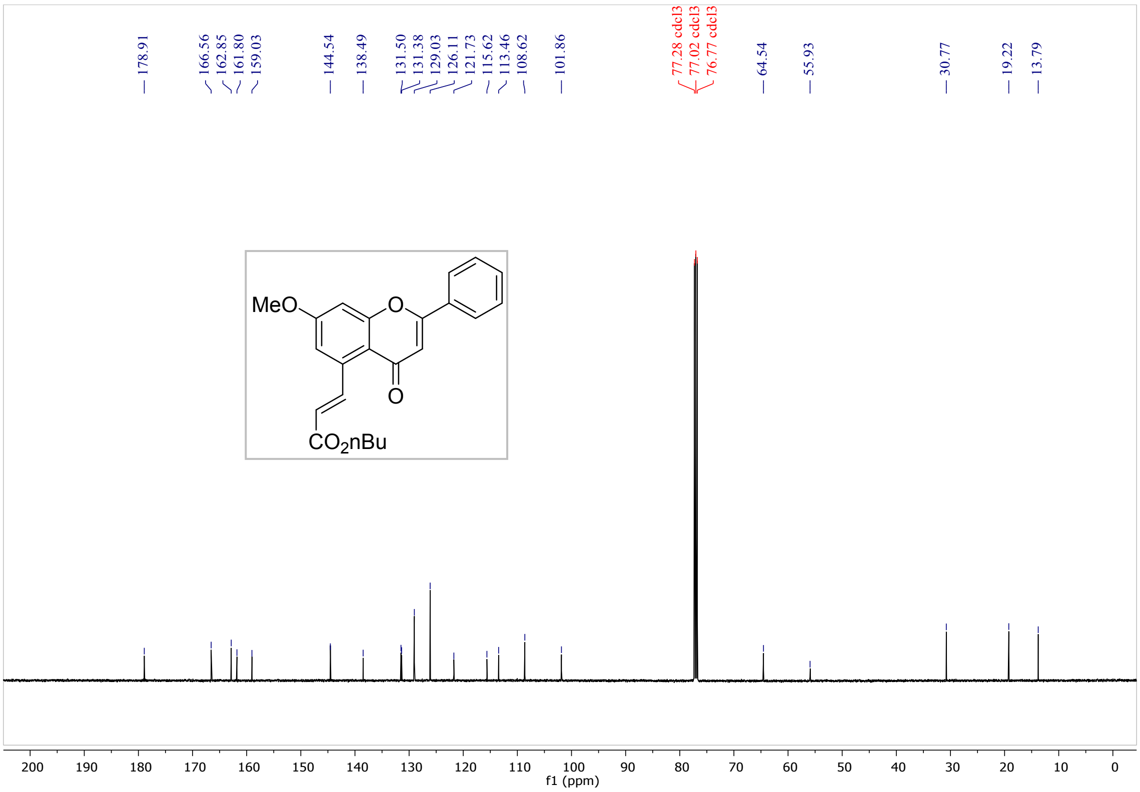

^{1}H NMR (400 MHz, $CDCl_3$) Spectra of Compound **9e**

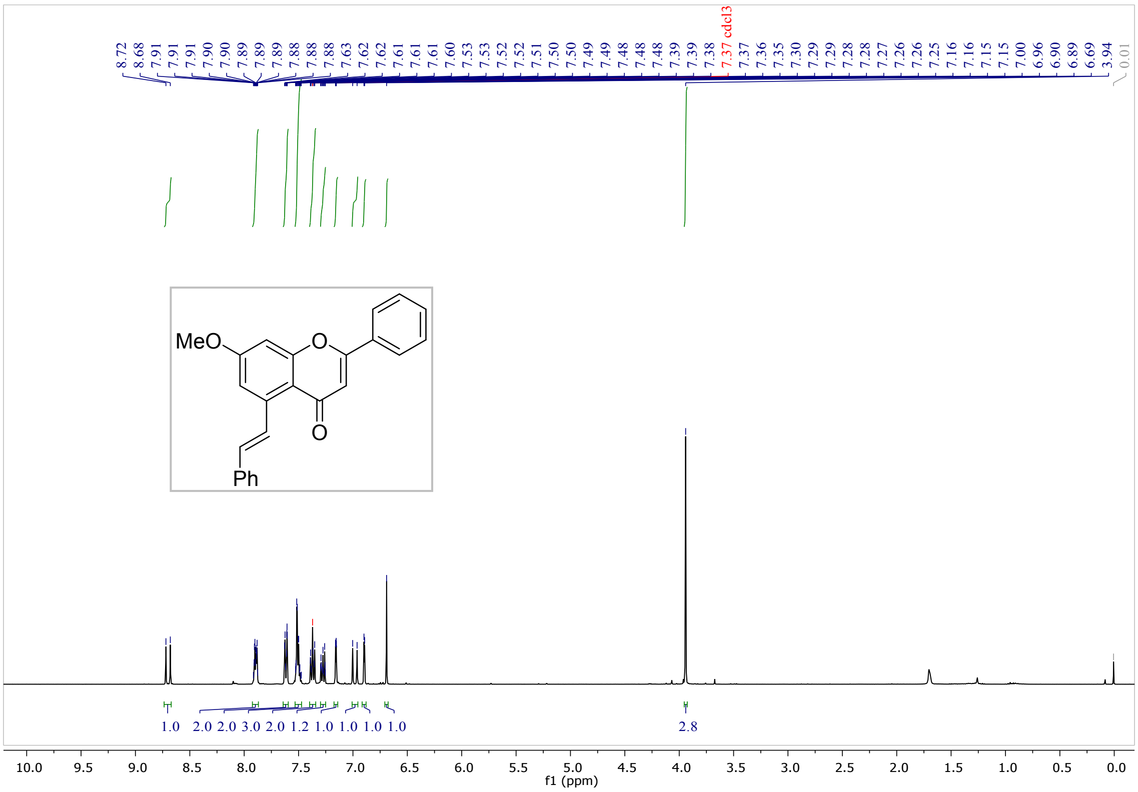

^{13}C{^{1}H} NMR (101 MHz, $CDCl_3$) Spectra of Compound **9e**

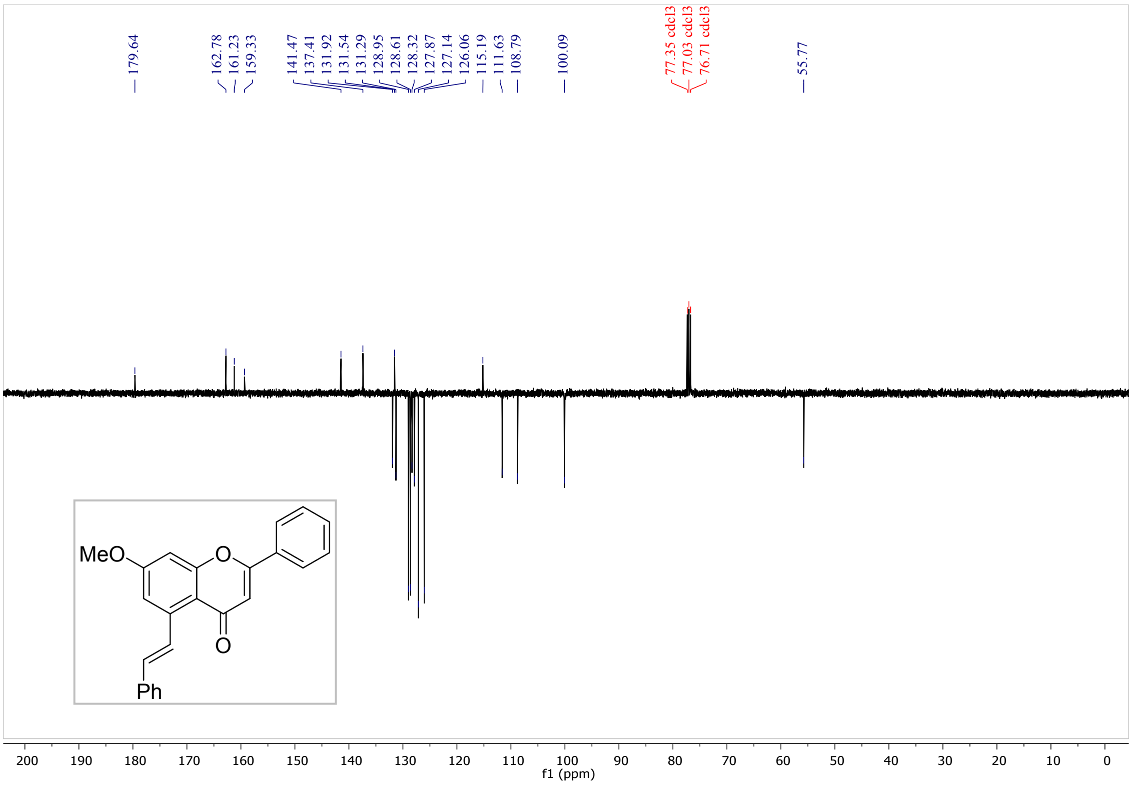

^{1}H NMR (400 MHz, $CDCl_3$) Spectra of Compound **9f**

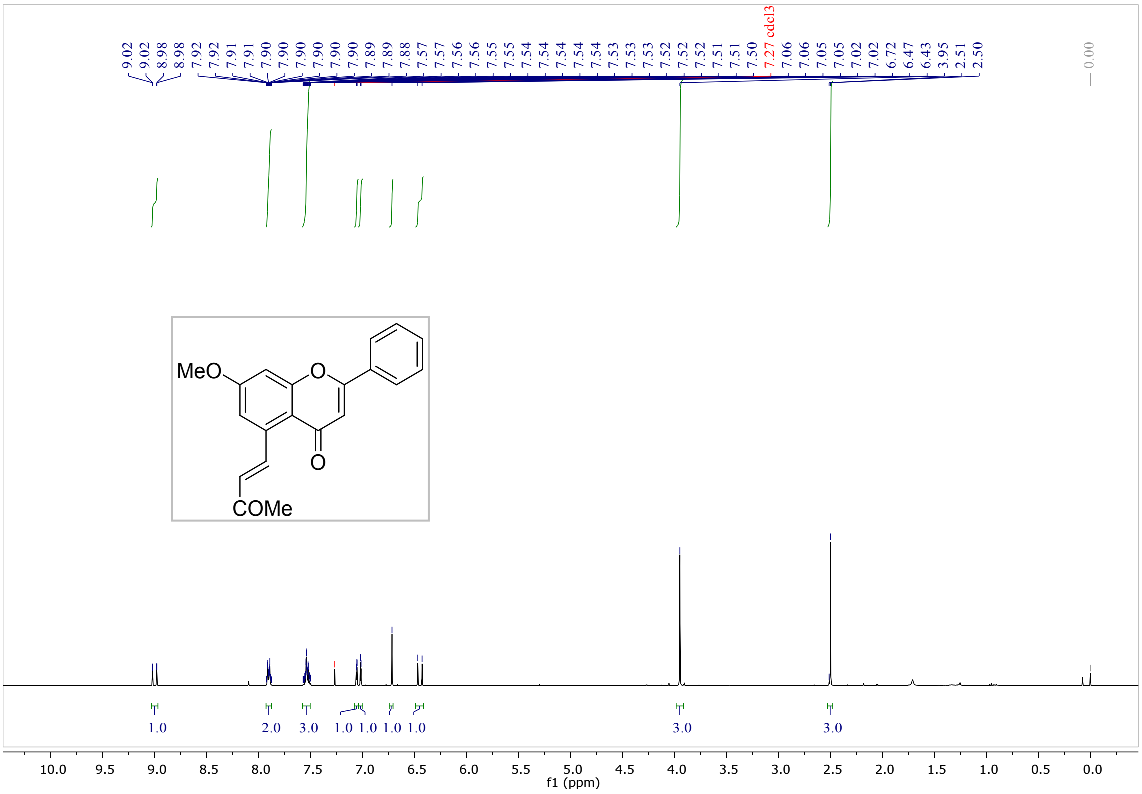

^{13}C{^{1}H} NMR (101 MHz, $CDCl_3$) Spectra of Compound **9f**

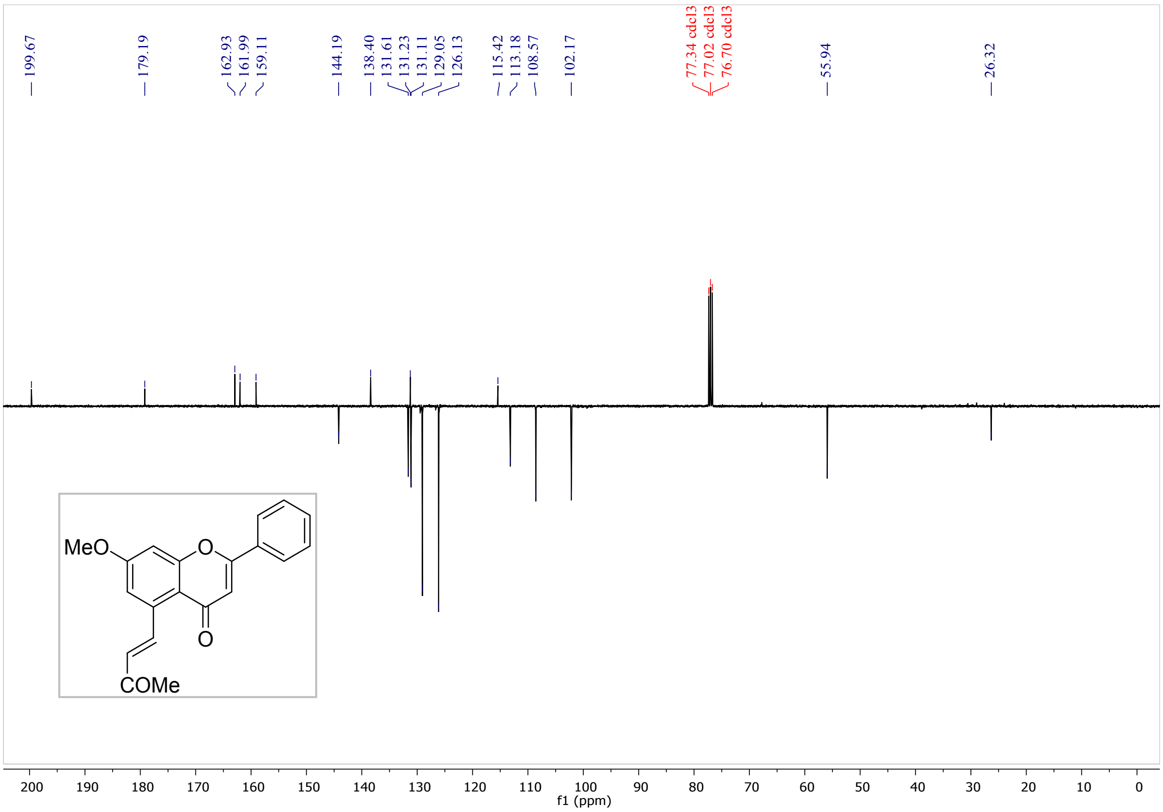

^{1}H NMR (400 MHz, $CDCl_3$) Spectra of Compound **9g**

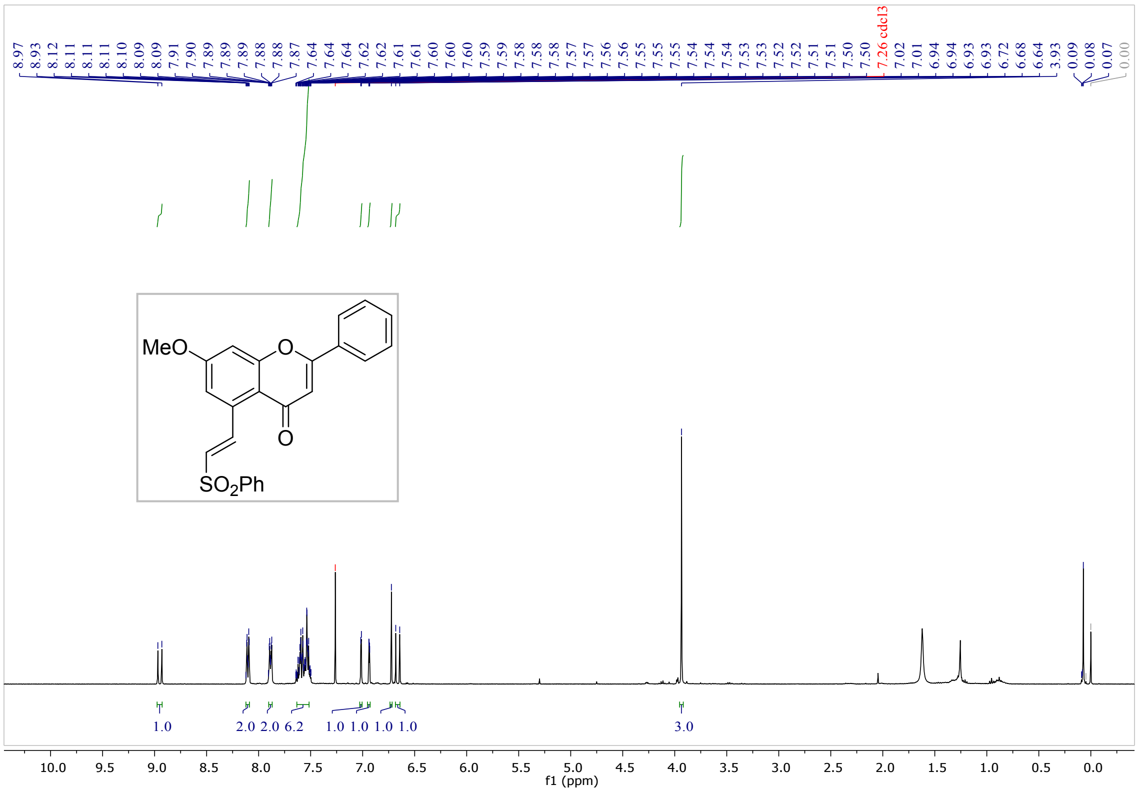

$^{13}C\{^{1}H\}$ NMR (101 MHz, $CDCl_3$) Spectra of Compound **9g**

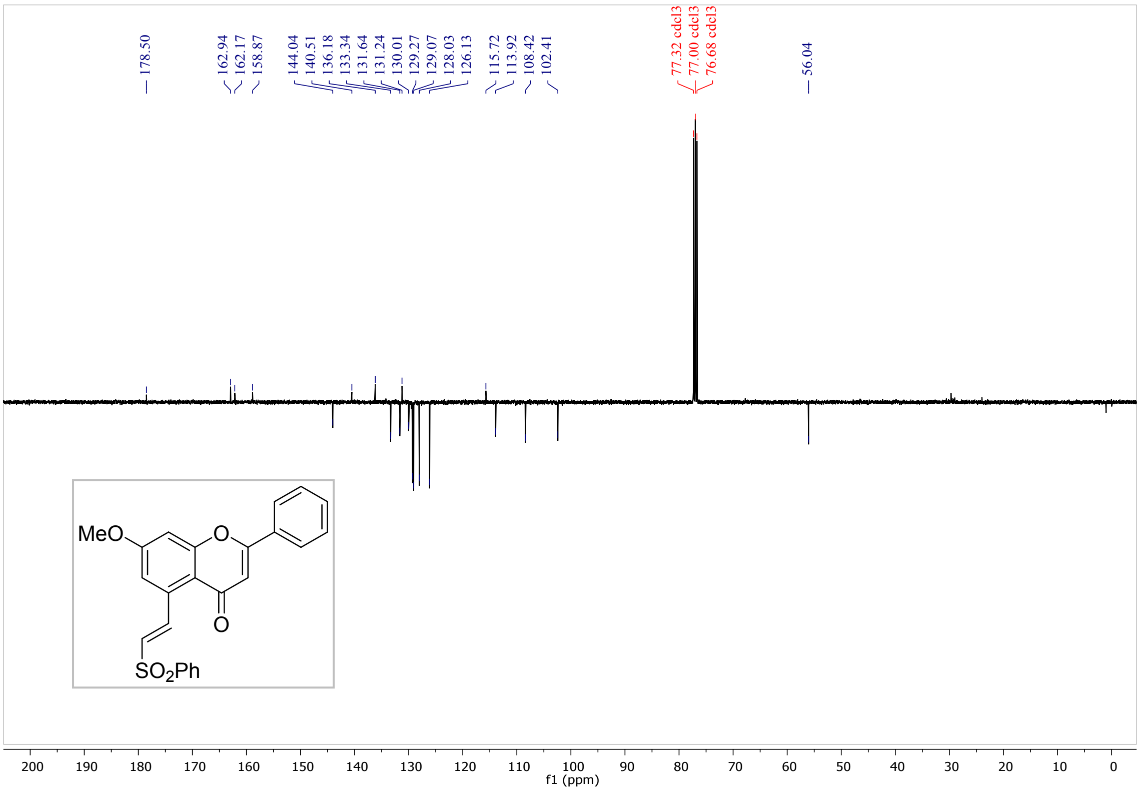

^{1}H NMR (400 MHz, $CDCl_3$) Spectra of Compound **9k**

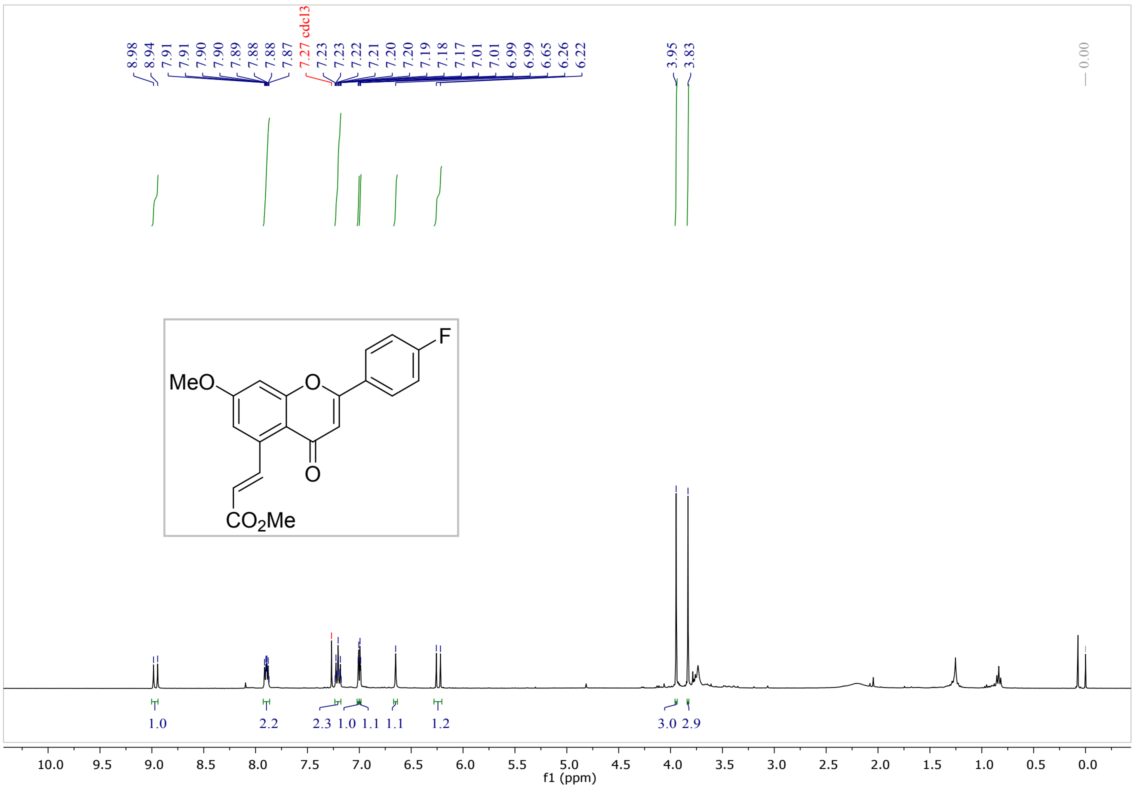

$^{13}C\{^1H\}$ NMR (126 MHz, $CDCl_3$) Spectra of Compound **9k**

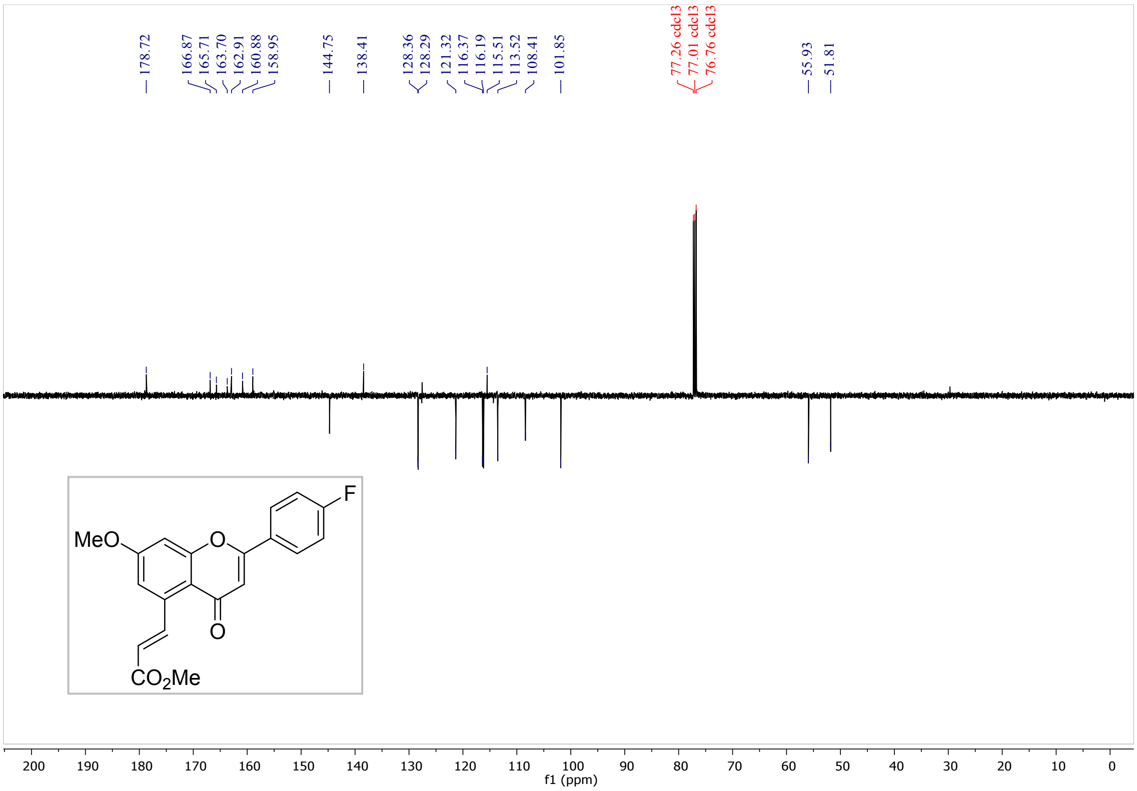

^1H NMR (500 MHz, $CDCl_3$) Spectra of Compound **9l**

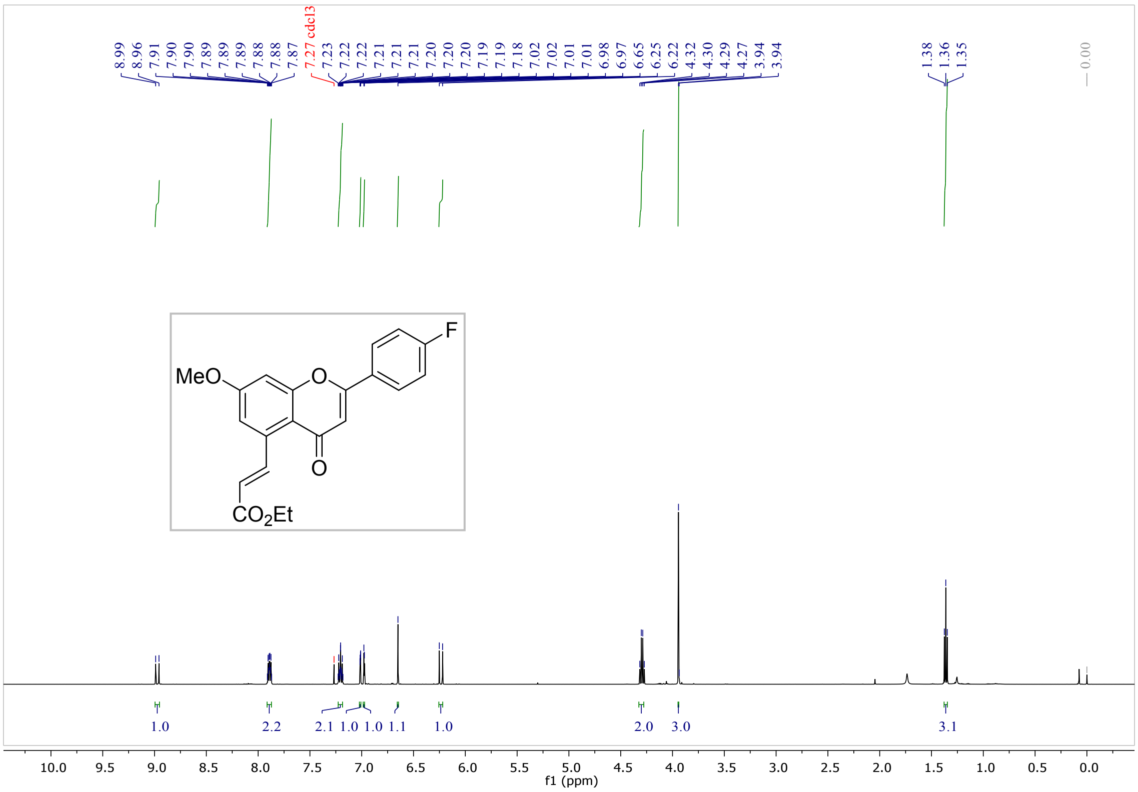

$^{13}C\{^{1}H\}$ NMR (126 MHz, $CDCl_3$) Spectra of Compound **9l**

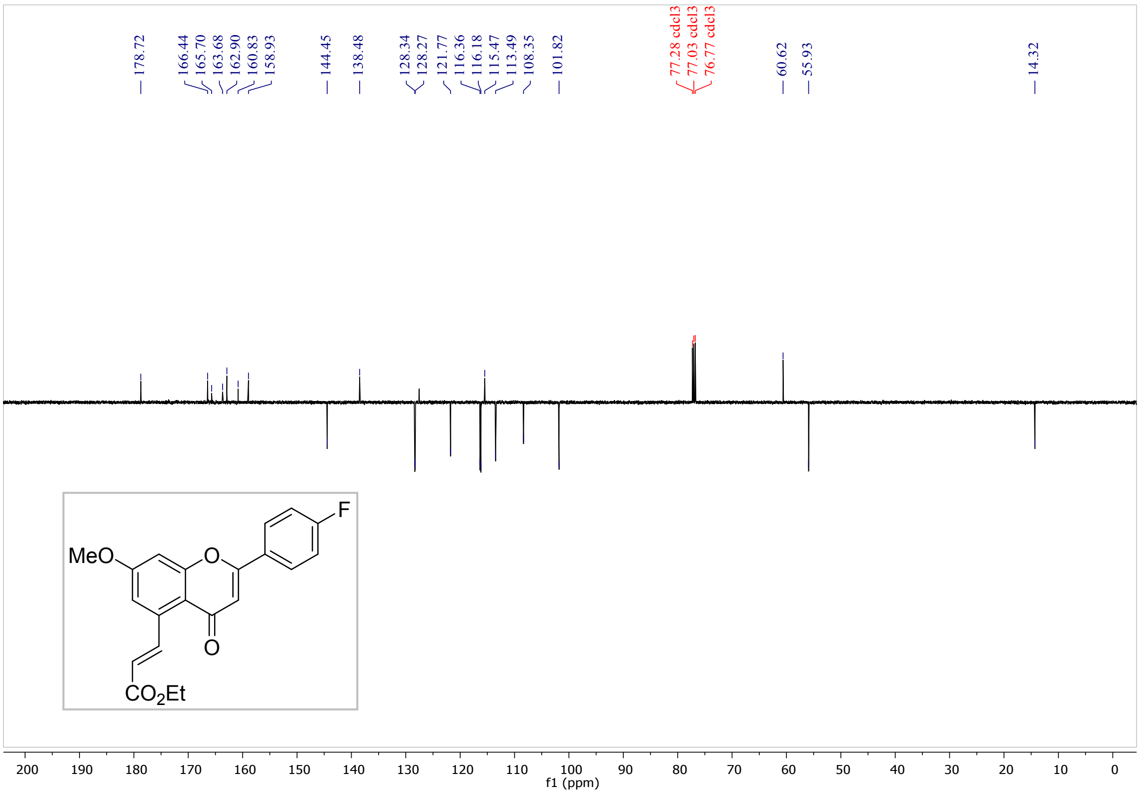

^{1}H NMR (500 MHz, $CDCl_3$) Spectra of Compound **9m**

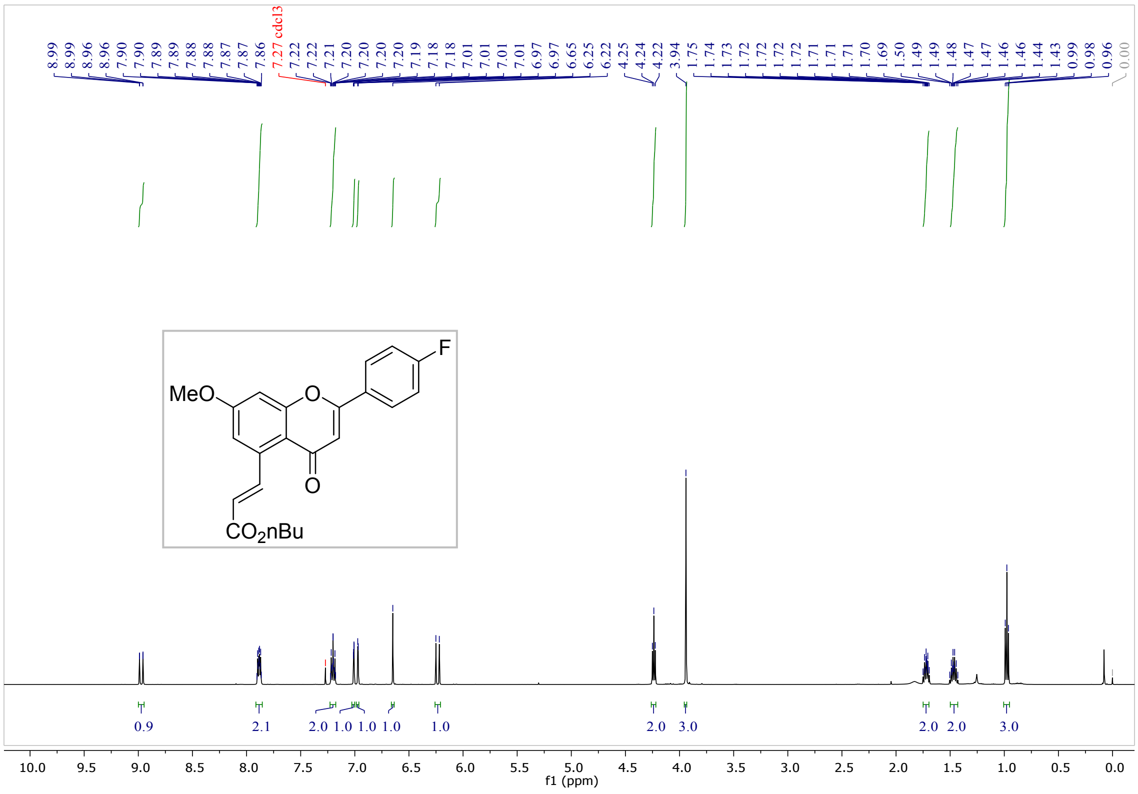

$^{13}C\{^{1}H\}$ NMR (126 MHz, $CDCl_3$) Spectra of Compound **9m**

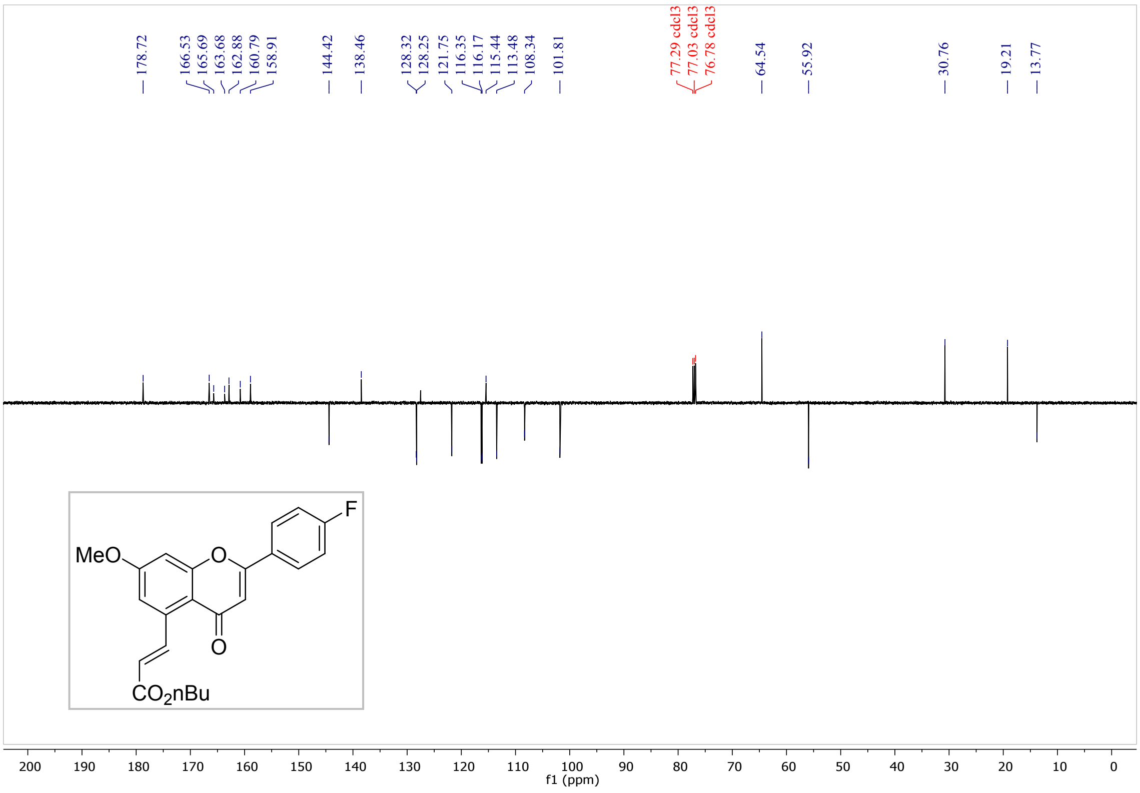

^{1}H NMR (500 MHz, $CDCl_3$) Spectra of Compound **9n**

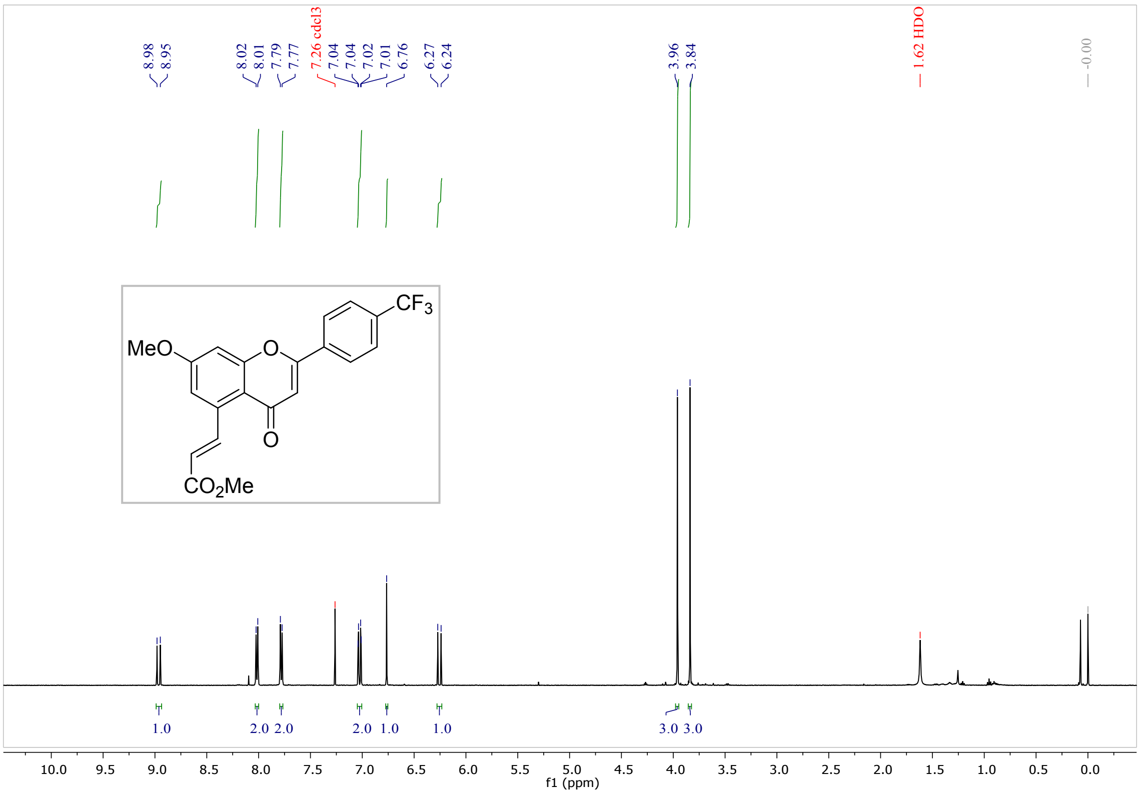

$^{13}C\{^{1}H\}$ NMR (126 MHz, $CDCl_3$) Spectra of Compound **9n**

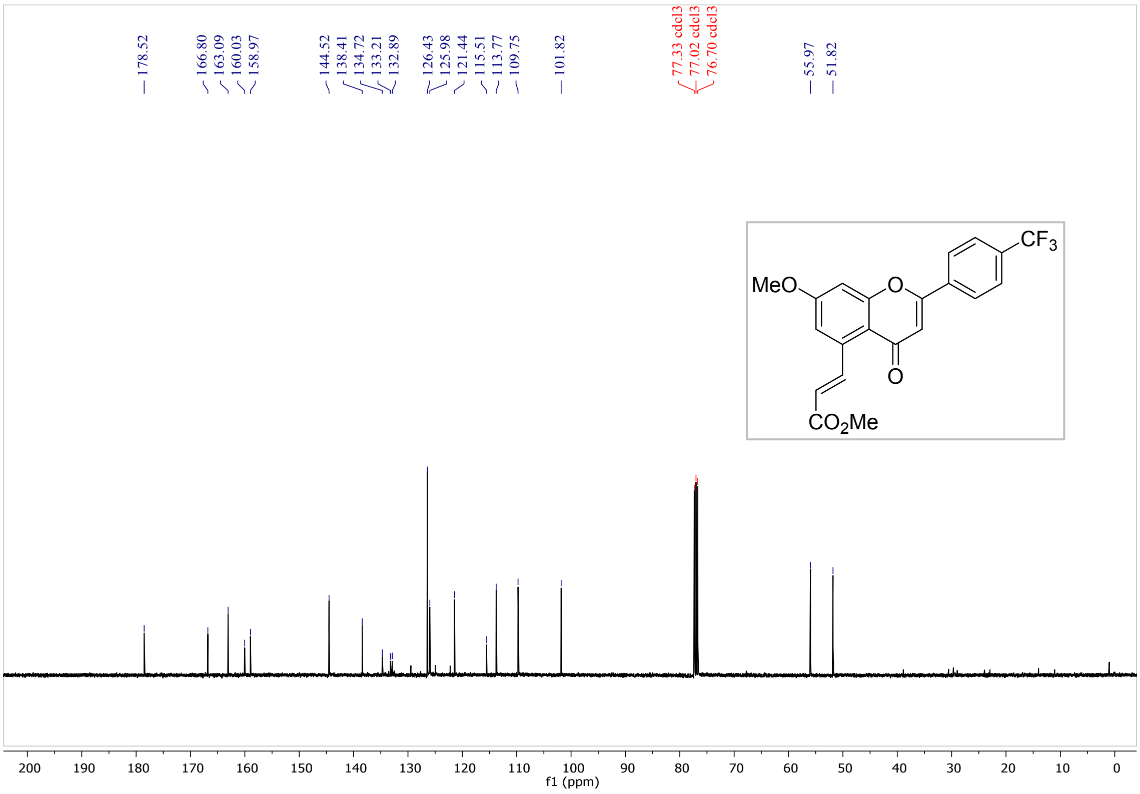

^{1}H NMR (500 MHz, $CDCl_3$) Spectra of Compound **9o**

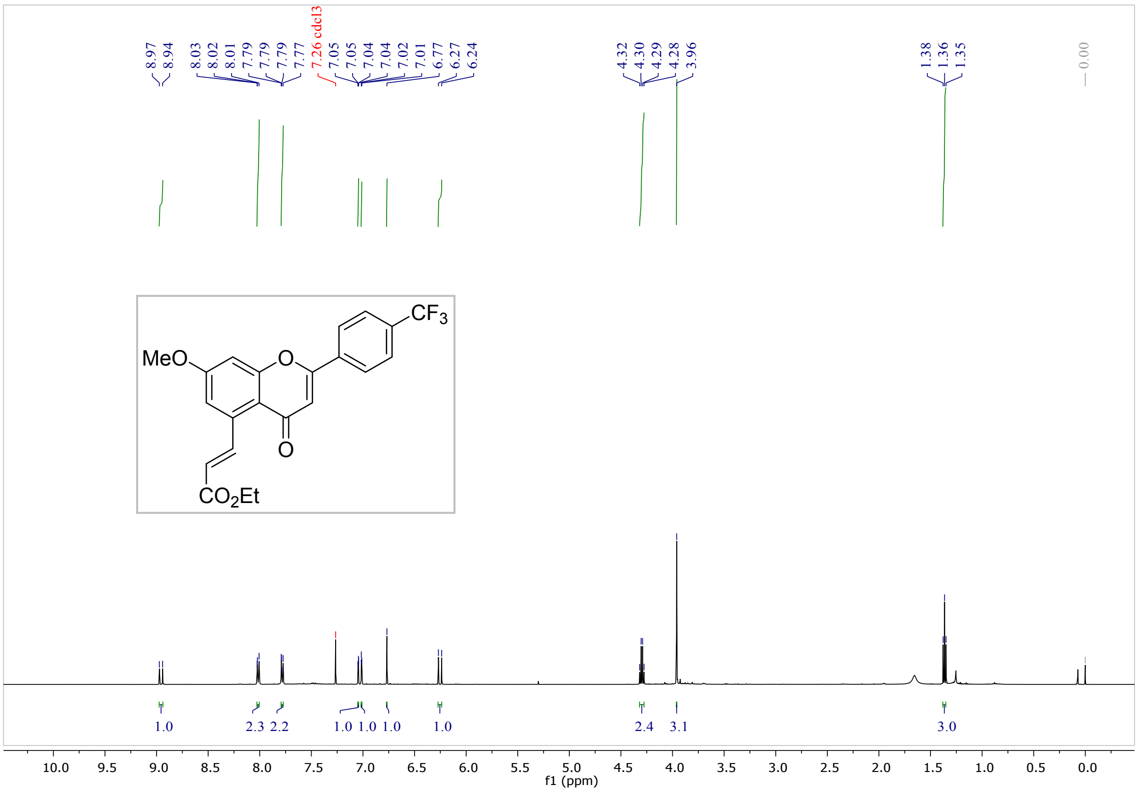

$^{13}C\{^1H\}$ NMR (126 MHz, $CDCl_3$) Spectra of Compound **9o**

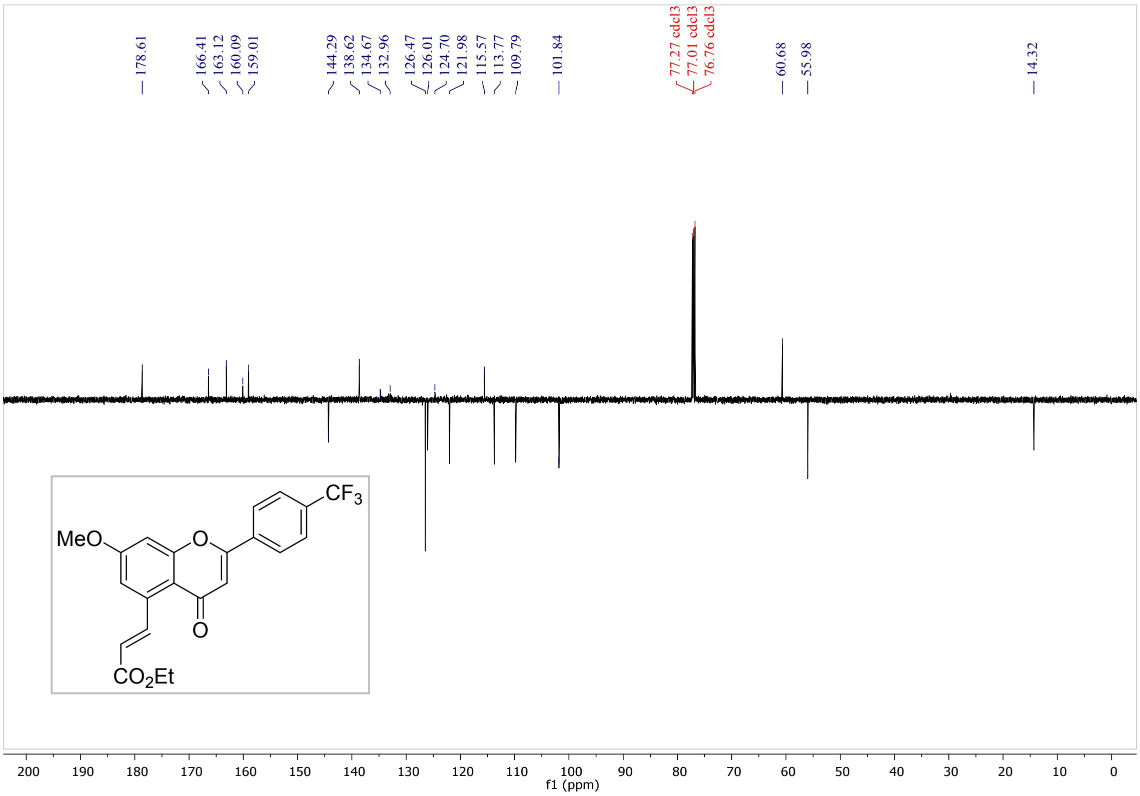

^1H NMR (500 MHz, $CDCl_3$) Spectra of Compound **9p**

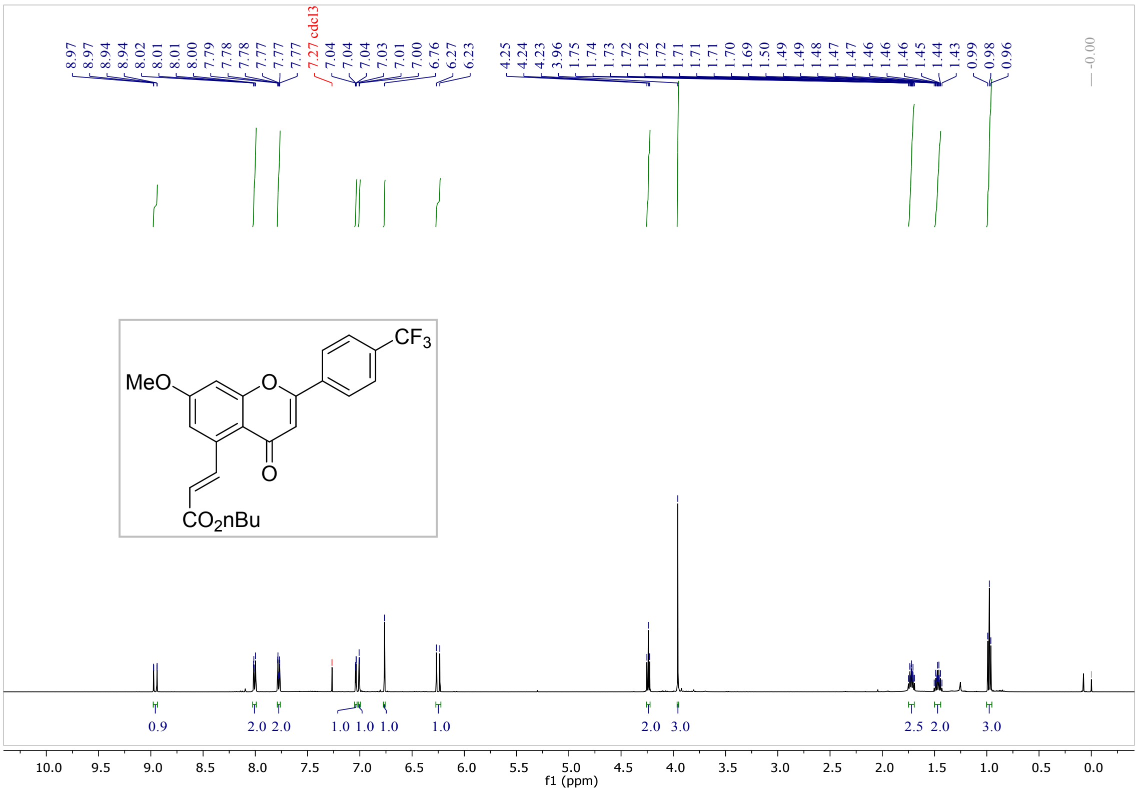

$^{13}C\{^{1}H\}$ NMR (126 MHz, $CDCl_3$) Spectra of Compound **9p**

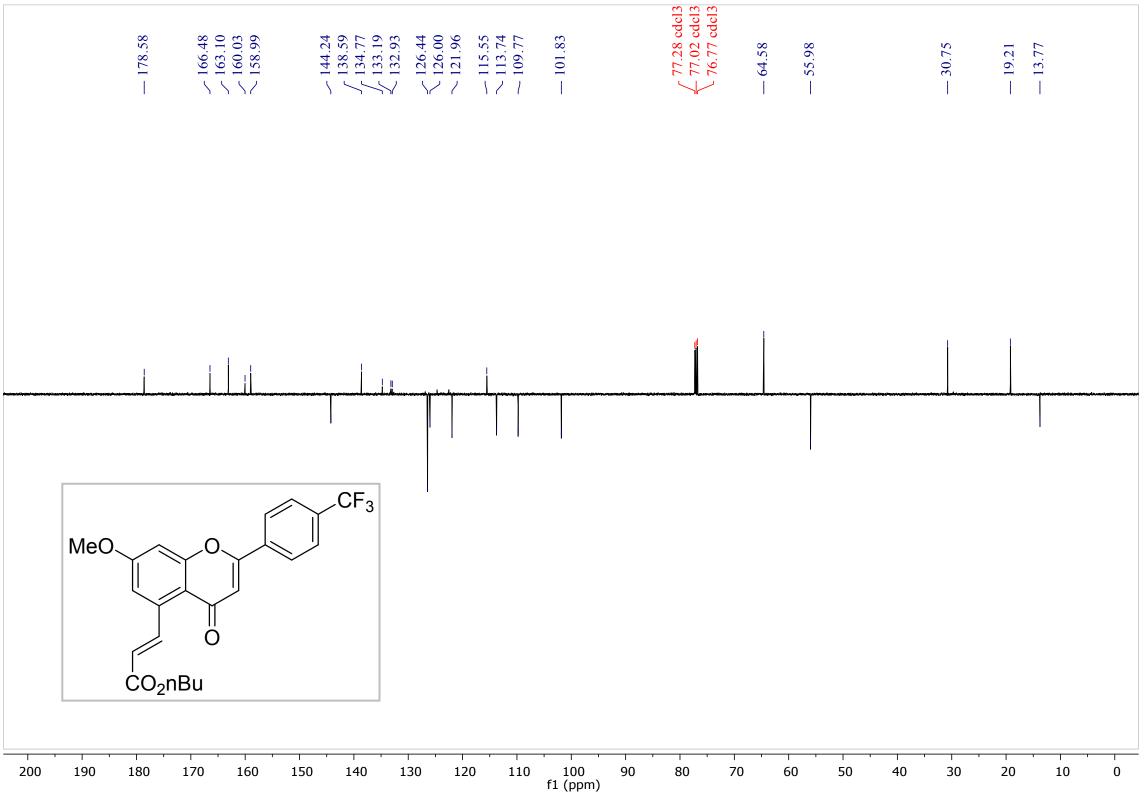

^{1}H NMR (500 MHz, $CDCl_3$) Spectra of Compound **9q**

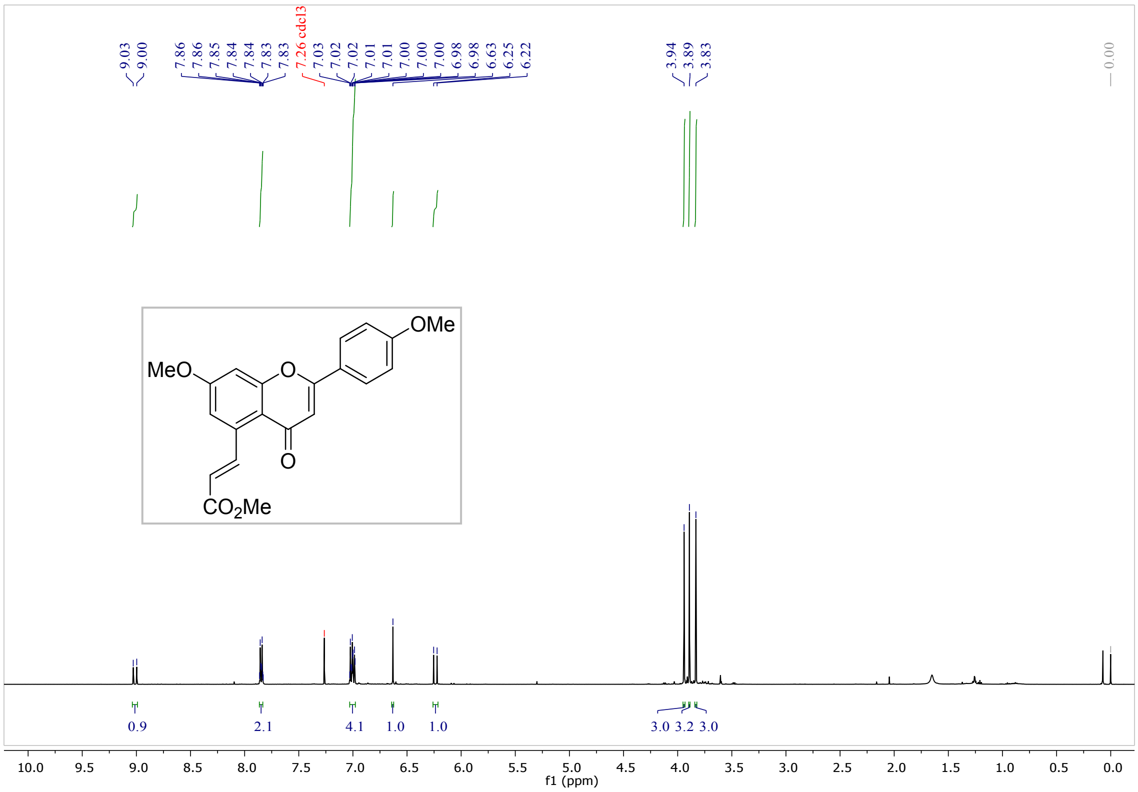

$^{13}C\{^1H\}$ NMR (126 MHz, $CDCl_3$) Spectra of Compound **9q**

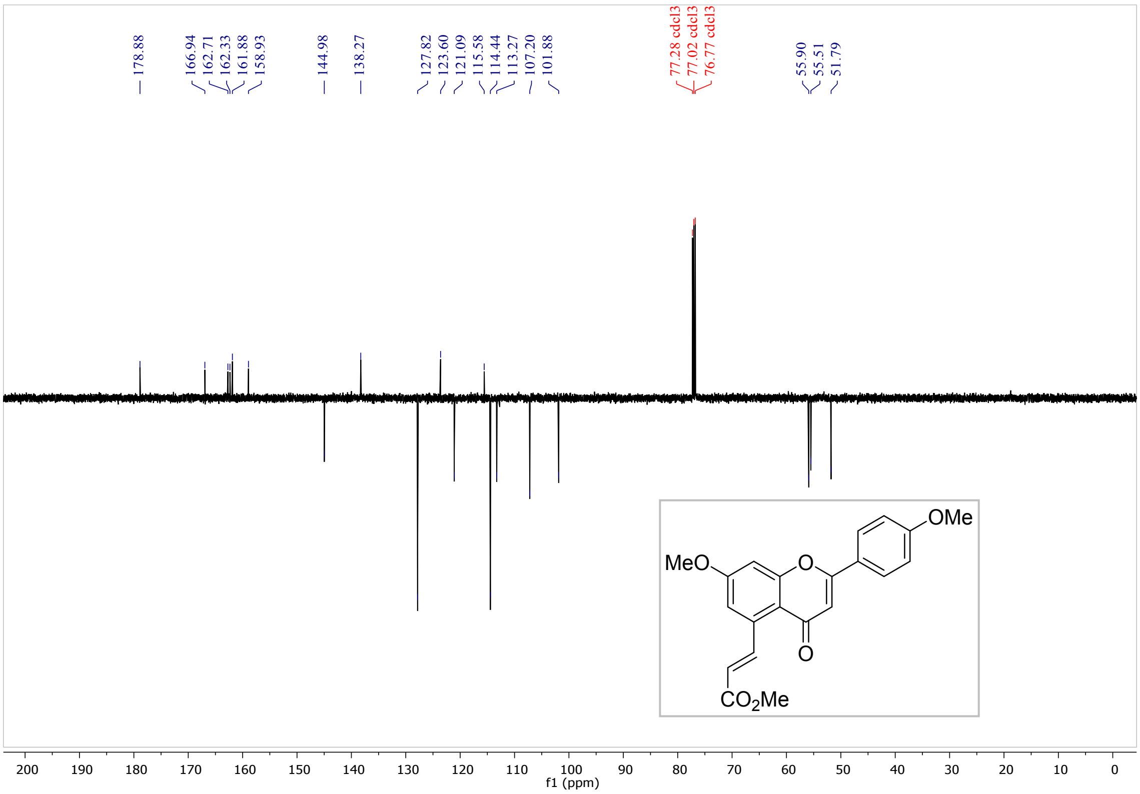

^1H NMR (500 MHz, $CDCl_3$) Spectra of Compound **9r**

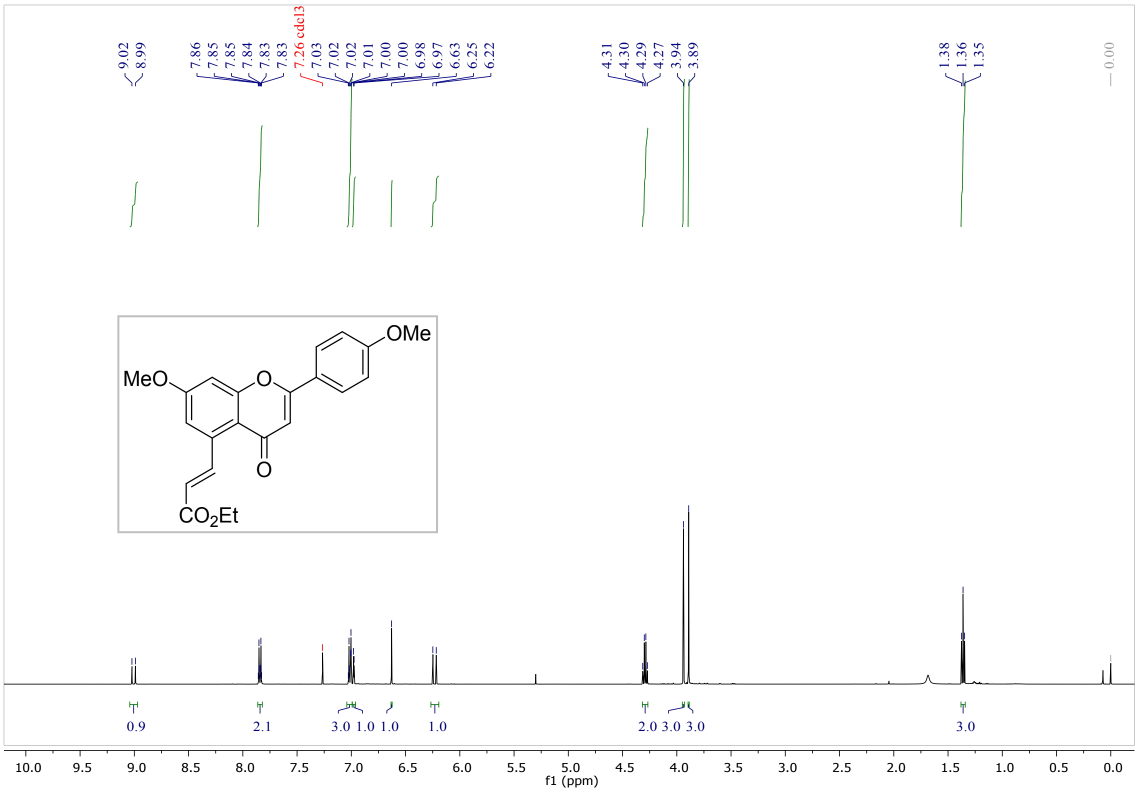

$^{13}C\{^{1}H\}$ NMR (126 MHz, $CDCl_3$) Spectra of Compound **9r**

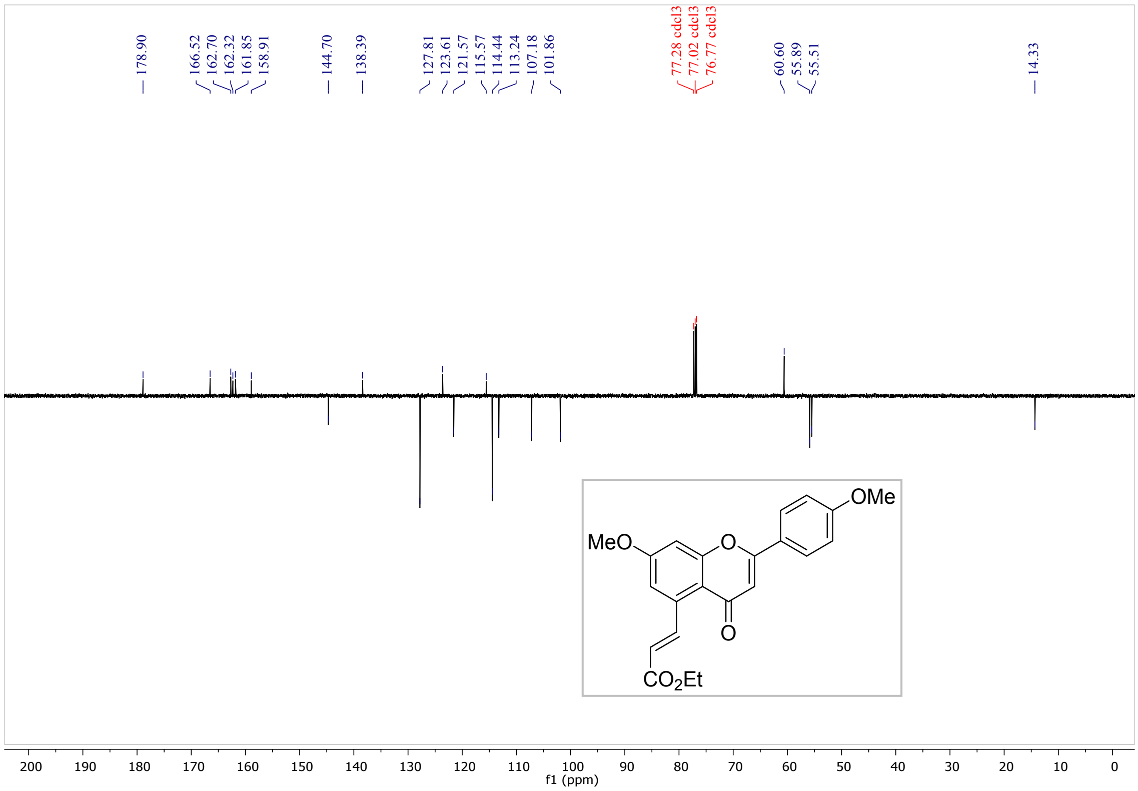

^{1}H NMR (500 MHz, $CDCl_3$) Spectra of Compound **9s**

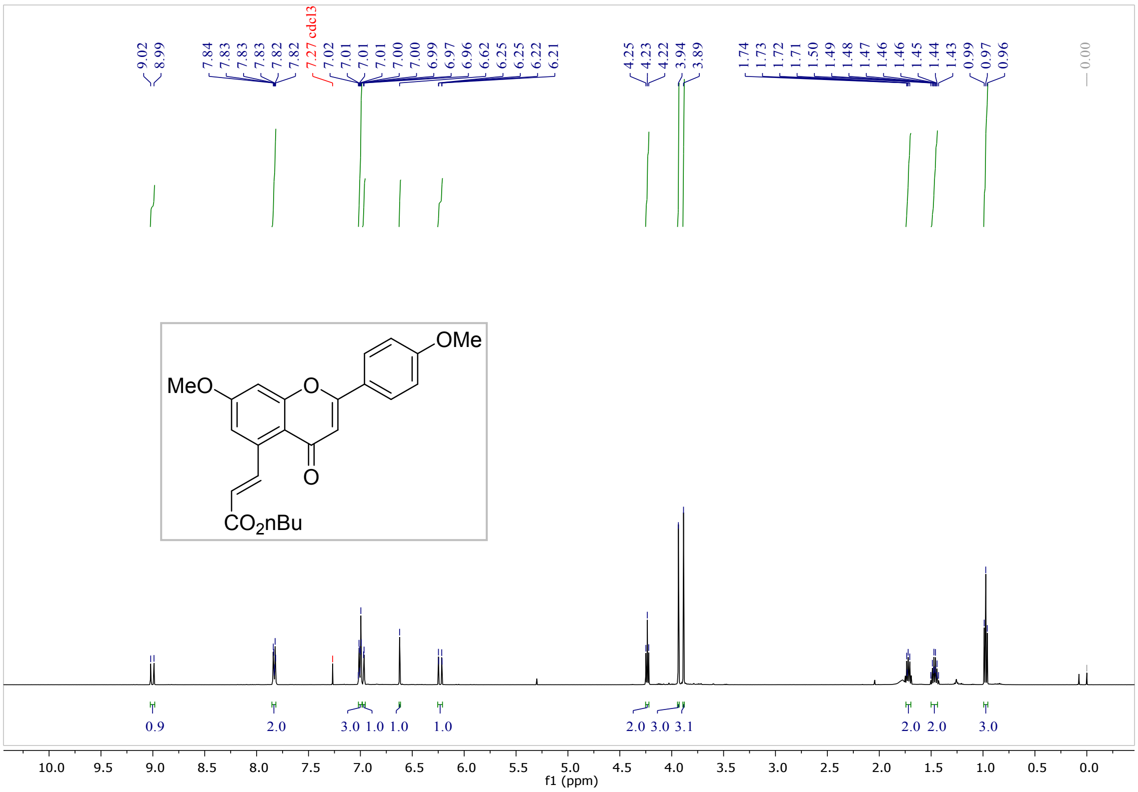

$^{13}C\{^1H\}$ NMR (126 MHz, $CDCl_3$) Spectra of Compound **9s**

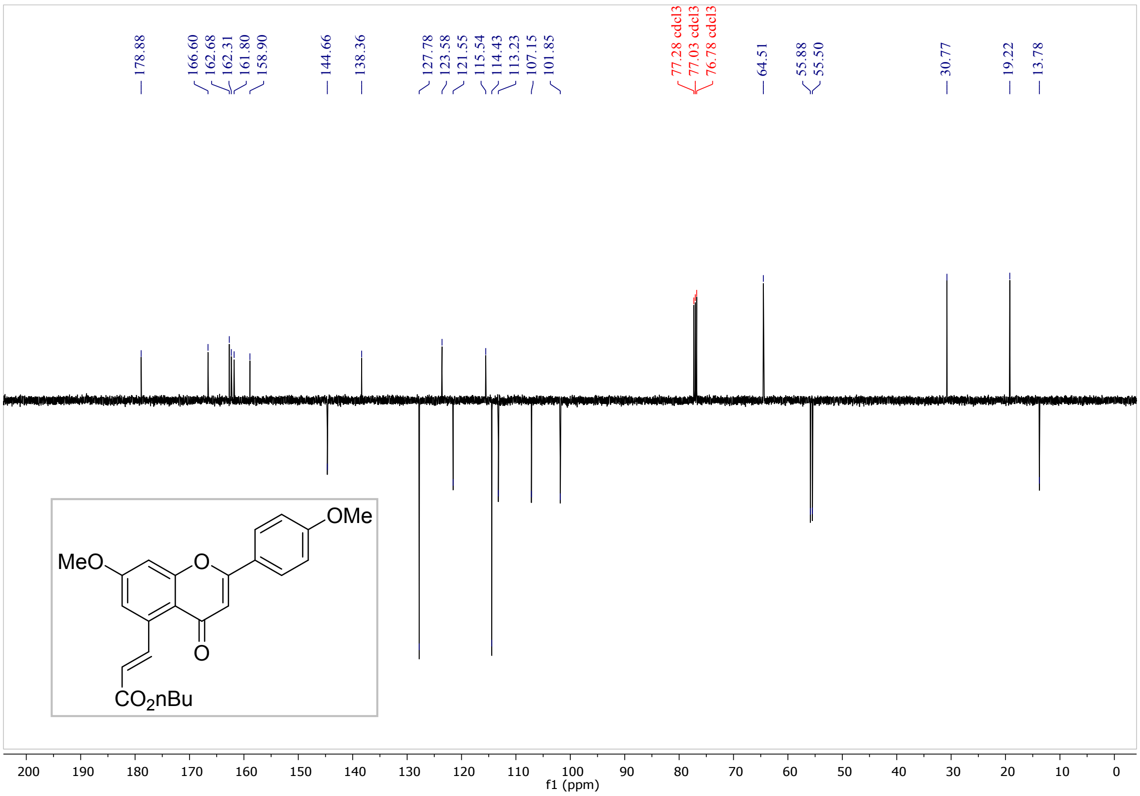

^1H NMR (500 MHz, $CDCl_3$) Spectra of Compound **9t**

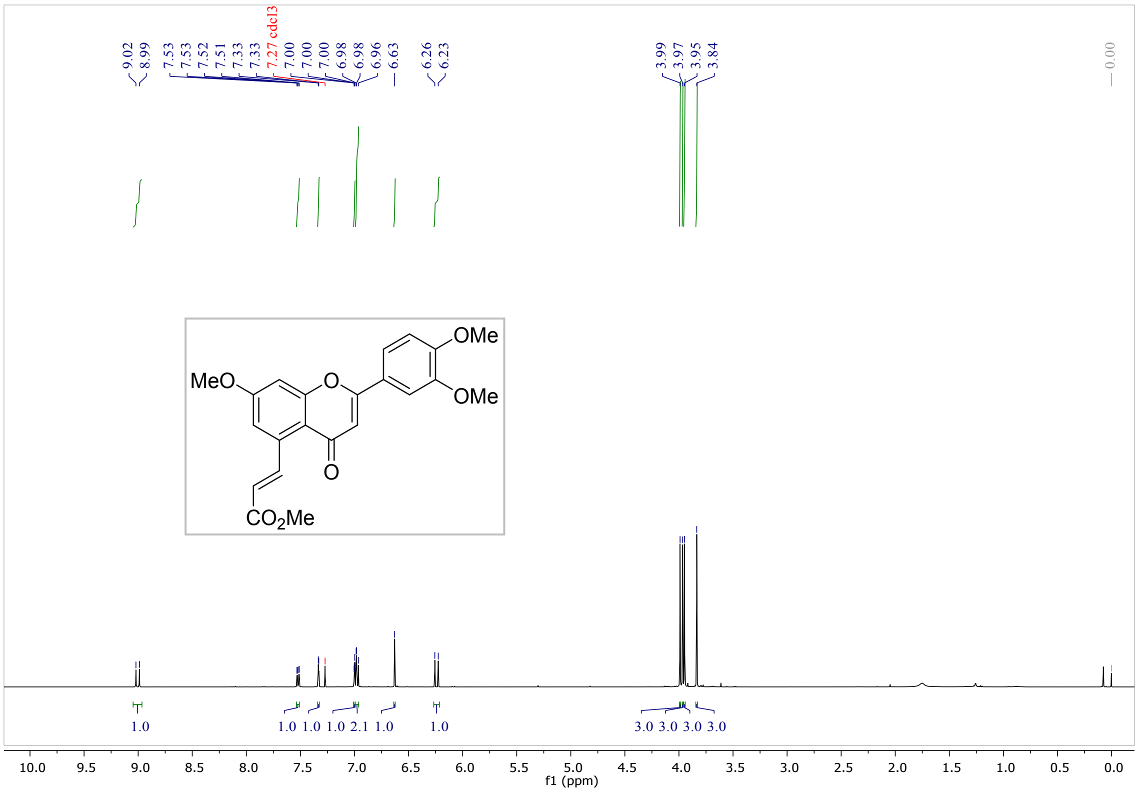

$^{13}C\{^1H\}$ NMR (126 MHz, $CDCl_3$) Spectra of Compound **9t**

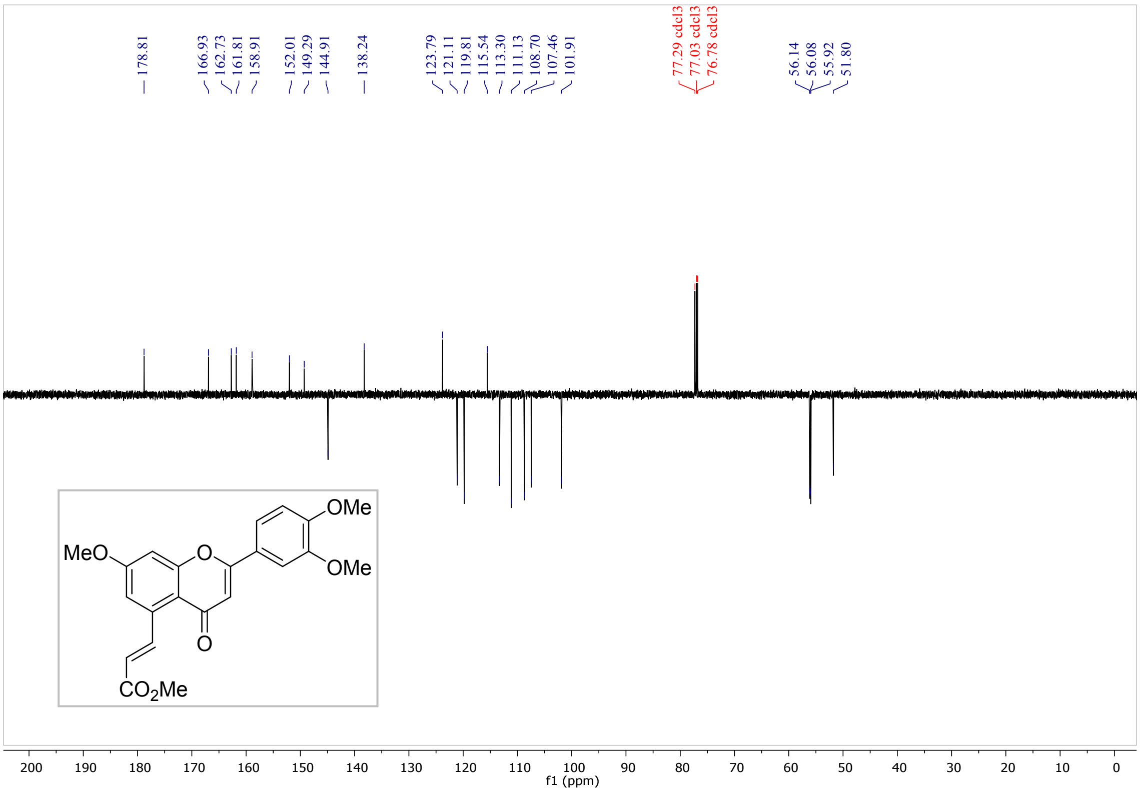

^1H NMR (500 MHz, $CDCl_3$) Spectra of Compound **9u**

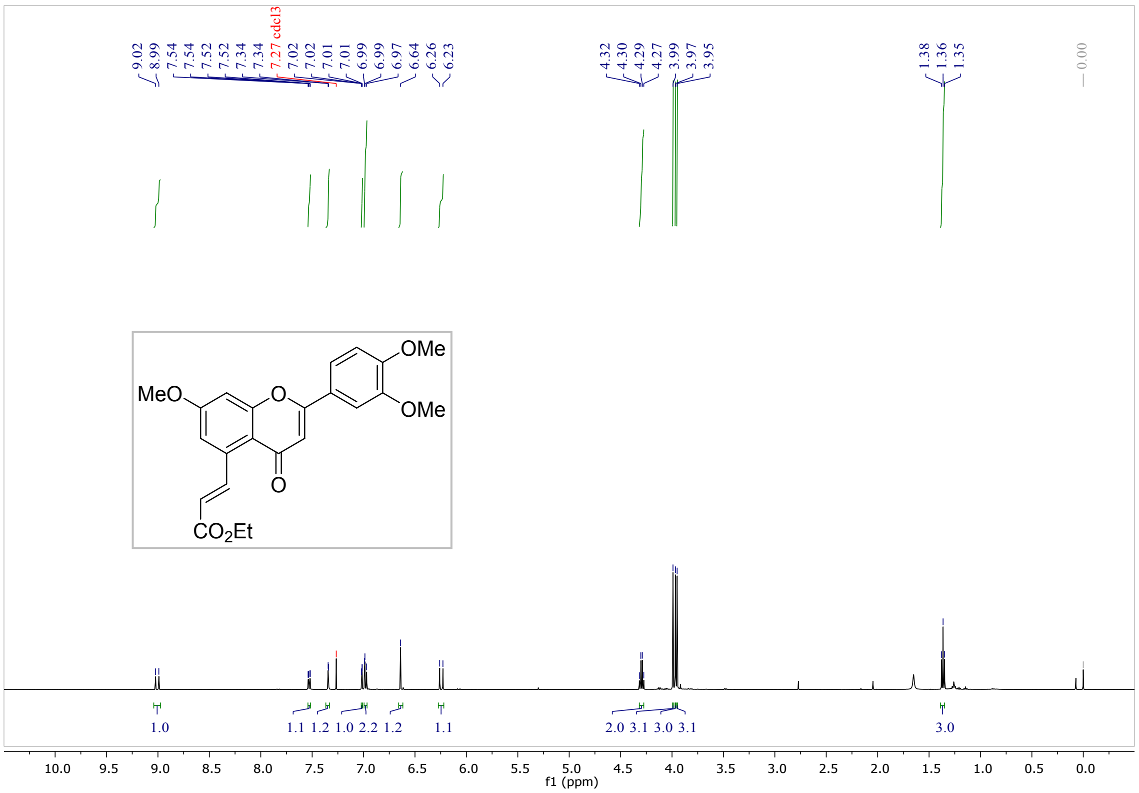

$^{13}C\{^1H\}$ NMR (101 MHz, $CDCl_3$) Spectra of Compound **9u**

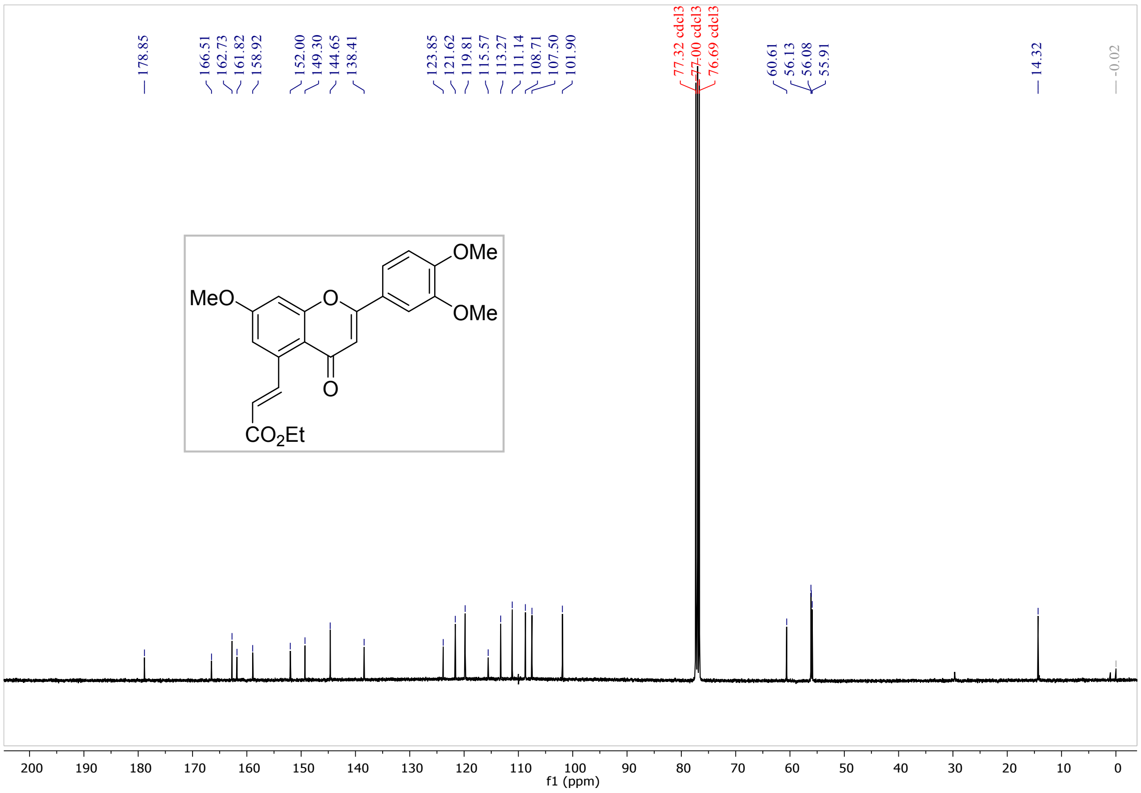

^1H NMR (500 MHz, $CDCl_3$) Spectra of Compound **9v**

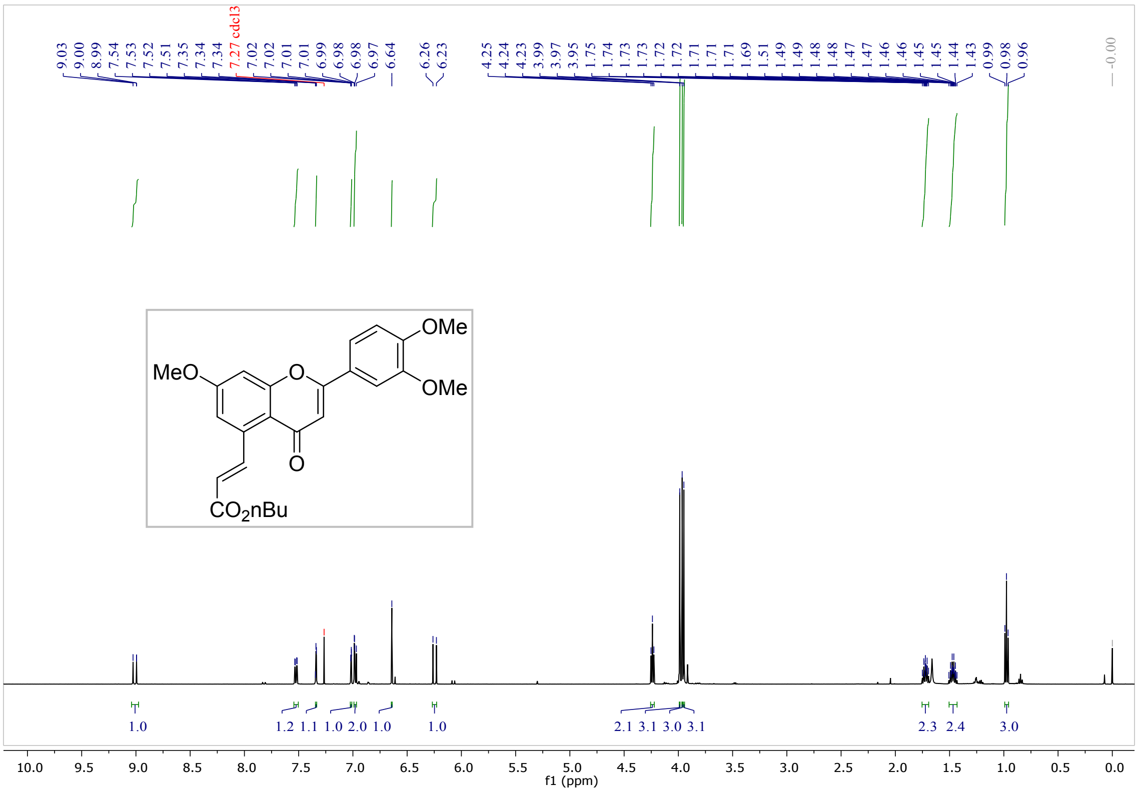

$^{13}C\{^{1}H\}$ NMR (101 MHz, $CDCl_3$) Spectra of Compound **9v**

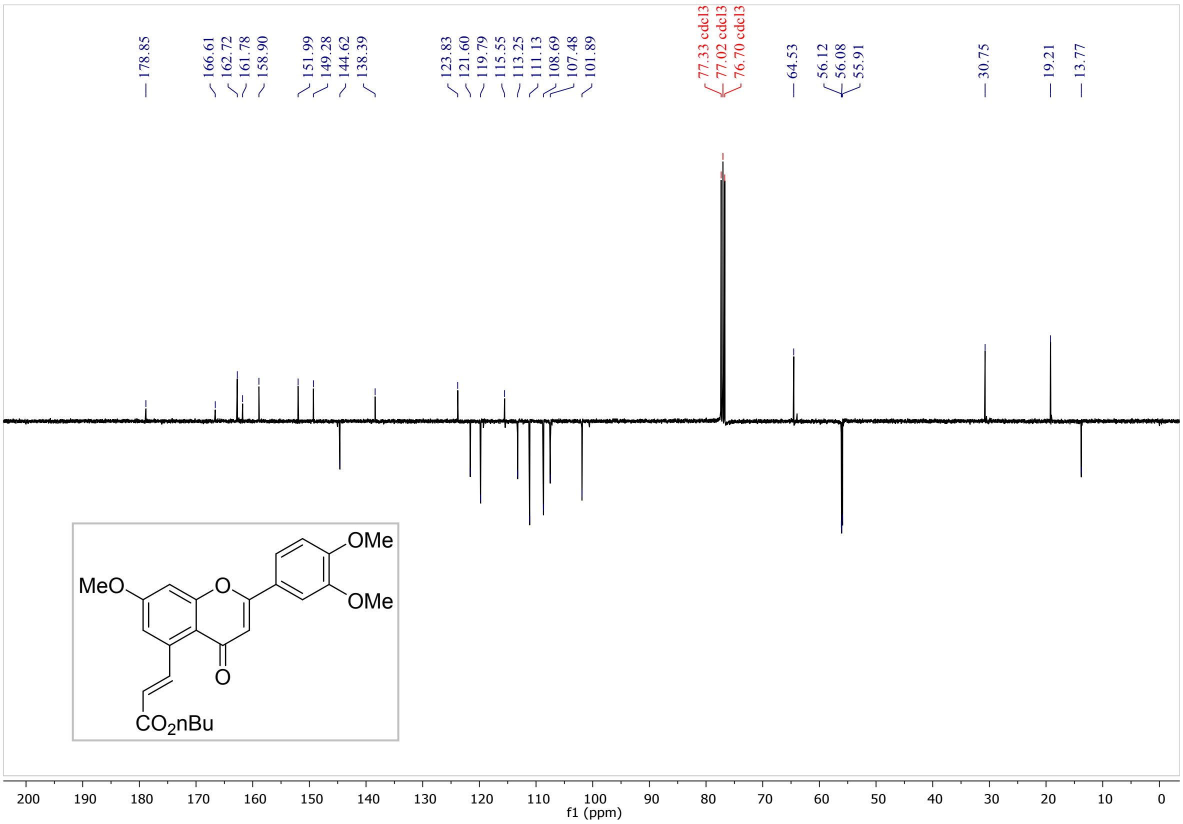

^{1}H NMR (500 MHz, $CDCl_3$) Spectra of Compound **9w**

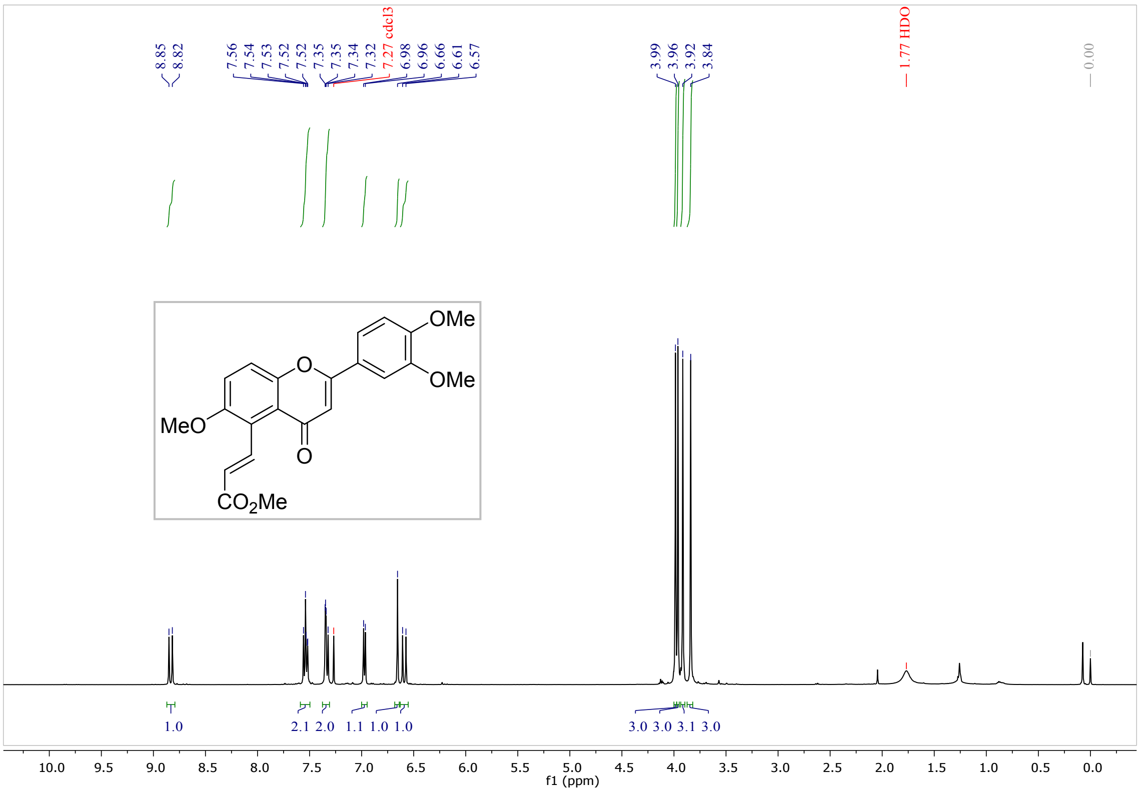

^{13}C{^{1}H} NMR (126 MHz, $CDCl_3$) Spectra of Compound **9w**

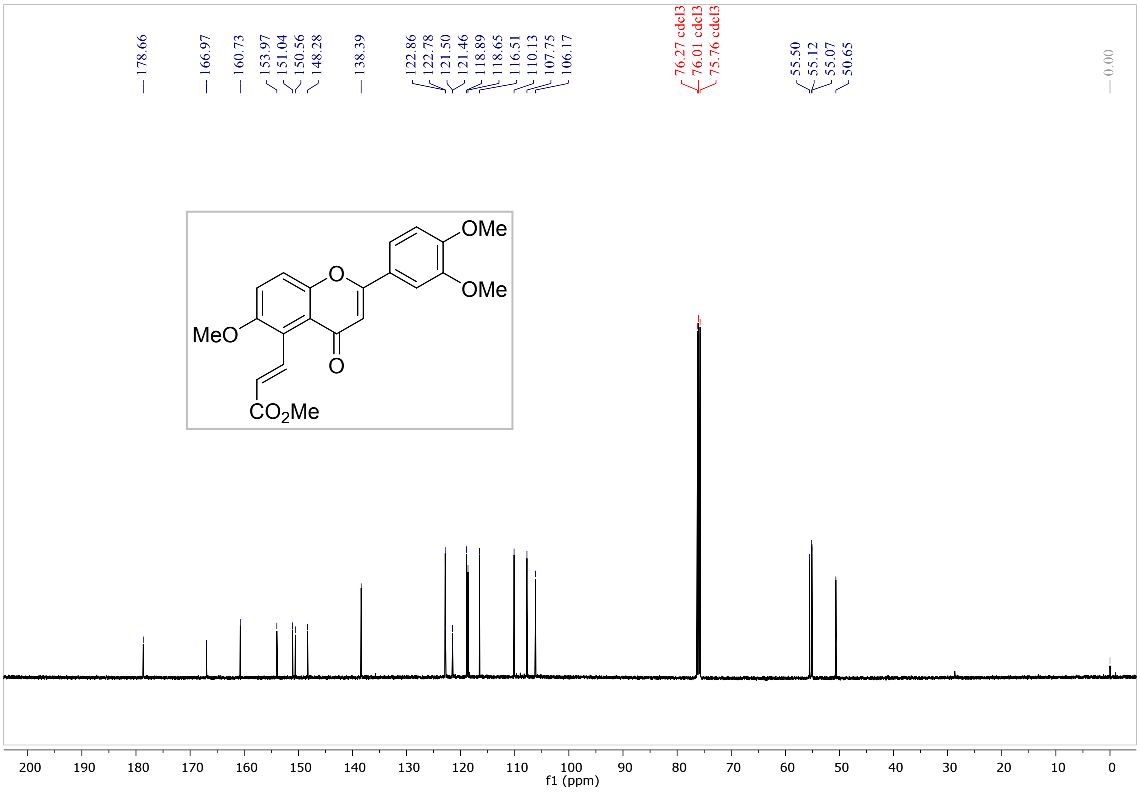

^{1}H NMR (500 MHz, $CDCl_3$) Spectra of Compound **9x**

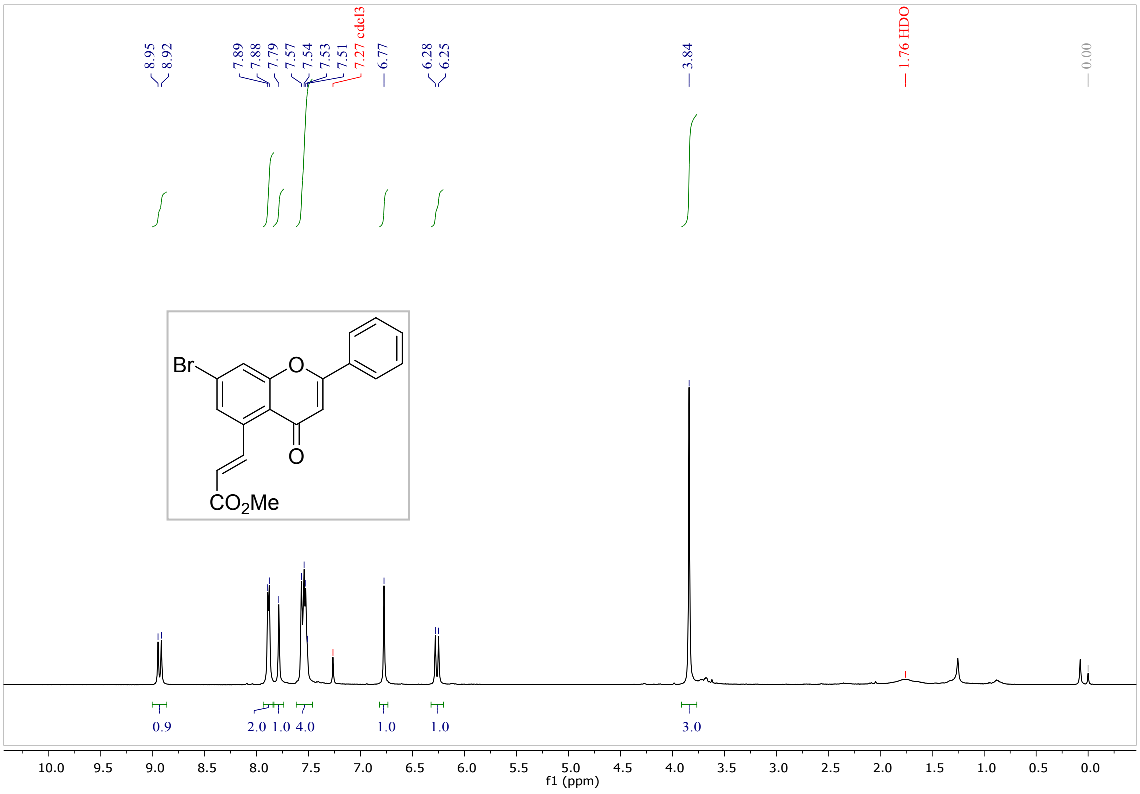

$^{13}C\{^1H\}$ NMR (126 MHz, $CDCl_3$) Spectra of Compound **9x**

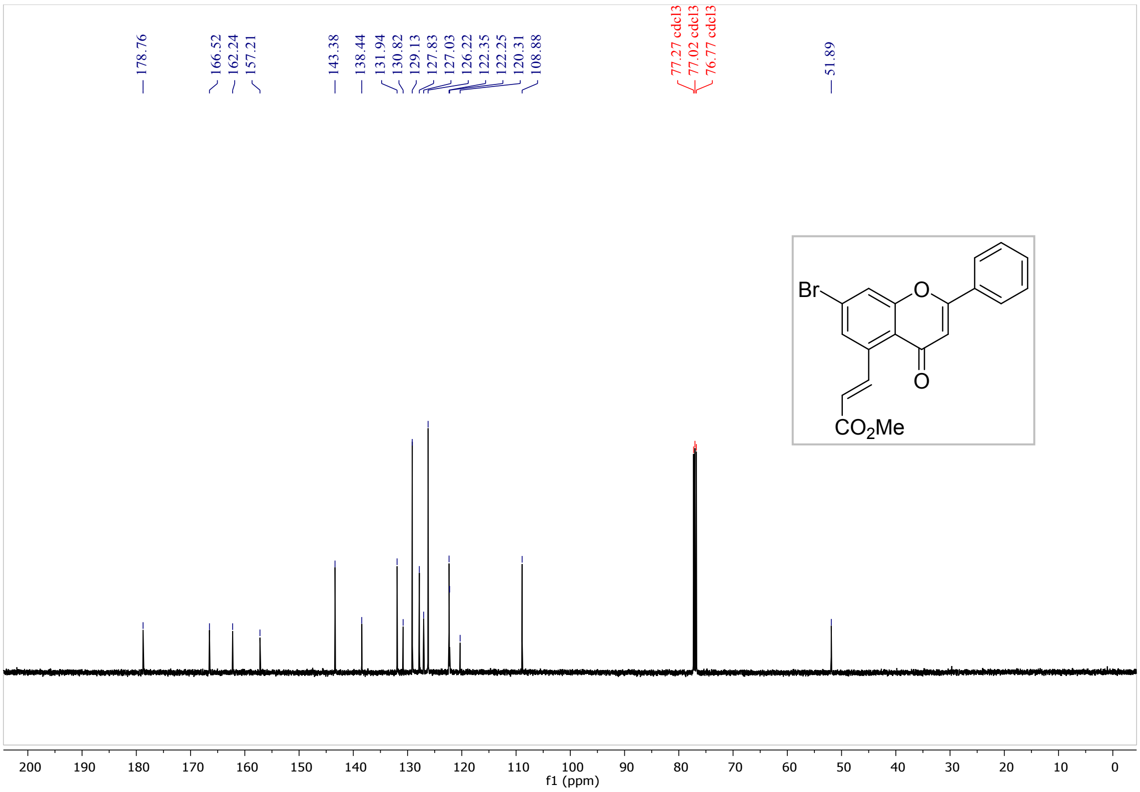

^1H NMR (500 MHz, $CDCl_3$) Spectra of Compound **11a**

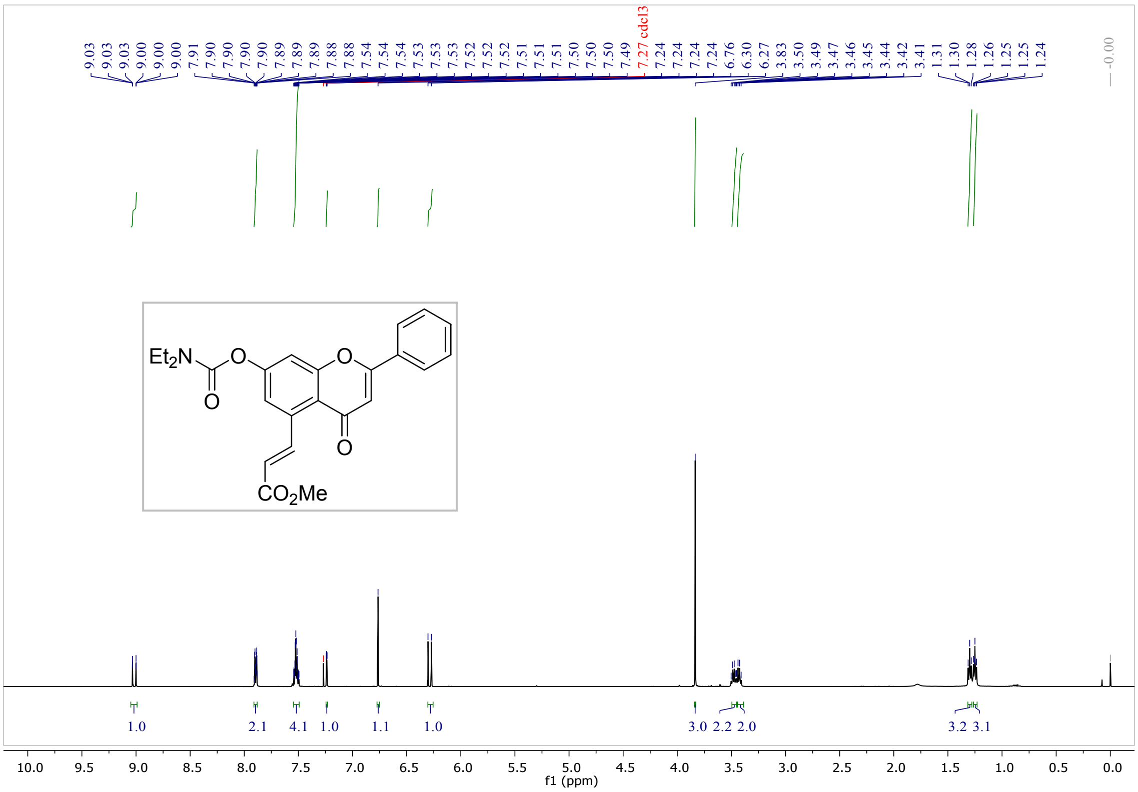

$^{13}C\{^{1}H\}$ NMR (126 MHz, $CDCl_3$) Spectra of Compound **11a**

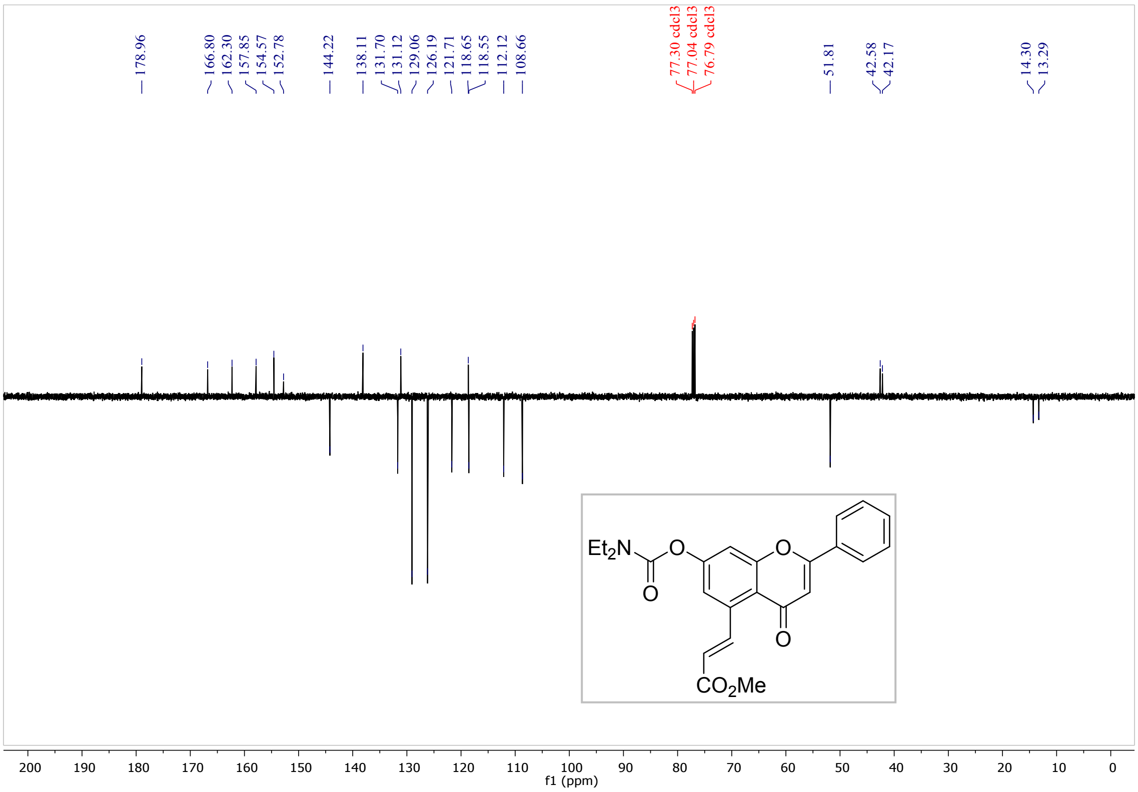

HSQC NMR (500, 126 MHz, $CDCl_3$) Spectra of Compound **11a**

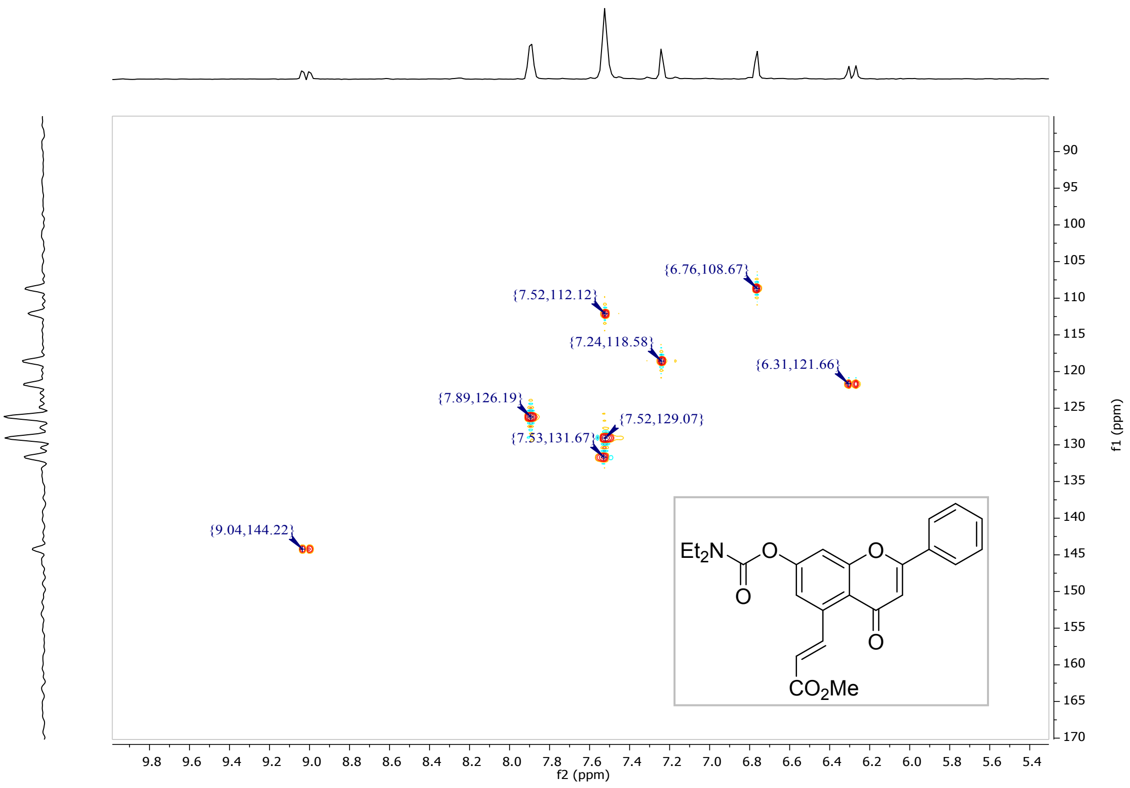

HMBC NMR (400, 101 MHz, $CDCl_3$) Spectra of Compound **11a**

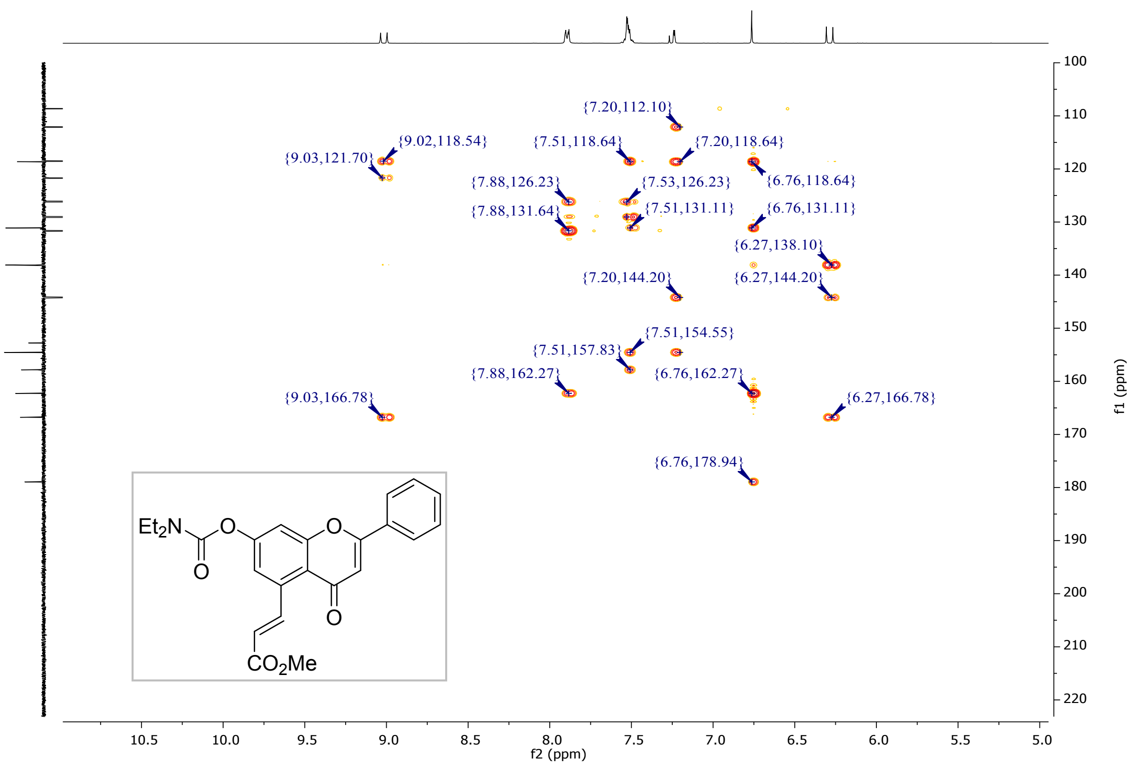

COSY NMR (400 MHz, $CDCl_3$) Spectra of Compound **11a**

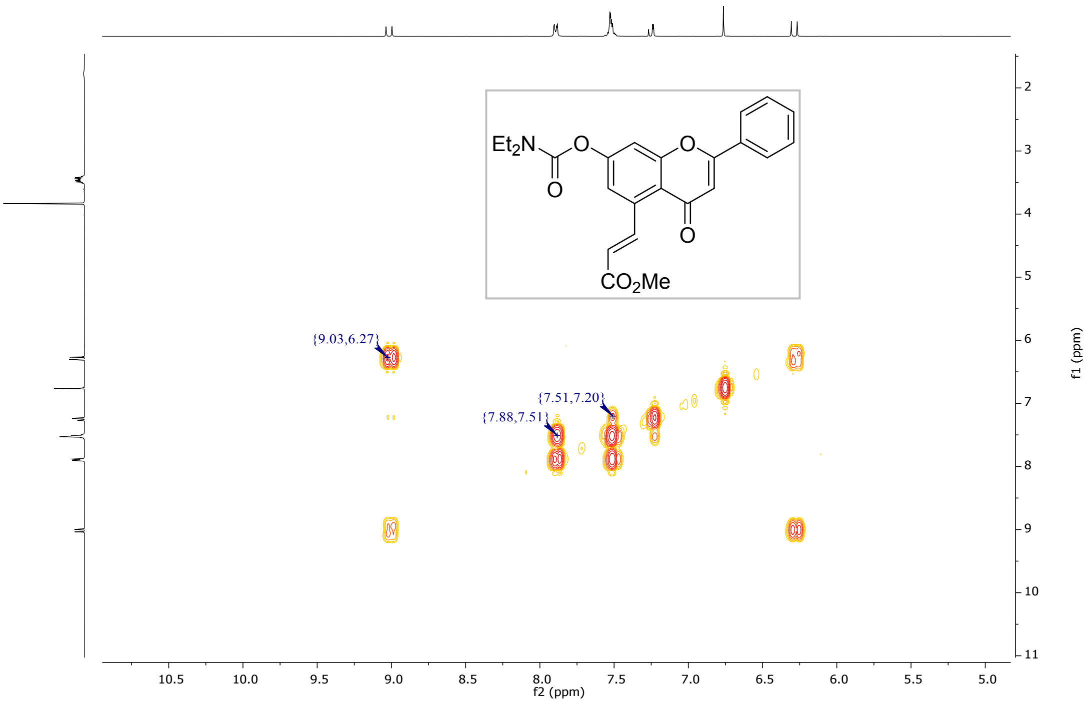

^{1}H NMR (400 MHz, $CDCl_3$) Spectra of Compound **11b**

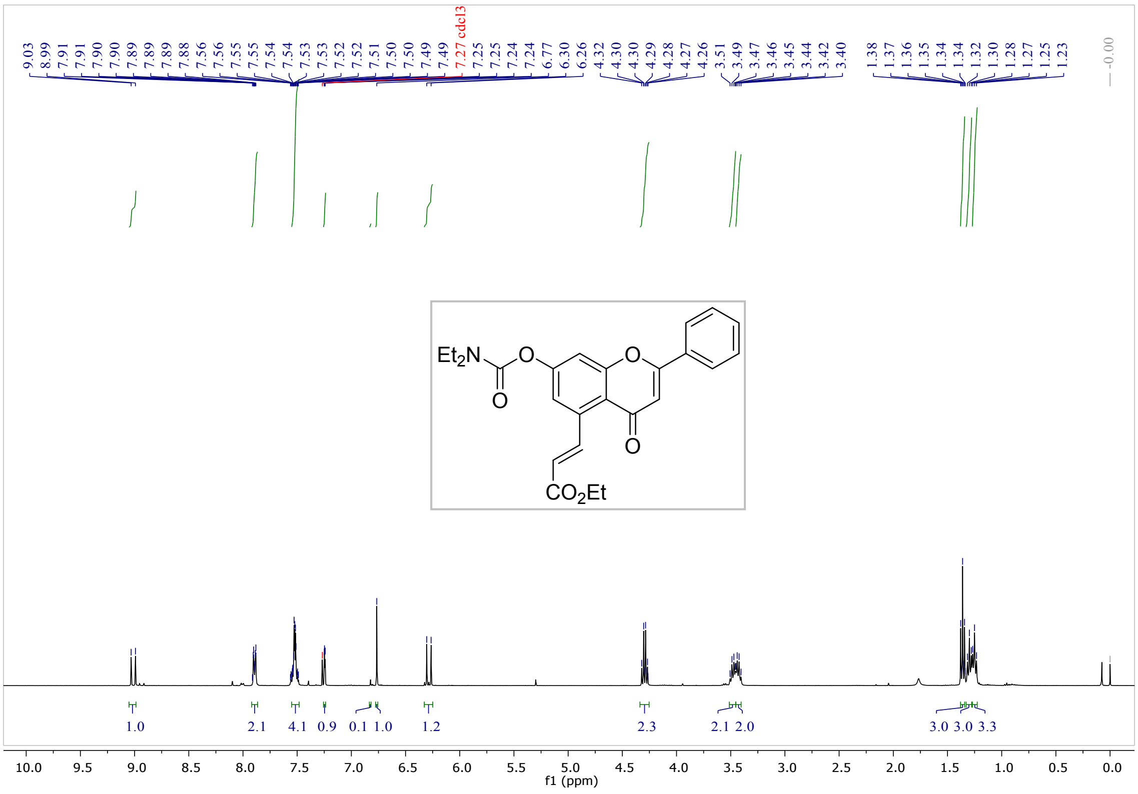

$^{13}C\{^{1}H\}$ NMR (126 MHz, $CDCl_3$) Spectra of Compound **11b**

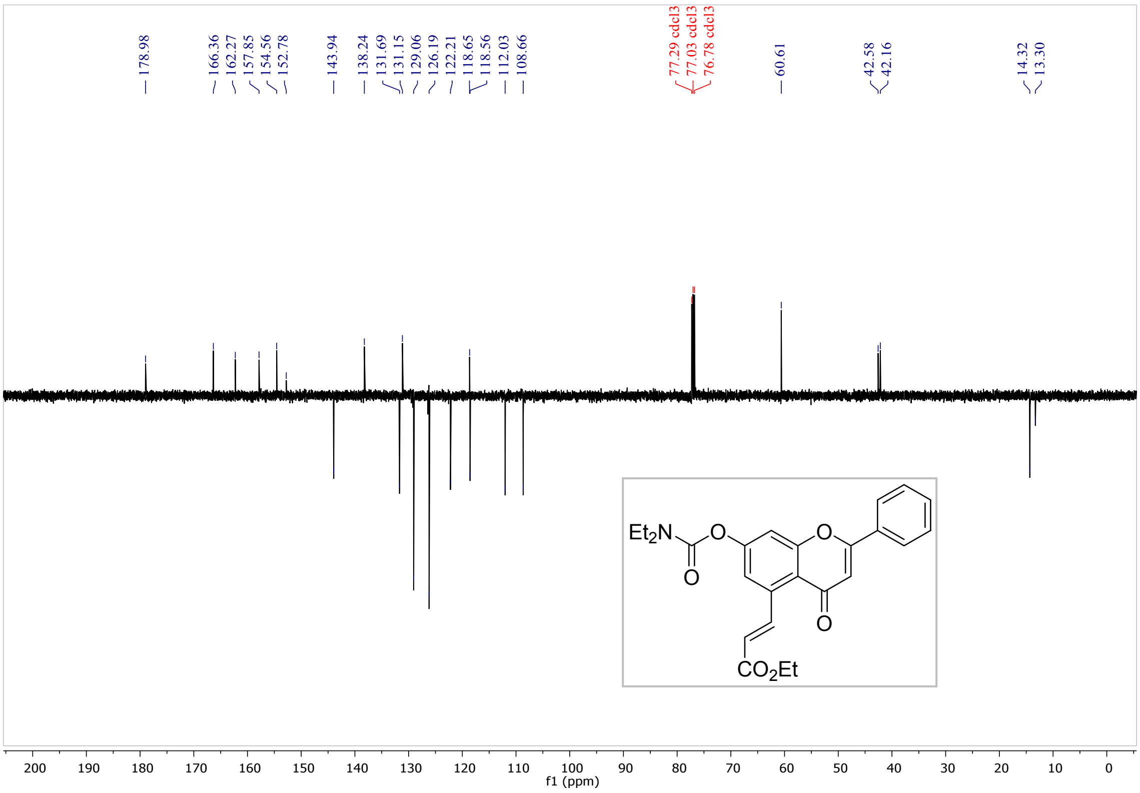

^{1}H NMR (500 MHz, $CDCl_3$) Spectra of Compound **11c**

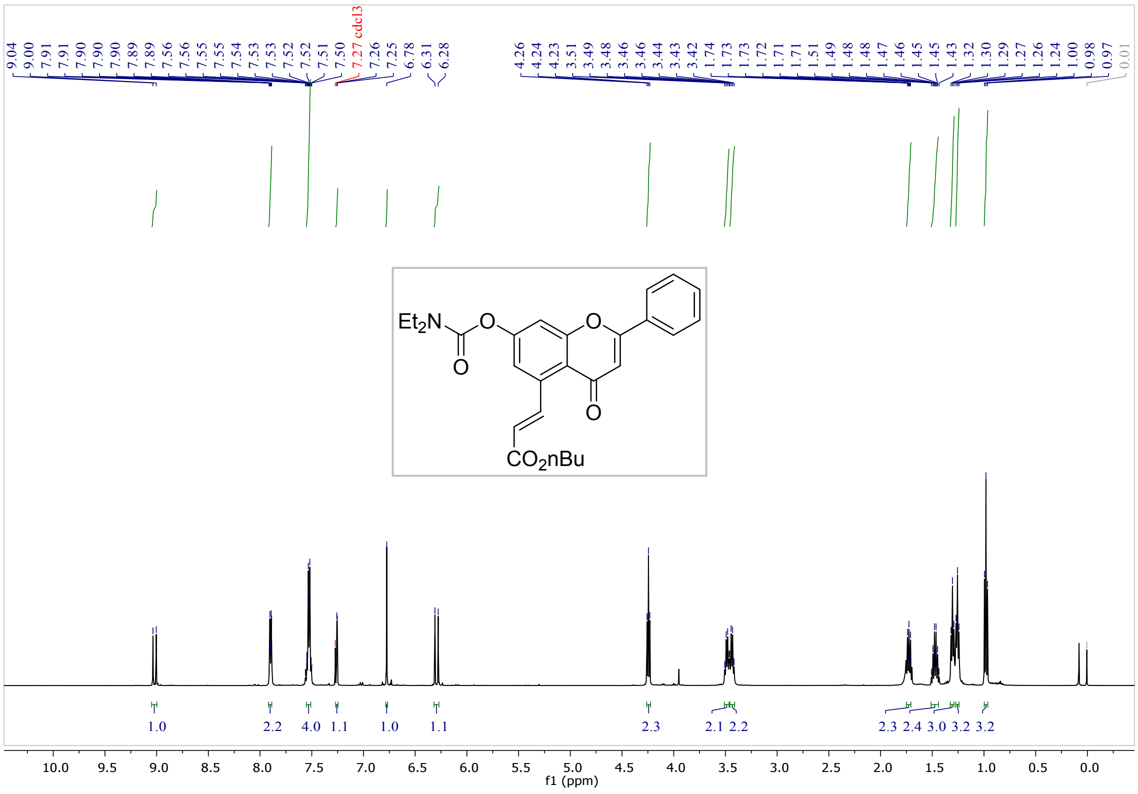

$^{13}C\{^1H\}$ NMR (126 MHz, $CDCl_3$) Spectra of Compound **11c**

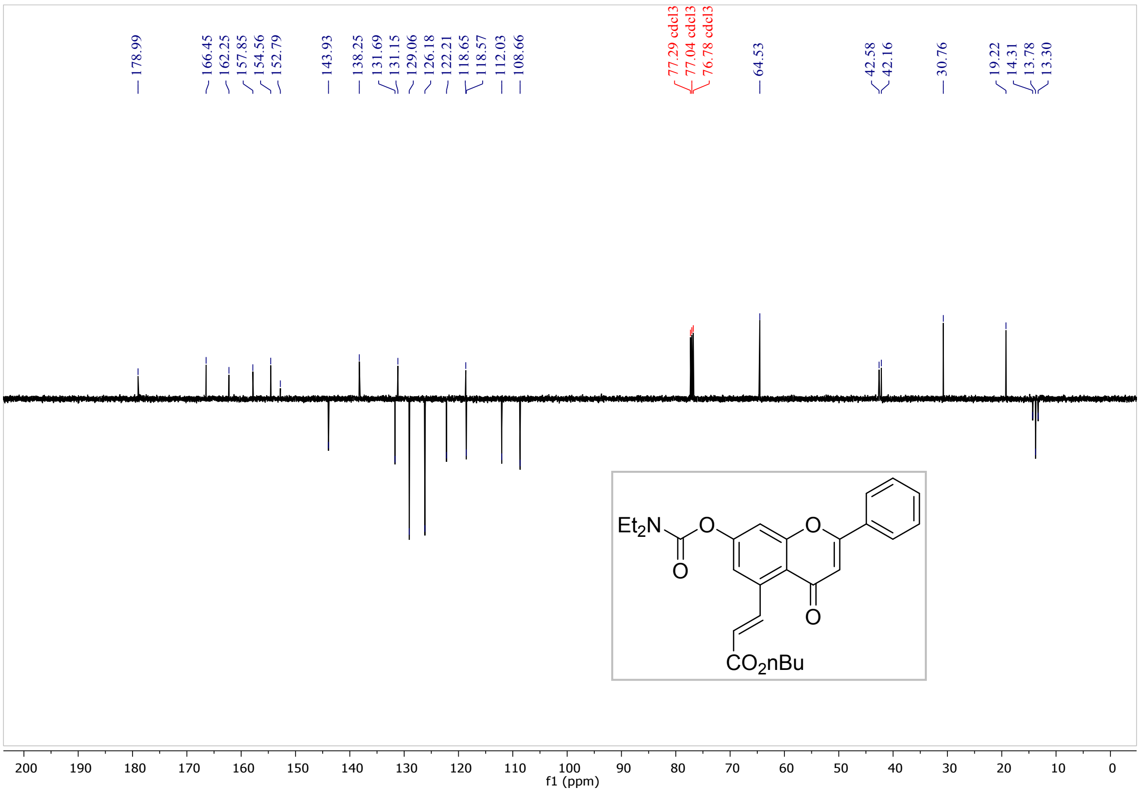

^1H NMR (400 MHz, $CDCl_3$) Spectra of Compound **11e**

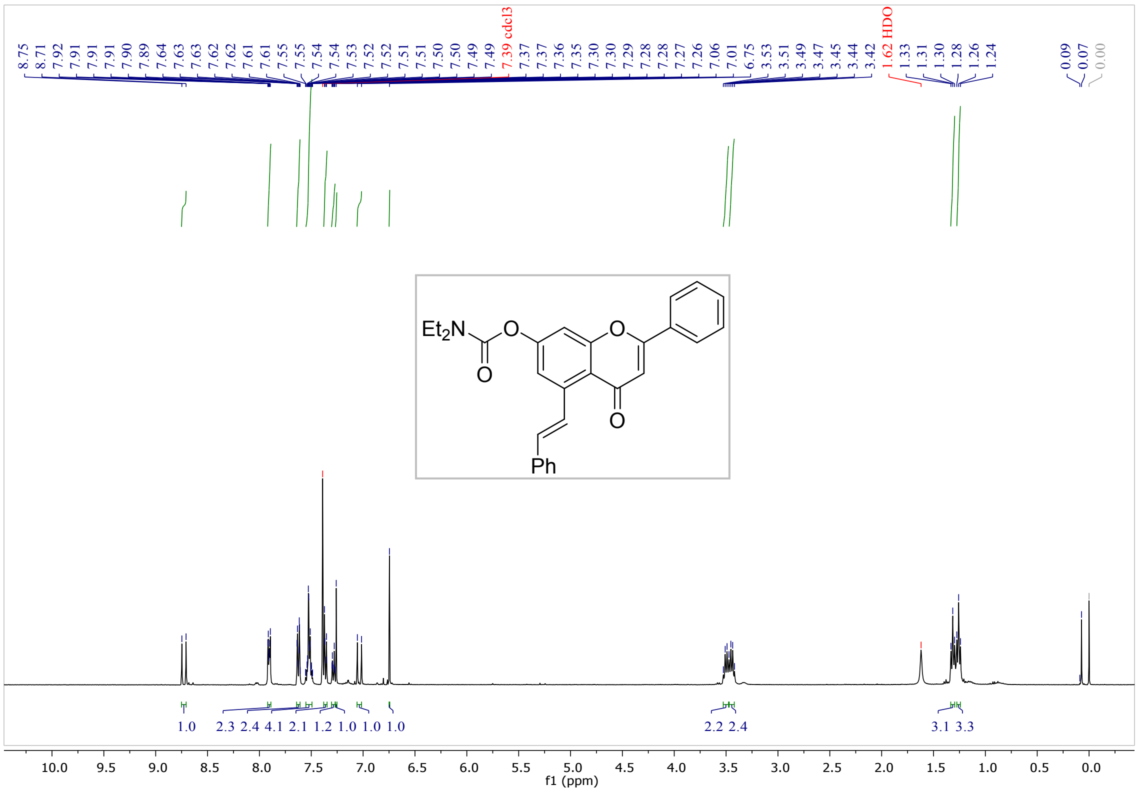

$^{13}C\{^{1}H\}$ NMR (101 MHz, $CDCl_3$) Spectra of Compound **11e**

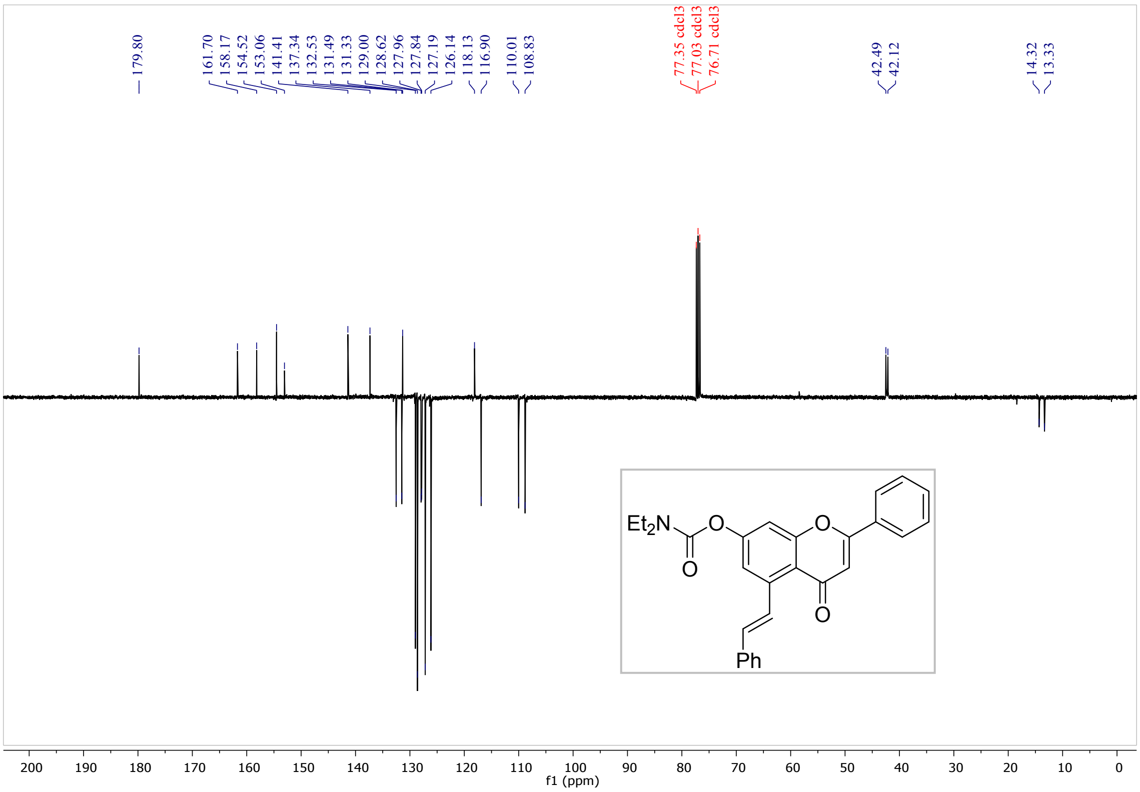

^{1}H NMR (500 MHz, $CDCl_3$) Spectra of Compound **11f**

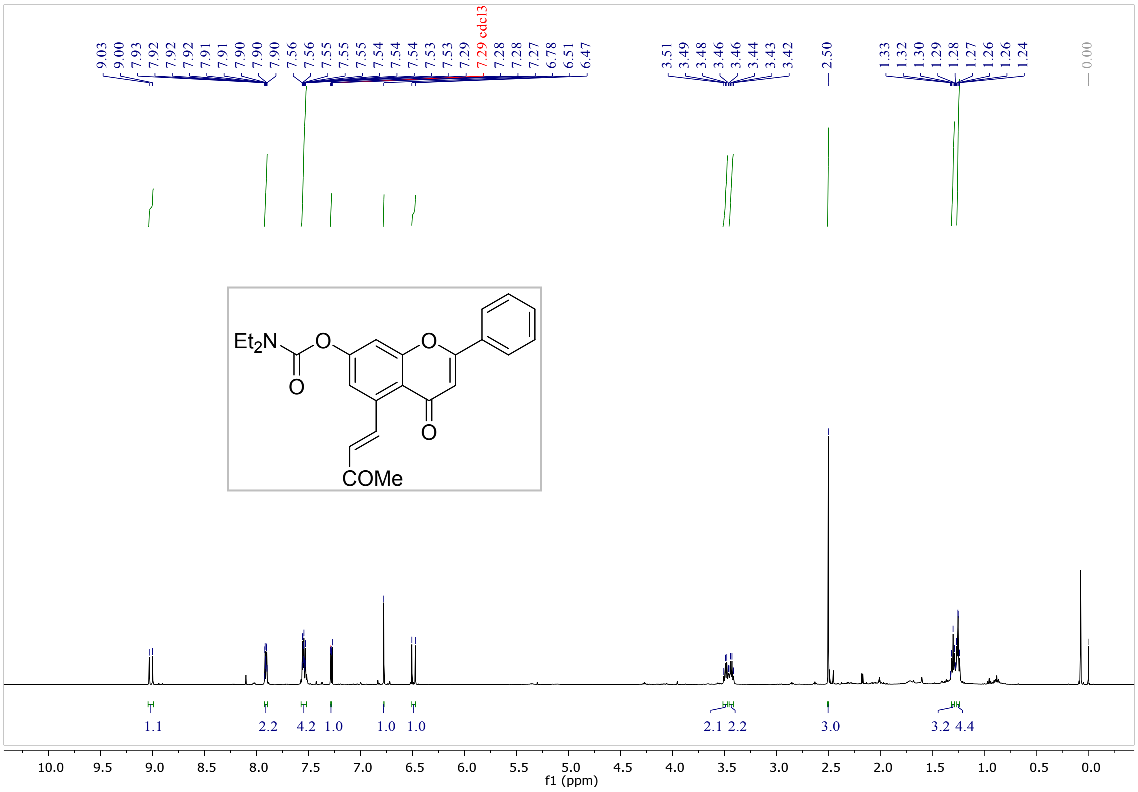

$^{13}C\{^{1}H\}$ NMR (126 MHz, $CDCl_3$) Spectra of Compound **11f**

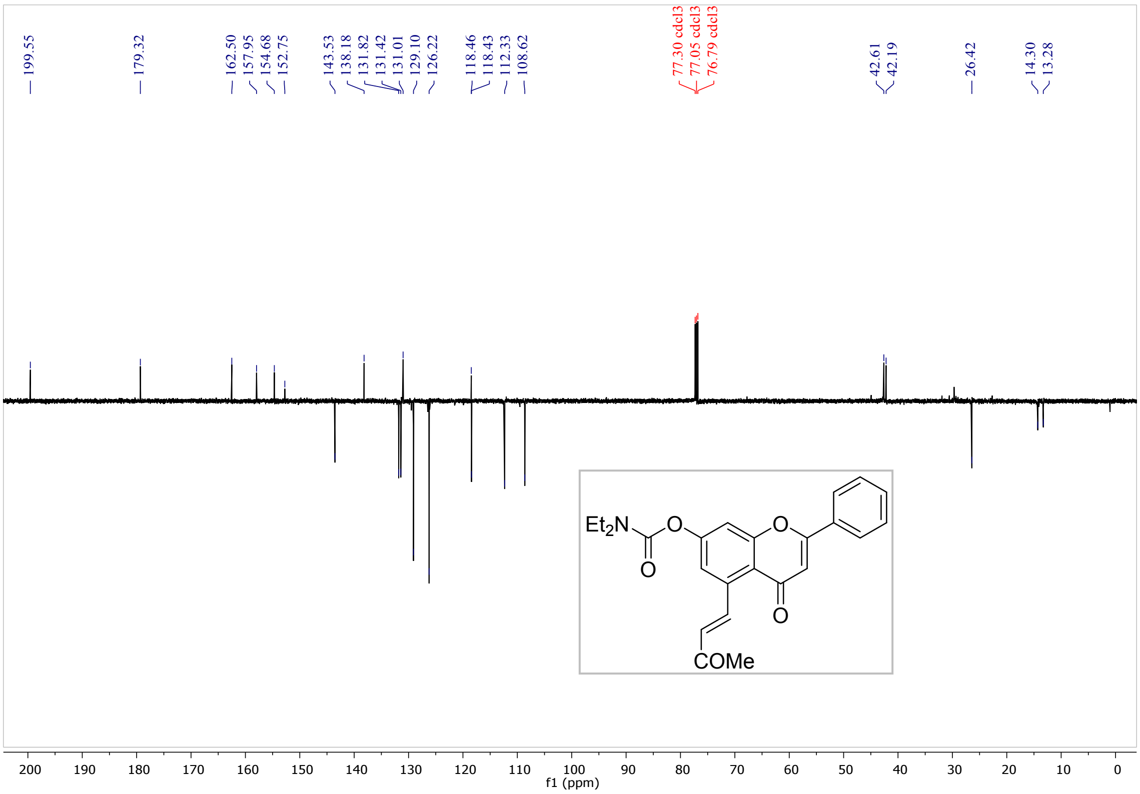

^{1}H NMR (500 MHz, $CDCl_3$) Spectra of Compound **11g**

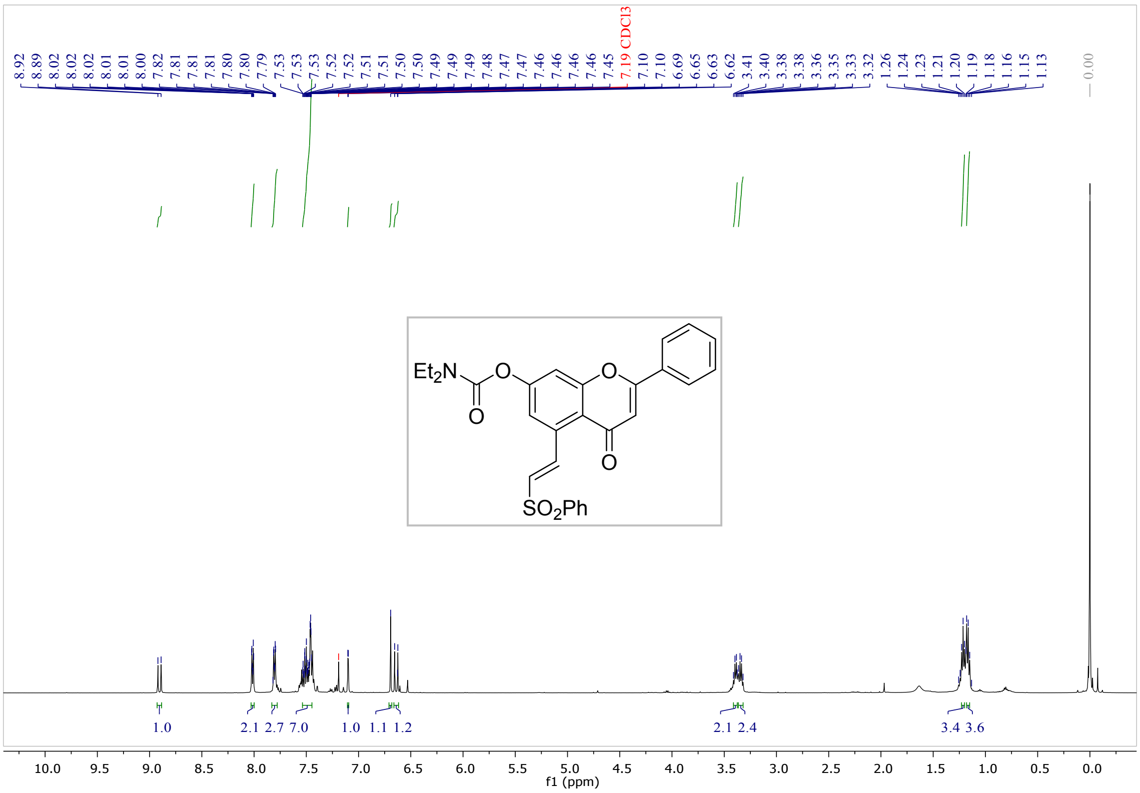

$^{13}C\{^{1}H\}$ NMR (126 MHz, $CDCl_3$) Spectra of Compound **11g**

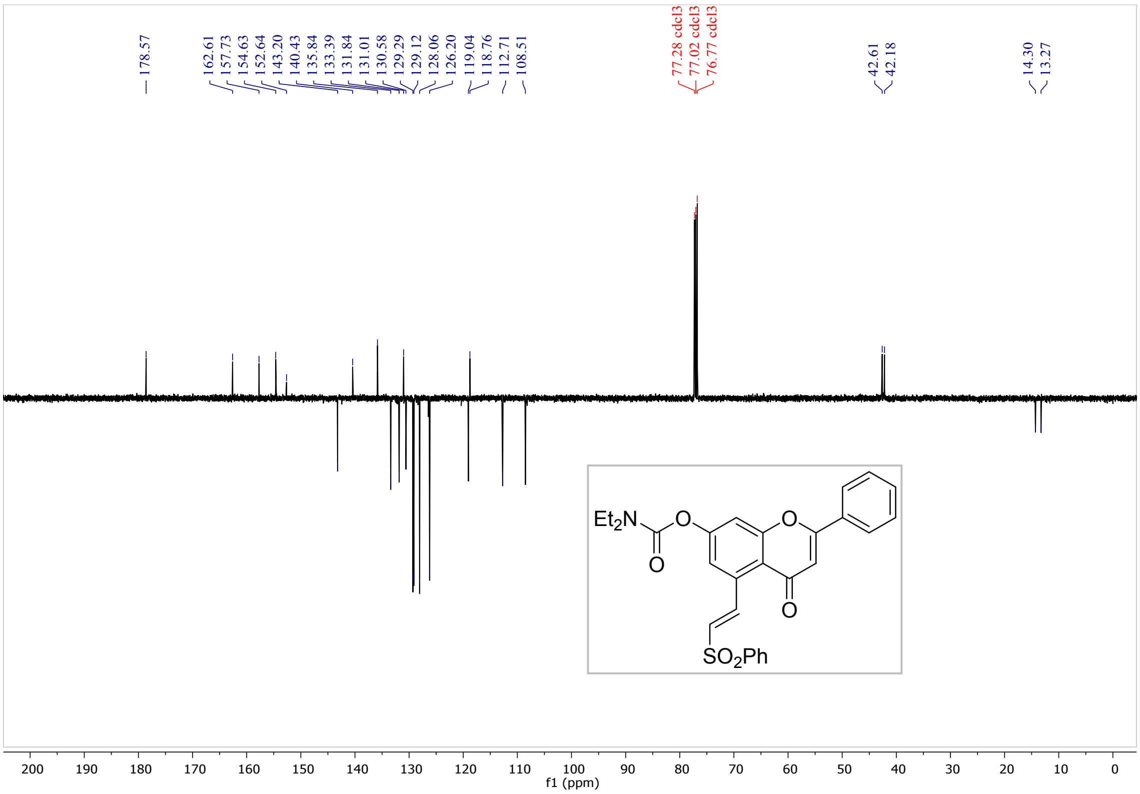

^{1}H NMR (500 MHz, $CDCl_3$) Spectra of Compound **11h**

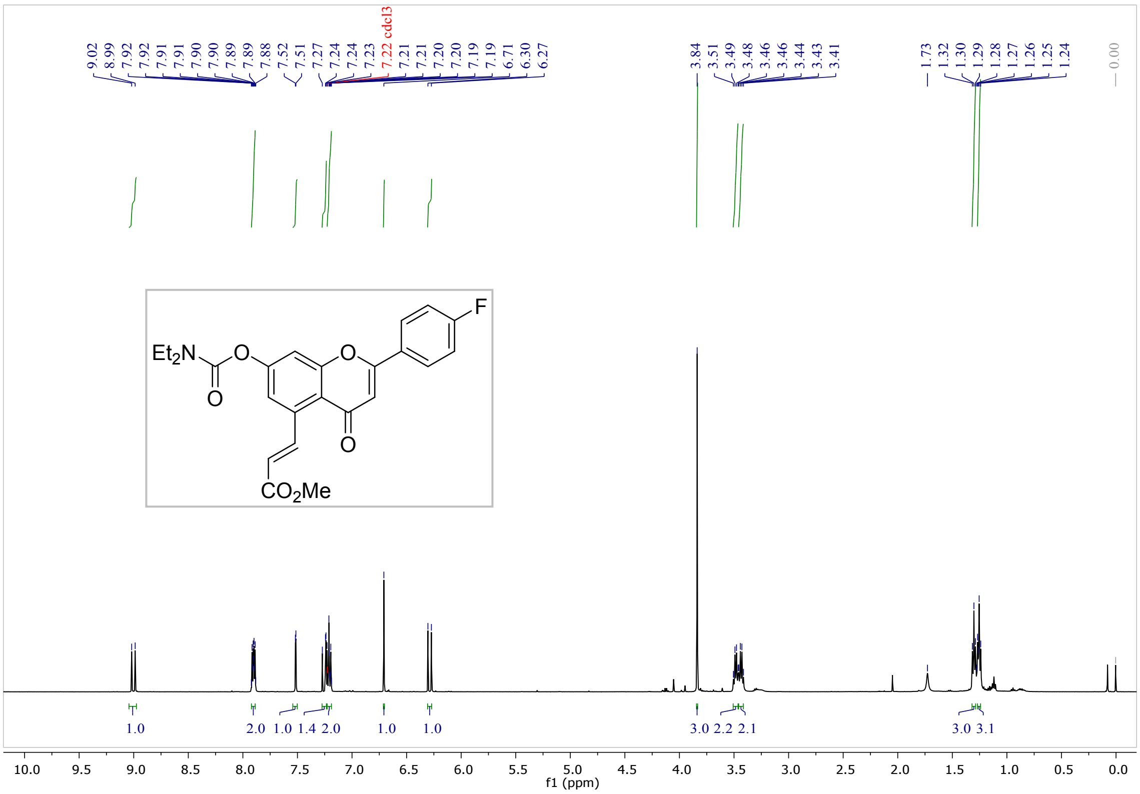

$^{13}C\{^1H\}$ NMR (126 MHz, $CDCl_3$) Spectra of Compound **11h**

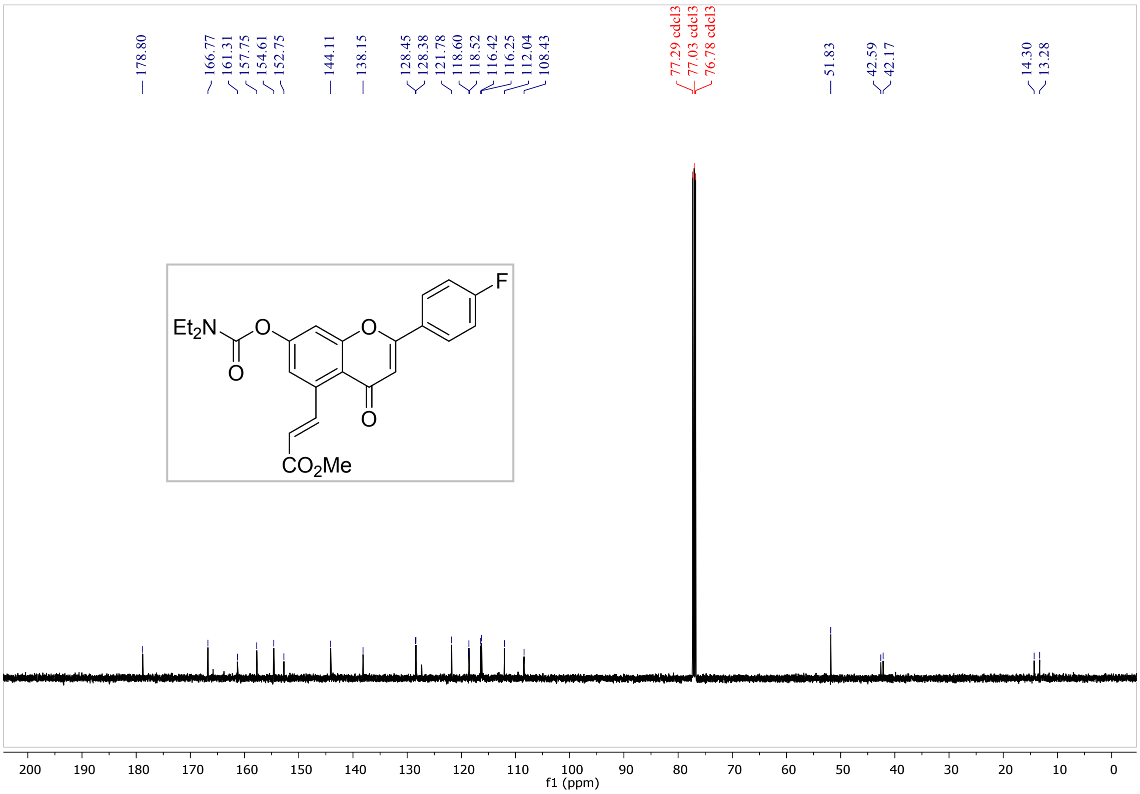

^1H NMR (400 MHz, $CDCl_3$) Spectra of Compound **11i**

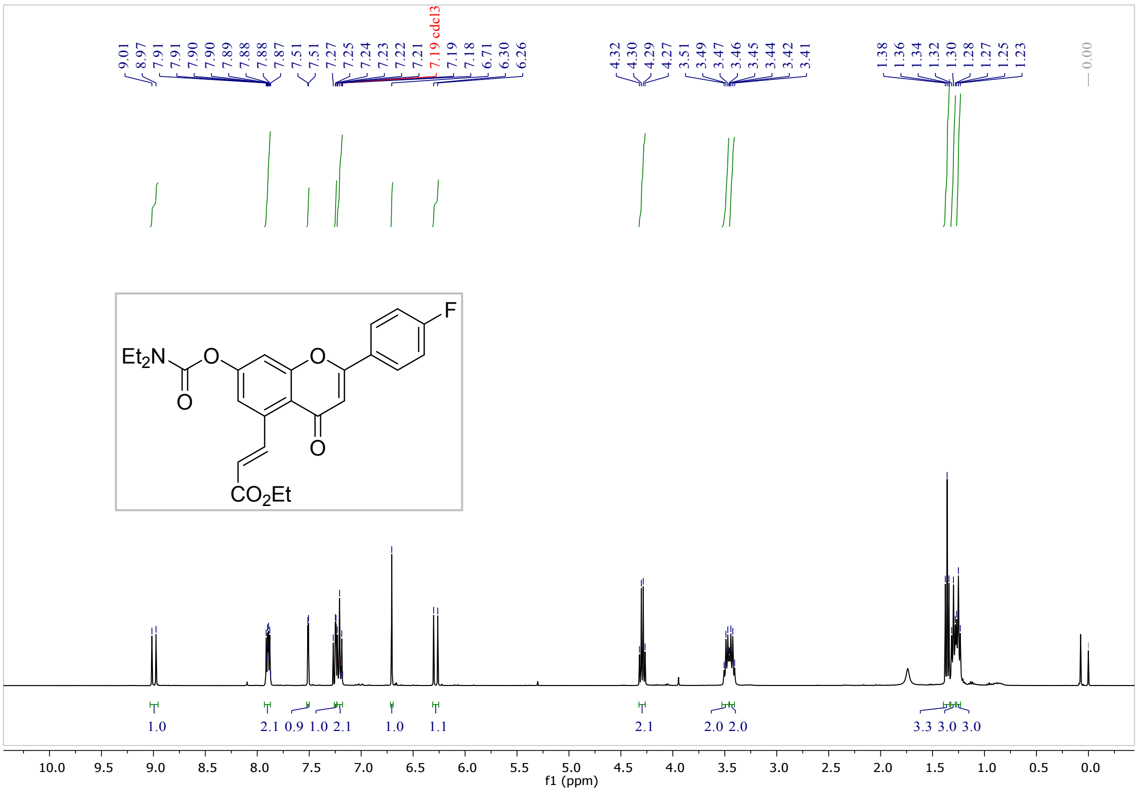

$^{13}C\{^1H\}$ NMR (101 MHz, $CDCl_3$) Spectra of Compound **11i**

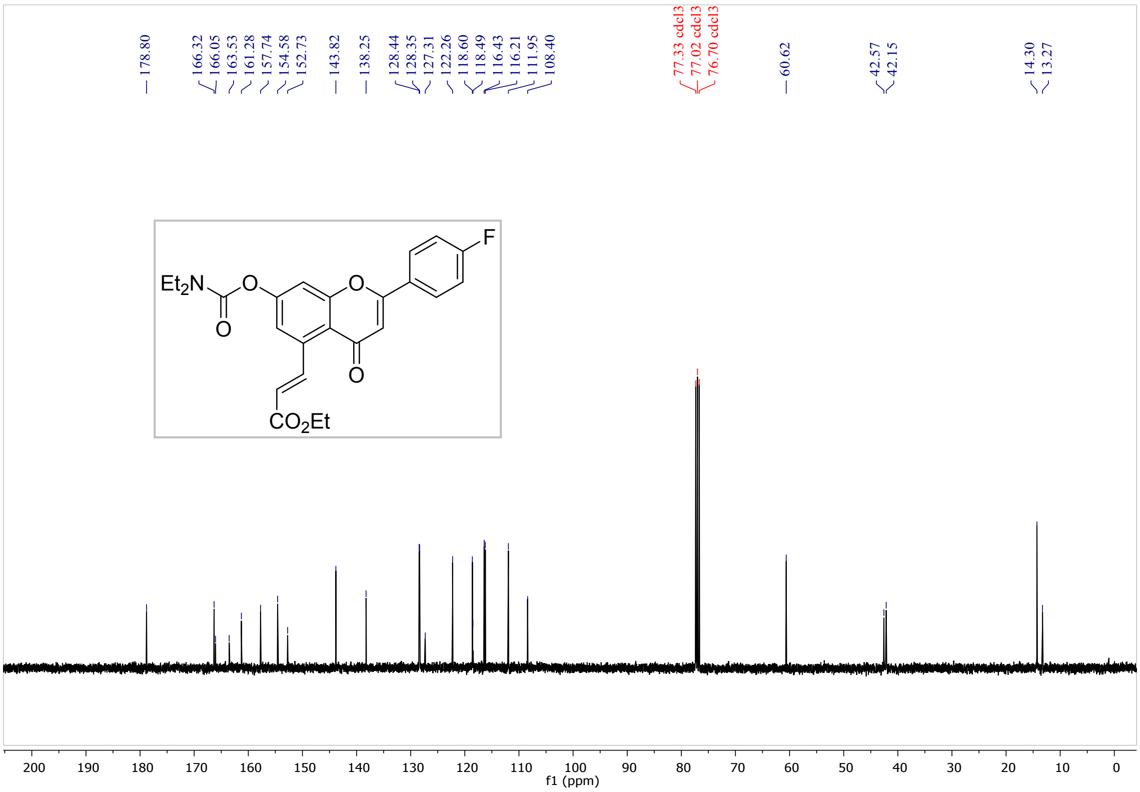

^1H NMR (500 MHz, $CDCl_3$) Spectra of Compound **11j**

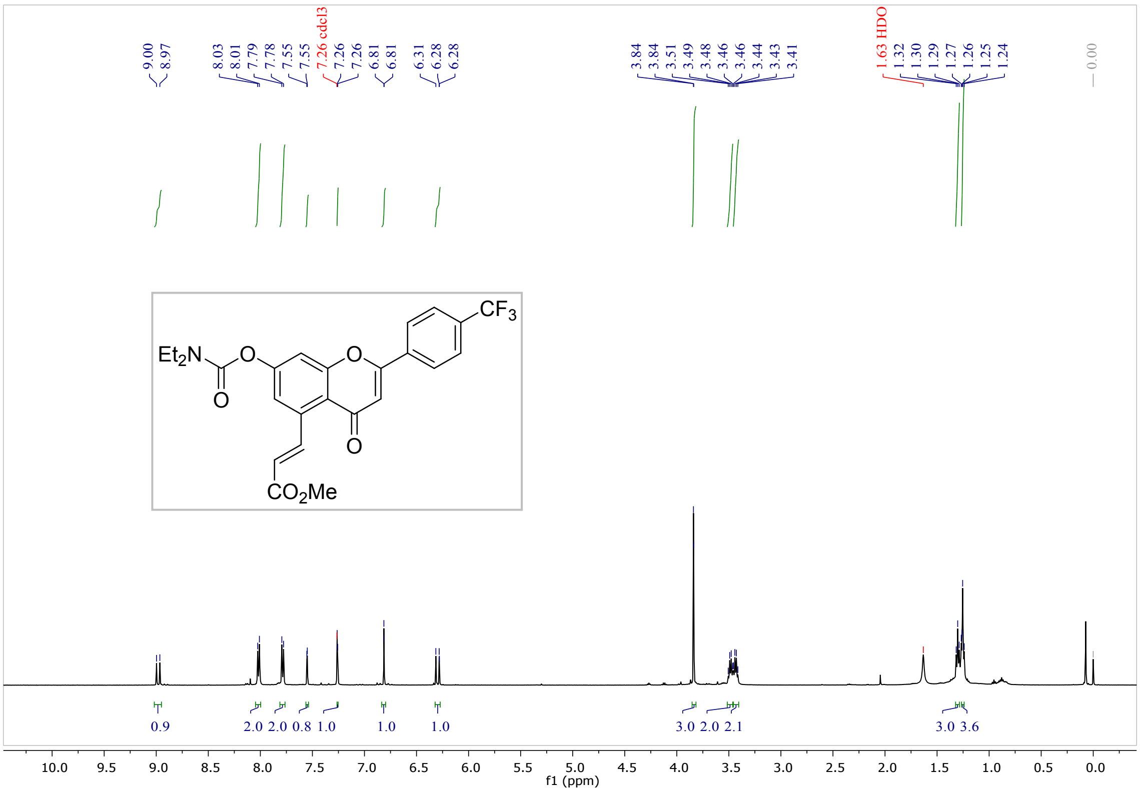

$^{13}C\{^{1}H\}$ NMR (126 MHz, $CDCl_3$) Spectra of Compound **11j**

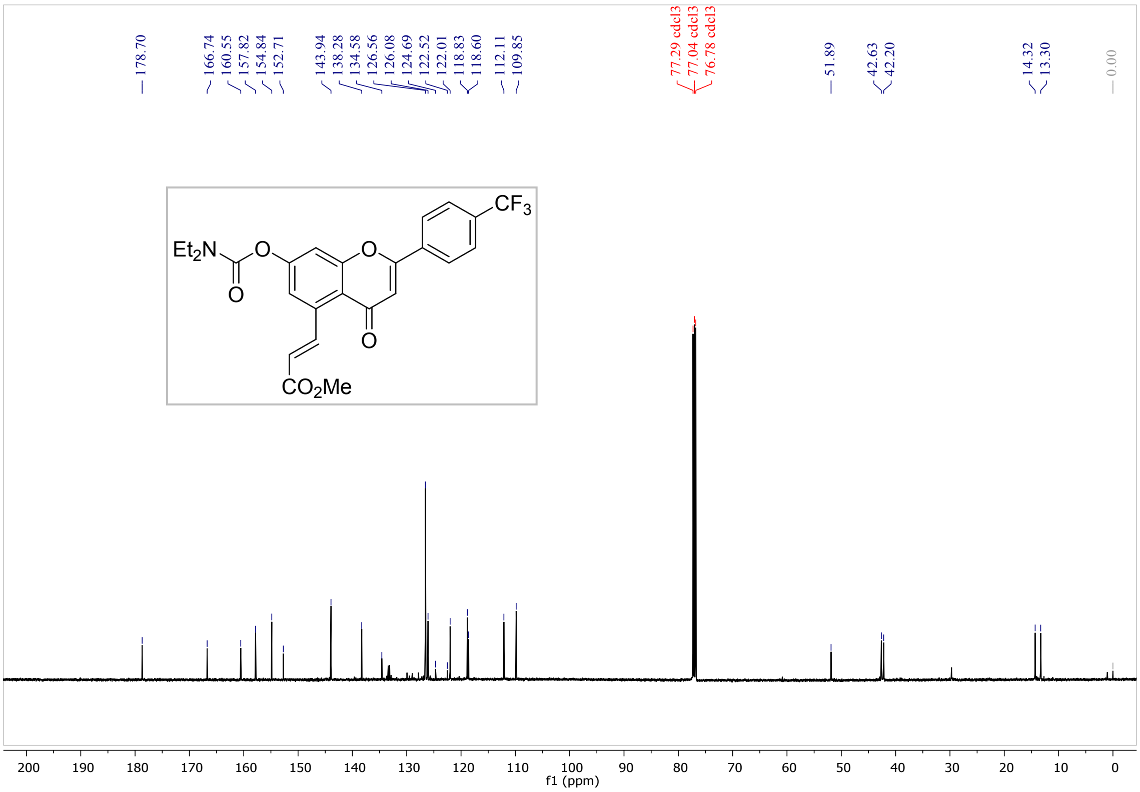

^{1}H NMR (500 MHz, $CDCl_3$) Spectra of Compound **11k**

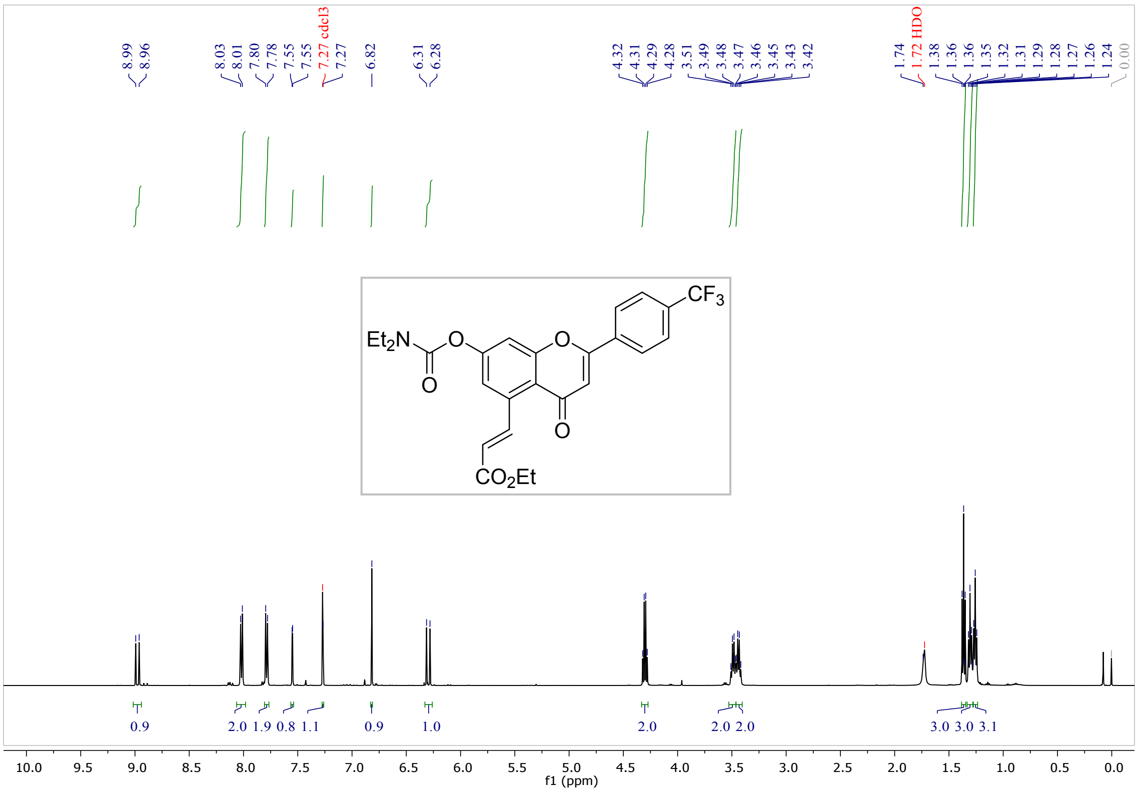

$^{13}C\{^{1}H\}$ NMR (126 MHz, $CDCl_3$) Spectra of Compound **11k**

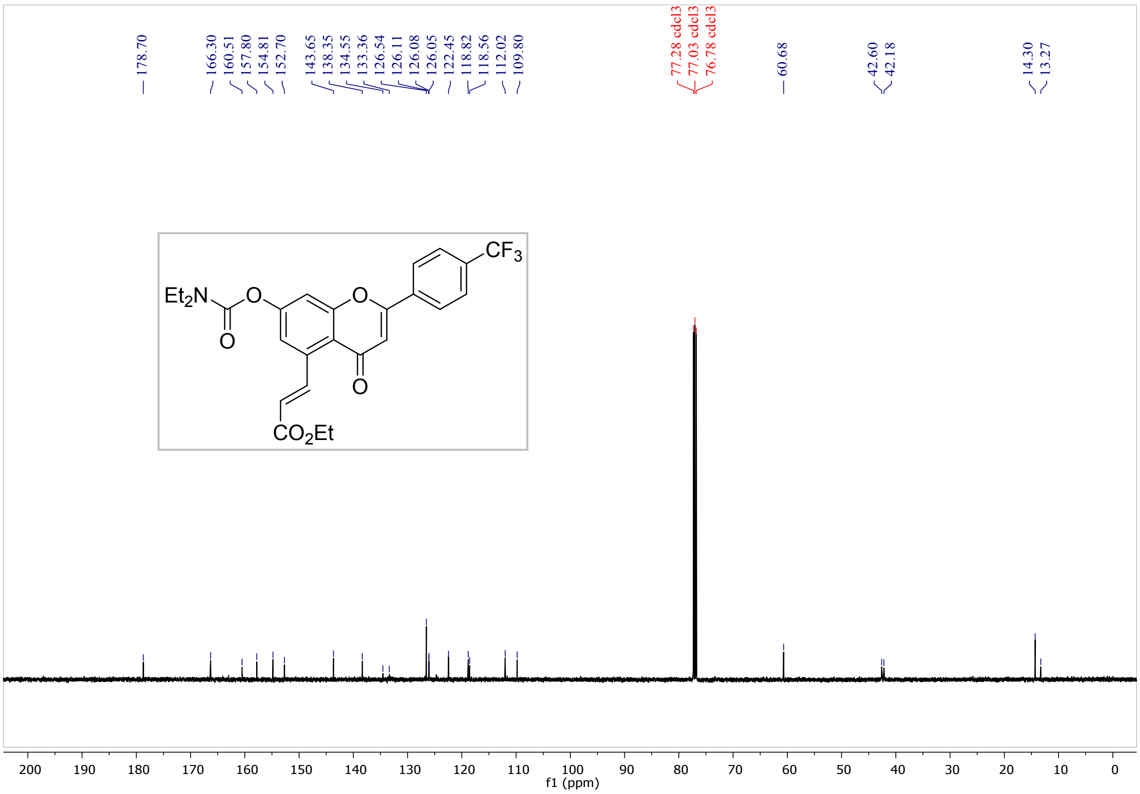

^{1}H NMR (400 MHz, $CDCl_3$) Spectra of Compound **11l**

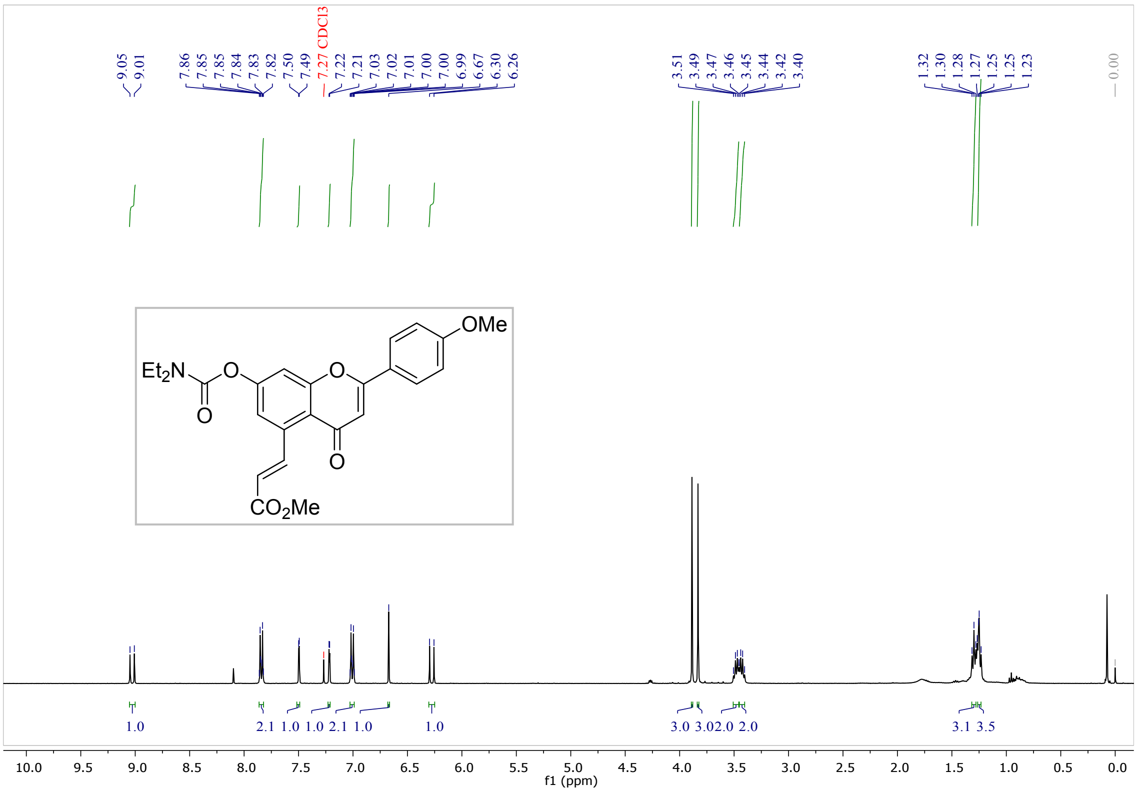

$^{13}C\{^{1}H\}$ NMR (101 MHz, $CDCl_3$) Spectra of Compound **11l**

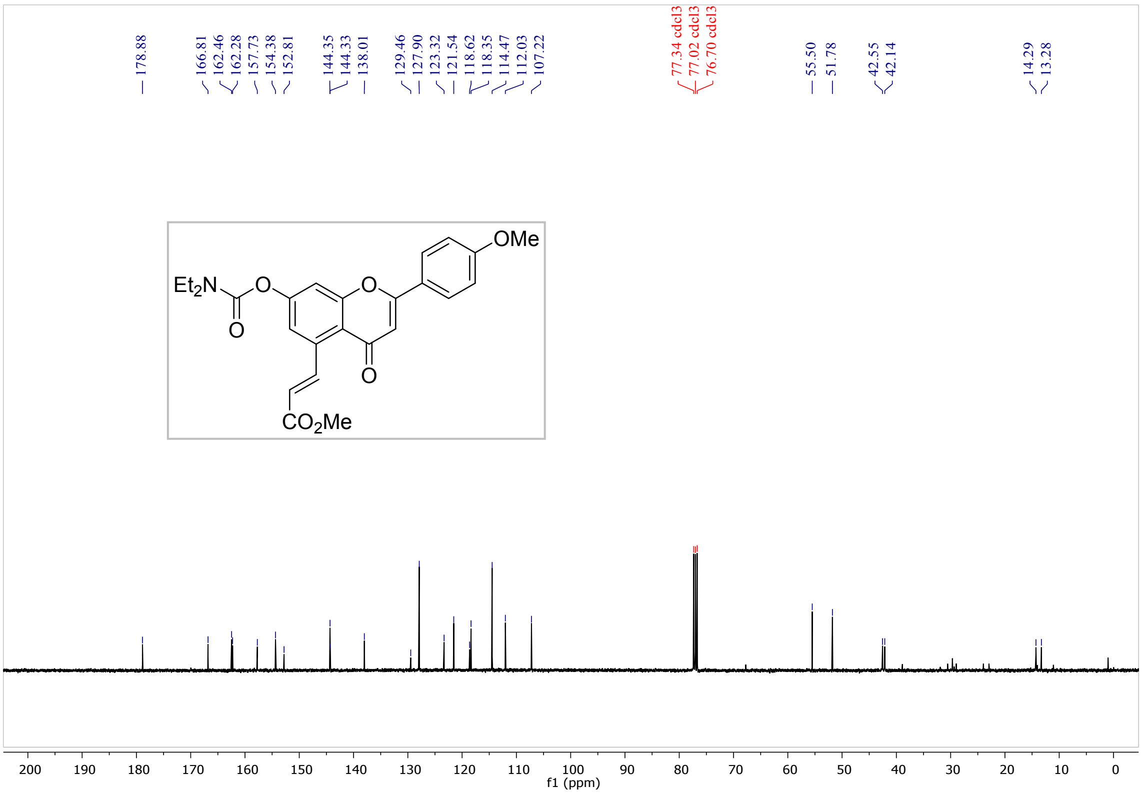

^{1}H NMR (500 MHz, $CDCl_3$) Spectra of Compound **11m**

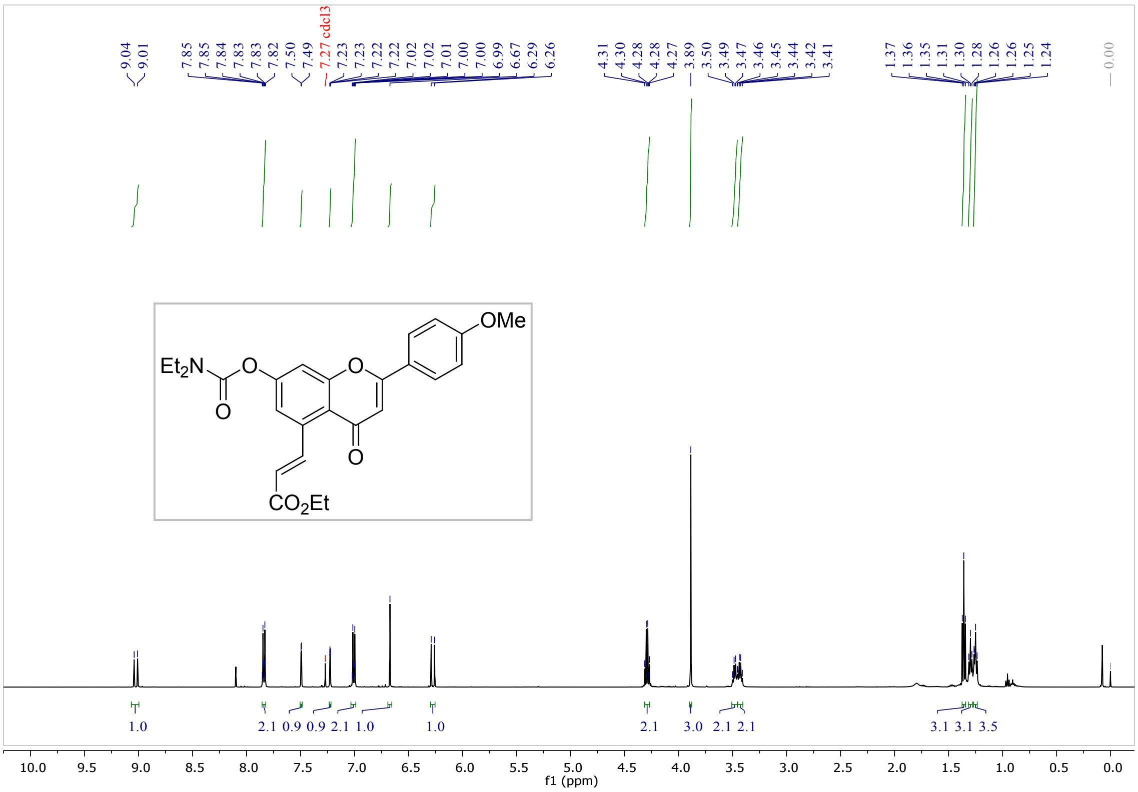

$^{13}C\{^{1}H\}$ NMR (126 MHz, $CDCl_3$) Spectra of Compound **11m**

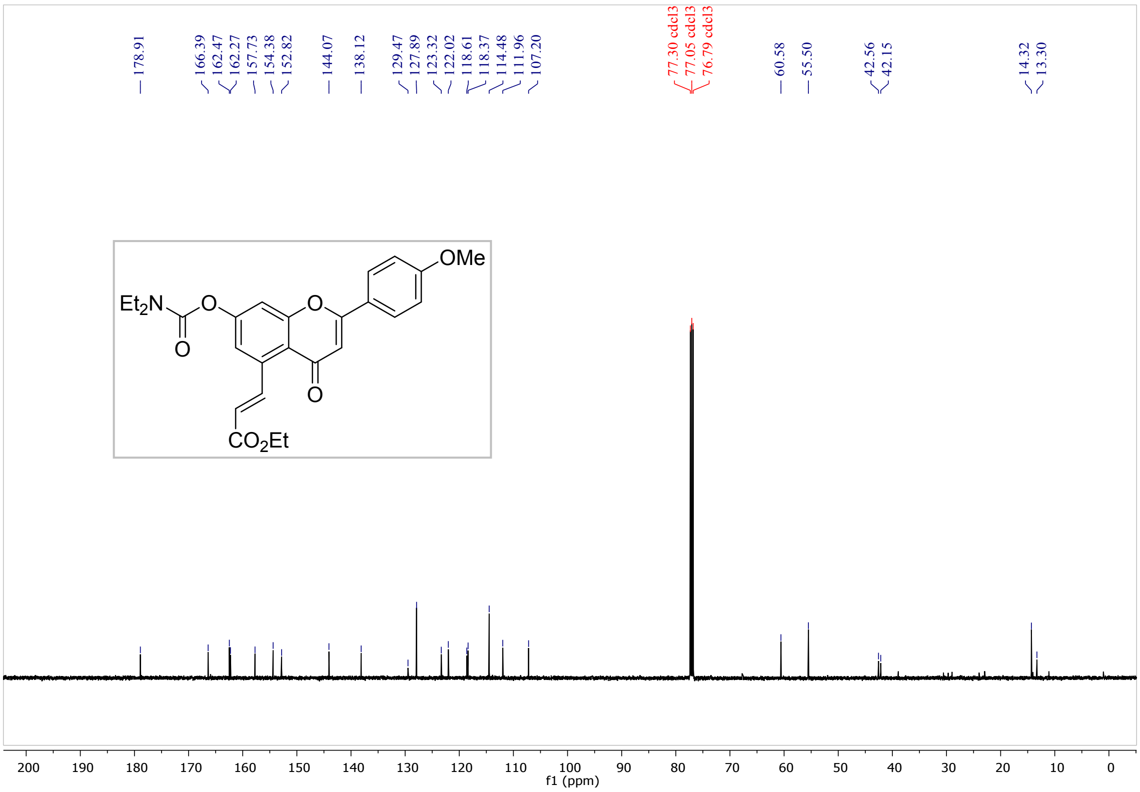

^{1}H NMR (400 MHz, $CDCl_3$) Spectra of Compound **11n**

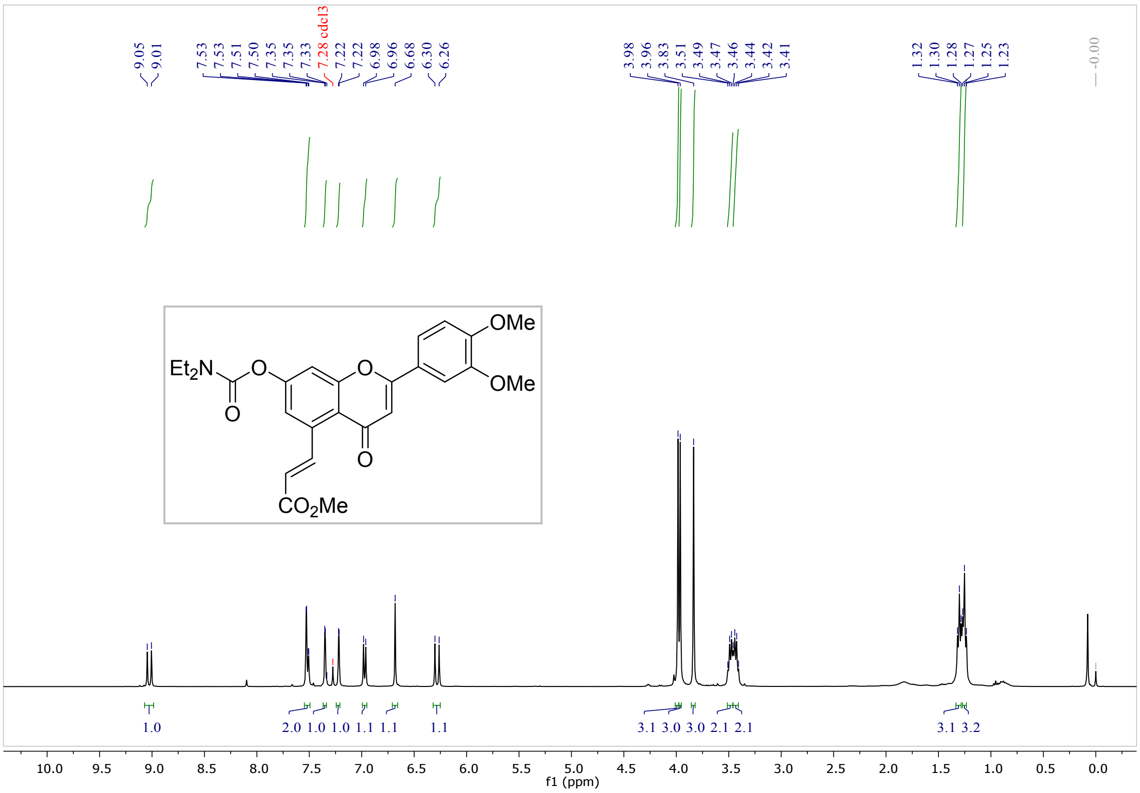

$^{13}C\{^{1}H\}$ NMR (101 MHz, $CDCl_3$) Spectra of Compound **11n**

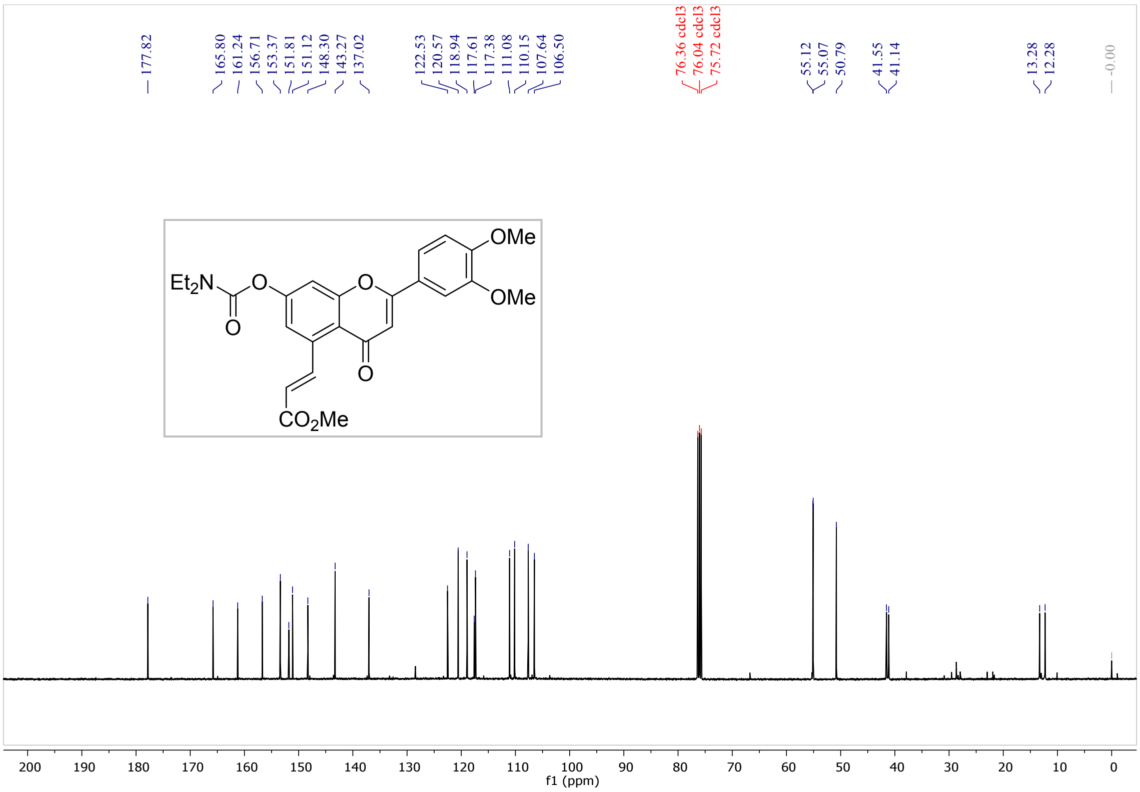

^{1}H NMR (500 MHz, $CDCl_3$) Spectra of Compound **11o**

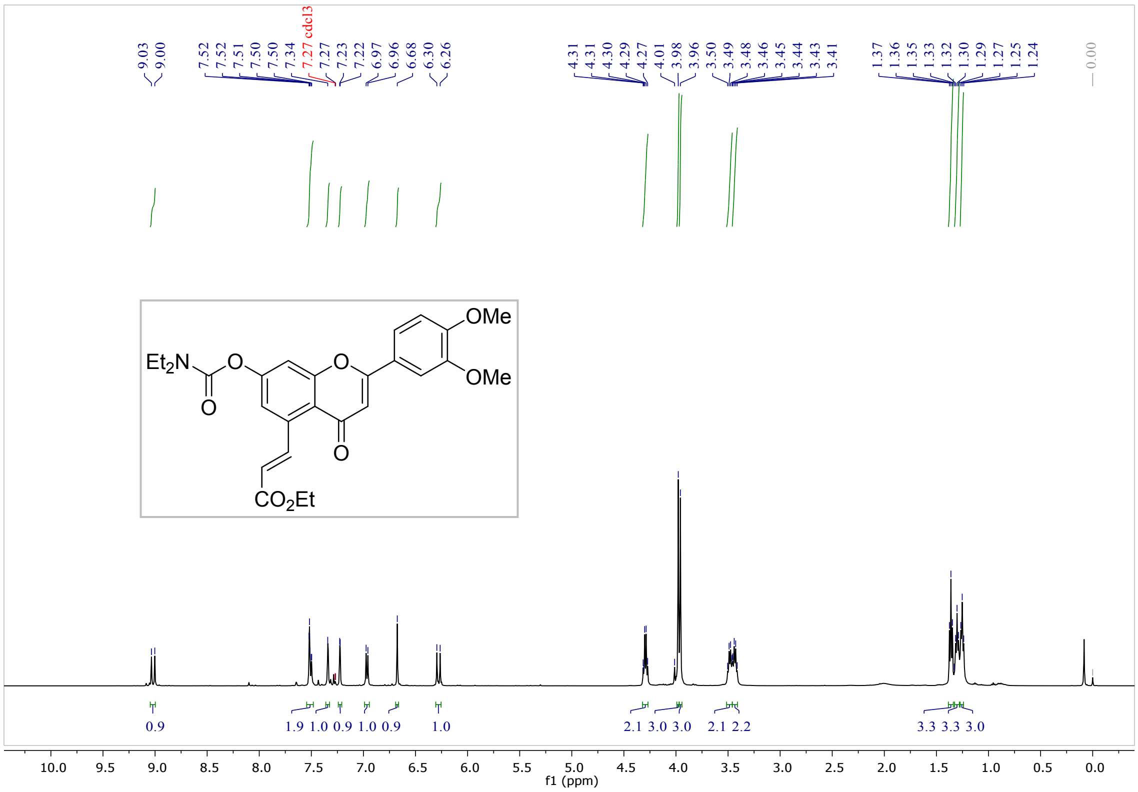

$^{13}C\{^{1}H\}$ NMR (101 MHz, $CDCl_3$) Spectra of Compound **11o**

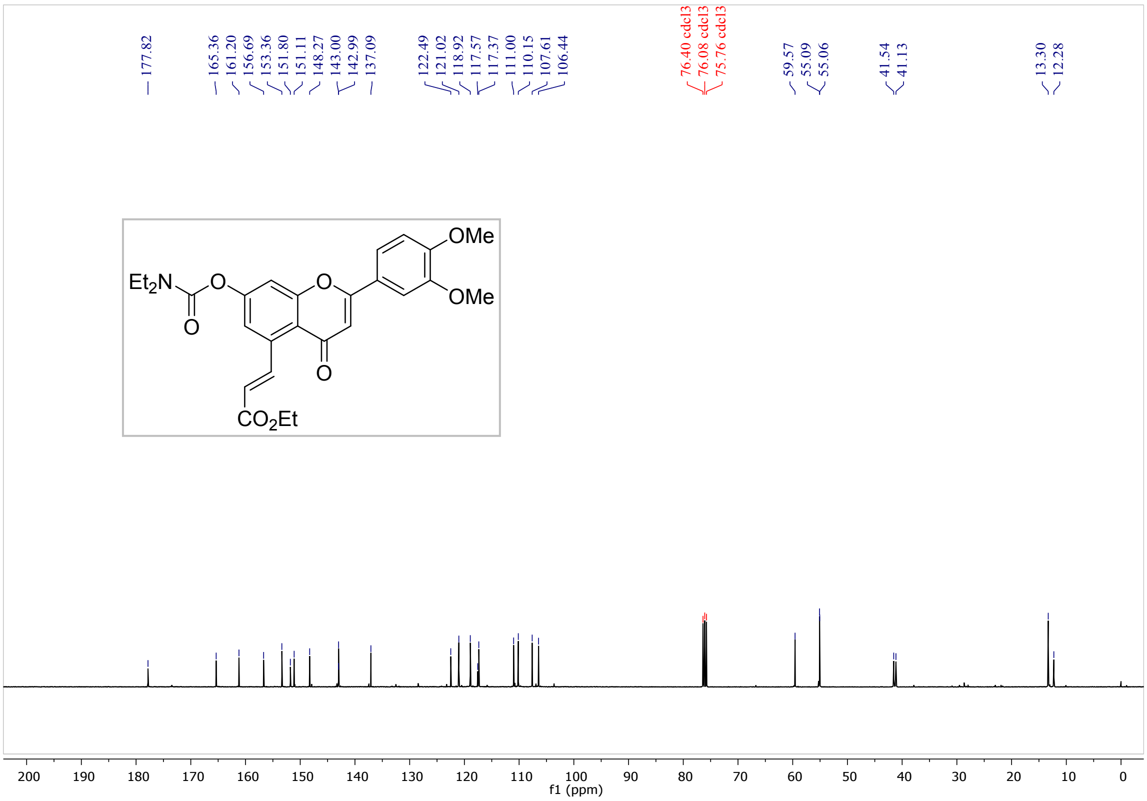

Supplement: Supplementary file 1 [file jo6c00235_si_001.pdf]
